# Nutritional supplements and cognition in healthy aging and mild cognitive impairment patients: a systematic review and network meta-analysis

**DOI:** 10.1016/j.tjpad.2026.100518

**Published:** 2026-02-28

**Authors:** Xing Liu, Chenyi Yang, Xinyi Wang, Huihui Liao, Huan Liu, Ji Ma, Yi Sun, Haiyun Wang

**Affiliations:** a The Third Central Clinical College of Tianjin Medical University, Tianjin 300170, China; b Department of Anesthesiology, Tianjin University Central Hospital, Tianjin 300170, China; c Department of Anesthesiology, Central Hospital, Tianjin University, Tianjin 300170, China; d Nankai University, Tianjin 300071, China; e Tianjin Key Laboratory of Extracorporeal Life Support for Critical Diseases, Tianjin, China

**Keywords:** Nutrient supplementation, Mild cognitive impairment, Cognitive function, Omega-3 polyunsaturated fatty acids, Blood biomarkers, Network meta-analysis

## Abstract

**Background:**

Nutritional supplementation is increasingly regarded as a potential strategy to preserve or enhance cognitive function in individuals with healthy aging and mild cognitive impairment (MCI). However, its overall efficacy remains uncertain due to inconsistent findings across clinical trials.

**Methods:**

In accordance with the guidelines outlined by the Preferred Reporting Items for Systematic Reviews and Meta-Analyses of Network Meta-Analyses, we conducted a comprehensive systematic search. Our inclusion criteria focused on randomized controlled trials (RCTs) that examined the impact of nutrient supplementation on cognitive function within healthy aging and MCI patients. The primary outcome of interest was the change in cognitive function, while the secondary outcome involved alterations in blood biochemical markers (e.g., homocysteine, vitamin B12, and serum folate levels).

**Results:**

The meta-analysis results indicated that docosahexaenoic acid (DHA)+eicosapentaenoic acid (EPA)+vitamin E+tryptophan+melatonin, as well as melatonin alone, significantly enhanced global cognitive function. Additionally, DHA, folic acid+DHA, and the DHA+EPA+vitamin E+tryptophan+melatonin were all effective in augmenting memory. DHA alone was found to be beneficial in improving processing speed, whereas vitamin D3 was associated with improvements in visuospatial function. Notably, sensitivity analyses revealed that while most domain-specific effects remained stable, the rankings of certain small-sample interventions were sensitive to study duration and sample size. Supplementation with folic acid, vitamin B12, and vitamin B6, whether administered individually or in combination, resulted in varying improvements in blood biomarkers, including homocysteine, vitamin B12, and serum folate levels.

**Conclusions:**

Nutritional supplementation demonstrates nuanced, domain-specific benefits rather than universal cognitive enhancement. While specific multi-nutrient combinations show potential, their effects are significantly influenced by baseline cognitive status, age, and intervention duration. Our findings suggest that nutritional strategies should be tailored to individual cognitive profiles, emphasizing the need for personalized interventions and further high-quality, longitudinal trials to confirm long-term clinical impact.

## 1 Introduction

The World Health Organization (WHO) projects that by 2050, the global population aged 60 and above will reach 2.1 billion, highlighting the inevitability of global aging [1]. Concurrently, the number of individuals affected by dementia is anticipated to nearly triple by 2050, with associated treatment costs projected to reach US$2.8 trillion by 2030 [2]. In light of this impending public health challenge, preventive strategies such as physical exercise [3,4], cognitive behavioral training [5], and the use of hearing aids [6,7] are crucial. Furthermore, nutrient supplementation has emerged as a straightforward, cost-effective intervention for maintaining cognitive function [8]. Directing research efforts towards individuals without dementia, including those with mild cognitive impairment (MCI), is particularly congruent with a prevention-oriented strategy, as the effectiveness of interventions is considerably limited once irreversible neurodegenerative changes have occurred [9,10].

Cognitive health in aging is modulated by multiple nutritional factors that operate through interconnected biological pathways. B vitamins are crucial in one-carbon metabolism and the regulation of homocysteine (Hcy), a metabolic byproduct of methionine metabolism, thereby influencing neuronal integrity and vascular function [11]. These effects are most consistently observed when interventions are started early, administered long-term, or targeted at individuals with low B-status or high homocysteine, rather than being a universal effect in all older adults [12,13]. In addition, omega-3 fatty acids support synaptic plasticity and exert anti-inflammatory effects; however, evidence is mixed, as randomized trials in healthy older adults show limited benefits [14,15], while several analyses report modest improvements in aged or MCI patients [16,17]. Furthermore, antioxidants play a crucial role in mitigating oxidative stress and protecting against neuronal damage. Observational studies and recent trials involving antioxidant nutrients, such as pomegranate juice and resveratrol, alongside polyphenols, suggest potential protection for specific cognitive domains (e.g., memory) and reduced dementia risk, although effects are heterogeneous and causality remains unconfirmed [18,19]. These nutrients do not function in isolation but engage in metabolic interactions, for instance, elevated Hcy can intensify oxidative stress, which may be alleviated by sufficient intake of B vitamins and antioxidants such as vitamin C, vitamin E, and polyphenols. Consequently, recent research has increasingly emphasized dietary patterns such as the Mediterranean diet, which integrates B vitamins, fatty acids, and antioxidants, offering a comprehensive framework for understanding their collective impact on cognitive aging.

Regarding its biochemical mechanism, Hcy is a metabolic byproduct generated during the metabolism of methionine, a process facilitated by S-adenosyl methionine and S-adenosylhomocysteine. Elevated levels of Hcy are strongly associated with cognitive decline and Alzheimer's disease (AD) due to their neurotoxic effects and their role in exacerbating β-amyloid deposition [20,21]. Furthermore, Hcy can be remethylated back to methionine through a methylation pathway that is dependent on cobalamin (vitamin B12). This metabolic pathway also requires essential cofactors, including vitamin B6, vitamin B12, and folate, for optimal functionality [11,22]. Consequently, monitoring blood levels of Hcy, vitamin B12, and folate provides an objective biochemical measure for assessing the efficacy of nutritional supplementation interventions on cognitive health.

The assessment of cognitive function is inherently complex, as singular tools such as the Mini-Mental State Examination (MMSE) or the Montreal Cognitive Assessment (MoCA) may fail to provide a comprehensive representation of an individual’s cognitive status [23]. Relying on these instruments in isolation can introduce bias and overlook nuanced changes within specific areas of performance. Therefore, a multidimensional approach that evaluates global cognition alongside distinct domains, which include attention, executive function, memory, processing speed, and visuospatial function, is essential. This holistic framework enhances research precision and provides a robust foundation for interpreting how interventions affect various aspects of cognitive health.

The aim of this study was to analyze published data to explore the effects of various dietary supplements (including B vitamins, omega-3 fatty acids, and melatonin) on specific cognitive function domains and related blood biochemical markers (such as homocysteine, vitamin B12, and folate levels) in individuals without dementia, with the aim of offering comprehensive, evidence-based recommendations for cognitive function enhancement within this population.

## 2 Methods

### 2.1 Basic information

The protocol for this systematic review and network meta-analysis (NMA) was prospectively registered with PROSPERO (CRD42024616019). As the study exclusively involved analysis of published aggregate data, it was exempt from requiring institutional review board approval or informed consent. We conducted this investigation in strict accordance with Cochrane Collaboration methodologies and reported findings following PRISMA-NMA guidelines (Supplementary supporting checklist). Our comprehensive search strategy, developed using the PICOS framework (detailed in eTables 1-2), was executed across four major biomedical databases (PubMed, Web of Science, Embase, and Cochrane CENTRAL) on 21 December 2024. Specifically, the search strategy across these databases utilized a combination of keywords and subject headings adapted for each database. The primary terms regarding cognitive status encompassed Alzheimer Disease, Cognitive Dysfunction, Cognition Disorders, and Healthy Volunteers. For nutritional interventions, the search focused on B-vitamins including Vitamin B Complex, Vitamin B6, Vitamin B12, Riboflavin, and Folic Acid, alongside other antioxidants such as Vitamin E and Ascorbic Acid. Additionally, Vitamin D3 24-Hydroxylase, Melatonin, and omega-3 fatty acids including Docosahexaenoic Acids and Eicosapentaenoic Acid were incorporated into the search logic. These categories were combined using Boolean operators to identify eligible studies. To ensure a thorough identification of eligible trials and minimize publication bias, this primary search was supplemented by manual scrutiny of the reference lists from all included studies. Additionally, we performed forward and backward citation tracking of relevant prior systematic reviews to identify any additional research that may have been missed during the initial electronic search.

### 2.2 Eligibility criteria

This study was restricted to randomized controlled trials to ensure the highest level of clinical evidence. Studies concerning nutritional supplements, which encompassing micronutrients (B-vitamins [B1, B2, B6, B12, folic acid], vitamins C, D3, and E, and multivitamins), omega-3 fatty acids (specifically DHA, EPA, or their combinations), and melatonin, underwent a rigorous screening process. Eligible studies included adult participants with either normal cognitive function or MCI; individuals with a clinical diagnosis of dementia were explicitly excluded. Cognitive status was determined based on the clinical diagnostic criteria reported in each study. Briefly, normal cognition was characterized by scores within age-and-education-adjusted normative ranges. MCI was defined according to established frameworks such as Petersen’s criteria, DSM-IV, or DSM-V, often supported by objective impairment on scales like the ADAS-Cog-13, SUCCAB, or WAIS-III. Additionally, inclusion required the assessment of outcomes in at least one cognitive domain, such as global cognition, attention, executive function, memory, processing speed, or visuospatial function. Where reported, data for related blood biochemical markers, including homocysteine, vitamin B12, serum folate, and erythrocyte folate, were also collected for analysis. Comprehensive details regarding the criteria are provided in eTable 3.

### 2.3 Identification of included studies

After the preliminary inclusion of potentially eligible studies, an additional screening process was conducted. Two researchers independently reviewed the titles, abstracts, and full texts of the identified articles in order to verify eligibility based on the predefined inclusion criteria.

### 2.4 Data extraction

Studies were considered eligible for inclusion if they provided an evaluation of cognitive function both prior to and following supplementation with nutritional supplements. During the data extraction process, we collected information regarding study design, participant demographics such as age and gender, and baseline cognitive status categorized as normal or mild cognitive impairment. Additionally, we recorded intervention details including the specific supplement type, dosage, and duration, alongside both cognitive and biochemical outcomes assessed at the beginning and end of each trial.

The primary outcome of this study was to evaluate changes in cognitive function, assessed across multiple domains such as global cognition, attention, executive function, memory, processing speed, and visuospatial function. In instances where multiple cognitive assessments were reported for the same domain, we prioritized the most commonly utilized test across all included studies to ensure consistency in the outcome variables. For studies reporting cognitive scores at various time points, we selected the score corresponding to the longest duration of intervention. Additionally, in scenarios where precise data for cognitive test scores were unavailable, we estimated the mean and variability based on the accessible data [24,25]. To mitigate the heterogeneity observed in the study outcomes, we exclusively incorporated the MMSE in the global cognition domain, as it was the predominant assessment tool employed across the majority of studies. This decision was informed by the varying interpretations of cognitive function outcomes in different studies; for instance, lower scores on the Alzheimer's Disease Assessment Scale-Cognitive Subscale (ADAS-cog) suggest better cognitive function, whereas higher scores on the MMSE indicate improved cognitive function. Data were extracted similarly for the remaining few domains of cognitive function.

### 2.5 Assessment of quality and risk of bias

Studies were classified as large sample size investigations if each treatment group comprised more than 100 participants [26]. The Cochrane Risk of Bias 2 (RoB2) tool was employed to systematically assess the risk of bias within the included studies, with further details provided in Supplementary Materials [27]. Additionally, the confidence in the evidence was evaluated using the web-based tool-Confidence in Network Meta­Analysis framework (CINeMA) (eTable 4).

### 2.6 Statistical analysis

In processing the data, when only pre- and post-intervention means were reported and no standard deviations were provided for these values, estimates were derived based on the maximum standard deviation observed in similar studies using the same measurement scale. When exact data for baseline and follow-up values were available, mean change values were calculated as the arithmetic differences between the baseline and follow-up means.

All analyses were performed using R (version 4.3.1) [28] along with the gemtc package (version 1.0-2) [29], which interfaces with OpenBUGS (version 3.2.3) [30] to run Markov Chain Monte Carlo simulations. A Bayesian network meta-analysis utilizing random effects and non-informative priors was conducted, incorporating placebo-controlled trials [31]. The primary analysis included all eligible trials, encompassing six cognition assessment domains and biochemical levels. For cognitive function assessment, including global cognition, attention, executive function, memory, processing speed, and visuospatial function, the standardized mean difference (SMD) was evaluated using Cohen's d with a 95% credibility interval (CrI) [32]. An SMD value of 0.20 indicates a small effect size between the experimental and control groups, 0.50 represents a moderate effect, and 0.80 signifies a large effect [33]. In addition, for biochemical analysis, the weighted mean difference (WMD) was used to assess the effects of different nutrient supplementation interventions. A random-effects model was computed using Markov chain Monte Carlo methods with Gibbs sampling based on simulations of 50,000 iterations in each of 4 chains. The homogeneity and consistency assumptions were evaluated using node splitting and the Bland-Altman method[[34], [35], [36]]. For each iteration, treatments were ranked based on their effect in comparison to an arbitrary baseline. Findings were considered as associations if the 95%CrI excluded the null value. A frequency table was created from these rankings and then normalized by the total number of iterations to calculate the rank probabilities. To evaluate the effects of various nutrient supplements on cognitive function, probability values were further summarized in this study and presented through the lower bound of the cumulative ranking curve (SUCRA) and probability ranking plots [37]. A SUCRA value of 0 indicated that a treatment was definitively the worst, while a value of 1 signified that the treatment was definitively the best. As part of the sensitivity analysis, all analyses of cognition domains were re-conducted to incorporate networks derived from studies that satisfied the following criteria: (1) studies with a mean participant age over 65 years, (2) studies involving individuals with an initial diagnosis of MCI, (3) studies with a sample size greater than 100 participants. Furthermore, to specifically evaluate the impact of long-term interventions, we performed an additional sensitivity analysis by restricting the network to studies with a duration of at least six months. These combined approaches aimed to verify the stability of our nutrient rankings under more stringent evidence criteria.

## 3 Results

**Fig. 1:**
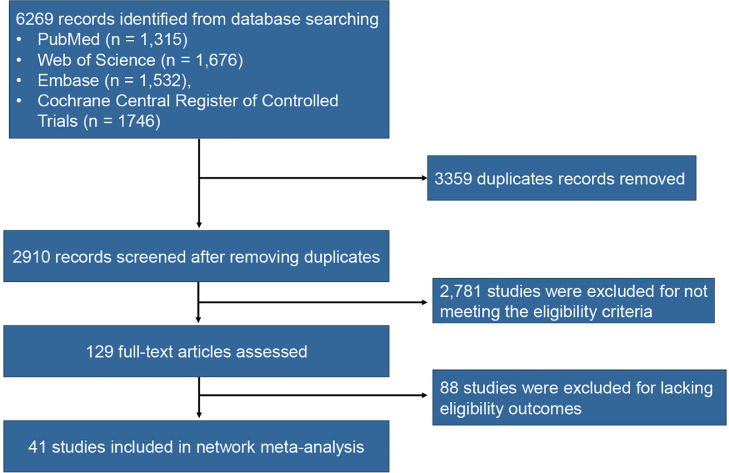
PRISMA flowchart of study selection for the network meta-analysis.

From an initial pool of 6,269 search results, 2,910 records remained after the removal of duplicates. Subsequent screening of titles and abstracts led to the selection of 129 records for full-text review. Of these, 88 studies did not satisfy the inclusion criteria, resulting in 41 RCTs deemed eligible for inclusion (Fig. 1). These RCTs investigated 13 distinct nutrients, either individually or in combination, as supplementation regimens, alongside placebo, culminating in a total of 21 treatment strategies. In total, 4,703 participants were randomized to the placebo group, while 5,258 participants were assigned to one of the active treatment groups, including vitamin B12, vitamin B2, vitamin B2+vitamin B6, vitamin B6, vitamin C+vitamin E, vitamin D3, DHA, DHA+EPA, DHA+EPA+vitamin E +tryptophan +melatonin, EPA, folic acid, folic acid+vitamin B1+vitamin B6, folic acid+vitamin B12, folic acid+vitamin B12+vitamin B2, folic acid+vitamin B12+vitamin B2+vitamin B6, folic acid+vitamin B12+vitamin B6, folic acid+vitamin D3, folic acid+DHA, melatonin, and multivitamin. Detailed study characteristics are provided in Table 1. The findings, assessed using the ROB2 evaluation criteria, indicated that all included studies exhibited a moderate or low risk of bias, with none demonstrating a high risk of bias (eFigure 1).

**Table 1:** Characteristics of included studies.

Study	Year	Country	Settings	Diagnostic criteria	No. of participants	Age, years	Male/female	Intervention	Types and dosages of nutritional supplements (per day)	Duration of intervention (years)	Cognitive domains	Biochemical analysis
Andrieu [38]	2017	France	Elderly	MMSE	380	75.1	128/252	Placebo		3 years	Global cognition (MMSE), executive function, processing speed	
					381	75.6	136/245	DHA+EPA	DHA 800mg, EPA 225 mg			
Baleztena [39]	2018	Spain	MCI	MMSE	44	87.8	30/14	Placebo		1 year	Global cognition (MMSE), attention, executive function, memory, visuospatial function	
					34	85.8	25/9	Multivitamin	DHA 750 mg, EPA 120 mg, vitamin E 15 mg, phosphatidylserine 45 mg, tryptophan 285 mg, vitamin B 12 15 μg, folate 750 μg, ginkgo biloba 180 mg			
Bo [40]	2017	China	MCI	Petersen’s criteria	42	NA	25/17	Placebo		0.5 years	Executive function, memory, processing speed, visuospatial function	
					44	NA	26/18	DHA+EPA	DHA 480 mg, EPA 720 mg			
Chiu [41]	2008	China	MCI	DSM-IV	15	76.5	8/7	Placebo		∼0.5 years	Global cognition (MMSE)	
					20	74	7/13	DHA+EPA	DHA 720 mg, EPA 1080 mg			
Dangour [42]	2015	UK	Elderly	MMSE	93	80.1	48/45	Placebo		1 year	Memory	Homocysteine, vitamin B12, serum folate
					91	79.9	46/47	Vitamin B12	Vitamin B12 1 mg			
De Jager [43]	2012	UK	MCI	Petersen’s criteria	113	76.7	40/73	Placebo		2 years	Global cognition (MMSE), executive function, memory	Homocysteine, vitamin B12, serum folate
					110	76.8	40/70	FA+Vitamin B12+Vitamin B6	Folic acid 0.8 mg, vitamin B12 0.5 mg, vitamin B6 20mg			
Duan [44]	2024	China	MCI	DSM-IV	95	69.52	35/60	Placebo		1 year	Attention, memory, visuospatial function	
					95	69.99	35/60	FA+Vitamin B1+Vitamin B6	Folic acid 180 μg, vitamin B1 0.96 mg, Vitamim B6 0.96 mg			
Durga [45]	2007	Netherlands	Elderly	MMSE	413	60	292/121	Placebo		3 years	Executive function, memory, processing speed	Homocysteine, serum folate, erythrocyte folate
					405	60	294/111	FA	Folic acid 800 μg			
Eussen [46]	2006	Netherlands	Elderly	MMSE	65	82	14/51	Placebo		∼0.5 years	Attention, executive function, memory	Homocysteine, vitamin B12, erythrocyte folate
					66	83	17/49	FA+Vitamin B12	Folic acid 400 μg, vitamin B12 1 mg			
					64	82	15/49	Vitamin B12	Vitamin B12 1 mg			
Ford [47]	2010	Australia	Elderly	MMSE	149	78.7	Man only	Placebo		2 years	Global cognition (MMSE), attention, memory, visuospatial function	Homocysteine
					150	79.3	Man only	FA+Vitamin B12+Vitamin B6	Folic acid 200 mg, vitamin B12 400 μg, Vitamim B6 25 mg			
Hu [48]	2018	China	MCI	Petersen’s criteria	82	66.6	32/50	Placebo		1 year	Attention, visuospatial function	
					93	67.22	43/50	Vitamin D3	Vitamin D3 400 IU			
Kang [49]	2008	USA	Elderly	TICS	532	72.5	Female only	Placebo		6.6 years	Global cognition (TICS), executive function, memory	
					521	72.5	Female only	FA+Vitamin B12+Vitamin B6	Folic acid 2.5 mg, vitamin B6 50 mg, vitamin B12 1 mg			
Kwok, [50]	2017	China	Elderly	MMSE	116	75.84	NA	Placebo		2.25 years	Global cognition (NTB-changed), executive function, memory, processing speed	Homocysteine, vitamin B12, serum folate
					120	74.67	NA	Vitamin B12	Methylcobalamin 1000 mg			
Kwok, [51]	2020	China	MCI	Petersen's criteria	120	78	NA	Placebo		2 years	Executive function, memory	Homocysteine, serum folate
					118	76.9	NA	FA+Vitamin B12	Folic acid 400 μg, methylcobalamin 500 μg			
Lee [52]	2013	Malaysia	MCI	MMSE	18	63.5	5/13	Placebo		1 year	Global cognition (MMSE), attention, executive function, memory, processing speed, visuospatial function	
					17	66.4	3/14	DHA+EPA	DHA 430 mg, EPA 150 mg			
Lewerin [53]	2005	Sweden	Elderly	NA	62	75.6	27/35	Placebo		∼0.33 years	Executive function, memory, visuospatial function	
					113	75.7	48/65	FA+Vitamin B12+Vitamin B6	Folic acid 800 μg, cyanocobalamin 500 μg, vitamin B6 3 mg,			
Li [54]	2020	China	MCI	DSM-V	60	70.38	27/33	Placebo		0.5 years	Attention, memory, visuospatial function	Homocysteine, serum folate
					60	70.2	24/36	FA	Folic acid 800 μg			
					60	70.33	24/36	FA+DHA	Folic acid 800 μg, DHA 800 mg			
					60	71.15	24/36	DHA	DHA 800 mg			
Lin [55]	2022	China	MCI	MMSE	34	78.1	25/9	Placebo		2 years	Global cognition (MMSE)	
					41	78.95	29/12	DHA	DHA 700 mg			
					40	77.8	27/13	EPA	EPA 1600 mg			
					42	76.73	27/15	DHA+EPA	DHA 350 mg, EPA 800 mg			
Liu [56]	2024	China	MCI	Petersen's criteria	135	62.4	49/86	Placebo		∼0.5 years	Global cognition (MoCA), attention, memory, processing speed	Homocysteine, serum folate
					134	62.8	56/78	FA	Folic acid 400 μg			
					133	62.6	64/69	FA+Vitamin D3	Folic acid 400 μg, vitamin D3 1600 IU			
Ma [57]	2019	China	MCI	MMSE	90	74.63	38/52	Placebo		2 years	Attention, executive function, memory, visuospatial function	Homocysteine, vitamin B12, serum folate
					90	74.82	39/51	FA	Folic acid 400 μg			
Mahmoudi [58]	2014	Iran	Elderly	MMSE	66	75.17	30/36	Placebo		∼0.5 years	Global cognition (MMSE)	
					70	74.13	31/39	DHA+EPA	DHA 180 mg, EPA 120 mg			
Maltais [59]	2022	Canada	Healthy adults	MMSE	97	50.5	34/63	Placebo		0.5 years	Executive function, memory, visuospatial function	
					96	49.4	30/66	DHA+EPA	DHA 800 mg, EPA 1700 mg			
McMahon [60]	2006	New Zealand	Elderly	MMSE	126	73.4	61/65	Placebo		2 years	Global cognition (MMSE), executive function, memory	Homocysteine, vitamin B12, serum folate
					127	73.6	80/47	FA+Vitamin B12+Vitamin B6	Folic acid 1000 μg,vitamin B12 500μg, vitamin B6 10 mg			
Mengelberg [61]	2022	New Zealand	MCI	RBANS	30	73.4	11/19	Placebo		1 year	Global cognition (Rtot), attention, executive function, memory, visuospatial function	
					30	72.33	14/16	DHA+EPA	DHA 1491 mg, EPA 351 mg			
Montero-Odasso [62]	2023	Canada	MCI	ADAS-Cog-13	35	72.4	19/16	Placebo		0.5 years	Attention, executive function, memory	
					34	73.1	13/21	Vitamin D3	Vitamin D3 4258 IU			
Naeini [63]	2014	Iran	MCI	MMSE	119	66.3	57/62	Placebo		1 year	Global cognition (MMSE)	
					127	66.5	63/64	Vitamin C+Vitamin E	Vitamin C 400 mg, vitamin E 300 mg			
Pathansali [64]	2003	UK	Elderly	MMSE	12	73.8	10/2	Placebo		∼0.08 years	Attention, memory	Homocysteine, vitamin B12, serum folate
					12	72.3	11/1	FA	Folic acid 5 mg			
Perła-Kaj´an [65]	2021	USA	MCI	MMSE	101	NA	NA	Placebo		2 years	Executive function, memory, processing speed	Homocysteine
					95	NA	NA	FA+Vitamin B12+Vitamin B6	Folic acid 0.8 mg, vitamin B12 0.5 mg, vitamin B6 20 mg			
Phillips [66]	2015	UK	MCI	MMSE	39	71.1	18/21	Placebo		∼0.33 years	Global cognition (MMSE), memory	
					37	71.1	16/21	DHA+EPA	DHA 625 mg, EPA 600 mg			
Pipingas [67]	2014	Australia	Young adults	SUCCAB	60	31.6	28/32	Placebo		∼0.33 years	Memory	Homocysteine, vitamin B12, erythrocyte folate
					56	31.8	24/32	Multivitamin	Multivitamin			
Rondanelli [68]	2012	Italy	MCI	MMSE	14	86.1	3/11	Placebo		0.25 years	Global cognition (MMSE), attention, executive function, memory, visuospatial function	
					11	85.3	2/9	DHA+EPA+Vitamin E+Trp+Mel	DHA 720 mg, EPA 286 mg, vitamin E 16 mg, tryptophan 95 mg, melatonin 5 mg			
Stott [69]	2005	Netherlands	Elderly	TICS	24	72.8	14/10	Placebo		1 year	Global cognition (TICS), processing speed	
					23	74.6	10/13	Vitamin B2	Vitamin B2 25 mg			
					23	72.9	12/11	FA+Vitamin B12	Folic acid 2.5 mg, vitamin B12 400 μg			
					23	74.7	11/12	Vitamin B6	Vitamin B6 25 mg			
					23	76.5	10/13	FA+Vitamin B12+Vitamin B2	Folic acid 2.5 mg, vitamin B12 400 μg, vitamin B2 25 mg			
					23	72.6	12/11	FA+Vitamin B12+Vitamin B6	Folic acid 2.5 mg, vitamin B12 400 μg, vitamin B6 25 mg			
					23	74	9/14	FA+Vitamin B12+Vitamin B2+Vitamin B6	Folic acid 2.5 mg, vitamin B12 400 μg, vitamin B2 25 mg, vitamin B6 25 mg			
					23	74.2	10/13	Vitamin B2+Vitamin B6	Vitamin B2 25 mg, vitamin B6 25 mg			
van der Zwaluw [70]	2014	Netherlands	Elderly	MMSE	431	72.6	255/176	Placebo		2 years	Global cognition (MMSE), attention, executive function, memory, processing speed	
					425	72.6	244/181	FA+Vitamin B12	Folic acid 400 μg, vitamin B12 500 μg			
van Uffelen [71]	2008	Australia	MCI	MMSE	74	75	41/33	Placebo		1 year	Global cognition (MMSE), executive function, memory	
					78	75	44/34	FA+Vitamin B12+Vitamin B6	Folic acid 5 mg, vitamin B12 0.4 mg, vitamin B6 50 mg			
Vauzour [72]	2023	UK	MCI	MoCA	121	65	51/70	Placebo		1 year	Global cognition (MoCA), attention, executive function, memory	
					94	66	42/52	DHA+EPA	DHA 1.1g, EPA 0.4g			
Wolters [73]	2005	Germany	Elderly	WAIS-III	109	64	NA	Placebo		0.5 years	Processing speed	
					111	63	NA	Multivitamin	Multivitamin			
Xu [74]	2020	China	MCI	MMSE	39	66.5	21/18	Placebo		0.5 years	Global cognition (MMSE)	
					40	66.6	21/19	Melatonin	Melatonin 0.15 mg/kg			
Yang [75]	2020	China	MCI	Petersen's criteria	90	66.6	39/51	Placebo		1 year	Attention, processing speed, visuospatial function	
					93	67.2	43/50	Vitamin D3	Vitamin D3 800 IU			
Yurko-Mauro [76]	2010	USA	Elderly	MMSE	218	70	87/131	Placebo		∼0.5 years	Global cognition (MMSE), memory	
					219	70	96/123	DHA	DHA 900 mg			
Zhang, [77]	2018	China	MCI	Petersen's criteria	120	73.6	42/78	Placebo		2 years	Attention, processing speed, visuospatial function	
					120	73.7	43/77	DHA	DHA 2 g			
Zhang, [78]	2017	China	MCI	Petersen's criteria	120	74.6	41/79	Placebo		1 year	Attention, processing speed, visuospatial function	
					120	74.5	43/77	DHA	DHA 2 g			

ADAS-Cog-13, Alzheimer's Disease Assessment Scale-Cognitive Subscale 13, B1, vitamin B1, B2, vitamin B2, B6, vitamin B6, B12, vitamin B12, C, vitamin C, D3, vitamin D3, DHA, docosahexaenoic acid, DSM-IV, Diagnostic and Statistical Manual of Mental Disorders, Fourth Edition, EPA, eicosapentaenoic acid, FA, folic acid, MCI, mild cognitive impairment, Mel, melatonin, MMSE, Mini-Mental State Examination, MoCA, Montreal Cognitive Assessment, NA, not available, RBANS, Repeatable Battery for the Assessment of Neuropsychological Status, SUCCAB, Swinburne University Computerized Cognitive Assessment Battery, TICS, Telephone Interview for Cognitive Status, Trp, tryptophan, WAIS-III, Wechsler Adult Intelligence Scale, Third Edition.

### 3.1 Global cognition

**Fig. 2:**
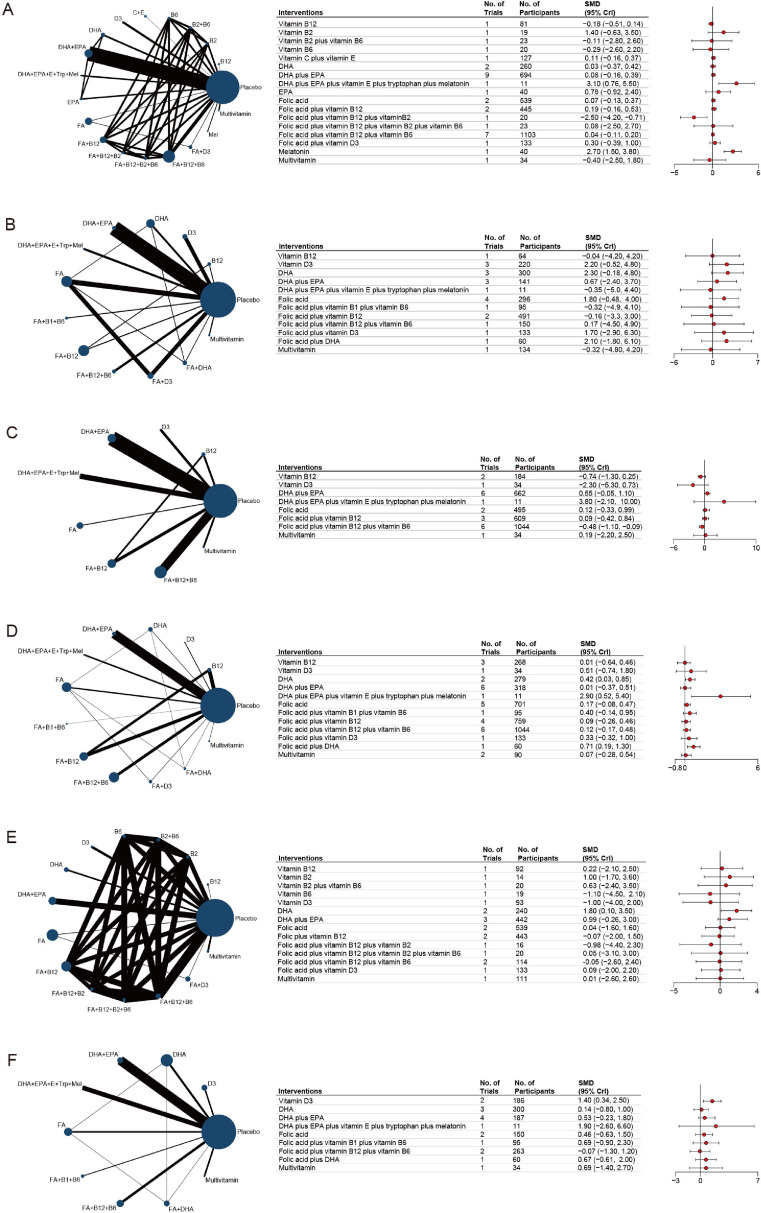
**Network meta-analysis diagram evaluating nutritional supplements across cognitive domains.** A, global cogniton. B, attention. C, executive function. D, memory. E, processing speed. F, visuospatial function. Node size scales with the number of trials per intervention; edge thickness represents the quantity of direct comparative trials between treatments. Standardized mean differences (SMDs) with 95% credibility intervals shown in the adjacent table represent the comparative efficacy of each intervention against the placebo as the common reference point.

Changes in global cognition, primarily reflecting the overall impact of nutrient supplementation on cognitive function in individuals without dementia, were predominantly assessed using the MMSE. This analysis included 25 studies, encompassing 18 treatments regimens and a total of 6,919 participants. Findings from the network meta-analysis indicated that, compared to placebo, nutrient supplementation significantly improved global cognitive functioning when administered either as melatonin alone (SMD: 2.70; 95% CI: 1.50 to 3.80; Fig. 2A) or as a combination of DHA, EPA, vitamin E, tryptophan, and melatonin (SMD: 3.10; 95% CI: 0.76 to 5.50; Fig. 2A). The effects of other supplementation regimens on global cognition varied. Among all interventions, DHA+EPA+vitamin E+tryptophan+melatonin exhibited the highest probability of being the most effective treatment (SUCRA = 0.957; eFigure 2C), followed closely by melatonin (SUCRA = 0.945) and vitamin B2 (SUCRA = 0.771; eFigure 2C). However, both interventions were each supported by only a single study. Among the treatments, DHA+EPA had the highest number of included trials (9 studies, 694 participants), while folic acid+vitamin B12+vitamin B6 had the largest sample size (7 studies, 1,103 participants). The observed effects on global cognition for these interventions were modest (DHA+EPA, SMD: 0.08; 95% CI: -0.16 to 0.39; SUCRA = 0.477; folic acid+vitamin B12+vitamin B6, SMD: 0.04; 95% CI: -0.11 to 0.20; SUCRA = 0.421; Fig. 2A). The SUCRA values and rank probability plots are presented in eFigure 2C-D of the Supplementary Materials. The certainty of evidence for the treatment measures, as evaluated using the CINeMA framework, was rated as low or very low (eTable 3A).

To further investigate the effects of nutrient supplementation on global cognition in specific subpopulations, additional stratified analyses were performed. Among participants aged over 65 years (eFigure 3A-D) and those with MCI (eFigure 4A-C), the results were generally consistent with the overall findings. However, when the analysis was restricted to studies with large sample sizes (eFigure 5A-C), no treatment demonstrated statistically significant efficacy in improving global cognition compared to placebo. Nonetheless, ranking results suggested that supplementation with folic acid+vitamin D3 may offer a relatively greater effect.

### 3.2 Attention

Attention, a fundamental cognitive domain, reflects an individual's ability to focus, sustain, and shift mental resources, serving as a foundation for higher-order cognitive functions. In our review, the cognitive domain of attention was primarily assessed using the trail making test-A, encompassing a total of 18 studies, 13 treatment regimens, and 3,692 participants. Results from the network meta-analysis revealed that none of the interventions significantly improved participants' attention levels compared to placebo (Fig. 2B). SUCRA analysis suggested that DHA supplementation (3 studies, 220 participants, SUCRA=0.769) and vitamin D3 supplementation (3 studies, 300 participants, SUCRA=0.742) appeared to be relatively effective approaches (eFigure 6A-D). The certainty of evidence, as evaluated using CINeMA, was rated as low or very low for all treatment measures (eTable 3B). Subgroup analyses conducted on participants older than 65 years and those with MCI revealed no significant improvements in attention across the treatment regimens. Nevertheless, DHA consistently emerged as a potentially beneficial supplementation strategy (eFigure 7A-D, 8A-D). Notably, when small-sample studies were excluded, DHA demonstrated a significant improvement in attention levels (SMD, 3.70; 95% CI, 0.72 to 6.50; SUCRA=0.921, eFigure 9A-C).

### 3.3 Executive function

Executive function assessment plays a crucial role in cognitive evaluation as it helps assess an individual's ability in planning, decision-making, problem-solving, and impulse control. In this study, executive function was primarily evaluated by verbal fluency test, and a total of 22 studies, 9 treatments, and 6097 participants were included. Network meta-analysis results revealed that none of the treatments significantly improved participants' executive function (Fig. 2C). However, supplementation with folic acid+vitamin B12+vitamin B6 appeared to have a negative effect on executive function (SMD: -0.48; 95% CI: -1.10 to -0.09). According to the SUCRA analysis, combined supplementation with DHA, EPA, vitamin E, tryptophan, and melatonin emerged as the most effective intervention (SUCRA = 0.903, eFigure 10A-D). The CINeMA evaluation indicated that the certainty of evidence was uniformly low (eTable 3C). In participants aged over 65 years, the effects of these treatments on executive function were consistent with the overall findings (eFigure 11A-D). Conversely, in participants with MCI, none of the treatments demonstrated a significant effect on executive function (eFigure 12A-C). Furthermore, after excluding small sample-size studies, no treatment regimen significantly improved executive function (eFigure 13A-C).

### 3.4 Memory

Memory assessment in cognitive evaluation measures an individual’s ability to encode, store, and retrieve information, reflecting essential cognitive functions for daily activities. The primary assessment tool for memory was the Auditory Verbal Learning Test (AVLT), encompassing 29 studies, 13 treatment regimens, and 7,267 participants. Network meta-analysis results revealed that, compared with placebo, DHA (SMD, 0.42; 95% CI, 0.03 to 0.85), DHA+EPA+vitamin E+tryptophan+melatonin (SMD, 2.90; 95% CI, 0.52 to 5.40), and folic acid+DHA (SMD, 0.71; 95% CI, 0.19 to 1.30) significantly improved memory performance (Fig. 2D). SUCRA analysis demonstrated SUCRA values of 0.98, 0.83, and 0.675 for these three treatment regimens, respectively (eFigure 14A-D). However, both DHA+EPA+vitamin E+tryptophan+melatonin, as well as folic acid+DHA, were evaluated in only one study each, and the findings for DHA+EPA+vitamin E+tryptophan+melatonin were limited by wide 95% CrIs. CINeMA assessments indicated that the certainty of evidence was low or very low across all regimens (eTable 3D). Subgroup analysis of participants aged over 65 years showed results consistent with the overall findings (eFigure 15A-D). When analyses were restricted to individuals with MCI, only DHA+EPA+vitamin E+tryptophan+melatonin demonstrated a significant improvement in memory among the 12 evaluated regimens (eFigure 16A-C). Further sensitivity analysis, including only large-sample studies, revealed that none of the six treatment regimens significantly outperformed placebo in enhancing memory. Folic acid+vitamin D3 appeared to demonstrate a relatively better effect (eFigure 17A-C).

### 3.5 Processing speed

The processing speed domain assesses an individual's ability to quickly and accurately process simple cognitive tasks, reflecting efficiency in mental operations. The primary criterion for evaluating processing speed was the Digital Symbol Test, with a total of 13 studies, 15 treatment regimens, and 4,365 participants included. Network meta-analysis revealed that DHA supplementation alone significantly improved participants' processing speed compared to placebo (SMD, 1.80; 95% CI, 0.10 to 3.50; Fig. 2E), with a SUCRA value of 0.891 (eFigure 18A-D). This was followed by DHA+EPA (SMD, 0.99; 95% CI, -0.26 to 3.00; SUCRA = 0.761). The CINeMA evaluation indicated a high certainty of evidence for DHA supplementation compared to placebo (eTable 3E), while DHA+EPA demonstrated a moderate certainty of evidence. The remaining treatment comparisons were rated as low or very low (eTable 3E). In subgroup analyses focusing on participants older than 65 years, DHA supplementation continued to show significant benefits (SMD, 1.80; 95% CI, 0.05 to 3.60; SUCRA = 0.914; eFigure 19A-C). However, among individuals with MCI, none of the six evaluated treatment regimens demonstrated a statistically significant effect on processing speed (eFigure 20A-C). When restricting the analysis to studies with large sample sizes, DHA supplementation once again showed a significant improvement in processing speed (SMD, 1.80; 95% CI, 0.72 to 2.80; SUCRA = 0.869; eFigure 21A-C), further supporting its efficacy in enhancing cognitive processing efficiency.

### 3.6 Visuospatial function

The visuospatial function domain assesses an individual's ability to perceive, interpret, and manipulate visual and spatial information, which is essential for tasks like navigation and object recognition. Visuospatial function was primarily evaluated using the Block Design test, with a total of 15 studies, 10 treatment regimens, and 2,405 participants included. Network meta-analysis revealed that vitamin D3 supplementation significantly improved visuospatial function compared to placebo (SMD, 1.40; 95% CI, 0.09 to 3.50; Fig. 2F, eFigure 22A-D). The SUCRA value for vitamin D3 was 0.842, indicating a high likelihood of being the most effective treatment. The remaining eight interventions varied in their impact on visuospatial function. The CINeMA evaluation demonstrated a high certainty of evidence for vitamin D3 supplementation compared to placebo (eTable 3F). In subgroup analyses focusing on participants older than 65 years, vitamin D3 supplementation (SMD, 1.50; 95% CI, 0.80 to 2.10; SUCRA = 0.88; eFigure 23A-C) and folic acid+DHA supplementation (SMD, 0.68; 95% CI, 0.02 to 1.30; SUCRA = 0.628; eFigure 23A-C) both showed significant improvements in visuospatial function. Furthermore, analyses restricted to individuals with MCI demonstrated that both vitamin D3 supplementation (SMD, 1.40; 95% CI, 0.67 to 2.20; SUCRA = 0.798; eFigure 24A-C) and DHA+EPA supplementation (SMD, 1.60; 95% CI, 0.42 to 2.80; SUCRA = 0.826; eFigure 24A-C) significantly improved visuospatial function. When focusing on studies with larger sample sizes, only two treatment regimens remained, and neither demonstrated a significant effect on visuospatial function (eFigure 25A-C).

### 3.7 Homocysteine

**Fig. 3:**
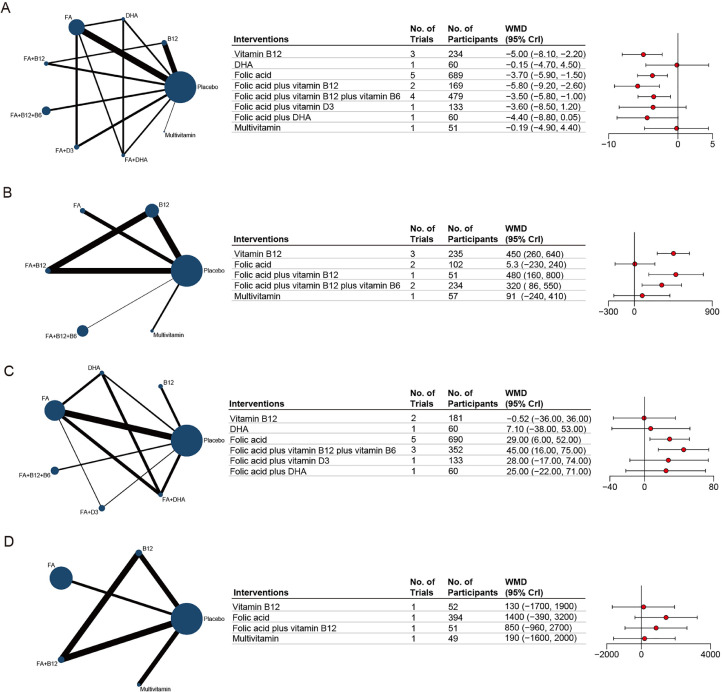
**Network meta-analysis plot of blood-based biomarkers associated with nutritional supplementation.** A, homocysteine. B, vitamin B12. C, serum folate. D, erythrocyte folate. Node size scales with the number of trials per intervention; edge thickness represents the volume of direct comparative evidence. Weighted mean differences (WMDs) with 95% confidence intervals are shown.

Due to the significant association between homocysteine concentrations and cognitive function, exploring changes in plasma total Hcy concentrations after nutrient supplementation holds clinical significance. A total of 14 studies, encompassing 9 treatment regimens and 3,489 participants were included to evaluate changes in homocysteine concentrations after nutrient supplementation. The results demonstrated that compared to placebo, supplementation with vitamin B12 (WMD, -5.00; 95% CI, -8.10 to -2.20), folic acid (WMD, -3.70; 95% CI, -5.90 to -1.50), folic acid+vitamin B12 (WMD, -5.80; 95% CI, -9.20 to -2.60), and folic acid+ vitamin B12+vitamin B6 (SMD, -3.50; 95% CI, -5.80 to -1.00) all significantly reduced tHcy concentrations (Fig. 3A). Additionally, SUCRA analysis indicated that all eight treatment modalities were more effective than placebo and the combined supplementation of folic acid and vitamin B12 showed the most pronounced effect, with the highest SUCRA score of 0.869 (eFigure 26A-C).

### 3.8 Vitamin B12

Markers of vitamin B12 are increasingly recognized as independent predictors of cognitive function. Therefore, we further analyzed changes in vitamin B12 levels following nutrient supplementation. A total of eight studies, encompassing six treatment strategies and 1,313 participants, were included in the network meta-analysis. The results demonstrated that, compared to placebo, supplementation with vitamin B12 (WMD: 450; 95% CI: 260 to 640), folic acid+vitamin B12 (WMD: 480; 95% CI: 160 to 800), and folic acid+vitamin B12+vitamin B6 (WMD: 320; 95% CI: 86 to 550) significantly increased vitamin B12 levels (Fig. 3B). SUCRA analysis further indicated that the most effective intervention was likely folic acid+vitamin B12 (SUCRA = 0.873) (eFigure 27A-B).

### 3.9 Folate

Serum folate and erythrocyte folate are two widely recognized biomarkers for assessing folate status, reflecting recent dietary folate intake and long-term folate reserves, respectively. In this study, folate concentrations were analyzed separately based on these two distinct biological compartments. For serum folate, a total of 10 studies, involving 7 treatment strategies and 2,720 participants, were included. Network meta-analysis revealed that, compared with placebo, supplementation with folic acid (WMD: 29.00; 95% CI: 6.00 to 52.00) and folic acid+vitamin B12+vitamin B6 (WMD: 45.00; 95% CI: 16.00 to 75.00) significantly increased serum folate concentrations (Fig. 3C). The SUCRA analysis further indicated that the most effective intervention was likely folic acid+vitamin B12+vitamin B6 (SUCRA = 0.883). Additionally, all six supplementation strategies demonstrated varying degrees of improvement in serum folate concentrations compared to placebo (eFigure 28A-B).

In contrast, for erythrocyte folate, 3 studies encompassing 5 treatment strategies and 1,062 participants were analyzed. The results suggested that nutrient supplementation had an uncertain effect on folate concentrations within red blood cells (Fig. 3D). However, SUCRA analysis indicated that folic acid supplementation (SUCRA = 0.876) appeared to be the most favorable approach among the evaluated strategies (eFigure 29A-B).

### 3.10 Sensitivity analysis

To enhance the robustness of our findings, a sensitivity analysis was performed by excluding studies with intervention durations shorter than six months. The SUCRA analysis indicated that melatonin continued to be the most effective intervention for global cognition. In terms of specific cognitive domains, the combination of folic acid and DHA was identified as the leading intervention for memory, whereas the superior efficacy of vitamin D3 for visuospatial function was consistent with the results of our primary analysis. Comprehensive results for all cognitive domains are available in eFigure 30.

## 4 Discussion

Oral nutrient supplementation has emerged as a promising approach for preserving cognitive health [61,45,78], yet the efficacy of individual regimens varies significantly across cognitive domains. Our findings demonstrate that a synergistic combination of DHA, EPA, vitamin E, tryptophan, and melatonin offers the most pronounced benefits for global cognition and memory function. In contrast, domain-specific effects were observed for single nutrients; DHA supplementation alone was most effective for enhancing processing speed, while vitamin D3 showed a distinct superiority in improving visuospatial function. Notably, while the impact on attention and executive functions remained limited, these results suggest that integrated, multi-nutrient protocols may be more effective than isolated interventions. By categorizing outcomes according to specific cognitive dimensions, this study provides a clearer hierarchy of evidence to guide personalized nutritional strategies in non-demented populations.

Within the RCTs considered in this analysis, vitamins B, C, D, and E have been extensively researched. Prior meta-analyses indicated that folic acid (B9) might mitigate progression to dementia in healthy elderly individuals, while no such benefits were observed for vitamins B12 or B6 [79]. Similarly, folic acid has been linked to cognitive improvements in Alzheimer's disease [80]. Regarding vitamin D, previous research reported a significant influence on overall cognition but found no effects on specific domains [81].Our study extends these findings by demonstrating that vitamin D3 alone significantly enhances visuospatial function, a specific domain previously underreported. Furthermore, while individual B vitamins showed limited efficacy in our analysis, we observed that combining folic acid with DHA significantly improved memory. This suggests that the cognitive benefits of vitamins may be optimized through synergistic nutrient interactions rather than isolated supplementation.

Omega-3 PUFAs exhibit anti-inflammatory and neuroprotective properties, with several studies showing they inhibit β-amyloid formation[[82], [83], [84], [85]]. While previous research by Tseng et al. indicated that high-dose EPA mitigated cognitive decline in AD patients [86], and another analysis found benefits for executive function in non-demented individuals [87], our findings provide a more nuanced perspective. Specifically, our analysis revealed that DHA alone significantly improved processing speed with a high certainty of evidence. Specifically, our analysis revealed that DHA alone significantly improved processing speed with a high certainty of evidence. This superior efficacy of DHA alone, compared to combined DHA and EPA protocols, is likely attributable to dosage differences. Specifically, studies using DHA alone provided doses up to 2 grams daily, whereas combined protocols were limited to 800 mg. By disaggregating these interventions, our results clarify that previously inconsistent findings in literature may stem from inadequate DHA dosages in combined supplementation regimens. Although no statistically significant differences were observed in the outcomes of omega-3 PUFA supplementation, the SUCRA analysis ranked the combination of DHA and EPA as the second most probable intervention for improving executive function and processing speed. This ranking indicates a potential trend that merits further investigation. However, it should not be interpreted as a definitive clinical benefit, particularly in light of the inherent limitations of SUCRA and the previously discussed dosage variations.

Melatonin is widely acknowledged for its efficacy in improving sleep quality [88] and offers additional benefits, such as free radical scavenging and oxidative stress reduction [89]. Quality sleep plays a significant role in enhancing cognitive function, which may contribute to slowing age-related cognitive decline. The findings of this study indicate that melatonin serves as an effective intervention for enhancing global cognition when compared to other nutritional supplements. Nonetheless, the study's scope was limited by its focus on a limited range of cognitive domains, thereby hindering the ability to draw more comprehensive conclusions.

Beyond B-vitamins, omega-3 PUFAs, and melatonin, recent evidence from the ADNI cohort highlights the neuroprotective potential of tryptophan, which appears to preserve cognitive function by supporting hippocampal structural integrity [90]. This effect is likely mediated by tryptophan serving as a precursor to serotonin, thereby modulating synaptic plasticity and neuroinflammatory pathways [91]. Conversely, the clinical efficacy of vitamin E remains controversial; for instance, alpha-tocopherol form of vitamin E has shown limited capacity to halt the progression from MCI to AD [92]. While our meta-analysis suggests that a synergistic combination of DHA, EPA, vitamin E, tryptophan, and melatonin may enhance cognition, this finding is currently limited by its reliance on a single study. The lack of comprehensive research into nutrient-nutrient interactions underscores the need for further clinical and mechanistic exploration to validate these potential synergistic effects.

While cognitive assessments highlighted improvements in specific cognitive areas, biochemical markers such as homocysteine, vitamin B12, and folate concentrations were analyzed to provide a more thorough understanding of the underlying metabolic changes. Emerging evidence indicates that vitamin B12-related biomarkers, including holotranscobalamin, homocysteine, and methylmalonic acid, are significantly associated with memory and executive function in patients with AD [93]. Additionally, recent study has demonstrated that, among individuals with AD or MCI, elevated homocysteine levels are associated with phosphorylated tau 217, particularly in those who are non-carriers of the APOE ε4 allele [94]. In the studies incorporated into our analysis, supplementation with folic acid, either independently or in conjunction with vitamins B6 or B12, was shown to significantly reduce Hcy levels and elevate serum concentrations of vitamin B12 and folate. Nonetheless, it is crucial to acknowledge that enhancements in these biochemical markers do not consistently correspond to measurable improvements in cognitive function among older adults or patients with MCI, as evidenced by the findings of Ford et al. [47]. This apparent discrepancy may arise from the multifactorial and complex nature of AD and MCI pathogenesis. Although serum vitamin B12 and Hcy levels are correlated with cognitive function, this relationship does not necessarily imply causation. While folic acid and vitamin B12 supplementation can effectively modulate these biochemical parameters, such as reducing Hcy and increasing serum vitamin B12 and folate levels, they may not directly reverse or significantly alleviate the neuronal damage or structural brain alterations that have already occurred.

In addition to individual nutrients, comprehensive dietary patterns such as the Mediterranean, DASH, and MIND diets have demonstrated significant neuroprotective potential [95,96]. These patterns prioritize the intake of omega-3 fatty acids, polyphenols, and monounsaturated fats while restricting pro-inflammatory components like saturated fats and refined sugars. Mechanistically, these diets are hypothesized to mitigate the production of proinflammatory cytokines and reactive oxygen species (ROS), which may subsequently reduce amyloid plaque formation and tau hyperphosphorylation. These patterns prioritize the intake of omega-3 fatty acids, polyphenols, and monounsaturated fats while restricting pro-inflammatory components like saturated fats and refined sugars. Mechanistically, these diets are hypothesized to mitigate the production of proinflammatory cytokines and ROS, which may subsequently reduce amyloid plaque formation and tau hyperphosphorylation[[97], [98], [99]]. Notably, the MIND diet has shown a distinct capacity to mitigate cognitive decline particularly in APOE-ε4 carriers and has been associated with reduced hippocampal atrophy [100,101]. Our findings align with this holistic perspective, as the superior efficacy observed in synergistic nutrient combinations (e.g., DHA, EPA, and Vitamin E) suggests that the cognitive benefits of nutrition are best realized through integrated dietary frameworks rather than isolated supplements. These results have significant implications for public health and clinical practice. First, they suggest that nutritional interventions for cognitive aging should transition from a "single-nutrient" approach to more comprehensive, evidence-based dietary patterns tailored to an individual’s genetic and metabolic profile. Second, the observed domain-specific benefits provide a foundation for personalized nutritional recommendations, such as prioritizing high-dose DHA for processing speed or Vitamin D3 for visuospatial function. Finally, policymakers should emphasize the promotion of brain-healthy diets, such as the MIND or Mediterranean patterns, as a cost-effective strategy for alleviating the global burden of cognitive decline in aging populations.

Regarding the strengths of this review, the primary merit lies in its comprehensive network meta-analysis that bridges the gap between biochemical marker changes and multi-domain cognitive performance. By evaluating interventions across six distinct cognitive domains, we provide a more granular evidence hierarchy than traditional pairwise meta-analyses, thereby offering a clearer perspective on domain-specific nutritional efficacy. However, this study has several limitations. Firstly, the study population was exclusively composed of individuals without dementia, thereby excluding patients with AD. This exclusion restricts the applicability of our findings to the broader AD population. Secondly, only 36% of the included studies featured large sample sizes, which may introduce small sample bias and compromise the robustness of the results. Thirdly, the inherent variability in intervention regimens was driven by evolving standards across different regions and time periods, which precluded a separate analysis of dosage and duration. Consequently, our findings are intended to highlight the directional neuroprotective potential of these nutrients rather than providing specific optimal dosage recommendations. Fourthly, as all interventions in the included studies were administered orally, factors such as the acceptability and safety of nutrient supplements were not explicitly addressed. Fifthly, non-dietary interventions, such as physical exercise, were not incorporated into this analysis, potentially neglecting synergistic effects between dietary and lifestyle interventions. Sixthly, our reliance on the final reported outcomes of published studies precluded a precise analysis of changes in cognitive performance over time. Seventhly, we prioritized the most frequently utilized assessment tool within each cognitive domain to ensure methodological consistency; however, this reliance on single tests rather than harmonized multi-test composites may not fully capture the complexity of each domain and could potentially increase the risk of false-positive results. Additionally, while SUCRA curves provide a probabilistic ranking of intervention efficacy, they have inherent methodological limitations, and the results should be interpreted with caution.

Despite these limitations, our findings provide a foundational framework for developing targeted nutritional interventions. Further high-quality RCTs are necessary to refine these protocols for broader clinical application. Future research should prioritize longitudinal trials using harmonized cognitive composites to evaluate the long-term impact of these nutrients on dementia incidence. Furthermore, exploring the synergy between integrated dietary patterns and lifestyle interventions remains essential for developing holistic preventive strategies.

## 5 Conclusion

In conclusion, our study, supported by comprehensive subgroup and sensitivity analyses, demonstrates that the cognitive benefits of nutritional supplementation are characterized by significant domain-specific variations. While multi-nutrient combinations may offer broader efficacy across multiple functions, our findings emphasize that individuals with specific cognitive deficits should prioritize targeted supplementation of particular nutrients. The consistency of these effects across different analytical models suggests that nutritional strategies for cognitive preservation should be tailored to the unique cognitive profile of the target population. This evidence-based approach provides a more precise and effective framework for personalizing nutritional interventions to support healthy brain aging.

## Funding

This work was supported by grants from the National Natural Science Foundation of China (82371205), Tianjin Health Research Project (TJWJ2023XK019), and Tianjin Public Health Science and Technology Major Project (24ZXGZSY00180).

## Ethics approval and consent to participate

Not applicable

## Availability of data and materials

All data generated or analyzed during this study are included in this published article and its supplementary information files.

## Declaration of the use of generative AI and AI-assisted technologies

The authors declare that no generative AI or AI-assisted technologies were used in the writing of this manuscript or in the preparation of figures, images, or artwork.

## CRediT authorship contribution statement

**Xing Liu:** Writing – original draft, Project administration, Investigation, Data curation, Conceptualization. **Chenyi Yang:** Writing – review & editing, Visualization, Investigation. **Xinyi Wang:** Visualization, Investigation. **Huihui Liao:** Investigation. **Huan Liu:** Investigation. **Ji Ma:** Investigation. **Yi Sun:** Investigation. **Haiyun Wang:** Writing – review & editing, Supervision, Conceptualization.

## Declaration of competing interest

The authors declare that they have no known competing financial interests or personal relationships that could have appeared to influence the work reported in this paper.

## Appendix Supplementary materials

Image, application 1

Image, application 2
