# Supplementary material for: Nutritional supplements and cognition in healthy aging and mild cognitive impairment patients: a systematic review and network meta-analysis
Source: J Prev Alzheimers Dis. 2026 Feb 28;13(5):100518. doi: 10.1016/j.tjpad.2026.100518 (PMC12966656; doi:10.1016/j.tjpad.2026.100518)
Supplement: Supplementary file 2 [file mmc2.docx]

**Supplementary Materials**

[Data collection and extraction 1](#_Toc185868861)

[Study quality assessment 2](#_Toc185868862)

[eTable 1 Search strategy 2](#_Toc185868863)

[eTable 1A Seach strategy of PubMed database 2](#_Toc185868864)

[eTable 1B Seach strategy of Web of Science database 7](#_Toc185868865)

[eTable 1C Seach strategy of Embase database 11](#_Toc185868866)

[eTable 1D Seach strategy of Cochrane Central Register of Controlled Trials 15](#_Toc185868867)

[eTable 2 Inclusion criteria 16](#_Toc185868868)

[eTable 3 List of excluded studies 17](#_Toc185868869)

[eTable 4 CINeMA assessment of the impact of different dietary supplement interventions on cognitive function in non-demented participants 25](#_Toc185868870)

[eTable 3A Global cognition 25](#_Toc185868871)

[eTable 3B Attention 29](#_Toc185868872)

[eTable 3C Executive function 30](#_Toc185868873)

[eTable 3D Memory 30](#_Toc185868874)

[eTable 3E Processing speed 32](#_Toc185868875)

[eTable 3F Visuospatial function 35](#_Toc185868876)

[eFigure 1 Risk of bias assessment for included studies using the ROB2 36](#_Toc185868877)

[Part Ⅰ Cognitive function assessment: Global cognition 37](#_Toc185868878)

[eFigure 2A Global cognition (All): Node splitting analysis 37](#_Toc185868879)

[eFigure 2B Global cognition (All): Bland Altman analysis 37](#_Toc185868880)

[eFigure 2C Global cognition (All): SUCRA plot 38](#_Toc185868881)

[eFigure 2D Global cognition (All): Ranking forest plot and ranking probability 39](#_Toc185868882)

[eFigure 3A Global cognition (Subgroup analysis-Age): A, Network plot; B, funnel plot; C, forest plot. 40](#_Toc185868883)

[eFigure 3B Global cognition (Subgroup analysis-Age): Node splitting analysis 41](#_Toc185868884)

[eFigure 3C Global cognition (Subgroup analysis-Age): SUCRA plot 42](#_Toc185868885)

[eFigure 3D Global cognition (Subgroup analysis-Age): Ranking forest plot and ranking probability 43](#_Toc185868886)

[eFigure 4A Global cognition (Subgroup analysis-MCI): A, Network plot; B, funnel plot; C, forest plot. 44](#_Toc185868887)

[eFigure 4B Global cognition (Subgroup analysis-MCI): SUCRA plot 45](#_Toc185868888)

[eFigure 4C Global cognition (Subgroup analysis-MCI): Ranking forest plot and ranking probability 46](#_Toc185868889)

[eFigure 5A Global cognition (Subgroup analysis-Sample size over 100): Network plot 47](#_Toc185868890)

[eFigure 5B Global cognition (Subgroup analysis-Sample size over 100): SUCRA plot 48](#_Toc185868891)

[eFigure 5C Global cognition (Subgroup analysis-Sample size over 100): Ranking forest plot and ranking probability 49](#_Toc185868892)

[Part Ⅱ Cognitive function assessment: Attention 50](#_Toc185868893)

[eFigure 6A Attention (All): Node splitting analysis 50](#_Toc185868894)

[eFigure 6B Attention (All): Bland Altman analysis 50](#_Toc185868895)

[eFigure 6C Attention (All): SUCRA plot 51](#_Toc185868896)

[eFigure 6D Attention (All): Ranking forest plot and ranking probability 52](#_Toc185868897)

[eFigure 7A Attention (Subgroup analysis-Age): A, Network plot; B, funnel plot; C, forest plot. 53](#_Toc185868898)

[eFigure 7B Attention (Subgroup analysis-Age): Node splitting analysis 54](#_Toc185868899)

[eFigure 7C Attention (Subgroup analysis-Age): SUCRA plot 54](#_Toc185868900)

[eFigure 7D Attention (Subgroup analysis-Age): Ranking forest plot and ranking probability 55](#_Toc185868901)

[eFigure 8A Attention (Subgroup analysis-MCI): A, Network plot; B, funnel plot; C, forest plot. 56](#_Toc185868902)

[eFigure 8B Attention (Subgroup analysis-MCI): Node splitting analysis 57](#_Toc185868903)

[eFigure 8C Attention (Subgroup analysis-MCI): SUCRA plot 57](#_Toc185868904)

[eFigure 8D Attention (Subgroup analysis-MCI): Ranking forest plot and ranking probability 58](#_Toc185868905)

[eFigure 9A Attention (Subgroup analysis-Sample size over 100): A, Network plot; B, funnel plot; C, forest plot. 59](#_Toc185868906)

[eFigure 9B Attention (Subgroup analysis-Sample size over 100): SUCRA plot 60](#_Toc185868907)

[eFigure 9C Attention (Subgroup analysis-Sample size over 100): Ranking forest plot and ranking probability 61](#_Toc185868908)

[Part Ⅲ Cognitive function assessment: Executive function 62](#_Toc185868909)

[eFigure 10A Executive function (All): Node splitting analysis 62](#_Toc185868910)

[eFigure 10B Executive function (All): Bland Altman analysis 62](#_Toc185868911)

[eFigure 10C Executive function (All): SUCRA plot 63](#_Toc185868912)

[eFigure 10D Executive function (All): Ranking forest plot and ranking probability 64](#_Toc185868913)

[eFigure 11A Executive function (Subgroup analysis-Age): A, Network plot; B, funnel plot; C, forest plot. 65](#_Toc185868914)

[eFigure 11B Executive function (Subgroup analysis-Age): Node splitting analysis 65](#_Toc185868915)

[eFigure 11C Executive function (Subgroup analysis-Age): SUCRA plot 66](#_Toc185868916)

[eFigure 11D Executive function (Subgroup analysis-Age): Ranking forest plot and ranking probability 67](#_Toc185868917)

[eFigure 12A Executive function (Subgroup analysis-MCI): A, Network plot; B, funnel plot; C, forest plot. 68](#_Toc185868918)

[eFigure 12B Executive function (Subgroup analysis-MCI): SUCRA plot 68](#_Toc185868919)

[eFigure 12C Executive function (Subgroup analysis-MCI): Ranking forest plot and ranking probability 69](#_Toc185868920)

[eFigure 13A Executive function (Subgroup analysis-Sample size over 100): A, Network plot; B, funnel plot; C, forest plot. 70](#_Toc185868921)

[eFigure 13B Executive function (Subgroup analysis-Sample size over 100): SUCRA plot 71](#_Toc185868922)

[eFigure 13C Executive function (Subgroup analysis-Sample size over 100): Ranking forest plot and ranking probability 72](#_Toc185868923)

[Part Ⅳ Cognitive function assessment: Memory 73](#_Toc185868924)

[eFigure 14A Memory (All): Node splitting analysis 73](#_Toc185868925)

[eFigure 14B Memory (All): Bland Altman analysis 73](#_Toc185868926)

[eFigure 14C Memory (All): SUCRA plot 74](#_Toc185868927)

[eFigure 14D Memory (All): Ranking forest plot and ranking probability 75](#_Toc185868928)

[eFigure 15A Memory (Subgroup analysis-Age): A, Network plot; B, funnel plot; C, forest plot. 76](#_Toc185868929)

[eFigure 15B Memory (Subgroup analysis-Age): Node splitting analysis 77](#_Toc185868930)

[eFigure 15C Memory (Subgroup analysis-Age): SUCRA plot 77](#_Toc185868931)

[eFigure 15D Memory (Subgroup analysis-Age): Ranking forest plot and ranking probability 78](#_Toc185868932)

[eFigure 16A Memory (Subgroup analysis-MCI): A, Network plot; B, funnel plot; C, forest plot. 79](#_Toc185868933)

[eFigure 16B Memory (Subgroup analysis-MCI): SUCRA plot 80](#_Toc185868934)

[eFigure 16C Memory (Subgroup analysis-MCI): Ranking forest plot and ranking probability 81](#_Toc185868935)

[eFigure 17A Memory (Subgroup analysis-Sample size over 100): A, Network plot; B, funnel plot; C, forest plot. 82](#_Toc185868936)

[eFigure 17B Memory (Subgroup analysis-Sample size over 100): SUCRA plot 83](#_Toc185868937)

[eFigure 17C Memory (Subgroup analysis-Sample size over 100): Ranking forest plot and ranking probability 84](#_Toc185868938)

[Part Ⅴ Cognitive function assessment: Processing speed 85](#_Toc185868939)

[eFigure 18A Processing speed (All): Node splitting analysis 85](#_Toc185868940)

[eFigure 18B Processing speed (All): Bland Altman analysis 85](#_Toc185868941)

[eFigure 18C Processing speed (All): SUCRA plot 86](#_Toc185868942)

[eFigure 18D Processing speed (All): Ranking forest plot and ranking probability 88](#_Toc185868943)

[eFigure 19A Processing speed (Subgroup analysis-Age): A, Network plot; B, funnel plot; C, forest plot. 89](#_Toc185868944)

[eFigure 19B Processing speed (Subgroup analysis-Age): SUCRA plot 90](#_Toc185868945)

[eFigure 19C Processing speed (Subgroup analysis-Age): Ranking forest plot and ranking probability 91](#_Toc185868946)

[eFigure 20A Processing speed (Subgroup analysis-MCI): A, Network plot; B, funnel plot; C, forest plot. 92](#_Toc185868947)

[eFigure 20B Processing speed (Subgroup analysis-MCI): SUCRA plot 93](#_Toc185868948)

[eFigure 20C Processing speed (Subgroup analysis-MCI): Ranking forest plot and ranking probability 94](#_Toc185868949)

[eFigure 21A Processing speed (Subgroup analysis-Sample size over 100): A, Network plot; B, funnel plot; C, forest plot. 95](#_Toc185868950)

[eFigure 21B Processing speed (Subgroup analysis-Sample size over 100): SUCRA plot 95](#_Toc185868951)

[eFigure 21C Processing speed (Subgroup analysis-Sample size over 100): Ranking forest plot and ranking probability 96](#_Toc185868952)

[Part Ⅵ Cognitive function assessment: Visuospatial function 97](#_Toc185868953)

[eFigure 22A Visuospatial function (All): Node splitting analysis 97](#_Toc185868954)

[eFigure 22B Visuospatial function (All): Bland Altman analysis 98](#_Toc185868955)

[eFigure 22C Visuospatial function (All): SUCRA plot 98](#_Toc185868956)

[eFigure 22D Visuospatial function (All): Ranking forest plot and ranking probability 99](#_Toc185868957)

[eFigure 23A Visuospatial function (Subgroup analysis-Age): A, Network plot; B, funnel plot; C, forest plot. 100](#_Toc185868958)

[eFigure 23B Visuospatial function (Subgroup analysis-Age): SUCRA plot 101](#_Toc185868959)

[eFigure 23C Visuospatial function (Subgroup analysis-Age): Ranking forest plot and ranking probability. 102](#_Toc185868960)

[eFigure 24A Visuospatial function (Subgroup analysis-MCI): A, Network plot; B, funnel plot; C, forest plot. 103](#_Toc185868961)

[eFigure 24B Visuospatial function (Subgroup analysis-MCI): SUCRA plot 103](#_Toc185868962)

[eFigure 24C Visuospatial function (Subgroup analysis-MCI): Ranking forest plot and ranking probability 104](#_Toc185868963)

[eFigure 25A Visuospatial function (Subgroup analysis-Sample size over 100): A, Network plot; B, funnel plot; C, forest plot. 105](#_Toc185868964)

[eFigure 25B Visuospatial function (Subgroup analysis-Sample size over 100): SUCRA plot 106](#_Toc185868965)

[eFigure 25C Visuospatial function (Subgroup analysis-Sample size over 100): Ranking forest plot and ranking probability 106](#_Toc185868966)

[Part Ⅶ Biochemical analysis: Homocysteine 107](#_Toc185868967)

[eFigure 26A Homocysteine: Node splitting analysis 107](#_Toc185868968)

[eFigure 26B Homocysteine: SUCRA plot 107](#_Toc185868969)

[eFigure 26C Homocysteine: Ranking forest plot and ranking probability 108](#_Toc185868970)

[Part Ⅷ Biochemical analysis: Vitamin B12 109](#_Toc185868971)

[eFigure 27A Vitamin B12: SUCRA plot 109](#_Toc185868972)

[eFigure 27B Vitamin B12: Ranking forest plot and ranking probability 110](#_Toc185868973)

[Part Ⅸ Biochemical analysis: Serum folate 111](#_Toc185868974)

[eFigure 28A Serum-folate: SUCRA plot 111](#_Toc185868975)

[eFigure 28B Serum-folate: Ranking forest plot and ranking probability 112](#_Toc185868976)

[Part Ⅹ Biochemical analysis: Erythrocyte folate 113](#_Toc185868977)

[eFigure 29A Erythrocyte folate: SUCRA plot 113](#_Toc185868978)

[eFigure 29B Erythrocyte folate: Ranking forest plot and ranking probability 114](#_Toc185868979)

## Data collection and extraction

A comprehensive search was conducted independently across multiple databases, and the retrieved datasets were exported to EndNote version X9 (Clarivate, Philadelphia, PA, USA) for deduplication. The subsequent process involved rigorous screening and data extraction. Initially, two authors (X.L. and H.L.) independently reviewed the titles and abstracts. Any discrepancies between reviewers were resolved through discussion. This was followed by a detailed review of the full-text articles and extraction of relevant data.

For the qualitative synthesis, information collected included the publication year, study location, demographic characteristics of participants (e.g., age, sex), baseline cognitive function evaluation criteria, assessment approaches used for each study group, details of the interventions (e.g., nutrient dosages and intervention duration), and other pertinent study parameters.

For the quantitative synthesis, we gathered detailed information on cognitive assessments, such as the tools employed, the cognitive domains evaluated (e.g., global cognition, attention, executive function, memory, processing speed, visuospatial function), and statistical metrics, including means, standard deviations (SDs), and sample sizes. When a cognitive domain was assessed with multiple tests in a single study, the most frequently used test across all studies was selected to ensure comparability. If cognitive test scores were reported at multiple time points, data from the longest intervention period were prioritized.

In instances where numerical results for cognitive tests were unavailable, approximate means and measures of variability were derived from the reported data. When medians and ranges were provided instead of means and SDs, appropriate statistical conversions were performed^1,2^. Additionally, pre- and post-intervention biochemical markers were extracted for analysis when reported (plasma total homocysteine, vitamin B12, serum folate, and erythrocyte folate).

Random-effect models were applied to account for inter-study variability, enhancing the robustness and generalizability of the findings. Careful consideration was given to selecting cognitive assessments and statistical methods to minimize heterogeneity and improve the reliability of the synthesized evidence.

## Study quality assessment

The Cochrane Risk of Bias 2 (RoB2) tool was utilized to systematically evaluate the risk of bias in the included studies. The assessment encompassed six key domains: (1) the randomization process, (2) deviations from intended interventions, (3) missing outcome data, (4) measurement of the outcome, (5) selection of reported results, and (6) the overall risk of bias. A study was deemed to have a low risk of bias only if all six domains were rated as low risk. Studies were categorized as having a moderate risk of bias if any domain was marked as "some concerns." If any domain was identified as having a high risk of bias, the study was classified as high risk. To provide a clear summary of the findings, a risk of bias summary and a corresponding chart were generated.

## eTable 1 Search strategy

### eTable 1A Seach strategy of PubMed database

| #ID | Topics | Category | Query | Records |
| --- | --- | --- | --- | --- |
| #1 | Disease | Alzheimer’s disease | ("Alzheimer Disease"[Mesh] OR "Alzheimer Syndrome"[Title/Abstract] OR "Alzheimer-Type Dementia (ATD)"[Title/Abstract] OR "Alzheimer Type Dementia (ATD)"[Title/Abstract] OR "Dementia, Alzheimer-Type (ATD)"[Title/Abstract] OR "Alzheimer's Diseases"[Title/Abstract] OR "Alzheimer Diseases"[Title/Abstract] OR "Alzheimers Diseases"[Title/Abstract] OR "Alzheimer Dementia"[Title/Abstract] OR "Alzheimer Dementias"[Title/Abstract] OR "Dementia, Alzheimer"[Title/Abstract] OR "Alzheimer's Disease"[Title/Abstract] OR "Dementia, Senile"[Title/Abstract] OR "Senile Dementia"[Title/Abstract] OR "Dementia, Alzheimer Type"[Title/Abstract] OR "Alzheimer Type Dementia"[Title/Abstract] OR "Senile Dementia, Alzheimer Type"[Title/Abstract] OR "Alzheimer Type Senile Dementia"[Title/Abstract] OR "Primary Senile Degenerative Dementia"[Title/Abstract] OR "Alzheimer clerosis"[Title/Abstract] OR Sclerosis, Alzheimer"[Title/Abstract] OR "Dementia, Primary Senile Degenerative"[Title/Abstract] OR "Dementia, Presenile"[Title/Abstract] OR "Presenile Dementia"[Title/Abstract] OR "Acute Confusional Senile Dementia"[Title/Abstract] OR "Senile Dementia, Acute Confusional"[Title/Abstract] OR "Alzheimer Disease, Early Onset"[Title/Abstract] OR "Early Onset Alzheimer Disease"[Title/Abstract] OR "Presenile Alzheimer Dementia"[Title/Abstract] OR "Alzheimer Disease, Late Onset"[Title/Abstract] OR "Late Onset Alzheimer Disease"[Title/Abstract] OR "Alzheimer's Disease, Focal Onset"[Title/Abstract] OR "Focal Onset Alzheimer's Disease"[Title/Abstract] OR "Familial Alzheimer Disease (FAD)"[Title/Abstract] OR "Alzheimer Disease, Familial (FAD)"[Title/Abstract] OR "Familial Alzheimer Diseases (FAD)"[Title/Abstract]) | 207,882 |
| #2 | Disease | Mild cognitive impairment | "Cognitive Dysfunction"[Mesh] OR "Cognitive Dysfunctions"[Title/Abstract] OR "Dysfunction, Cognitive"[Title/Abstract] OR "Dysfunctions, Cognitive"[Title/Abstract] OR "Cognitive Disorder"[Title/Abstract] OR "Cognitive Disorders"[Title/Abstract] OR "Disorder, Cognitive"[Title/Abstract] OR "Disorders, Cognitive"[Title/Abstract] OR "Cognitive Impairments"[Title/Abstract] OR "Cognitive Impairment"[Title/Abstract] OR "Impairment, Cognitive"[Title/Abstract] OR "Impairments, Cognitive"[Title/Abstract] OR "Mild Cognitive Impairment"[Title/Abstract] OR "Cognitive Impairment, Mild"[Title/Abstract] OR "Cognitive Impairments, Mild"[Title/Abstract] OR "Impairment, Mild Cognitive"[Title/Abstract] OR "Impairments, Mild Cognitive"[Title/Abstract] OR "Mild Cognitive Impairments"[Title/Abstract] OR "Cognitive Decline"[Title/Abstract] OR "Cognitive Declines"[Title/Abstract] OR "Decline, Cognitive"[Title/Abstract] OR "Declines, Cognitive"[Title/Abstract] OR "Mental Deterioration"[Title/Abstract] OR "Deterioration, Mental"[Title/Abstract] OR "Deteriorations, Mental"[Title/Abstract] OR "Mental Deteriorations"[Title/Abstract] | 148,632 |
| #3 | Disease | Cognition disorder | "Cognition Disorders"[Mesh] OR "Disorder, Cognition"[Title/Abstract] OR "Disorders, Cognition"[Title/Abstract] OR "Overinclusion"[Title/Abstract] | 125,584 |
| #4 | Disease | Healthy Volunteers | "Healthy Volunteers"[Mesh] OR "Healthy Volunteer"[Title/Abstract] OR "Volunteer, Healthy"[Title/Abstract] OR "Healthy Participants"[Title/Abstract] OR "Healthy Participant"[Title/Abstract] OR "Participant, Healthy"[Title/Abstract] OR "Participants, Healthy"[Title/Abstract] OR "Healthy Subjects"[Title/Abstract] OR "Healthy Subject"[Title/Abstract] OR "Subject, Healthy"[Title/Abstract] OR "Subjects, Healthy"[Title/Abstract] OR "Human Volunteers"[Title/Abstract] OR "Human Volunteer"[Title/Abstract] OR "Volunteer, Human"[Title/Abstract] OR "Volunteers, Human"[Title/Abstract] OR "Normal Volunteers"[Title/Abstract] OR "Normal Volunteer"[Title/Abstract] OR "Volunteer, Normal"[Title/Abstract] OR "Volunteers, Normal"[Title/Abstract] | 177,862 |
| #5 | Interventions | Vitamin B complex | ("Vitamin B Complex"[Mesh] OR "Neurobion"[Title/Abstract] OR "B Vitamins"[Title/Abstract] OR "B Vitamin"[Title/Abstract] OR "Vitamin, B"[Title/Abstract]) | 24,669 |
| #6 | Interventions | Vitamin D3 | ("Vitamin D3 24-Hydroxylase"[Mesh] OR "4-Hydroxylase, Vitamin D3"[Title/Abstract] OR "Vitamin D3 24 Hydroxylase"[Title/Abstract] OR "Vitamin D 24-Hydroxylase"[Title/Abstract] OR "Vitamin D 24 Hydroxylase"[Title/Abstract] OR "1 alpha,25-Dihydroxycholecalciferol-24-Hydroxylase"[Title/Abstract] OR "1 alpha,25 Dihydroxycholecalciferol 24 Hydroxylase"[Title/Abstract] OR "alpha,25-Dihydroxycholecalciferol-24-Hydroxylase, 1"[Title/Abstract] OR "1 alpha,25-Dihydroxyvitamin D(3)-24-Hydroxylase"[Title/Abstract] OR "1,25-Dihydroxyvitamin D3 24-Hydroxylase"[Title/Abstract] OR "1,25 Dihydroxyvitamin D3 24 Hydroxylase"[Title/Abstract] OR "D3 24-Hydroxylase, 1,25-Dihydroxyvitamin"[Title/Abstract] OR "25-Hydroxycholecalciferol-24-Hydroxylase"[Title/Abstract] OR "25 Hydroxycholecalciferol 24 Hydroxylase"[Title/Abstract] OR "CYP24A1"[Title/Abstract] OR "Calcitriol 24-Hydroxylase"[Title/Abstract] OR "Calcitriol 24 Hydroxylase"[Title/Abstract] OR "Cytochrome P-450 CYP24A1"[Title/Abstract] OR "CYP24A1, Cytochrome P-450"[Title/Abstract] OR "Cytochrome P 450 CYP24A1"[Title/Abstract] OR "P-450 CYP24A1, Cytochrome"[Title/Abstract]) | 1,913 |
| #7 | Interventions | Vitamin B6 | "Vitamin B 6"[Mesh] | 17,025 |
| #8 | Interventions | Vitamin E | "Vitamin E"[Mesh] | 35,322 |
| #9 | Interventions | Vitamin B12 | ("Vitamin B 12"[Mesh] OR "B 12, Vitamin"[Title/Abstract] OR "Cyanocobalamin"[Title/Abstract] OR "Vitamin B12"[Title/Abstract] OR "B12, Vitamin"[Title/Abstract] OR "Cobalamins"[Title/Abstract] OR "Cobalamin"[Title/Abstract] OR "Eritron"[Title/Abstract]) | 34,750 |
| #10 | Interventions | Riboflavin | ("Riboflavin"[Mesh] OR "Vitamin B 2"[Title/Abstract] OR "Vitamin G"[Title/Abstract] OR "Vitamin B2"[Title/Abstract]) | 17,134 |
| #11 | Interventions | Vitamin C | ("Ascorbic Acid"[Mesh] OR "Acid, Ascorbic"[Title/Abstract] OR "L-Ascorbic Acid"[Title/Abstract] OR "Acid, L-Ascorbic"[Title/Abstract] OR "L Ascorbic Acid"[Title/Abstract] OR "Vitamin C"[Title/Abstract] OR "Ferrous Ascorbate"[Title/Abstract] OR "Ascorbate, Ferrous"[Title/Abstract] OR "Magnesium Ascorbate"[Title/Abstract] OR "Ascorbate, Magnesium"[Title/Abstract] OR "Magnesium Ascorbicum"[Title/Abstract] OR "Magnesium di-L-Ascorbate"[Title/Abstract] OR "di-L-Ascorbate, Magnesium"[Title/Abstract] OR "Magnesium di L Ascorbate"[Title/Abstract] OR "Magnorbin"[Title/Abstract] OR "Hybrin"[Title/Abstract] OR "Sodium Ascorbate"[Title/Abstract] OR "Ascorbate, Sodium"[Title/Abstract] OR "Ascorbic Acid, Monosodium Salt"[Title/Abstract]) | 62,359 |
| #12 | Interventions | DHA | ("Docosahexaenoic Acids"[Mesh] OR "Acids, Docosahexaenoic"[Title/Abstract] OR "Docosahexenoic Acids"[Title/Abstract] OR "Acids, Docosahexenoic"[Title/Abstract] OR "Docosahexaenoic Acid"[Title/Abstract] OR "Acid, Docosahexaenoic"[Title/Abstract] OR "Docosahexaenoic Acid (All-Z Isomer)"[Title/Abstract] OR "Docosahexaenoic Acid, 4,7,10,13,16,19-(All-Z-Isomer)"[Title/Abstract] OR "Docosahexaenoic Acid, 4,7,10,13,16,19-Isomer, Sodium Salt"[Title/Abstract] OR "Docosahexaenoic Acid, 3,6,9,12,15,18-Isomer"[Title/Abstract] OR "Docosahexaenoic Acid, Sodium Salt"[Title/Abstract] OR "Docosahexaenoic Acid, 4,7,10,13,16,19-Isomer"[Title/Abstract] OR "Docosahexaenoic Acid, 4,7,10,13,16,19-(All-Z-Isomer), Potassium Salt"[Title/Abstract] OR "Docosahexaenoic Acid Dimer (All-Z Isomer)"[Title/Abstract] OR "Docosahexaenoic Acid, 4,7,10,13,16,19-(All-Z-Isomer), Cesium Salt"[Title/Abstract] OR "Docosahexaenoic Acid, 4,7,10,13,16,19-(All-Z-Isomer), Cerium Salt"[Title/Abstract] OR "Docosahexaenoate"[Title/Abstract] OR "Docosahexaenoic Acid, 4,7,10,13,16,19-(Z,Z,Z,Z,Z,E-Isomer)"[Title/Abstract]) | 19,123 |
| #13 | Interventions | EPA | ("Eicosapentaenoic Acid"[Mesh] OR "Icosapent"[Title/Abstract] OR "5,8,11,14,17-Eicosapentaenoic Acid"[Title/Abstract] OR "5,8,11,14,17-Icosapentaenoic Acid"[Title/Abstract] OR "omega-3-Eicosapentaenoic Acid"[Title/Abstract] OR "omega 3 Eicosapentaenoic Acid"[Title/Abstract] OR "Timnodonic Acid"[Title/Abstract] OR "Eicosapentanoic Acid"[Title/Abstract] OR "Acid, Eicosapentanoic"[Title/Abstract]) | 8,035 |
| #14 | Interventions | Melatonin | "Melatonin"[Mesh] | 24,564 |
| #15 | Interventions | Vitamins | "Vitamins" [Pharmacological Action] | 364,847 |
| #16 | Interventions | Folic acid | ("Folic Acid"[Mesh] OR "Pteroylglutamic Acid"[Title/Abstract] OR "Vitamin M"[Title/Abstract] OR "Vitamin B9"[Title/Abstract] OR "B9, Vitamin"[Title/Abstract] OR "Folacin"[Title/Abstract] OR "Folvite"[Title/Abstract] OR "Folic Acid, Potassium Salt"[Title/Abstract] OR "Folic Acid, Monopotassium Salt"[Title/Abstract] OR "Folic Acid, Sodium Salt"[Title/Abstract] OR "Folic Acid, Monosodium Salt"[Title/Abstract] OR "Folic Acid, (D)-Isomer"[Title/Abstract] OR "Folic Acid, (DL)-Isomer"[Title/Abstract] OR "Folic Acid, Calcium Salt (1:1)"[Title/Abstract] OR "Folate"[Title/Abstract]) | 59,071 |
| #17 | Study design | RCTs | (randomized controlled trial[pt] OR controlled clinical trial[pt] OR clinical trials as topic[mesh:noexp] OR trial[ti] OR random*[tiab] OR placebo*[tiab]) | 2,068,268 |
| #18 | Diseases | Combination | #1 OR #2 OR #3 OR #4 | 539,955 |
| #19 | Interventions | Combination | #5 OR #6 OR #7 OR #8 OR #9 OR #10 OR #11 OR #12OR #13 OR #14 OR #15 OR #16 | 5,523,287 |
| #20 | Final |  | #17 AND #18 AND #19 | 16,863 |

### eTable 1B Seach strategy of Web of Science database

| #ID | Topics | Category | Query | Records |
| --- | --- | --- | --- | --- |
| #1 | Disease | Alzheimer’s disease | TS=("Alzheimer Disease" OR "Alzheimer Syndrome" OR "Alzheimer-Type Dementia (ATD)" OR "Alzheimer Type Dementia (ATD)" OR "Dementia, Alzheimer-Type (ATD)" OR "Alzheimer's Diseases" OR "Alzheimer Diseases" OR "Alzheimers Diseases" OR "Alzheimer Dementia" OR "Alzheimer Dementias" OR "Dementia, Alzheimer" OR "Alzheimer's Disease" OR "Dementia, Senile" OR "Senile Dementia" OR "Dementia, Alzheimer Type" OR "Alzheimer Type Dementia" OR "Senile Dementia, Alzheimer Type" OR "Alzheimer Type Senile Dementia" OR "Primary Senile Degenerative Dementia" OR "Alzheimer Sclerosis" OR "Sclerosis, Alzheimer" OR "Dementia, Primary Senile Degenerative" OR "Dementia, Presenile" OR "Presenile Dementia" OR "Acute Confusional Senile Dementia" OR "Senile Dementia, Acute Confusional" OR "Alzheimer Disease, Early Onset" OR "Early Onset Alzheimer Disease" OR "Presenile Alzheimer Dementia" OR "Alzheimer Disease, Late Onset" OR "Late Onset Alzheimer Disease" OR "Alzheimer's Disease, Focal Onset" OR "Focal Onset Alzheimer's Disease" OR "Familial Alzheimer Disease (FAD)" OR "Alzheimer Disease, Familial (FAD)" OR "Familial Alzheimer Diseases (FAD)") | 189,294 |
| #2 | Disease | Mild cognitive impairment | TS=("Cognitive Dysfunction" OR "Cognitive Dysfunctions" OR "Dysfunction, Cognitive" OR "Dysfunctions, Cognitive" OR "Cognitive Disorder" OR "Cognitive Disorders" OR "Disorder, Cognitive" OR "Disorders, Cognitive" OR "Cognitive Impairments" OR "Cognitive Impairment" OR "Impairment, Cognitive" OR "Impairments, Cognitive" OR "Mild Cognitive Impairment" OR "Cognitive Impairment, Mild" OR "Cognitive Impairments, Mild" OR "Impairment, Mild Cognitive" OR "Impairments, Mild Cognitive" OR "Mild Cognitive Impairments" OR "Cognitive Decline" OR "Cognitive Declines" OR "Decline, Cognitive" OR "Declines, Cognitive" OR "Mental Deterioration" OR "Deterioration, Mental" OR "Deteriorations, Mental" OR "Mental Deteriorations") | 179,444 |
| #3 | Disease | Cognition disorder | TS=("Cognition Disorders" OR "Disorder, Cognition" OR "Disorders, Cognition" OR "Overinclusion") | 648 |
| #4 | Disease | Healthy Volunteers | TS=("Healthy Volunteers" OR "Healthy Volunteer" OR "Volunteer, Healthy" OR "Healthy Participants" OR "Healthy Participant" OR "Participant, Healthy" OR "Participants, Healthy" OR "Healthy Subjects" OR "Healthy Subject" OR "Subject, Healthy" OR "Subjects, Healthy" OR "Human Volunteers" OR "Human Volunteer" OR "Volunteer, Human" OR "Volunteers, Human" OR "Normal Volunteers" OR "Normal Volunteer" OR "Volunteer, Normal" OR "Volunteers, Normal") | 194,528 |
| #5 | Interventions | Vitamin B complex | TS=("Vitamin B Complex" OR "Neurobion" OR "B Vitamins" OR "B Vitamin" OR "Vitamin, B") | 19,127 |
| #6 | Interventions | Vitamin D3 | TS=("Vitamin D3 24-Hydroxylase" OR "4-Hydroxylase, Vitamin D3" OR "Vitamin D3 24 Hydroxylase" OR "Vitamin D 24-Hydroxylase" OR "Vitamin D 24 Hydroxylase" OR "1 alpha,25-Dihydroxycholecalciferol-24-Hydroxylase" OR "1 alpha,25 Dihydroxycholecalciferol 24 Hydroxylase" OR "alpha,25-Dihydroxycholecalciferol-24-Hydroxylase, 1" OR "1 alpha,25-Dihydroxyvitamin D(3)-24-Hydroxylase" OR "1,25-Dihydroxyvitamin D3 24-Hydroxylase" OR "1,25 Dihydroxyvitamin D3 24 Hydroxylase" OR "D3 24-Hydroxylase, 1,25-Dihydroxyvitamin" OR "25-Hydroxycholecalciferol-24-Hydroxylase" OR "25 Hydroxycholecalciferol 24 Hydroxylase" OR "CYP24A1" OR "Calcitriol 24-Hydroxylase" OR "Calcitriol 24 Hydroxylase" OR "Cytochrome P-450 CYP24A1" OR "CYP24A1, Cytochrome P-450" OR "Cytochrome P 450 CYP24A1" OR "P-450 CYP24A1, Cytochrome") | 1,469 |
| #7 | Interventions | Vitamin B6 | TS=("Vitamin B 6" OR "Vitamin B6" OR "Pyridoxine" OR "Pyridoxal" OR "Pyridoxamine") | 14,205 |
| #8 | Interventions | Vitamin E | TS=("Vitamin E" OR "Tocopherol" OR "Alpha-tocopherol" OR "Gamma-tocopherol" OR "Tocotrienol") | 63,989 |
| #9 | Interventions | Vitamin B12 | TS=("Vitamin B 12" OR "B 12, Vitamin" OR "Cyanocobalamin" OR "Vitamin B12" OR "B12, Vitamin" OR "Cobalamins" OR "Cobalamin" OR "Eritron") | 21,693 |
| #10 | Interventions | Riboflavin | TS=("Riboflavin" OR "Vitamin B 2" OR "Vitamin G" OR "Vitamin B2") | 13,033 |
| #11 | Interventions | Vitamin C | TS=("Ascorbic Acid" OR "Acid, Ascorbic" OR "L-Ascorbic Acid" OR "Acid, L-Ascorbic" OR "L Ascorbic Acid" OR "Vitamin C" OR "Ferrous Ascorbate" OR "Ascorbate, Ferrous" OR "Magnesium Ascorbate" OR "Ascorbate, Magnesium" OR "Magnesium Ascorbicum" OR "Magnesium di-L-Ascorbate" OR "di-L-Ascorbate, Magnesium" OR "Magnesium di L Ascorbate" OR "Magnorbin" OR "Hybrin" OR "Sodium Ascorbate" OR "Ascorbate, Sodium" OR "Ascorbic Acid, Monosodium Salt") | 94,281 |
| #12 | Interventions | DHA | TS=("Docosahexaenoic Acids" OR "Acids, Docosahexaenoic" OR "Docosahexenoic Acids" OR "Acids, Docosahexenoic" OR "Docosahexaenoic Acid" OR "Acid, Docosahexaenoic" OR "Docosahexaenoic Acid (All-Z Isomer)" OR "Docosahexaenoic Acid, 4,7,10,13,16,19-(All-Z-Isomer)" OR "Docosahexaenoic Acid, 4,7,10,13,16,19-Isomer, Sodium Salt" OR "Docosahexaenoic Acid, 3,6,9,12,15,18-Isomer" OR "Docosahexaenoic Acid, Sodium Salt" OR "Docosahexaenoic Acid, 4,7,10,13,16,19-Isomer" OR "Docosahexaenoic Acid, 4,7,10,13,16,19-(All-Z-Isomer), Potassium Salt" OR "Docosahexaenoic Acid Dimer (All-Z Isomer)" OR "Docosahexaenoic Acid, 4,7,10,13,16,19-(All-Z-Isomer), Cesium Salt" OR "Docosahexaenoic Acid, 4,7,10,13,16,19-(All-Z-Isomer), Cerium Salt" OR "Docosahexaenoate" OR "Docosahexaenoic Acid, 4,7,10,13,16,19-(Z,Z,Z,Z,Z,E-Isomer)") | 25,328 |
| #13 | Interventions | EPA | TS=("Eicosapentaenoic Acid" OR "Icosapent" OR "5,8,11,14,17-Eicosapentaenoic Acid" OR "5,8,11,14,17-Icosapentaenoic Acid" OR "omega-3-Eicosapentaenoic Acid" OR "omega 3 Eicosapentaenoic Acid" OR "Timnodonic Acid" OR "Eicosapentanoic Acid" OR "Acid, Eicosapentanoic") | 17,009 |
| #14 | Interventions | Melatonin | TS=("Melatonin") | 38,095 |
| #15 | Interventions | Vitamins | TS=("Vitamins") AND TS=("Pharmacological Action") | 4 |
| #16 | Interventions | Folic acid | TS=("Folic Acid" OR "Pteroylglutamic Acid" OR "Vitamin M" OR "Vitamin B9" OR "B9, Vitamin" OR "Folacin" OR "Folvite" OR "Folic Acid, Potassium Salt" OR "Folic Acid, Monopotassium Salt" OR "Folic Acid, Sodium Salt" OR "Folic Acid, Monosodium Salt" OR "Folic Acid, (D)-Isomer" OR "Folic Acid, (DL)-Isomer" OR "Folic Acid, Calcium Salt (1:1)" OR "Folate") | 52,515 |
| #17 | Study design | RCTs | TI=(random* OR placebo* OR trial) OR AB=(random* OR placebo*) | 2,008,733 |
| #18 | Diseases | Combination | #1 OR #2 OR #3 OR #4 | 505,502 |
| #19 | Interventions | Combination | #5 OR #6 OR #7 OR #8 OR #9 OR #10 OR #11 OR #12OR #13 OR #14 OR #15 OR #16 | 301,779 |
| #20 | Final |  | #17 AND #18 AND #19 | 1,676 |

### eTable 1C Seach strategy of Embase database

| #ID | Topics | Category | Query | Records |
| --- | --- | --- | --- | --- |
| #1 | Disease | Alzheimer’s disease | 'alzheimer disease'/exp/mj OR 'alzheimer syndrome':ab,ti OR 'alzheimer-type dementia (ATD)':ab,ti OR 'alzheimer type dementia (ATD)':ab,ti OR 'dementia, alzheimer-type (ATD)':ab,ti OR 'alzheimers diseases':ab,ti OR 'alzheimer diseases':ab,ti OR 'alzheimers diseases':ab,ti OR 'alzheimer dementia':ab,ti OR 'alzheimer dementias':ab,ti OR 'dementia, alzheimer':ab,ti OR 'alzheimers disease':ab,ti OR 'dementia, senile':ab,ti OR 'senile dementia':ab,ti OR 'dementia, alzheimer type':ab,ti OR 'alzheimer type dementia':ab,ti OR 'senile dementia, alzheimer type':ab,ti OR 'alzheimer type senile dementia':ab,ti OR 'primary senile degenerative dementia':ab,ti OR 'alzheimer sclerosis':ab,ti OR 'sclerosis, alzheimer':ab,ti OR 'dementia, primary senile degenerative':ab,ti OR 'dementia, presenile':ab,ti OR 'presenile dementia':ab,ti OR 'acute confusional senile dementia':ab,ti OR 'senile dementia, acute confusional':ab,ti OR 'alzheimer disease, early onset':ab,ti OR 'early onset alzheimer disease':ab,ti OR 'presenile alzheimer dementia':ab,ti OR 'alzheimer disease, late onset':ab,ti OR 'late onset alzheimer disease':ab,ti OR 'alzheimers disease, focal onset':ab,ti OR 'focal onset alzheimers disease':ab,ti OR 'familial alzheimer disease (FAD)':ab,ti OR 'alzheimer disease, familial (FAD)':ab,ti OR 'familial alzheimer diseases (FAD)':ab,ti | 159,981 |
| #2 | Disease | Mild cognitive impairment | 'cognitive dysfunction'/exp/mj OR 'cognitive dysfunction':ab,ti OR 'cognitive dysfunctions':ab,ti OR 'dysfunction, cognitive':ab,ti OR 'dysfunctions, cognitive':ab,ti OR 'cognitive disorder':ab,ti OR 'cognitive disorders':ab,ti OR 'disorder, cognitive':ab,ti OR 'disorders, cognitive':ab,ti OR 'cognitive impairments':ab,ti OR 'cognitive impairment':ab,ti OR 'impairment, cognitive':ab,ti OR 'impairments, cognitive':ab,ti OR 'mild cognitive impairment':ab,ti OR 'cognitive impairment, mild':ab,ti OR 'cognitive impairments, mild':ab,ti OR 'impairment, mild cognitive':ab,ti OR 'impairments, mild cognitive':ab,ti OR 'mild cognitive impairments':ab,ti OR 'cognitive decline':ab,ti OR 'cognitive declines':ab,ti OR 'decline, cognitive':ab,ti OR 'declines, cognitive':ab,ti OR 'mental deterioration':ab,ti OR 'deterioration, mental':ab,ti OR 'deteriorations, mental':ab,ti OR 'mental deteriorations':ab,ti | 478,098 |
| #3 | Disease | Cognitive disorders | 'cognition disorders'/exp/mj OR 'disorder, cognition':ab,ti OR 'disorders, cognition':ab,ti OR 'overinclusion':ab,ti | 364,782 |
| #4 | Disease | Healthy Volunteers | 'healthy volunteers'/exp/mj OR 'healthy volunteer':ab,ti OR 'volunteer, healthy':ab,ti OR 'healthy participants':ab,ti OR 'healthy participant':ab,ti OR 'participant, healthy':ab,ti OR 'participants, healthy':ab,ti OR 'healthy subjects':ab,ti OR 'healthy subject':ab,ti OR 'subject, healthy':ab,ti OR 'subjects, healthy':ab,ti OR 'human volunteers':ab,ti OR 'human volunteer':ab,ti OR 'volunteer, human':ab,ti OR 'volunteers, human':ab,ti OR 'normal volunteers':ab,ti OR 'normal volunteer':ab,ti OR 'volunteer, normal':ab,ti OR 'volunteers, normal':ab,ti | 221,864 |
| #5 | Interventions | Vitamin B complex | 'vitamin b complex'/exp/mj OR 'neurobion':ab,ti OR 'b vitamins':ab,ti OR 'b vitamin':ab,ti OR 'vitamin, b':ab,ti | 27,997 |
| #6 | Interventions | Vitamin D3 | 'vitamin d3 24-hydroxylase'/exp/mj OR '4-hydroxylase, vitamin d3':ab,ti OR 'vitamin d3 24 hydroxylase':ab,ti OR 'vitamin d 24-hydroxylase':ab,ti OR 'vitamin d 24 hydroxylase':ab,ti OR '1 alpha,25-dihydroxycholecalciferol-24-hydroxylase':ab,ti OR '1 alpha,25 dihydroxycholecalciferol 24 hydroxylase':ab,ti OR 'alpha,25-dihydroxycholecalciferol-24-hydroxylase, 1':ab,ti OR '1 alpha,25-dihydroxyvitamin d(3)-24-hydroxylase':ab,ti OR '1,25-dihydroxyvitamin d3 24-hydroxylase':ab,ti OR '1,25 dihydroxyvitamin d3 24 hydroxylase':ab,ti OR 'd3 24-hydroxylase, 1,25-dihydroxyvitamin':ab,ti OR '25-hydroxycholecalciferol-24-hydroxylase':ab,ti OR '25 hydroxycholecalciferol 24 hydroxylase':ab,ti OR 'cyp24a1':ab,ti OR 'calcitriol 24-hydroxylase':ab,ti OR 'calcitriol 24 hydroxylase':ab,ti OR 'cytochrome p-450 cyp24a1':ab,ti OR 'cyp24a1, cytochrome p-450':ab,ti OR 'cytochrome p 450 cyp24a1':ab,ti OR 'p-450 cyp24a1, cytochrome':ab,ti | 2,019 |
| #7 | Interventions | Vitamin B6 | 'vitamin b 6'/exp/mj OR 'vitamin b6':ab,ti OR 'pyridoxine':ab,ti OR 'pyridoxal':ab,ti OR 'pyridoxamine':ab,ti | 27,942 |
| #8 | Interventions | Vitamin E | 'vitamin e'/exp/mj OR 'vitamin e':ab,ti OR 'tocopherol':ab,ti OR 'alpha-tocopherol':ab,ti OR 'gamma-tocopherol':ab,ti OR 'tocotrienol':ab,ti | 63,111 |
| #9 | Interventions | Vitamin B12 | 'vitamin b12'/exp/mj OR 'b 12, vitamin':ab,ti OR 'cyanocobalamin':ab,ti OR 'vitamin b12':ab,ti OR 'b12, vitamin':ab,ti OR 'cobalamins':ab,ti OR 'cobalamin':ab,ti OR 'eritron':ab,ti | 39,245 |
| #10 | Interventions | Riboflavin | 'riboflavin'/exp/mj OR 'vitamin b 2':ab,ti OR 'vitamin g':ab,ti OR 'vitamin b2':ab,ti | 10,326 |
| #11 | Interventions | Vitamin C | 'ascorbic acid'/exp/mj OR 'acid, ascorbic':ab,ti OR 'l-ascorbic acid':ab,ti OR 'acid, l-ascorbic':ab,ti OR 'l ascorbic acid':ab,ti OR 'vitamin c':ab,ti OR 'ferrous ascorbate':ab,ti OR 'ascorbate, ferrous':ab,ti OR 'magnesium ascorbate':ab,ti OR 'ascorbate, magnesium':ab,ti OR 'magnesium ascorbicum':ab,ti OR 'magnesium di-l-ascorbate':ab,ti OR 'di-l-ascorbate, magnesium':ab,ti OR 'magnesium di l ascorbate':ab,ti OR 'magnorbin':ab,ti OR 'hybrin':ab,ti OR 'sodium ascorbate':ab,ti OR 'ascorbate, sodium':ab,ti OR 'ascorbic acid, monosodium salt':ab,ti | 63,726 |
| #12 | Interventions | DHA | 'docosahexaenoic acids'/exp/mj OR 'acids, docosahexaenoic':ab,ti OR 'docosahexenoic acids':ab,ti OR 'acids, docosahexenoic':ab,ti OR 'docosahexaenoic acid':ab,ti OR 'acid, docosahexaenoic':ab,ti OR 'docosahexaenoic acid (all-z isomer)':ab,ti OR 'docosahexaenoic acid, 4,7,10,13,16,19-(all-z-isomer)':ab,ti OR 'docosahexaenoic acid, 4,7,10,13,16,19-isomer, sodium salt':ab,ti OR 'docosahexaenoic acid, 3,6,9,12,15,18-isomer':ab,ti OR 'docosahexaenoic acid, sodium salt':ab,ti OR 'docosahexaenoic acid, 4,7,10,13,16,19-isomer':ab,ti OR 'docosahexaenoic acid, 4,7,10,13,16,19-(all-z-isomer), potassium salt':ab,ti OR 'docosahexaenoic acid dimer (all-z isomer)':ab,ti OR 'docosahexaenoic acid, 4,7,10,13,16,19-(all-z-isomer), cesium salt':ab,ti OR 'docosahexaenoic acid, 4,7,10,13,16,19-(all-z-isomer), cerium salt':ab,ti OR 'docosahexaenoate':ab,ti OR 'docosahexaenoic acid, 4,7,10,13,16,19-(z,z,z,z,z,e-isomer)':ab,ti | 20,764 |
| #13 | Interventions | EPA | 'eicosapentaenoic acid'/exp/mj OR 'icosapent':ab,ti OR '5,8,11,14,17-eicosapentaenoic acid':ab,ti OR '5,8,11,14,17-icosapentaenoic acid':ab,ti OR 'omega-3-eicosapentaenoic acid':ab,ti OR 'omega 3 eicosapentaenoic acid':ab,ti OR 'timnodonic acid':ab,ti OR 'eicosapentanoic acid':ab,ti OR 'acid, eicosapentanoic':ab,ti | 6.817 |
| #14 | Interventions | Melatonin | 'melatonin'/exp/mj OR 'melatonin':ab,ti | 40,409 |
| #15 | Interventions | Folic acid | ('folic acid'/exp OR 'pteroylglutamic acid':ab,ti OR 'vitamin m':ab,ti OR 'vitamin b9':ab,ti OR 'b9, vitamin':ab,ti OR 'folacin':ab,ti OR 'folvite':ab,ti OR 'folic acid, potassium salt':ab,ti OR 'folic acid, monopotassium salt':ab,ti OR 'folic acid, sodium salt':ab,ti OR 'folic acid, monosodium salt':ab,ti OR 'folic acid, (d)-isomer':ab,ti OR 'folic acid, (dl)-isomer':ab,ti OR 'folic acid, calcium salt (1:1)':ab,ti OR 'folate':ab,ti) | 94,803 |
| #16 | Study design | RCTs | ('controlled clinical trial'/exp OR (random* OR placebo*):ti,ab OR trial:ti) AND [embase]/lim | 2,221,717 |
| #17 | Diseases | Combination | #1 OR #2 OR #3 OR #4 | 697,318 |
| #18 | Interventions | Combination | #5 OR #6 OR #7 OR #8 OR #9 OR #10 OR #11 OR #12 OR #13 OR #14 OR #15 | 331,630 |
| #19 | Final |  | #16 AND #17 AND #18 | 1,532 |

### eTable 1D Seach strategy of Cochrane Central Register of Controlled Trials

| #ID | Topics | Category | Query | Records |
| --- | --- | --- | --- | --- |
| #1 | Disease | Alzheimer’s disease | MeSH descriptor: [Alzheimer Disease] explode all trees | 15,350 |
| #2 | Disease | Mild cognitive impairment | MeSH descriptor: [Cognitive Dysfunction] explode all trees | 10,797 |
| #3 | Disease | Cognitive disorders | MeSH descriptor: [Cognitive disorders] explode all trees | 11,206 |
| #4 | Disease | Healthy Volunteers | MeSH descriptor: [Healthy Volunteers] explode all trees | 59,049 |
| #5 | Interventions | Vitamin B complex | MeSH descriptor: [Vitamin B complex] explode all trees | 1,964 |
| #6 | Interventions | Vitamin D3 | MeSH descriptor: [Vitamin D3 ] explode all trees | 5,288 |
| #7 | Interventions | Vitamin B6 | MeSH descriptor: [Vitamin B6] explode all trees | 1,388 |
| #8 | Interventions | Vitamin E | MeSH descriptor: [Vitamin E] explode all trees | 13,202 |
| #9 | Interventions | Vitamin B12 | MeSH descriptor: [Vitamin B12] explode all trees | 5,495 |
| #10 | Interventions | Riboflavin | MeSH descriptor: [Riboflavin] explode all trees | 1,211 |
| #11 | Interventions | Vitamin C | MeSH descriptor: [Vitamin C] explode all trees | 4,481 |
| #12 | Interventions | DHA | MeSH descriptor: [DHA] explode all trees | 3,227 |
| #13 | Interventions | EPA | MeSH descriptor: [EPA] explode all trees | 3,303 |
| #14 | Interventions | Melatonin | MeSH descriptor: [Melatonin] explode all trees | 4,343 |
| #15 | Interventions | Folic acid | MeSH descriptor: [Folic acid] explode all trees | 5,882 |
| #16 | Disease | Combination | #1 OR #2 OR #3 OR #4 | 90,874 |
| #17 | Interventions | Combination | #5 OR #6 OR #7 OR #8 OR #9 OR #10 OR #11 OR #12 OR #13 OR #14 OR #15 | 36,627 |
| #18 | Study design | RCTs | (randomised controlled trial):ti,ab,kw | 901,144 |
| #19 | Study design | RCTs | MeSH descriptor: [Randomized Controlled Trial] explode all trees | 1,144,573 |
| #20 | Study design | RCTs | MeSH descriptor: [Random Allocation] explode all trees | 43,437 |
| #21 | Study design | RCTs | (random allocation):ti,ab,kw | 123,835 |
| #22 | Study design | Combination | #18 OR #19 OR #20 OR #21 | 1,484,992 |
| #23 | Final |  | #16 AND #17 AND #22 | 2,270 (Trials 1746) |

## eTable 2 Inclusion criteria

| **Domains** | | **Inclusion criteria** |
| --- | --- | --- |
| P | Population | Adults aged >18 years. Participants must be explicitly stated as non-demented, or the proportion of participants diagnosed with Alzheimer’s disease must be less than half of the total participants. |
| I | Intervention | Observation of the use of dietary substances as an intervention. Studies involving pharmacological or exercise interventions are excluded. |
| C | Comparison | Control groups receiving either no dietary intervention, a placebo, or standard care (if applicable). |
| O | Outcome | Assessment of the effects of dietary interventions on specific health outcomes, such as cognitive function, biomarkers, or other relevant measures. |
| S | Study design | Only Randomized Controlled Trials (RCTs) are included. Studies with other designs are excluded. |

## eTable 3 List of excluded studies

| Reason | No. | References |
| --- | --- | --- |
| Wrong outcomes | 1 | Atmadja TFA, Kusharto CM, Sinaga T: Supplementation of catfish (clarias gariepinus) oil enriched with omega-3 softcapsule improve oxidative stress and cognitive function in elderly people. Annals of Nutrition and Metabolism 2019, 75(3):70. |
| Wrong outcomes | 2 | Beauchet O, Launay CP, Galery K, Vilcocq C, Dontot-Payen F, Rousseau B, Benoit V, Allali G: Effects of Vitamin D and Calcium Fortified Yogurts on Gait, Cognitive Performances, and Serum 25-Hydroxyvitamin D Concentrations in Older Community-Dwelling Females: Results from the GAit, MEmory, Dietary and Vitamin D (GAME-D2) Randomized Controlled Trial. Nutrients 2019, 11(12). |
| Wrong outcomes | 3 | Bo Y, Zhang X, Wang Y, You J, Cui H, Zhu Y, Pang W, Liu W, Jiang Y, Lu Q: The n-3 Polyunsaturated Fatty Acids Supplementation Improved the Cognitive Function in the Chinese Elderly with Mild Cognitive Impairment: A Double-Blind Randomized Controlled Trial. Nutrients 2017, 9(1). |
| Wrong outcomes | 4 | Byrn MA, Adams W, Penckofer S, Emanuele MA: Vitamin D Supplementation and Cognition in People with Type 2 Diabetes: A Randomized Control Trial. Journal of diabetes research 2019, 2019:5696391. |
| Wrong outcomes | 5 | Cazzola R, Rondanelli M, Faliva M, Cestaro B: Effects of DHA-phospholipids, melatonin and tryptophan supplementation on erythrocyte membrane physico-chemical properties in elderly patients suffering from mild cognitive impairment. Experimental Gerontology 2012, 47(12):974-978. |
| Wrong outcomes | 6 | Dysken MW, Sano M, Asthana S, Vertrees JE, Pallaki M, Llorente M, Love S, Schellenberg GD, McCarten JR, Malphurs J et al: Effect of vitamin E and memantine on functional decline in Alzheimer disease: The TEAM-AD VA cooperative randomized trial. JAMA 2014, 311(1):33-44. |
| Wrong outcomes | 7 | Ford AH, Flicker L, Alfonso H, Thomas J, Clarnette R, Martins R, Almeida OP: Vitamins B12, B6, and folic acid for cognition in older men. Neurology 2010, 75(17):1540-1547. |
| Wrong outcomes | 8 | Freund-Levi Y, Eriksdotter-Jönhagen M, Cederholm T, Basun H, Faxén-Irving G, Garlind A, Vedin I, Vessby B, Wahlund LO, Palmblad J: ω-3 fatty acid treatment in 174 patients with mild to moderate Alzheimer disease:: OmegAD study -: A randomized double-blind trial. ARCHIVES OF NEUROLOGY 2006, 63(10):1402-+. |
| Wrong outcomes | 9 | Jakubowski H, Zioła-Frankowska A, Frankowski M, Perła-Kaján J, Refsum H, De Jager CA, Smith AD: B Vitamins Prevent Iron-Associated Brain Atrophy and Domain-Specific Effects of Iron, Copper, Aluminum, and Silicon on Cognition in Mild Cognitive Impairment. Journal of Alzheimer's Disease 2021, 84(3):1039-1055. |
| Wrong outcomes | 10 | Jernerén F, Cederholm T, Refsum H, Smith AD, Turner C, Palmblad J, Eriksdotter M, Hjorth E, Faxen-Irving G, Wahlund LO et al: Homocysteine status modifies the treatment effect of omega-3 fatty acids on cognition in a randomized clinical trial in mild to moderate Alzheimer's disease: The OmegAD Study. Journal of Alzheimer's Disease 2019, 69(1):189-197. |
| Wrong outcomes | 11 | Jernerén F, Elshorbagy AK, Oulhaj A, Smith SM, Refsum H, Smith AD: Brain atrophy in cognitively impaired elderly: The importance of long-chain ω-3 fatty acids and B vitamin status in a randomized controlled trial. American Journal of Clinical Nutrition 2015, 102(1):215-221. |
| Wrong outcomes | 12 | Jia J, Hu J, Huo X, Miao R, Zhang Y, Ma F: Effects of vitamin D supplementation on cognitive function and blood Aβ-related biomarkers in older adults with Alzheimer's disease: a randomised, double-blind, placebo-controlled trial. Journal of neurology, neurosurgery, and psychiatry 2019, 90(12):1347-1352. |
| Wrong outcomes | 13 | Jorde R, Kubiak J, Svartberg J, Fuskevåg OM, Figenschau Y, Martinaityte I, Grimnes G: Vitamin D supplementation has no effect on cognitive performance after four months in mid-aged and older subjects. J Neurol Sci 2019, 396:165-171. |
| Wrong outcomes | 14 | Köbe T, Witte A, Schnelle A, Lesemann A, Fabian S, Tesky VA, Pantel J, Flöel A: Combined omega-3 fatty acids, aerobic exercise and cognitive stimulation prevents decline in gray matter volume of the frontal, Parietal and cingulate cortex in patients with mild cognitive impairment. NeuroImage 2016, 131:226-238. |
| Wrong outcomes | 15 | Külzow N, Witte AV, Kerti L, Grittner U, Schuchardt JP, Hahn A, Flöel A: Impact of Omega-3 Fatty Acid Supplementation on Memory Functions in Healthy Older Adults. Journal of Alzheimer's Disease 2016, 51(3):713-725. |
| Wrong outcomes | 16 | Kuszewski JC, Wong RHX, Howe PRC: Effects of Long-Chain Omega-3 Polyunsaturated Fatty Acids on Endothelial Vasodilator Function and Cognition-Are They Interrelated? Nutrients 2017, 9(5). |
| Wrong outcomes | 17 | Kwok T, Lee J, Law CB, Pan PC, Yung CY, Choi KC, Lam LC: A randomized placebo controlled trial of homocysteine lowering to reduce cognitive decline in older demented people. Clinical Nutrition 2011, 30(3):297-302. |
| Wrong outcomes | 18 | Lawlor PG, McNamara-Kilian MT, MacDonald AR, Momoli F, Tierney S, Lacaze-Masmonteil N, Dasgupta M, Agar M, Pereira JL, Currow DC et al: Melatonin to prevent delirium in patients with advanced cancer: a double blind, parallel, randomized, controlled, feasibility trial. BMC palliative care 2020, 19(1):163. |
| Wrong outcomes | 19 | Ma F, Wu T, Zhao J, Han F, Marseglia A, Liu H, Huang G: Effects of 6-Month Folic Acid Supplementation on Cognitive Function and Blood Biomarkers in Mild Cognitive Impairment: A Randomized Controlled Trial in China. J Gerontol A Biol Sci Med Sci 2016, 71(10):1376-1383. |
| Wrong outcomes | 20 | Maltais M, de Souto Barreto P, Bowman GL, Smith AD, Cantet C, Andrieu S, Rolland Y: Omega-3 Supplementation for the Prevention of Cognitive Decline in Older Adults: Does It Depend on Homocysteine Levels? Journal of Nutrition, Health and Aging 2022, 26(6):615-620. |
| Wrong outcomes | 21 | Menczel Schrire Z, Phillips CL, Duffy SL, Marshall NS, Mowszowski L, La Monica HM, Gordon CJ, Chapman JL, Saini B, Lewis SJG et al: Feasibility of 3-month melatonin supplementation for brain oxidative stress and sleep in mild cognitive impairment: protocol for a randomised, placebo-controlled study. BMJ Open 2021, 11(2):e041500. |
| Wrong outcomes | 22 | Milte CM, Sinn N, Street SJ, Buckley JD, Coates AM, Howe PR: Erythrocyte polyunsaturated fatty acid status, memory, cognition and mood in older adults with mild cognitive impairment and healthy controls. Prostaglandins, leukotrienes, and essential fatty acids 2011, 84(5-6):153-161. |
| Wrong outcomes | 23 | O'Callaghan N, Parletta N, Milte CM, Benassi-Evans B, Fenech M, Howe PR: Telomere shortening in elderly individuals with mild cognitive impairment may be attenuated with ω-3 fatty acid supplementation: a randomized controlled pilot study. Nutrition 2014, 30(4):489-491. |
| Wrong outcomes | 24 | Ogawa T, Sawane K, Ookoshi K, Kawashima R: Supplementation with Flaxseed Oil Rich in Alpha-Linolenic Acid Improves Verbal Fluency in Healthy Older Adults. Nutrients 2023, 15(6). |
| Wrong outcomes | 25 | Rondanelli M, Opizzi A, Faliva M, Mozzoni M, Antoniello N, Cazzola R, Savarè R, Cerutti R, Grossi E, Cestaro B: Effects of a diet integration with an oily emulsion of DHA-phospholipids containing melatonin and tryptophan in elderly patients suffering from mild cognitive impairment. Nutr Neurosci 2012, 15(2):46-54. |
| Wrong outcomes | 26 | Rutjes AW, Denton DA, Di Nisio M, Chong LY, Abraham RP, Al-Assaf AS, Anderson JL, Malik MA, Vernooij RW, Martínez G et al: Vitamin and mineral supplementation for maintaining cognitive function in cognitively healthy people in mid and late life. Cochrane Database Syst Rev 2018, 12(12):Cd011906. |
| Wrong outcomes | 27 | Schwarz C, Wirth M, Gerischer L, Grittner U, Witte AV, Köbe T, Flöel A: Effects of Omega-3 Fatty Acids on Resting Cerebral Perfusion in Patients with Mild Cognitive Impairment: A Randomized Controlled Trial. The journal of prevention of Alzheimer's disease 2018, 5(1):26-30. |
| Wrong outcomes | 28 | Shea T, Remington R, Bechtel C, Sammar AM, Larsen D, Lortie JJ, Dosanjh L, Smyers K, Hoffman H, Fishman P: A nutritional formulation for cognitive performance and mood in Alzheimer's disease and mild cognitive impairment: A phase II multisite randomized trial with an open-label extension. Alzheimer's and Dementia 2013, 9(4):P658-P659. |
| Wrong outcomes | 29 | Smith AD, Smith SM, de Jager CA, Whitbread P, Johnston C, Agacinski G, Oulhaj A, Bradley KM, Jacoby R, Refsum H: Homocysteine-lowering by b vitamins slows the rate of accelerated brain atrophy in mild cognitive impairment: A randomized controlled trial. PLoS ONE 2010, 5(9):1-10. |
| Wrong outcomes | 30 | Stein MS, Scherer SC, Ladd KS, Harrison LC: A randomized controlled trial of high-dose vitamin D2 followed by intranasal insulin in Alzheimer's disease. Journal of Alzheimer's Disease 2011, 26(3):477-484. |
| Wrong outcomes | 31 | Sun Y, Lu CJ, Chien KL, Chen ST, Chen RC: Efficacy of multivitamin supplementation containing vitamins B6 and B12 and folic acid as adjunctive treatment with a cholinesterase inhibitor in Alzheimer's disease: a 26-week, randomized, double-blind, placebo-controlled study in Taiwanese patients. Clin Ther 2007, 29(10):2204-2214. |
| Wrong outcomes | 32 | Tyrberg E, Hagberg L, Andersson LM, Nilsson S, Yilmaz A, Mellgren Å, Blennow K, Zetterberg H, Gisslén M: The effect of vitamin B supplementation on neuronal injury in people living with HIV: a randomized controlled trial. Brain communications 2022, 4(6):fcac259. |
| Wrong outcomes | 33 | Vercambre MN, Grodstein F, Kang JH: Dietary fat intake in relation to cognitive change in high-risk women with cardiovascular disease or vascular factors. Eur J Clin Nutr 2010, 64(10):1134-1140. |
| Wrong outcomes | 34 | Wade AG, Farmer M, Harari G, Fund N, Laudon M, Nir T, Frydman-Marom A, Zisapel N: Add-on prolonged-release melatonin for cognitive function and sleep in mild to moderate Alzheimer's disease: A 6-month, randomized, placebo-controlled, multicenter trial. Clinical Interventions in Aging 2014, 9:947-961. |
| Wrong outcomes | 35 | Walker JG, Batterham PJ, Mackinnon AJ, Jorm AF, Hickie I, Fenech M, Kljakovic M, Crisp D, Christensen H: Oral folic acid and vitamin B-12 supplementation to prevent cognitive decline in community-dwelling older adults with depressive symptoms--the Beyond Ageing Project: a randomized controlled trial. The American journal of clinical nutrition 2012, 95(1):194-203. |
| Wrong outcomes | 36 | Wu Y, Smith AD, Bastani NE, Refsum H, Kwok T: The dihydrofolate reductase 19-bp deletion modifies the beneficial effect of B-vitamin therapy in mild cognitive impairment: pooled study of two randomized placebo-controlled trials. Human molecular genetics 2022, 31(7):1151-1158. |
| Wrong outcomes | 37 | Wu Y, Smith AD, Refsum H, Kwok T: Effectiveness of B Vitamins and Their Interactions with Aspirin in Improving Cognitive Functioning in Older People with Mild Cognitive Impairment: Pooled Post-Hoc Analyses of Two Randomized Trials. The journal of nutrition, health & aging 2021, 25(10):1154-1160. |
| Wrong outcomes | 38 | Yurko-Mauro K: Cognitive and cardiovascular benefits of docosahexaenoic acid in aging and cognitive decline. Current Alzheimer Research 2010, 7(3):190-196. |
| Alzheimer’s disease | 39 | Aisen PS, Schafer KA, Grundman M, Pfeiffer E, Sano M, Davis KL, Farlow MR, Jin S, Thomas RG, Thal LJ: Effects of rofecoxib or naproxen vs placebo on Alzheimer disease progression: a randomized controlled trial. Jama 2003, 289(21):2819-2826. |
| Alzheimer’s disease | 40 | Aisen PS, Schneider LS, Sano M, Diaz-Arrastia R, van Dyck CH, Weiner MF, Bottiglieri T, Jin S, Stokes KT, Thomas RG et al: High-dose B vitamin supplementation and cognitive decline in Alzheimer disease: a randomized controlled trial. Jama 2008, 300(15):1774-1783. |
| Alzheimer’s disease | 41 | Annweiler C, Fantino B, Parot-Schinkel E, Thiery S, Gautier J, Beauchet O: Alzheimer's disease - input of vitamin D with mEmantine assay (AD-IDEA trial): Study protocol for a randomized controlled trial. Trials 2011, 12. |
| Alzheimer’s disease | 42 | Cao D, Zhang Y, Zhang S, Li J, Yang Q, Wang P: Risk of Alzheimer's disease and genetically predicted levels of 1400 plasma metabolites: a Mendelian randomization study. Sci Rep 2024, 14(1):26078. |
| Alzheimer’s disease | 43 | Chen H, Liu S, Ge B, Zhou D, Li M, Li W, Ma F, Liu Z, Ji Y, Huang G: Effects of Folic Acid and Vitamin B12 Supplementation on Cognitive Impairment and Inflammation in Patients with Alzheimer’s Disease: A Randomized, Single-Blinded, Placebo-Controlled Trial. Journal of Prevention of Alzheimer's Disease 2021, 8(3):249-256. |
| Alzheimer’s disease | 44 | Chen L, Sun X, Wang Z, Lu Y, Chen M, He Y, Xu H, Zheng L: The impact of plasma vitamin C levels on the risk of cardiovascular diseases and Alzheimer's disease: A Mendelian randomization study. Clinical nutrition (Edinburgh, Scotland) 2021, 40(10):5327-5334. |
| Alzheimer’s disease | 45 | Connelly PJ, Prentice NP, Cousland G, Bonham J: A randomised double-blind placebo-controlled trial of folic acid supplementation of cholinesterase inhibitors in Alzheimer's disease. Int J Geriatr Psychiatry 2008, 23(2):155-160. |
| Alzheimer’s disease | 46 | De Souto Barreto P, Rolland Y, Cesari M, Dupuy C, Andrieu S, Vellas B: Effects of multidomain lifestyle intervention, omega-3 supplementation or their combination on physical activity levels in older adults: Secondary analysis of the Multidomain Alzheimer Preventive Trial (MAPT) randomised controlled trial. Age and Ageing 2018, 47(2):281-288. |
| Alzheimer’s disease | 47 | Dysken MW: Effect of Vitamin E and Memantine on Functional Decline in Alzheimer Disease: The TEAM-AD VA Cooperative Randomized Trial (vol 311, pg 33, 2014). JAMA-JOURNAL OF THE AMERICAN MEDICAL ASSOCIATION 2014, 311(11):1161-1161. |
| Alzheimer’s disease | 48 | Dysken MW, Sano M, Asthana S, Vertrees JE, Pallaki M, Llorente M, Love S, Schellenberg GD, McCarten JR, Malphurs J et al: Effect of vitamin E and memantine on functional decline in Alzheimer disease: The TEAM-AD VA cooperative randomized trial. JAMA 2014, 311(1):33-44. |
| Alzheimer’s disease | 49 | Freund-Levi Y, Eriksdotter-Jönhagen M, Cederholm T, Basun H, Faxén-Irving G, Garlind A, Vedin I, Vessby B, Wahlund LO, Palmblad J: Omega-3 fatty acid treatment in 174 patients with mild to moderate Alzheimer disease: OmegAD study: a randomized double-blind trial. Arch Neurol 2006, 63(10):1402-1408. |
| Alzheimer’s disease | 50 | Freund-Levi Y, Eriksdotter-Jönhagen M, Cederholm T, Basun H, Faxén-Irving G, Garlind A, Vedin I, Vessby B, Wahlund LO, Palmblad J: ω-3 fatty acid treatment in 174 patients with mild to moderate Alzheimer disease:: OmegAD study -: A randomized double-blind trial. ARCHIVES OF NEUROLOGY 2006, 63(10):1402-+. |
| Alzheimer’s disease | 51 | Fu LL, Vollkommer T, Fuest S, Gosau M, Feng H, Yan M, Smeets R, Friedrich RE: The Role of 25-OH Vitamin D in Alzheimer's Disease through Mendelian Randomization and MRI. QJM : monthly journal of the Association of Physicians 2024. |
| Alzheimer’s disease | 52 | Galasko DR, Peskind E, Clark CM, Quinn JF, Ringman JM, Jicha GA, Cotman C, Cottrell B, Montine TJ, Thomas RG et al: Antioxidants for Alzheimer disease: A randomized clinical trial with cerebrospinal fluid biomarker measures. Archives of Neurology 2012, 69(7):836-841. |
| Alzheimer’s disease | 53 | Gehrman PR, Connor DJ, Martin JL, Shochat T, Corey-Bloom J, Ancoli-Israel S: Melatonin fails to improve sleep or agitation in double-blind randomized placebo-controlled trial of institutionalized patients with Alzheimer disease. The American journal of geriatric psychiatry : official journal of the American Association for Geriatric Psychiatry 2009, 17(2):166-169. |
| Alzheimer’s disease | 54 | Geldmacher DS, Fritsch T, McClendon MJ, Landreth G: A randomized pilot clinical trial of the safety of pioglitazone in treatment of patients with alzheimer disease. Archives of Neurology 2011, 68(1):45-50. |
| Alzheimer’s disease | 55 | Ilboudo Y, Yoshiji S, Lu T, Butler-Laporte G, Zhou S, Richards JB: Vitamin D, Cognition, and Alzheimer's Disease: Observational and Two-Sample Mendelian Randomization Studies. Journal of Alzheimer's disease : JAD 2024, 99(4):1243-1260. |
| Alzheimer’s disease | 56 | Jernerén F, Cederholm T, Refsum H, Smith AD, Turner C, Palmblad J, Eriksdotter M, Hjorth E, Faxen-Irving G, Wahlund LO et al: Homocysteine status modifies the treatment effect of omega-3 fatty acids on cognition in a randomized clinical trial in mild to moderate Alzheimer's disease: The OmegAD Study. Journal of Alzheimer's Disease 2019, 69(1):189-197. |
| Alzheimer’s disease | 57 | Jia J, Hu J, Huo X, Miao R, Zhang Y, Ma F: Effects of Vitamin D supplementation on cognitive function and blood Aβ-related biomarkers in older adults with Alzheimer's disease: A randomised, double-blind, placebo-controlled trial. Journal of Neurology, Neurosurgery and Psychiatry 2019, 90(12):1347-1352. |
| Alzheimer’s disease | 58 | Jyvakorpi SK, Puranen T, Pitkala KH, Suominen MH: Nutritional treatment of aged individuals with Alzheimer disease living at home with their spouses: study protocol for a randomized controlled trial. Trials 2012, 13. |
| Alzheimer’s disease | 59 | Larsson SC, Traylor M, Malik R, Dichgans M, Burgess S, Markus HS: Modifiable pathways in Alzheimer's disease: Mendelian randomisation analysis. BMJ (Clinical research ed) 2017, 359:j5375. |
| Alzheimer’s disease; meta-analysis | 60 | Lee CY, Chan L, Hu CJ, Hong CT, Chen JH: Role of vitamin B12 and folic acid in treatment of Alzheimer's disease: a meta-analysis of randomized control trials. Aging 2024, 16(9):7856-7869. |
| Alzheimer’s disease | 61 | Liu G, Zhao Y, Jin S, Hu Y, Wang T, Tian R, Han Z, Xu D, Jiang Q: Circulating vitamin E levels and Alzheimer's disease: a Mendelian randomization study. Neurobiol Aging 2018, 72:189.e181-189.e189. |
| Alzheimer’s disease | 62 | Liu H, Zhang Y, Hu Y, Zhang H, Wang T, Han Z, Gao S, Wang L, Liu G: Mendelian randomization to evaluate the effect of plasma vitamin C levels on the risk of Alzheimer’s disease. Genes and Nutrition 2021, 16(1). |
| Alzheimer’s disease | 63 | Meng L, Wang Z, Ming YC, Shen L, Ji HF: Are micronutrient levels and supplements causally associated with the risk of Alzheimer's disease? A two-sample Mendelian randomization analysis. Food Funct 2022, 13(12):6665-6673. |
| Alzheimer’s disease | 64 | Phillips MA, Childs CE, Calder PC, Rogers PJ: No effect of omega-3 fatty acid supplementation on cognition and mood in individuals with cognitive impairment and probable Alzheimer’s disease: A randomised controlled trial. International Journal of Molecular Sciences 2015, 16(10):24600-24613. |
| Alzheimer’s disease | 65 | Quinn JF, Raman R, Thomas RG, Yurko-Mauro K, Nelson EB, Van Dyck C, Galvin JE, Emond J, Jack CR, Weiner M et al: Docosahexaenoic acid supplementation and cognitive decline in Alzheimer disease: A randomized trial. JAMA 2010, 304(17):1903-1911. |
| Alzheimer’s disease | 66 | Remington R, Bechtel C, Larsen D, Samar A, Doshanjh L, Fishman P, Luo Y, Smyers K, Page R, Morrell C et al: A Phase II Randomized Clinical Trial of a Nutritional Formulation for Cognition and Mood in Alzheimer's Disease. Journal of Alzheimer's Disease 2015, 45(2):395-405. |
| Alzheimer’s disease | 67 | Richard E, Kuiper R, Dijkgraaf MG, Van Gool WA: Vascular care in patients with Alzheimer's disease with cerebrovascular lesions-a randomized clinical trial. J Am Geriatr Soc 2009, 57(5):797-805. |
| Alzheimer’s disease | 68 | Sato Y, Honda Y, Umeno K, Hayashida N, Iwamoto J, Takeda T, Matsumoto H: The prevention of hip fracture with menatetrenone and risedronate plus calcium supplementation in elderly patients with Alzheimer disease: a randomized controlled trial. The Kurume medical journal 2011, 57(4):117-124. |
| Alzheimer’s disease | 69 | Sato Y, Iwamoto J, Kanoko T, Satoh K: Amelioration of osteoporosis and hypovitaminosis D by sunlight exposure in hospitalized, elderly women with Alzheimer's disease: a randomized controlled trial. Journal of bone and mineral research : the official journal of the American Society for Bone and Mineral Research 2005, 20(8):1327-1333. |
| Alzheimer’s disease | 70 | Sato Y, Kanoko T, Satoh K, Iwamoto J: RETRACTED: The prevention of hip fracture with risedronate and ergocalciferol plus calcium supplementation in elderly women with Alzheimer disease: A randomized controlled trial (Retracted Article. See vol. 176, pg. 1256, 2016). Archives of internal medicine 2005, 165(15):1737-1742. |
| Alzheimer’s disease | 71 | Shah RC, Kamphuis PJ, Leurgans S, Swinkels SH, Sadowsky CH, Bongers A, Rappaport SA, Quinn JF, Wieggers RL, Scheltens P et al: The S-Connect study: Results from a randomized, controlled trial of Souvenaid in mild-to-moderate Alzheimer's disease. Alzheimer's Research and Therapy 2013, 5(6). |
| Alzheimer’s disease | 72 | Shea T, Remington R, Bechtel C, Sammar AM, Larsen D, Lortie JJ, Dosanjh L, Smyers K, Hoffman H, Fishman P: A nutritional formulation for cognitive performance and mood in Alzheimer's disease and mild cognitive impairment: A phase II multisite randomized trial with an open-label extension. Alzheimer's and Dementia 2013, 9(4):P658-P659. |
| Alzheimer’s disease | 73 | Shinto L, Quinn J, Montine T, Dodge HH, Woodward W, Baldauf-Wagner S, Waichunas D, Bumgarner L, Bourdette D, Silbert L et al: A randomized placebo-controlled pilot trial of omega-3 fatty acids and alpha lipoic acid in Alzheimer's disease. Journal of Alzheimer's Disease 2014, 38(1):111-120. |
| Alzheimer’s disease | 74 | Stein MS, Scherer SC, Ladd KS, Harrison LC: A randomized controlled trial of high-dose vitamin D2 followed by intranasal insulin in Alzheimer's disease. Journal of Alzheimer's Disease 2011, 26(3):477-484. |
| Alzheimer’s disease; meta-analysis | 75 | Sumsuzzman DM, Choi J, Jin Y, Hong Y: Neurocognitive effects of melatonin treatment in healthy adults and individuals with Alzheimer's disease and insomnia: A systematic review and meta-analysis of randomized controlled trials. Neuroscience and Biobehavioral Reviews 2021, 127:459-473. |
| Alzheimer’s disease | 76 | Sun X, He C, Yang S, Li W, Qu H: Mendelian randomization to evaluate the effect of folic acid supplement on the risk of Alzheimer disease. Medicine 2024, 103(6):e37021. |
| Alzheimer’s disease | 77 | Sun Y, Lu CJ, Chien KL, Chen ST, Chen RC: Efficacy of multivitamin supplementation containing vitamins B6 and B12 and folic acid as adjunctive treatment with a cholinesterase inhibitor in Alzheimer's disease: a 26-week, randomized, double-blind, placebo-controlled study in Taiwanese patients. Clin Ther 2007, 29(10):2204-2214. |
| Alzheimer’s disease | 78 | Sun Y, Lu CJ, Chien KL, Chen ST, Chen RC: Efficacy of multivitamin supplementation containing vitamins B<sub>6</sub> and B<sub>12</sub> and folic acid as adjunctive treatment with a cholinesterase inhibitor in Alzheimer's disease:: A 26-week, randomized, double-blind, placebo-controlled study in Taiwanese patients. CLINICAL THERAPEUTICS 2007, 29(10):2204-2214. |
| Alzheimer’s disease | 79 | Tofiq A, Zetterberg H, Blennow K, Basun H, Cederholm T, Eriksdotter M, Faxén-Irving G, Hjorth E, Jernerén F, Schultzberg M et al: Effects of peroral omega-3 fatty acid supplementation on cerebrospinal fluid biomarkers in patients with alzheimer's disease: A randomized controlled trial - The OmegAD study. Journal of Alzheimer's Disease 2021, 83(3):1291-1301. |
| Alzheimer’s disease | 80 | Tomata Y, Larsson SC, Hägg S: Polyunsaturated fatty acids and risk of Alzheimer's disease: a Mendelian randomization study. Eur J Nutr 2020, 59(4):1763-1766. |
| Alzheimer’s disease; meta-analysis | 81 | Tseng PT, Zeng BS, Suen MW, Wu YC, Correll CU, Zeng BY, Kuo JS, Chen YW, Chen TY, Tu YK et al: Efficacy and acceptability of anti-inflammatory eicosapentaenoic acid for cognitive function in Alzheimer's dementia: A network meta-analysis of randomized, placebo-controlled trials with omega-3 fatty acids and FDA-approved pharmacotherapy. Brain, Behavior, and Immunity 2023, 111:352-364. |
| Alzheimer’s disease; meta-analysis | 82 | Tseng PT, Zeng BY, Chen YW, Yang CP, Su KP, Chen TY, Wu YC, Tu YK, Lin PY, Carvalho AF et al: The Dose and Duration-dependent Association between Melatonin Treatment and Overall Cognition in Alzheimer's Dementia: A Network Meta- Analysis of Randomized Placebo-Controlled Trials. Curr Neuropharmacol 2022, 20(10):1816-1833. |
| Alzheimer’s disease | 83 | Wade AG, Farmer M, Harari G, Fund N, Laudon M, Nir T, Frydman-Marom A, Zisapel N: Add-on prolonged-release melatonin for cognitive function and sleep in mild to moderate Alzheimer's disease: A 6-month, randomized, placebo-controlled, multicenter trial. Clinical Interventions in Aging 2014, 9:947-961. |
| Alzheimer’s disease | 84 | Wang J, Huang Y, Bei C, Yang H, Lin Z, Xu L: Causal associations of antioxidants with Alzheimer's disease and cognitive function: A Mendelian randomisation study. Journal of Epidemiology and Community Health 2024, 78(7):424-430. |
| Alzheimer’s disease | 85 | Wang L, Qiao Y, Zhang H, Zhang Y, Hua J, Jin S, Liu G: Circulating Vitamin D Levels and Alzheimer's Disease: A Mendelian Randomization Study in the IGAP and UK Biobank. J Alzheimers Dis 2020, 73(2):609-618. |
| Alzheimer’s disease; meta-analysis | 86 | Wang YY, Zheng W, Ng CH, Ungvari GS, Wei W, Xiang YT: Meta-analysis of randomized, double-blind, placebo-controlled trials of melatonin in Alzheimer's disease. International Journal of Geriatric Psychiatry 2017, 32(1):50-57. |
| Alzheimer’s disease | 87 | Williams DM, Hägg S, Pedersen NL: Circulating antioxidants and Alzheimer disease prevention: a Mendelian randomization study. The American journal of clinical nutrition 2019, 109(1):90-98. |
| Alzheimer’s disease | 88 | Zhang H, Wang T, Han Z, Wang L, Zhang Y, Wang L, Liu G: Impact of Vitamin D Binding Protein Levels on Alzheimer's Disease: A Mendelian Randomization Study. J Alzheimers Dis 2020, 74(3):991-998. |

## eTable 4 CINeMA assessment of the impact of different dietary supplement interventions on cognitive function in non-demented participants

### eTable 3A Global cognition


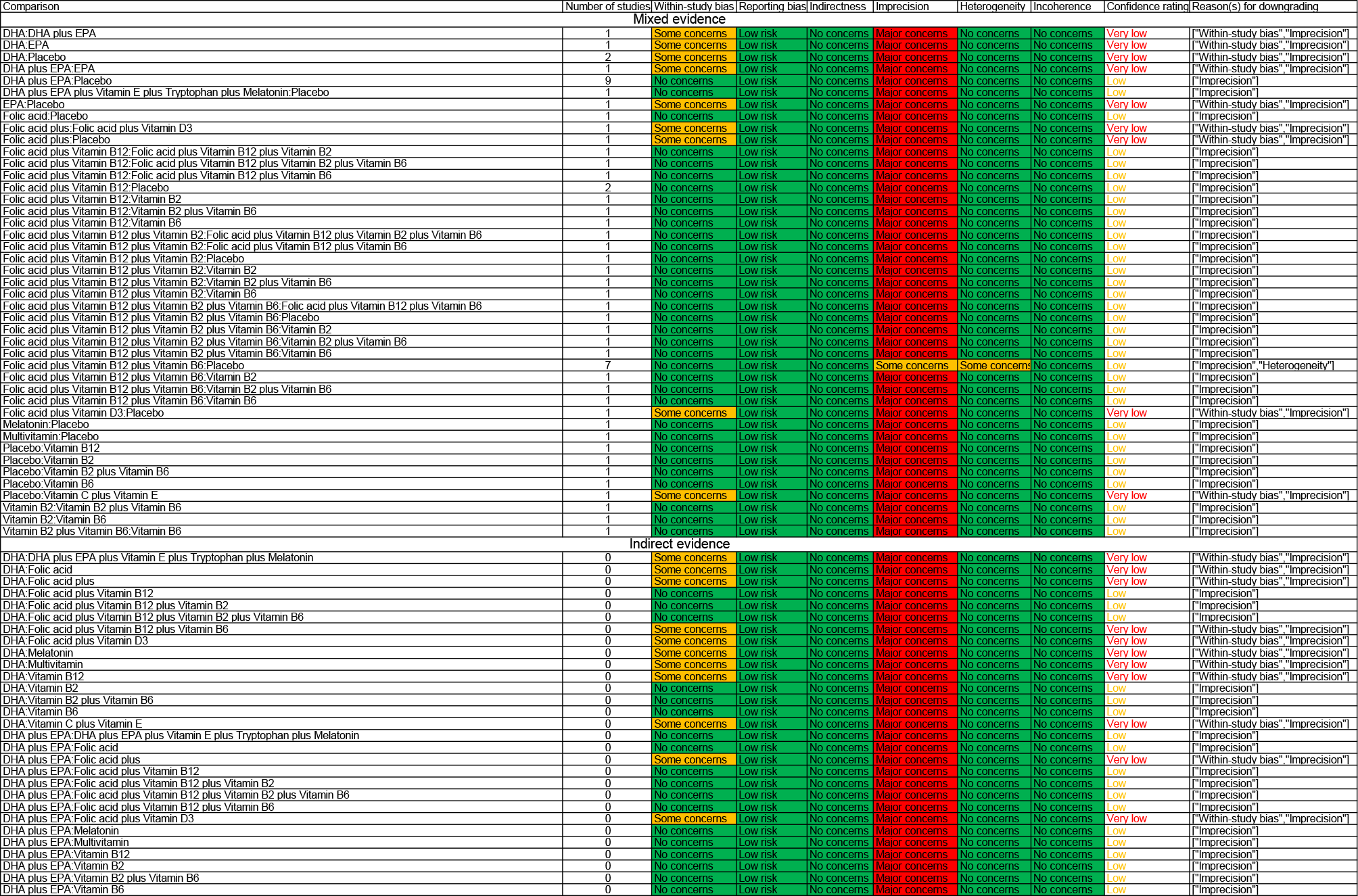

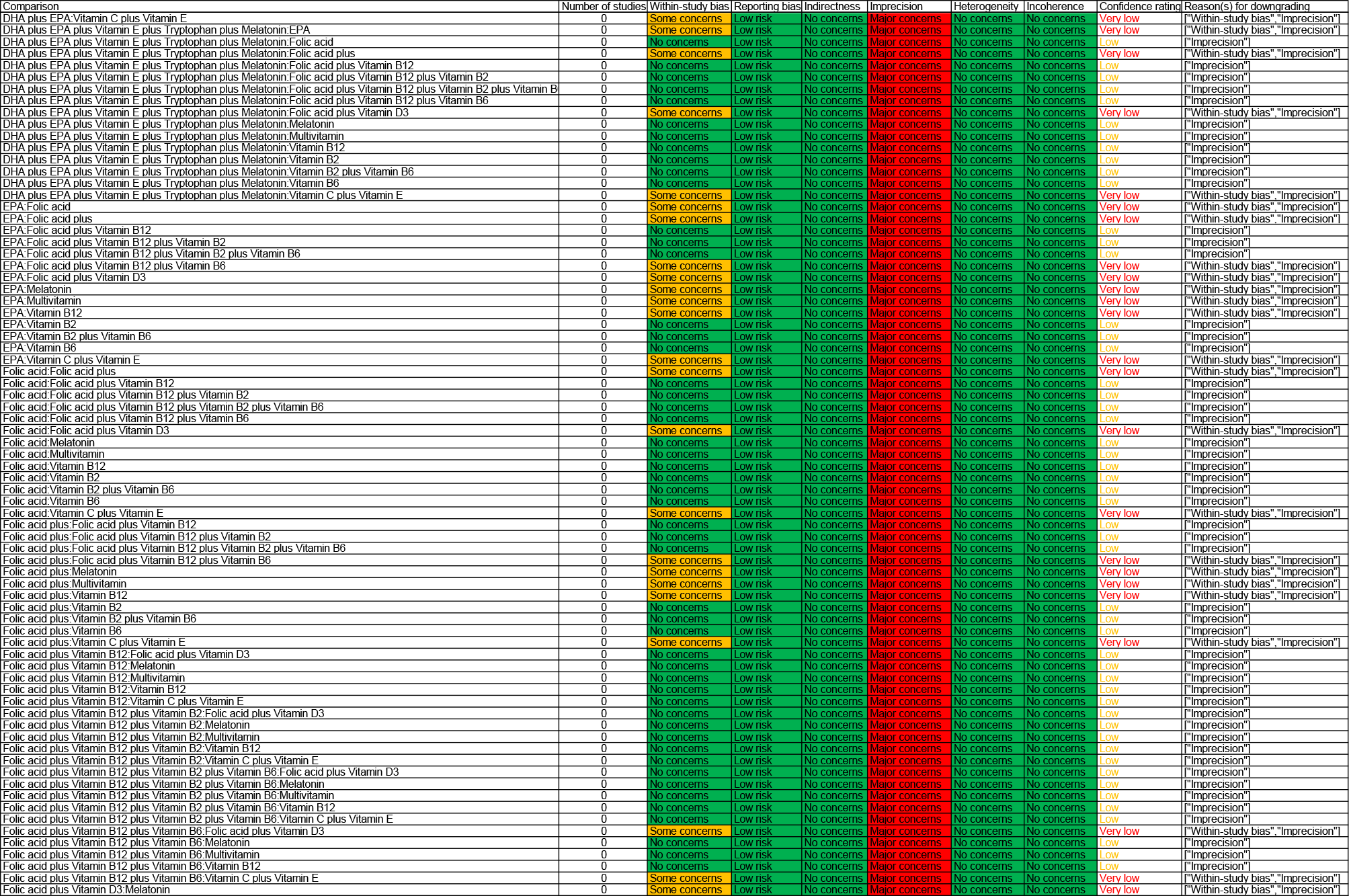

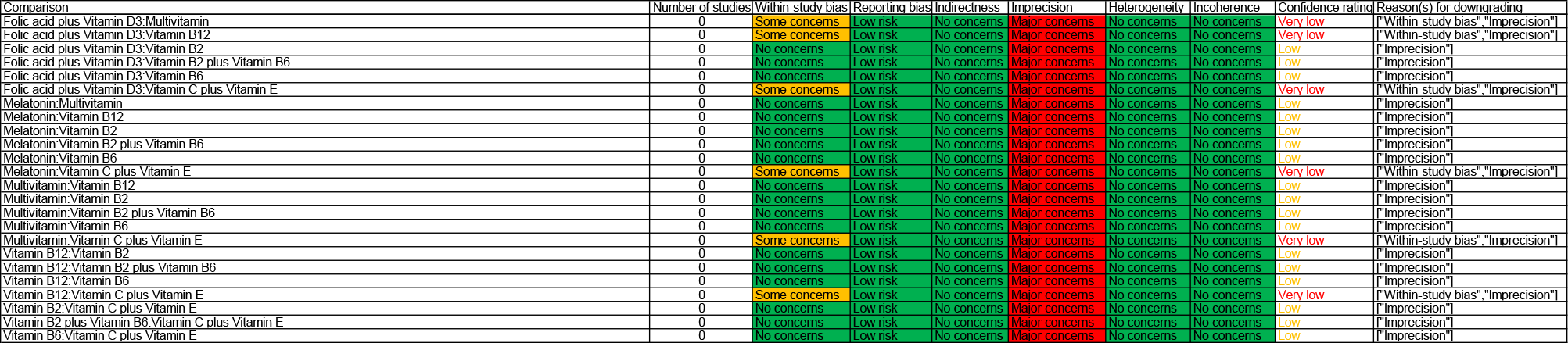


###
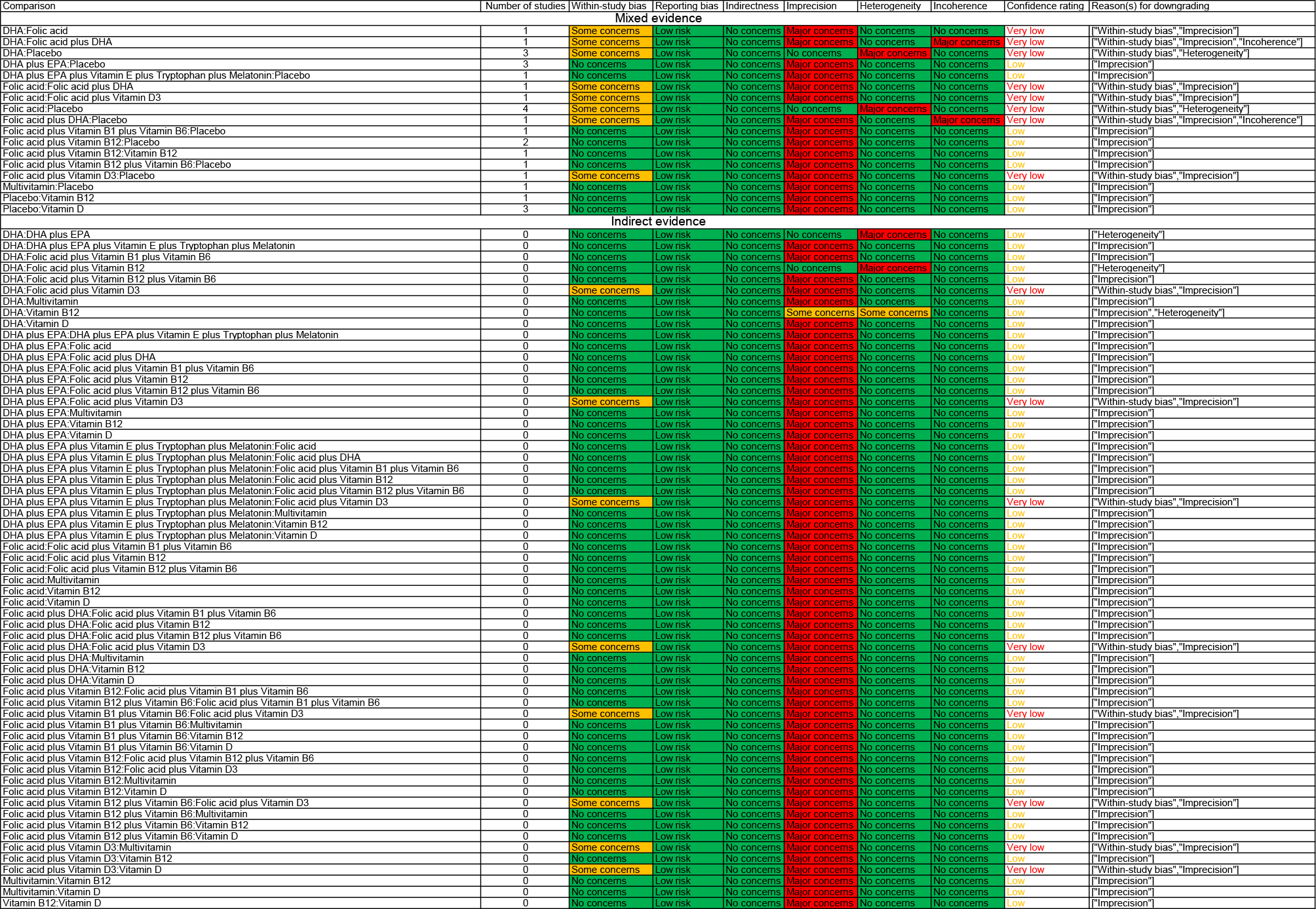
eTable 3B Attention

### eTable 3C Executive function


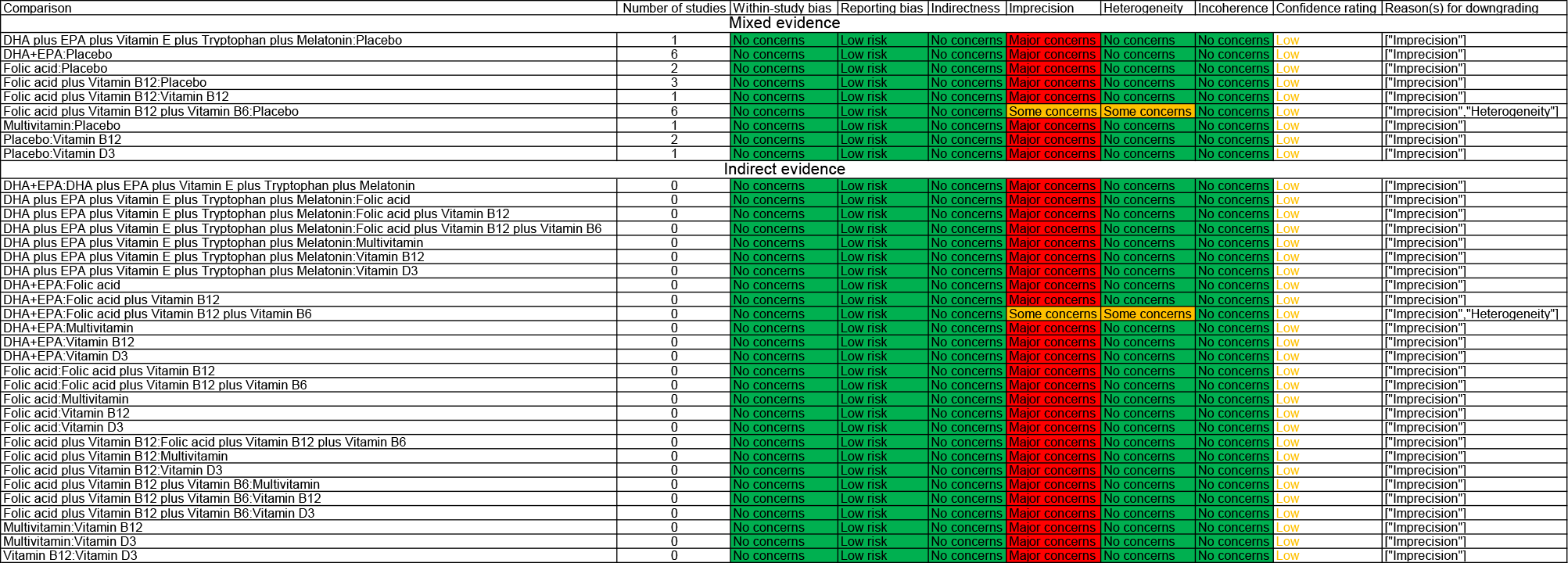


### eTable 3D Memory


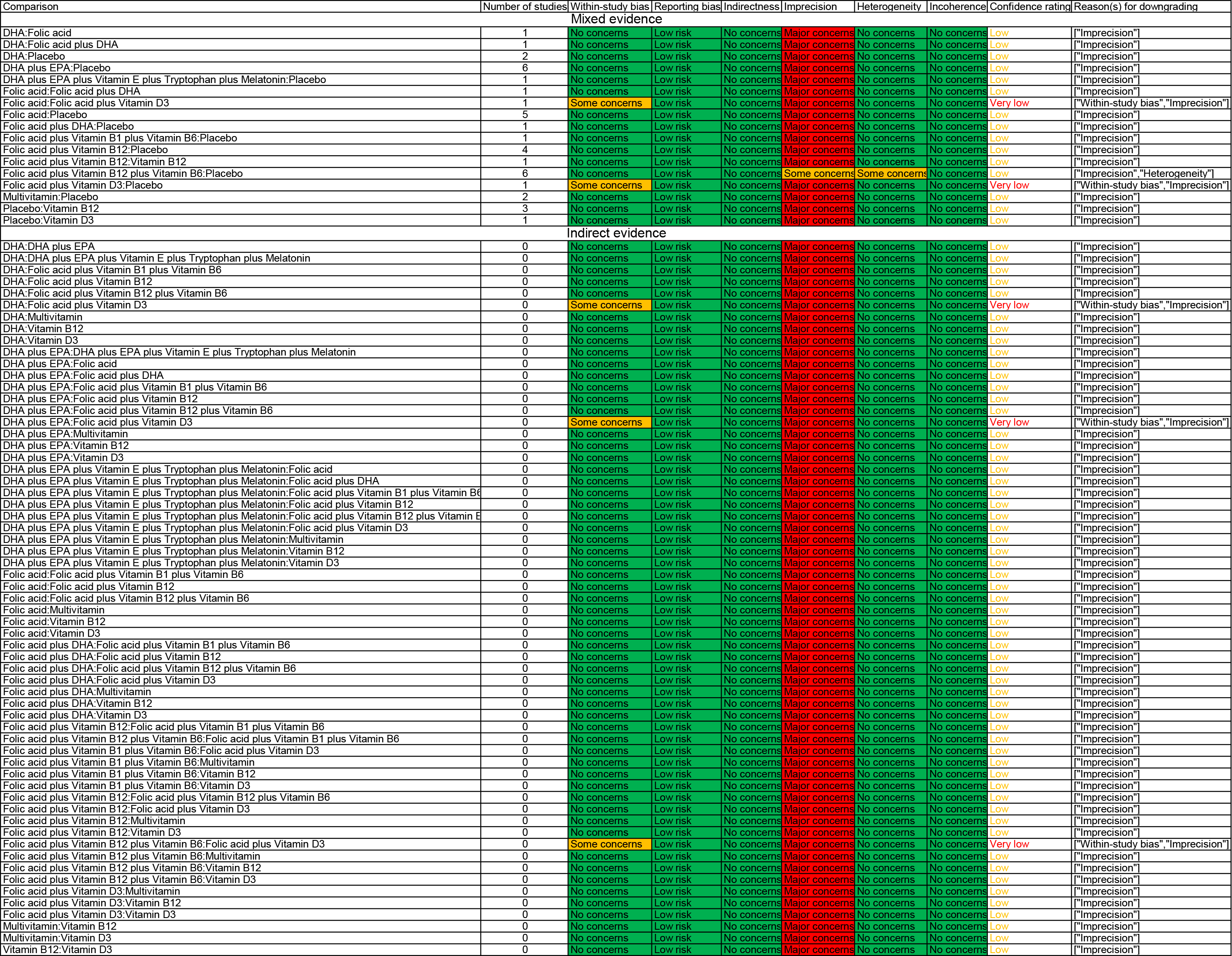


### eTable 3E Processing speed


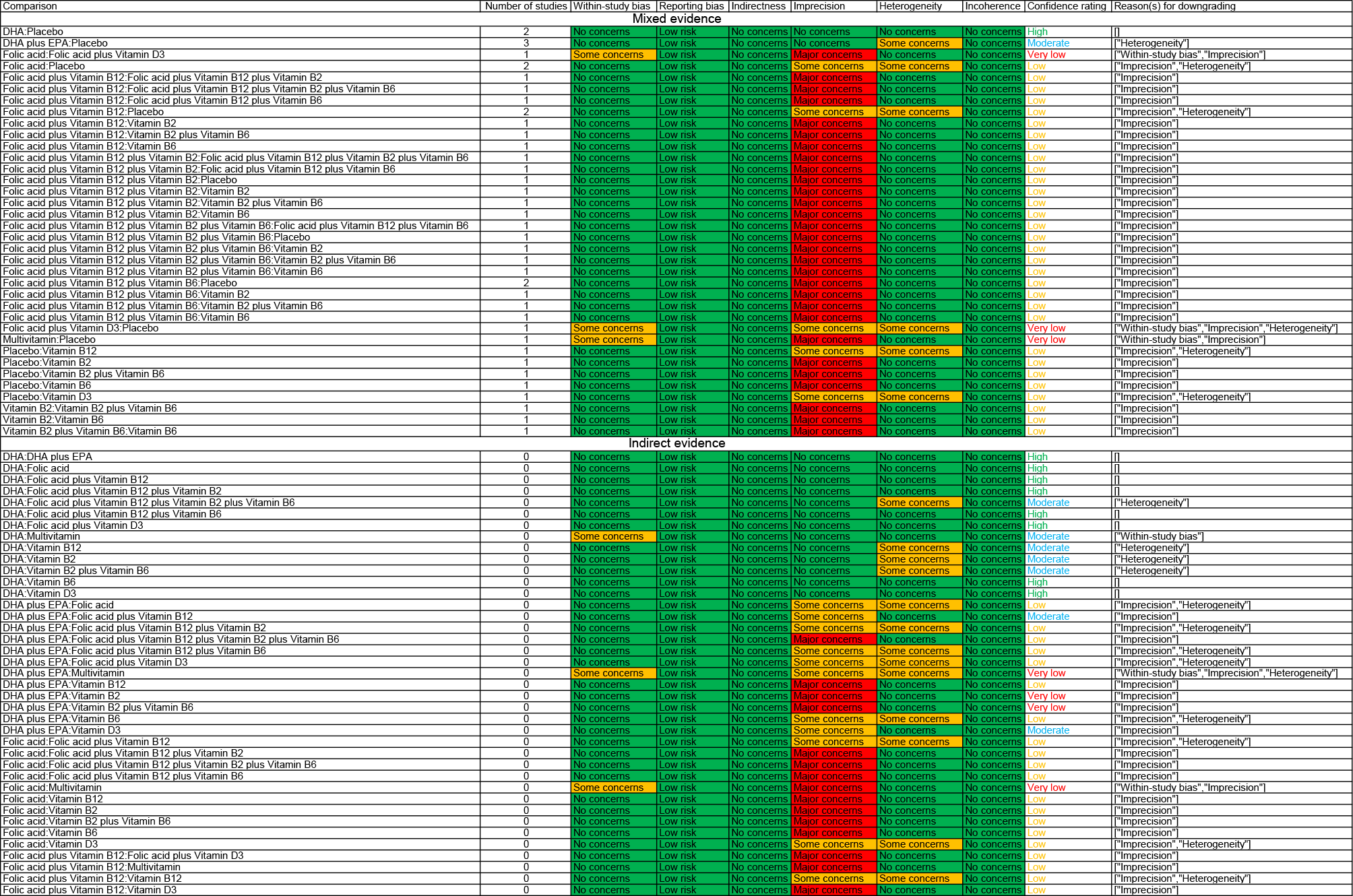


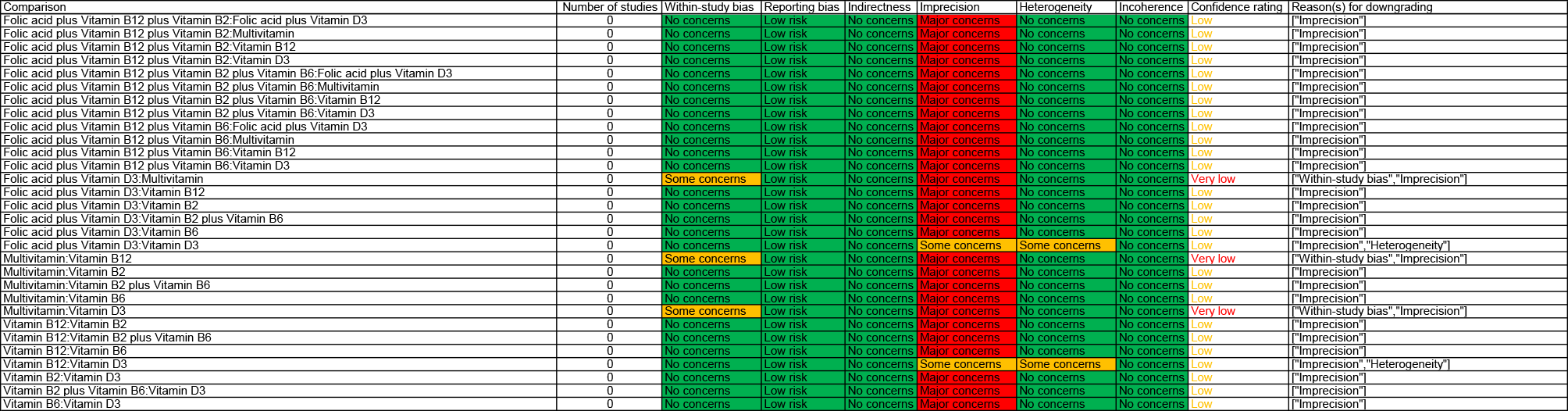


### eTable 3F Visuospatial function


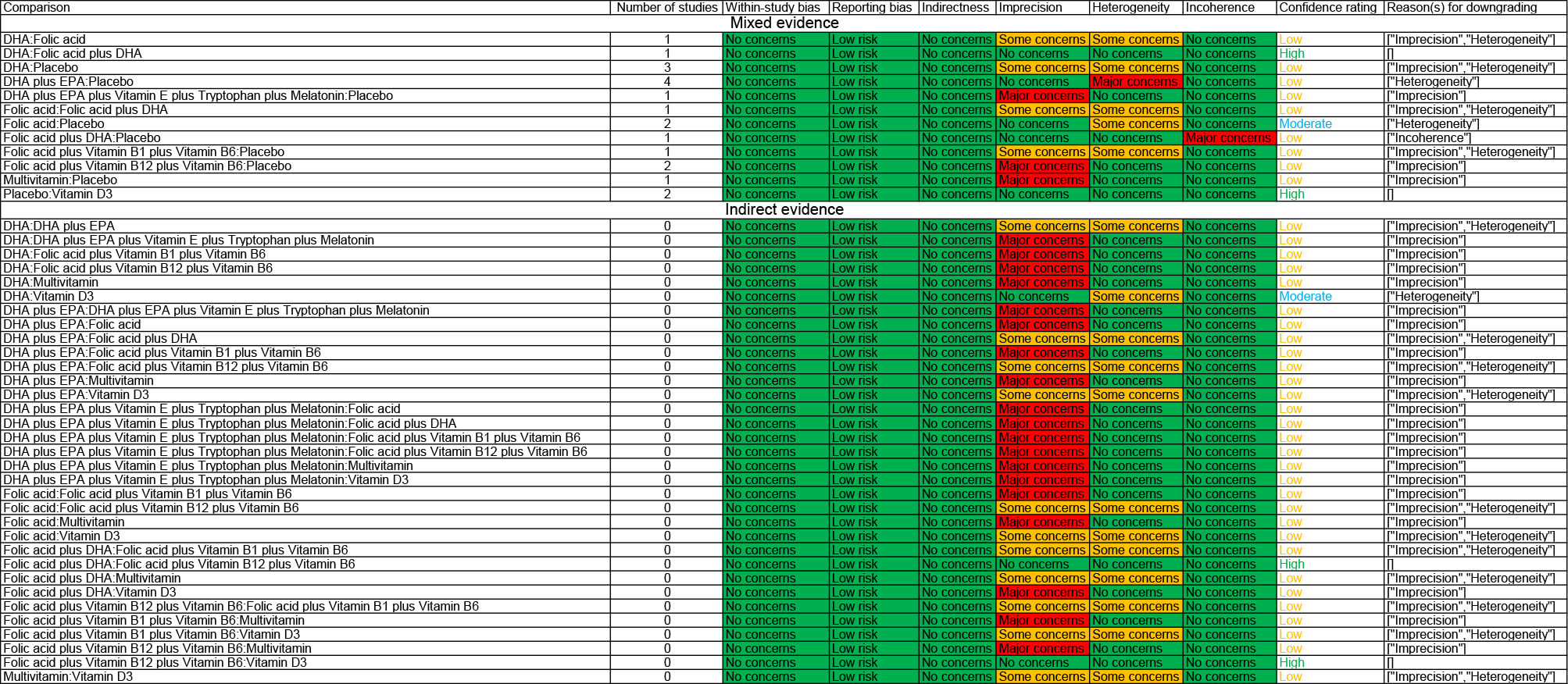


## eFigure 1 Risk of bias assessment for included studies using the ROB2


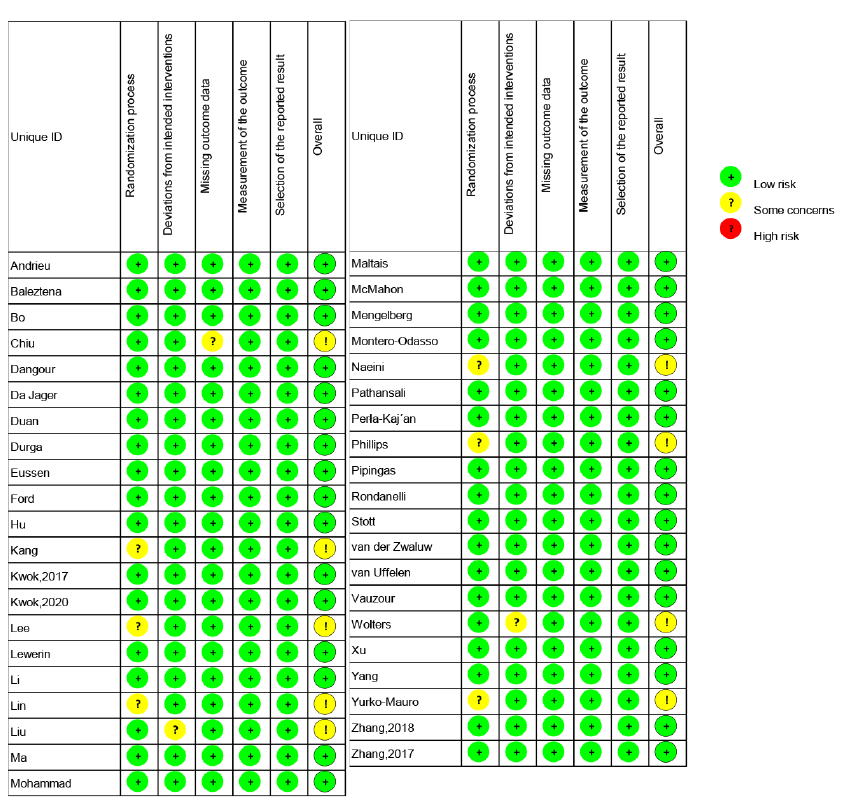


## Part Ⅰ Cognitive function assessment: Global cognition

### eFigure 2A Global cognition (All): Node splitting analysis


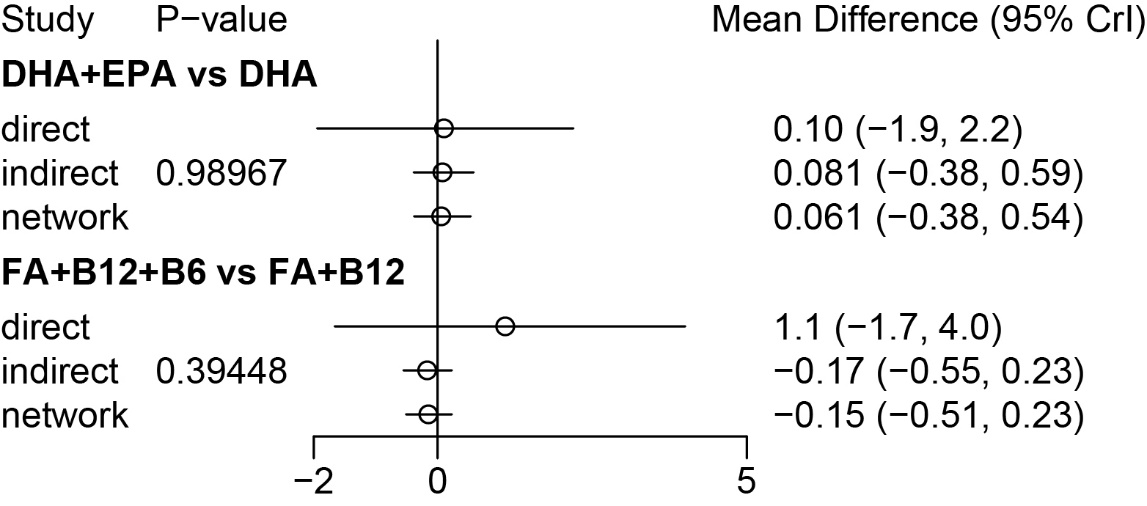


### eFigure 2B Global cognition (All): Bland Altman analysis


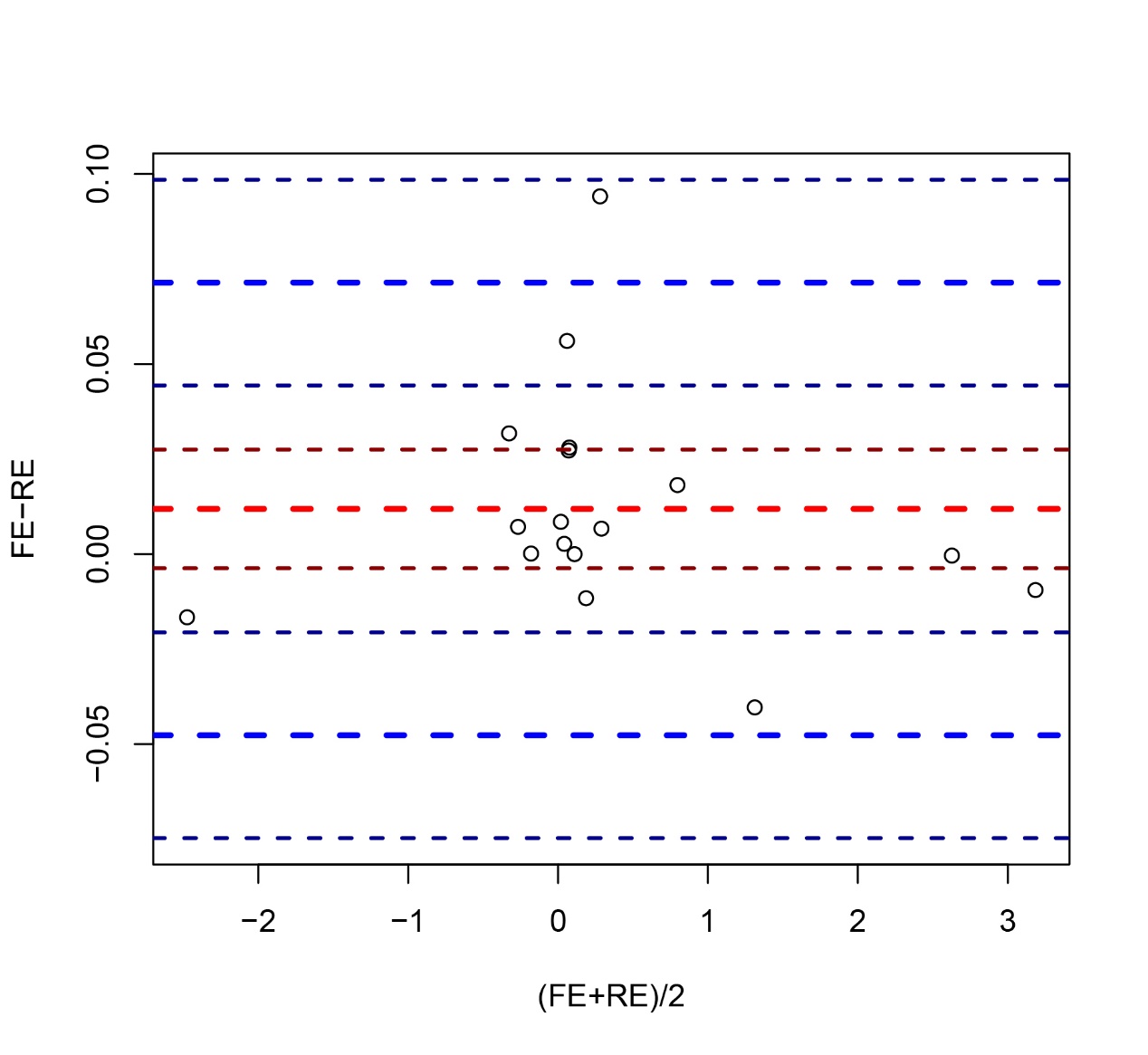


### eFigure 2C Global cognition (All): SUCRA plot


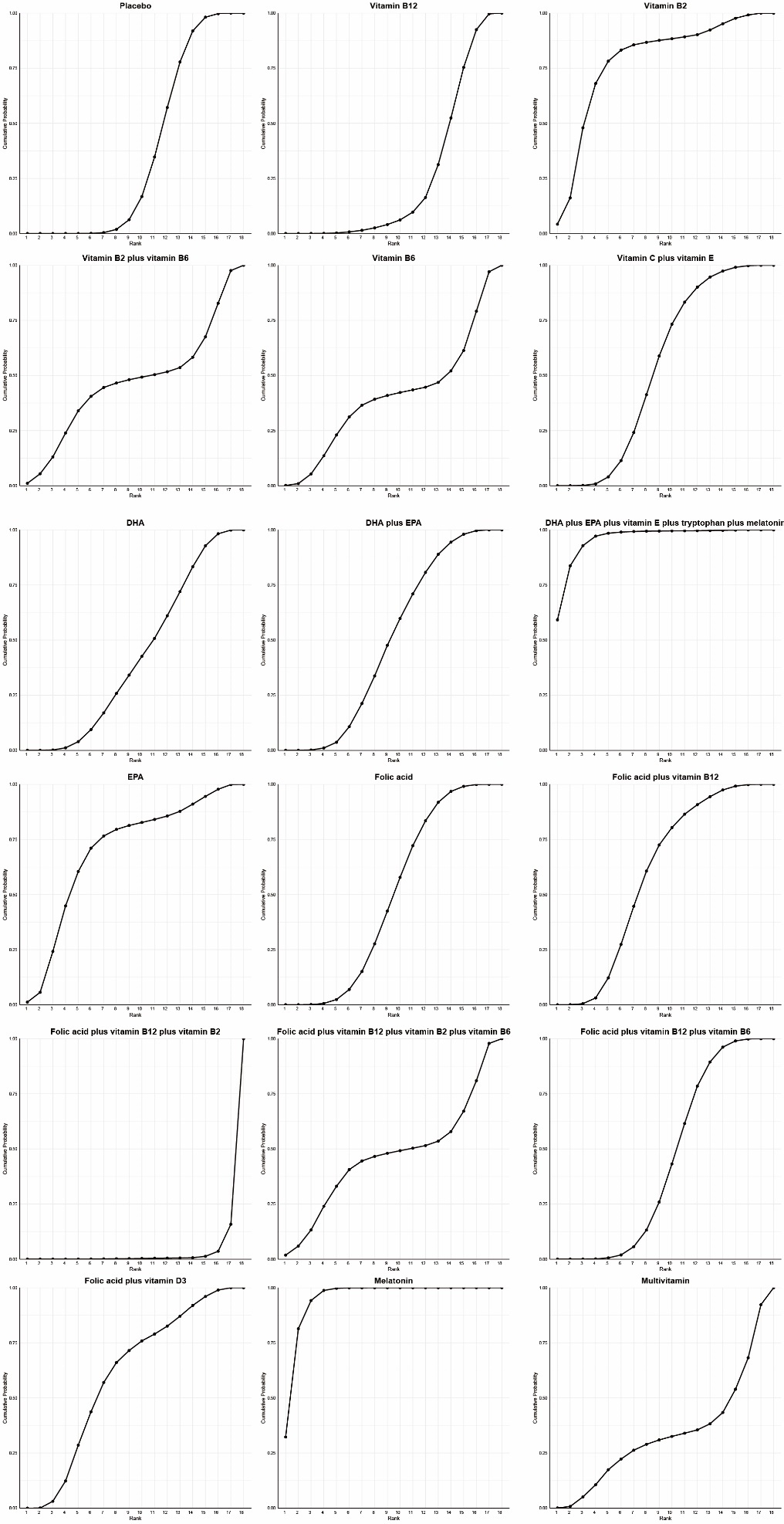


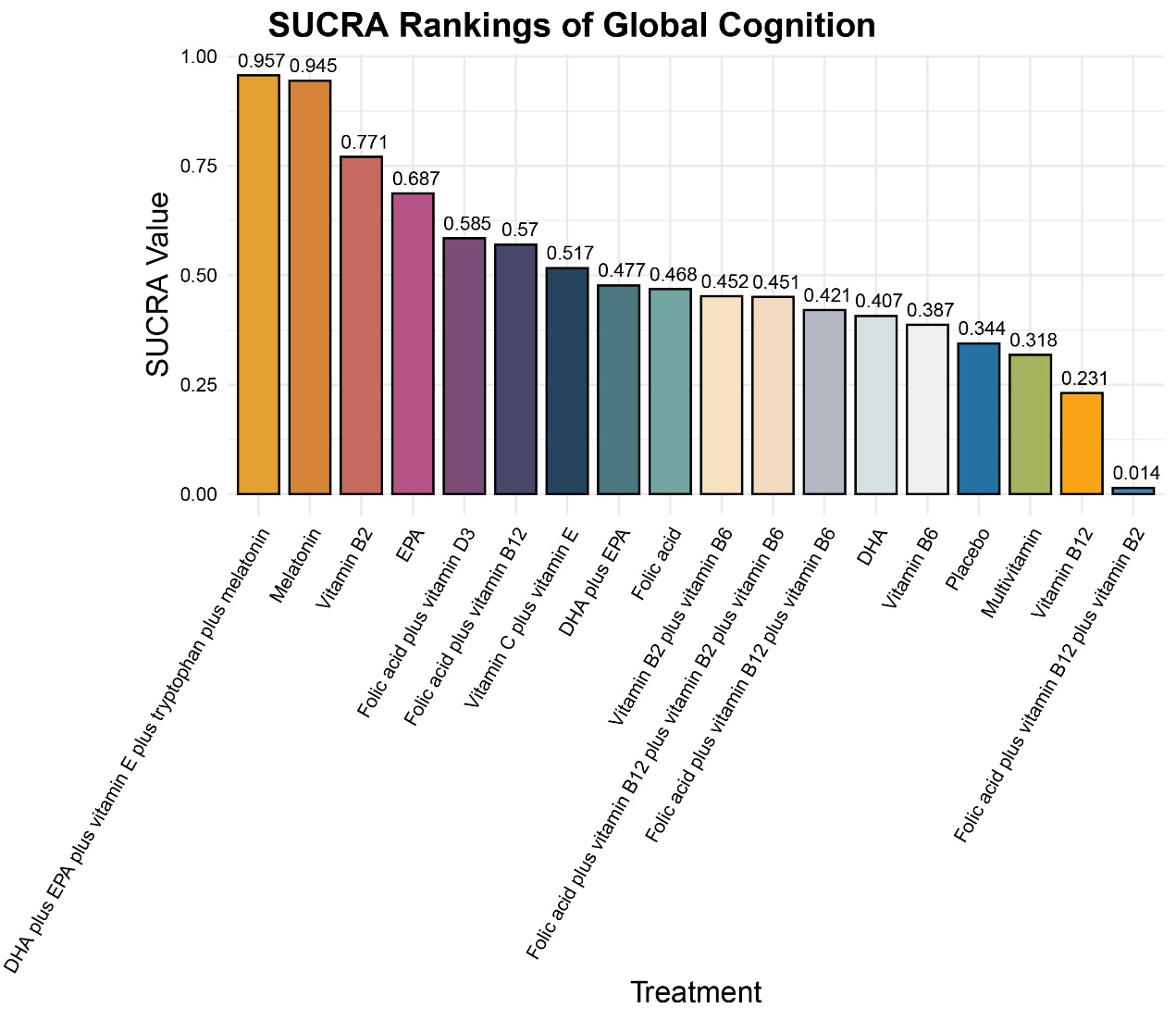


### eFigure 2D Global cognition (All): Ranking forest plot and ranking probability


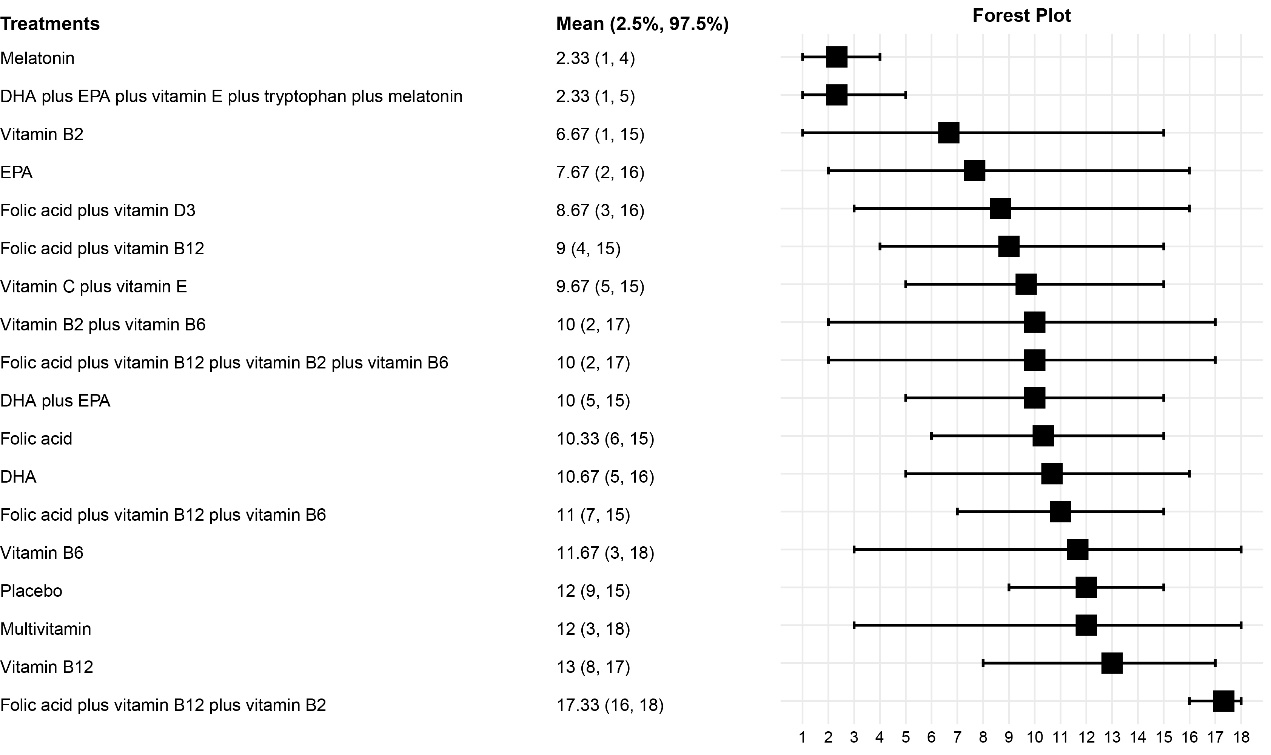


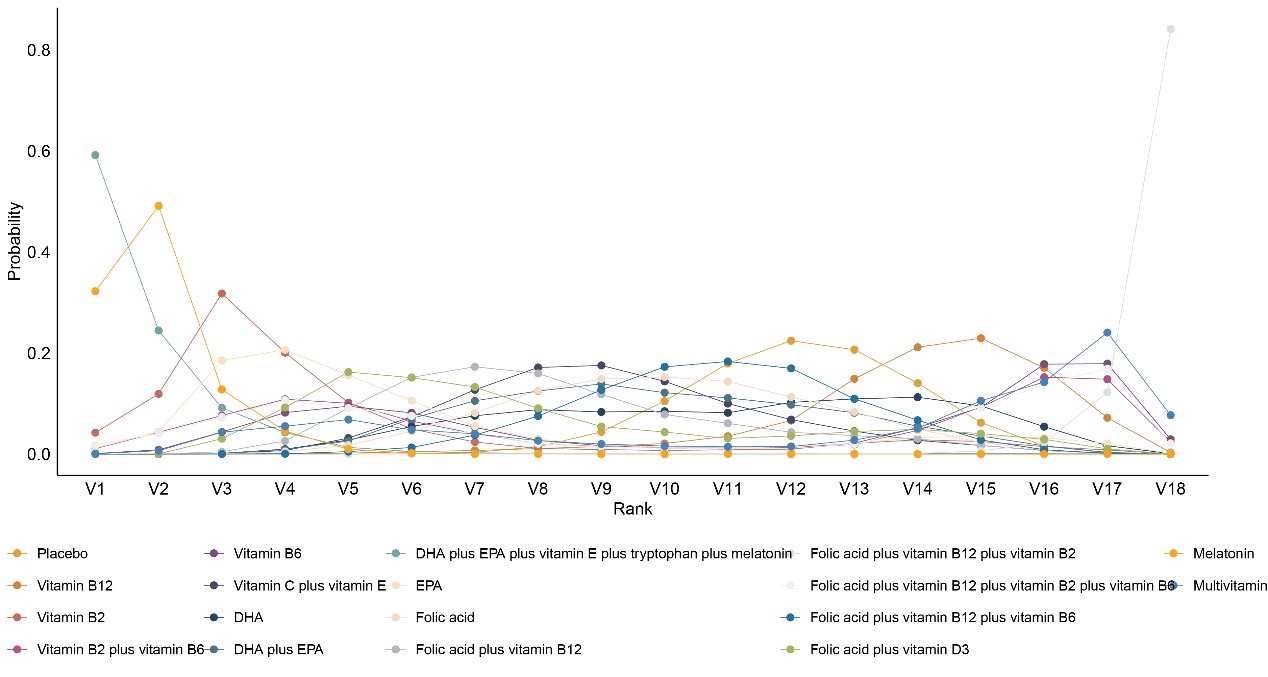


### eFigure 3A Global cognition (Subgroup analysis-Age): A, Network plot; B, funnel plot; C, forest plot.


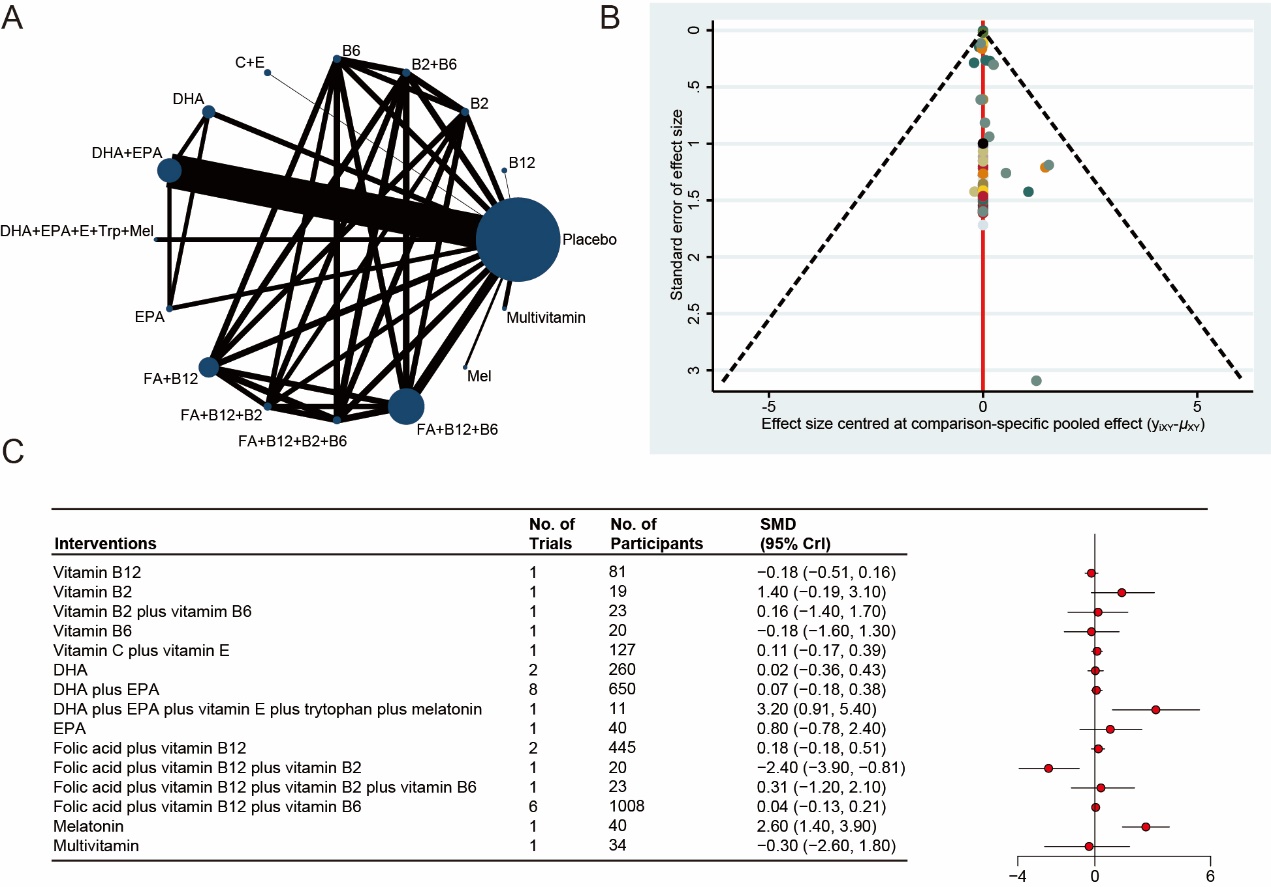


### eFigure 3B Global cognition (Subgroup analysis-Age): Node splitting analysis


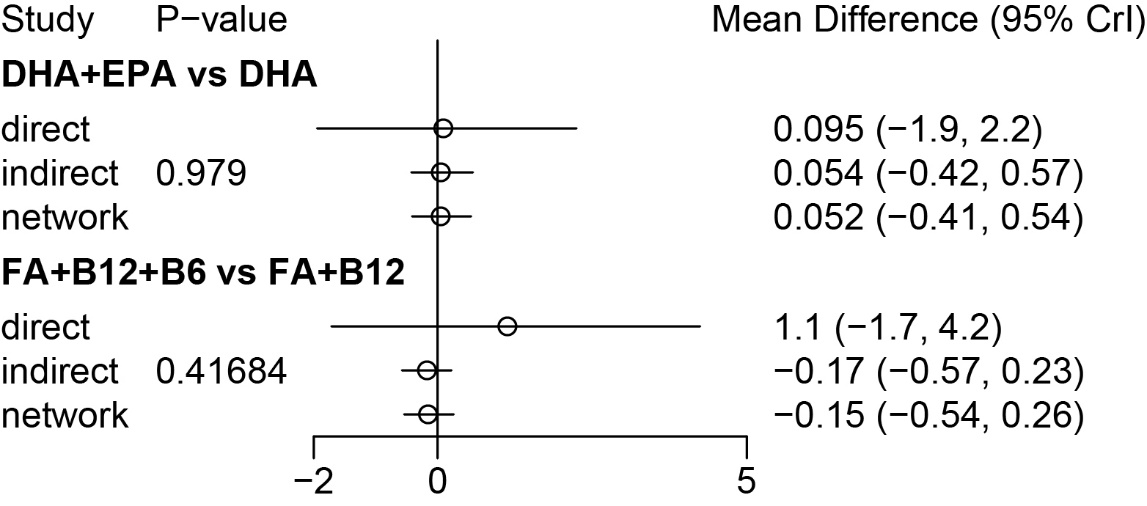


### eFigure 3C Global cognition (Subgroup analysis-Age): SUCRA plot


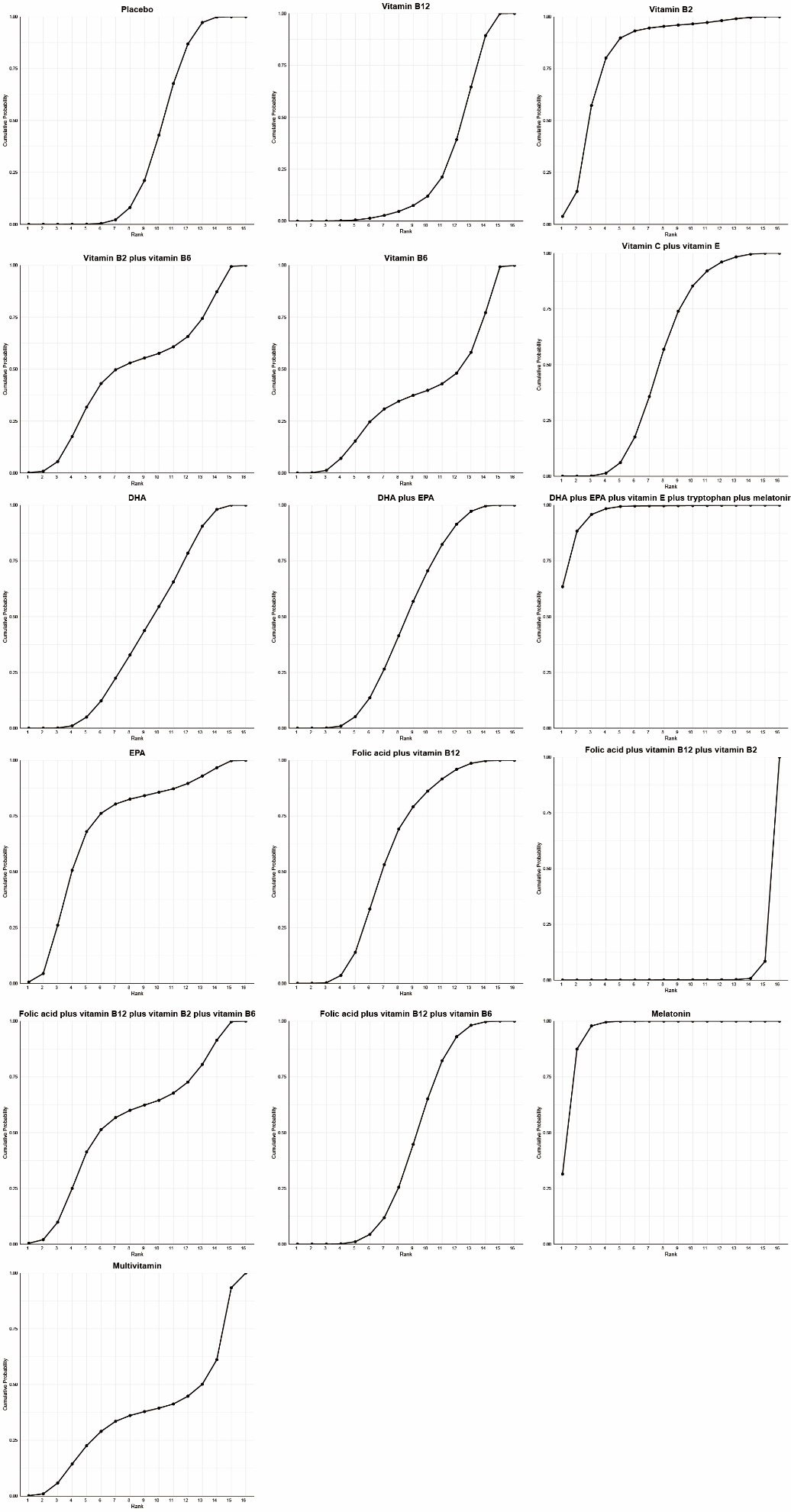


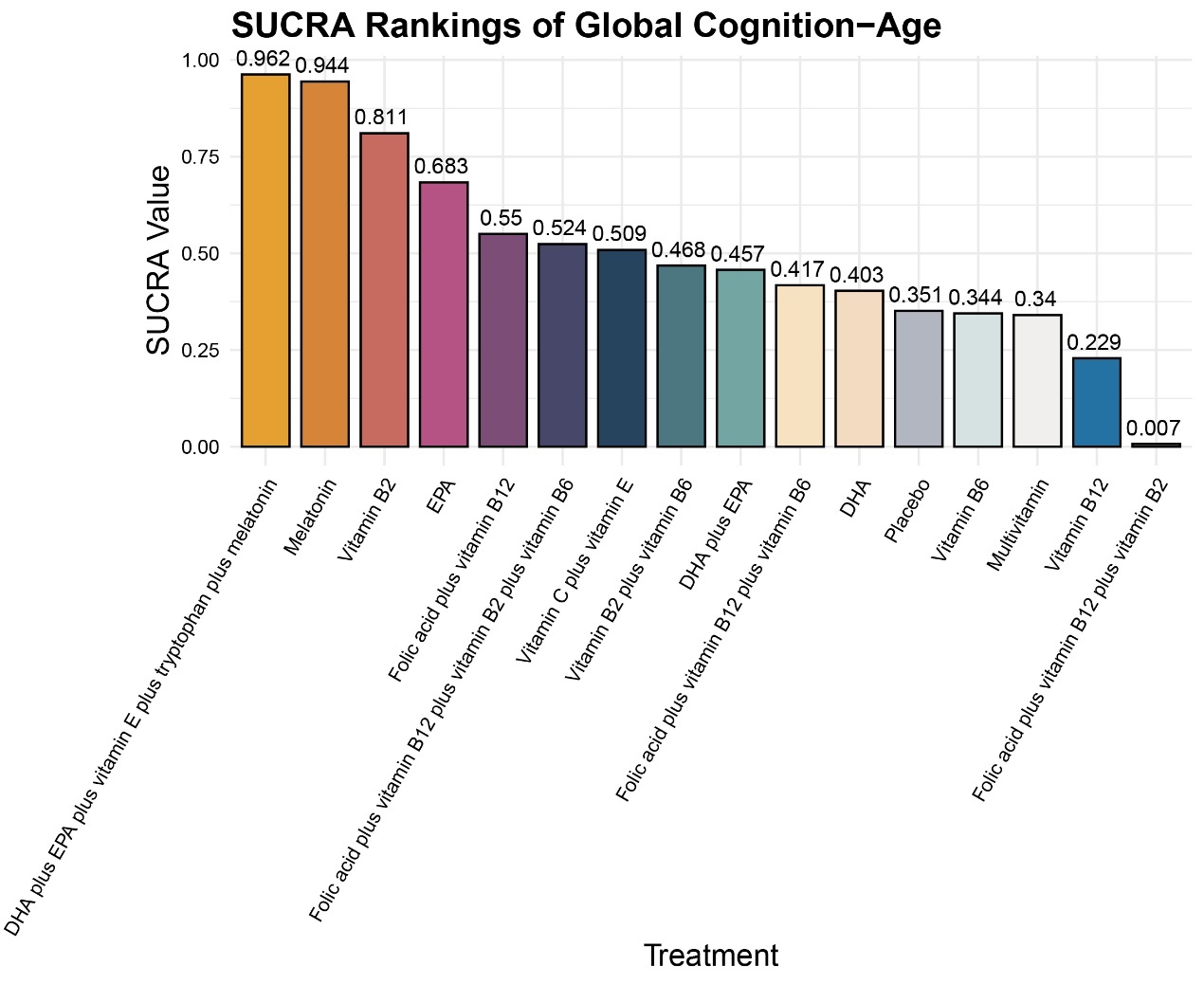


### eFigure 3D Global cognition (Subgroup analysis-Age): Ranking forest plot and ranking probability


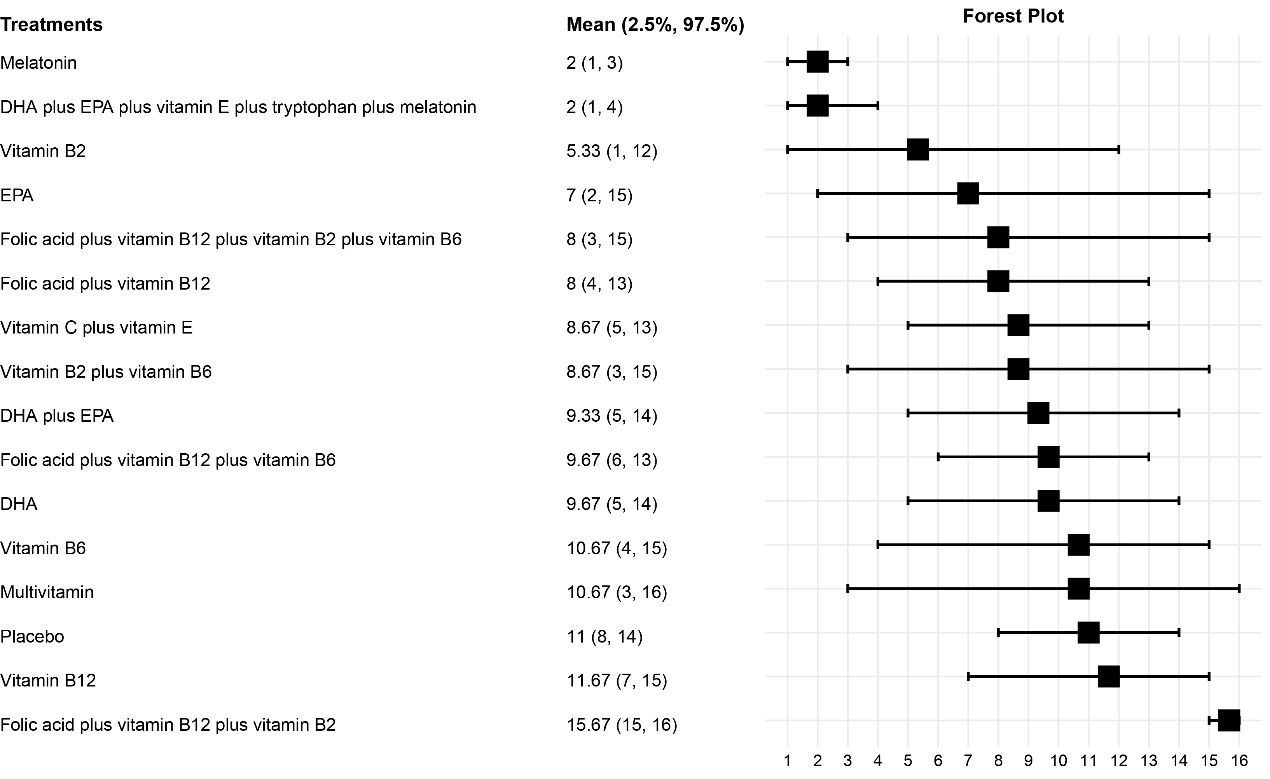


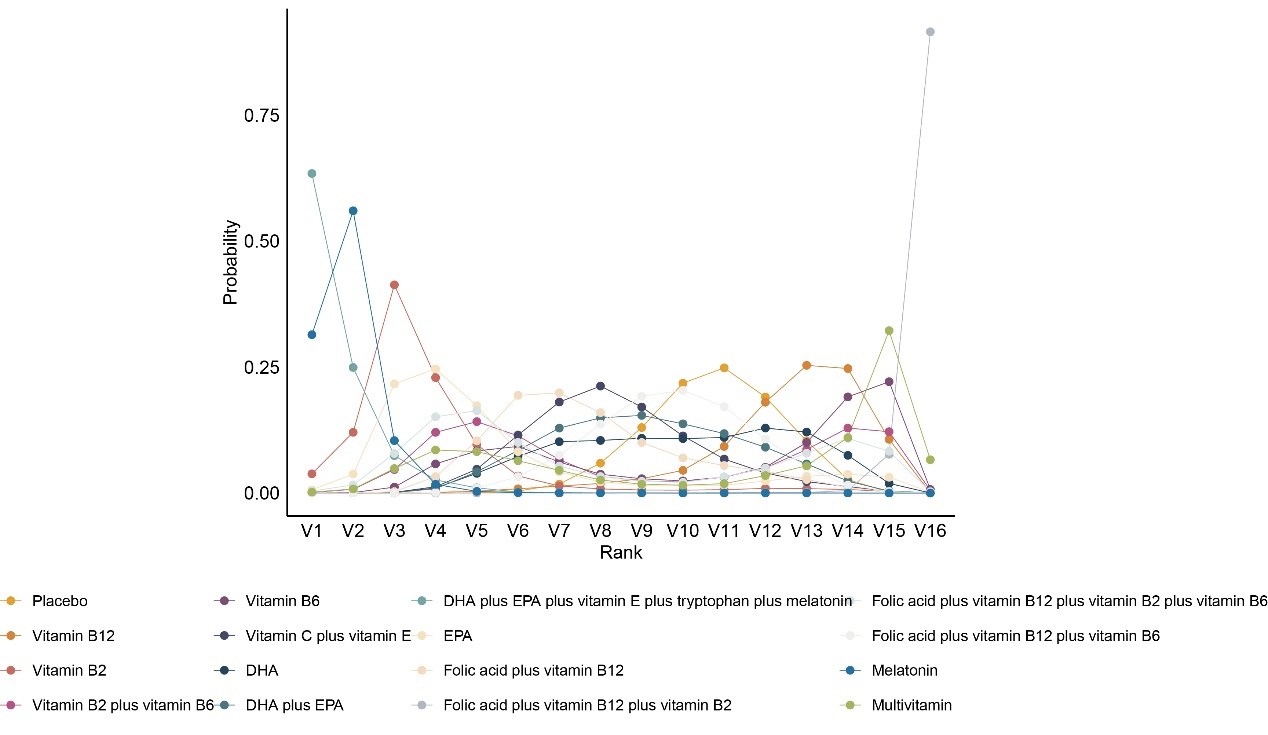


### eFigure 4A Global cognition (Subgroup analysis-MCI): A, Network plot; B, funnel plot; C, forest plot.


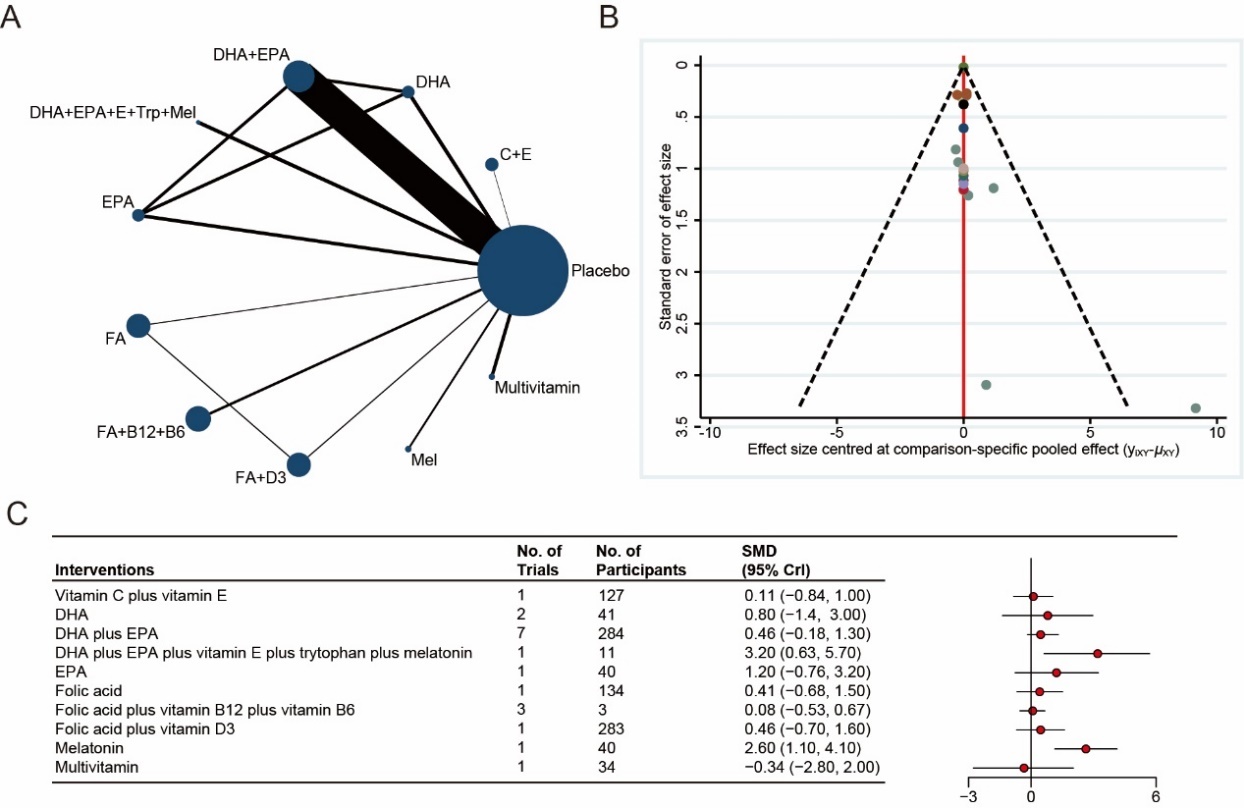


### eFigure 4B Global cognition (Subgroup analysis-MCI): SUCRA plot


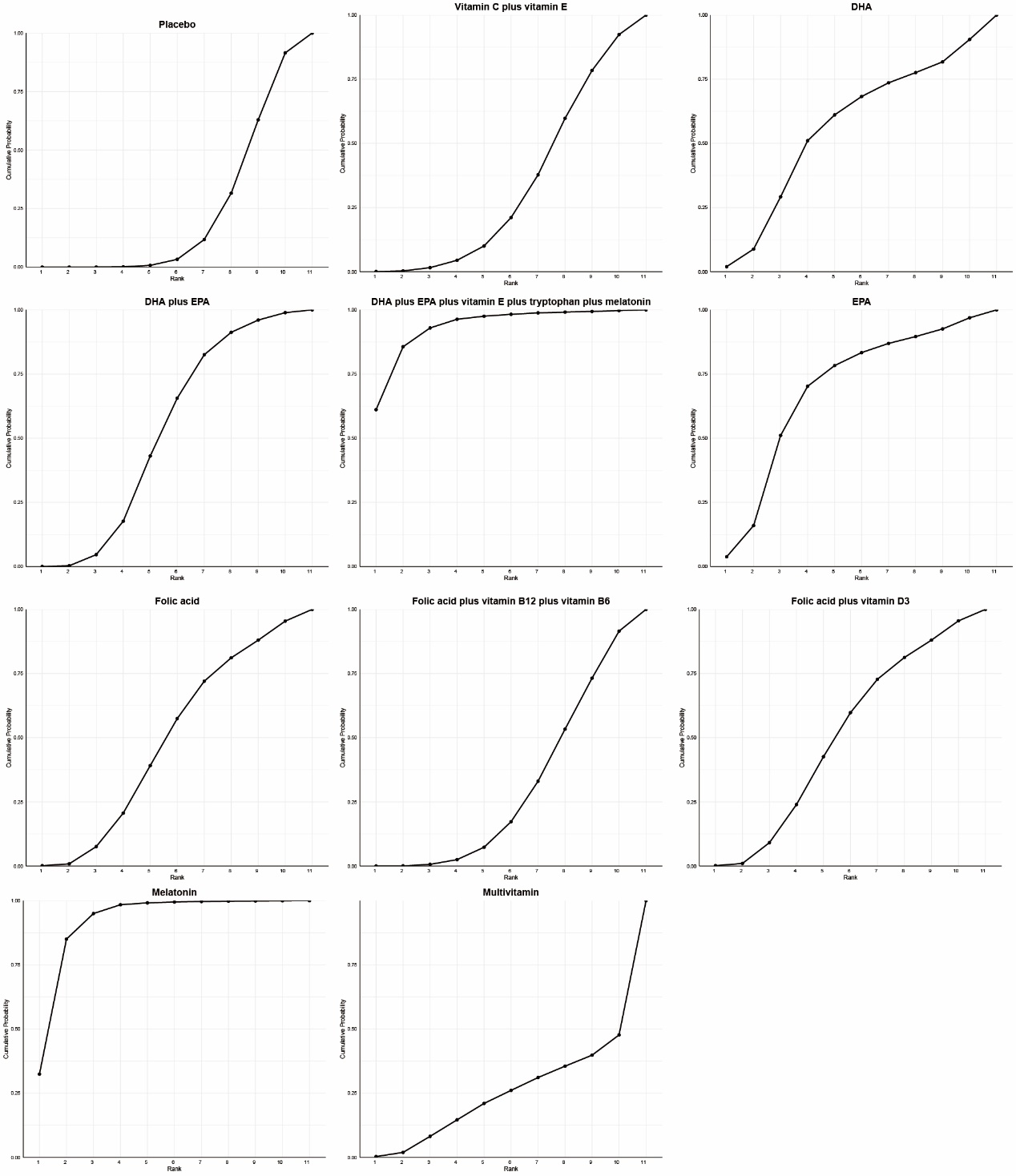


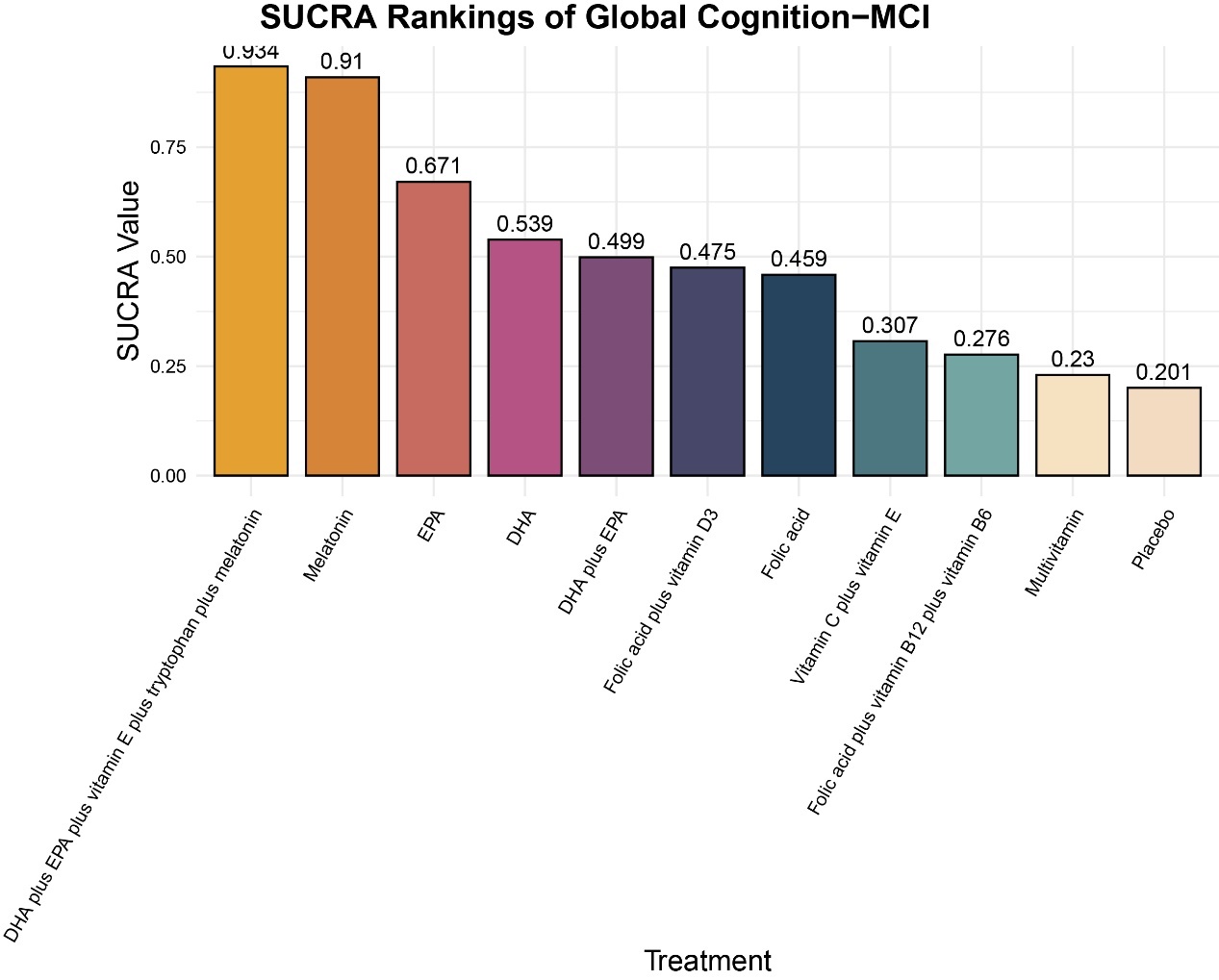


### eFigure 4C Global cognition (Subgroup analysis-MCI): Ranking forest plot and ranking probability


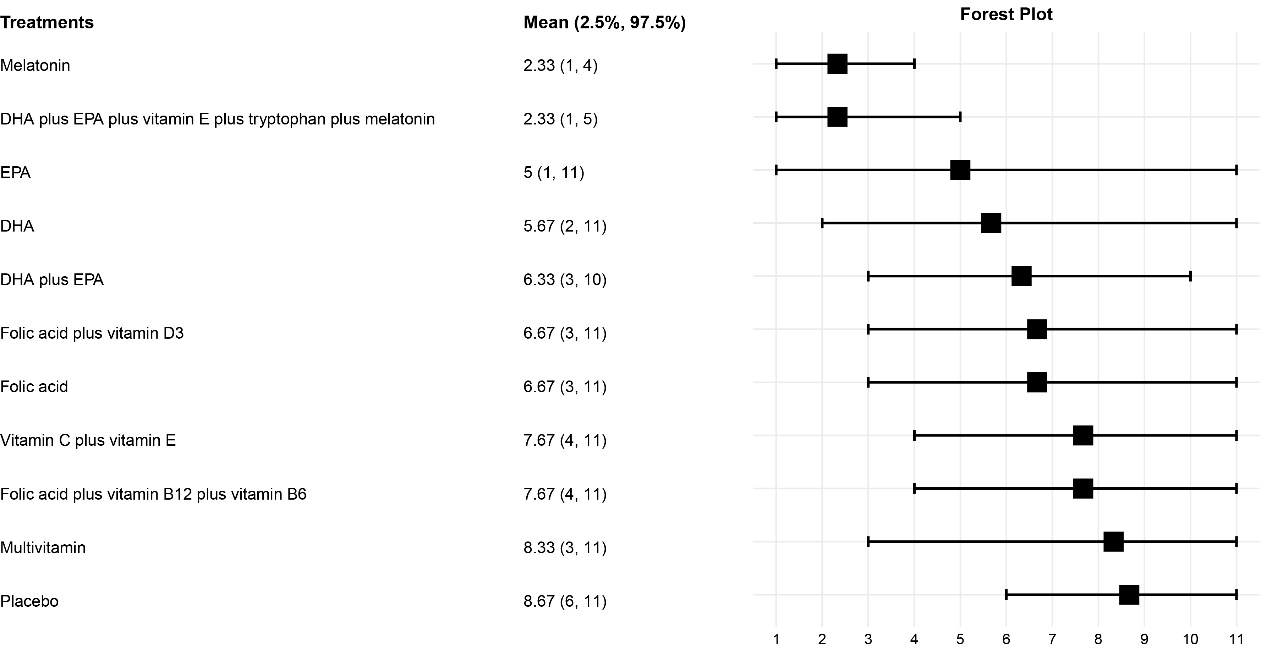


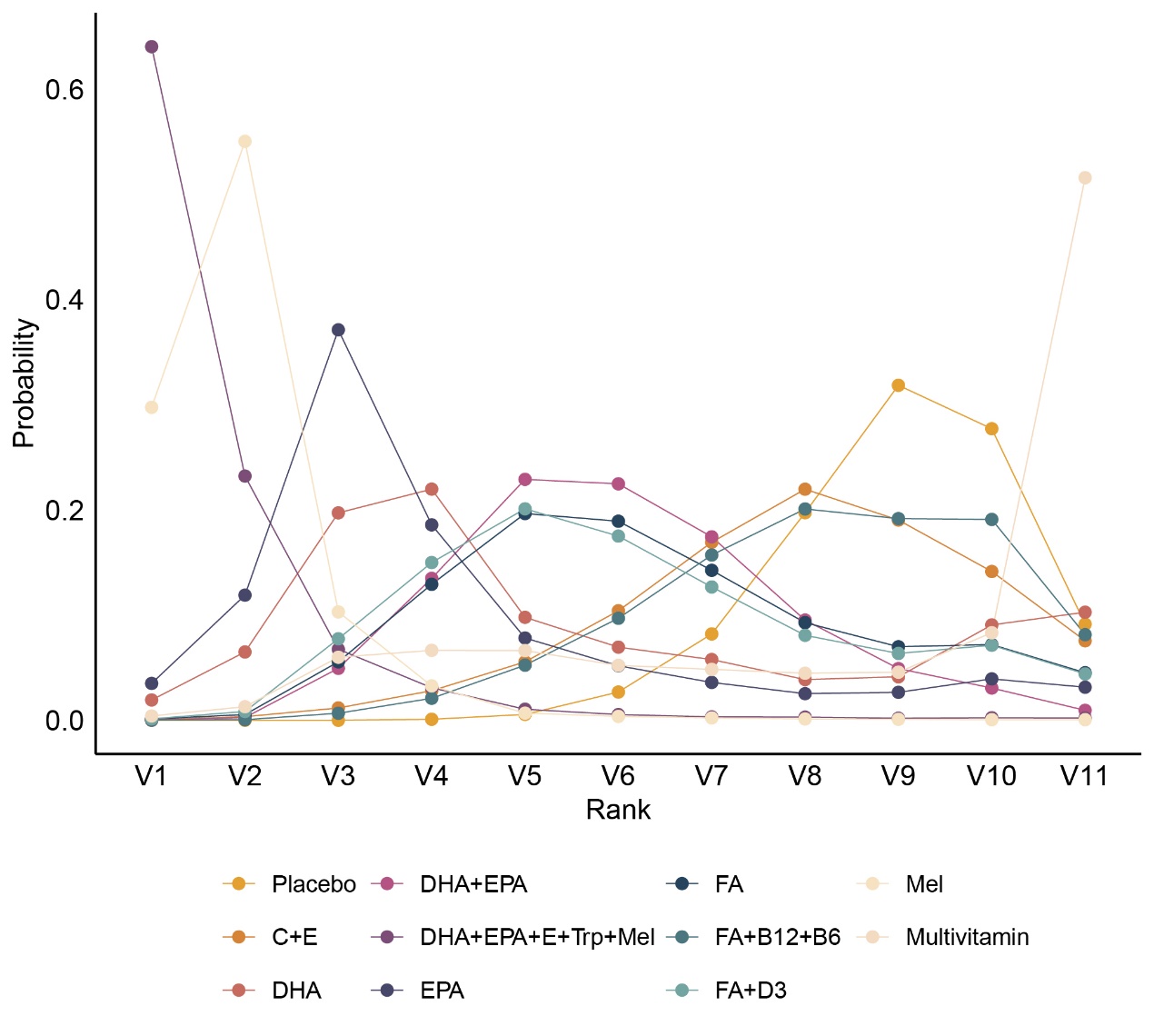


### eFigure 5A Global cognition (Subgroup analysis-Sample size over 100): Network plot


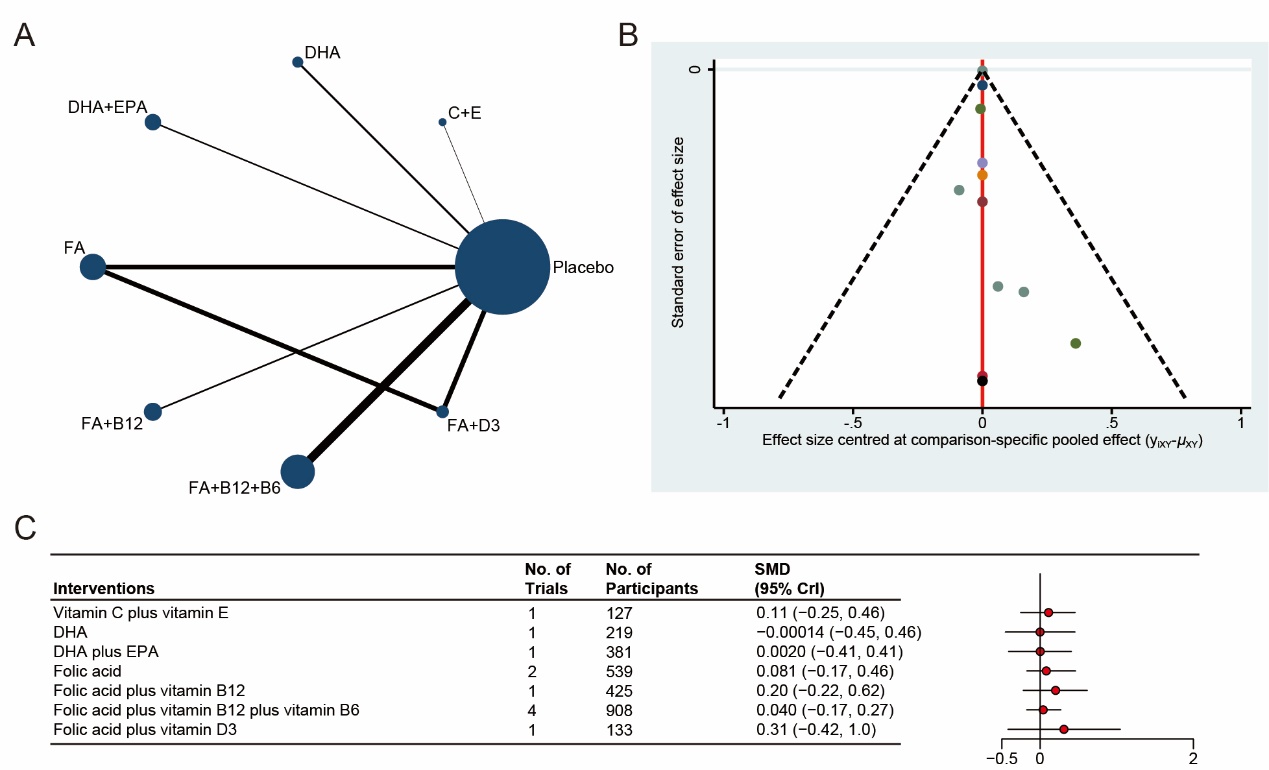


### eFigure 5B Global cognition (Subgroup analysis-Sample size over 100): SUCRA plot


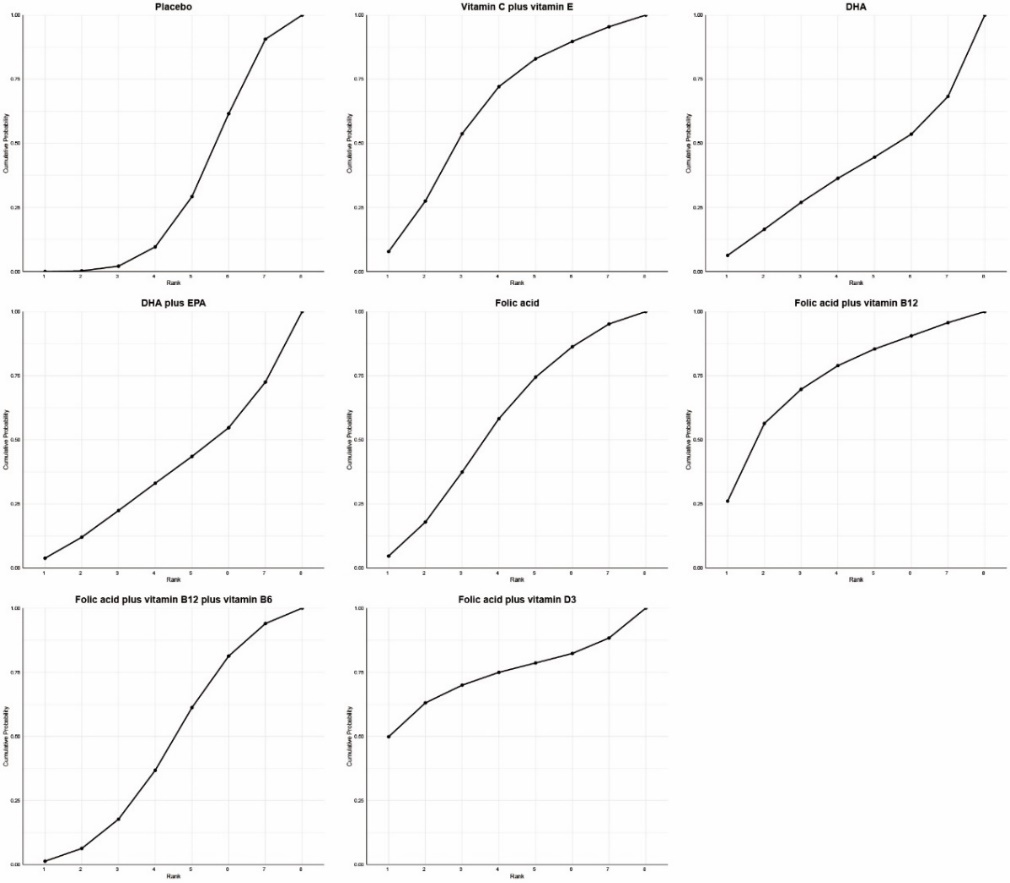


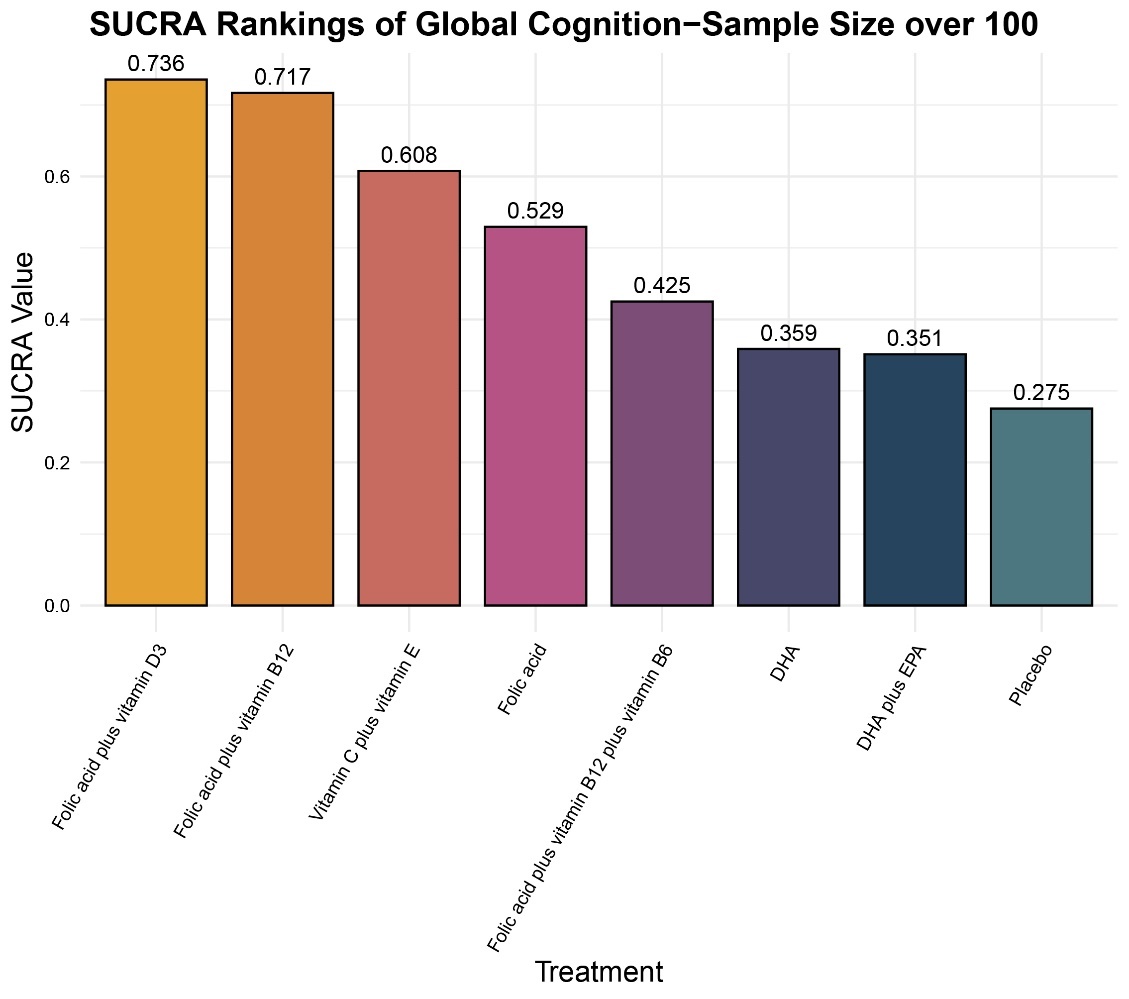


### eFigure 5C Global cognition (Subgroup analysis-Sample size over 100): Ranking forest plot and ranking probability


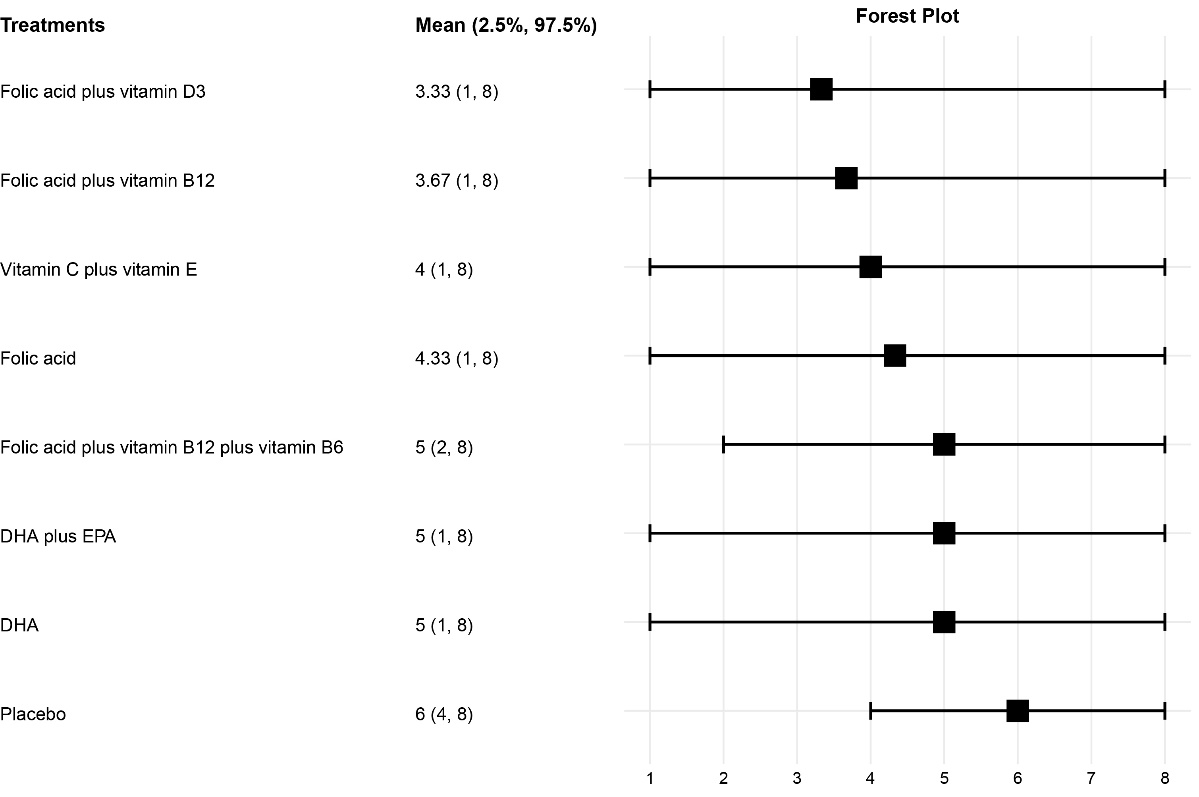


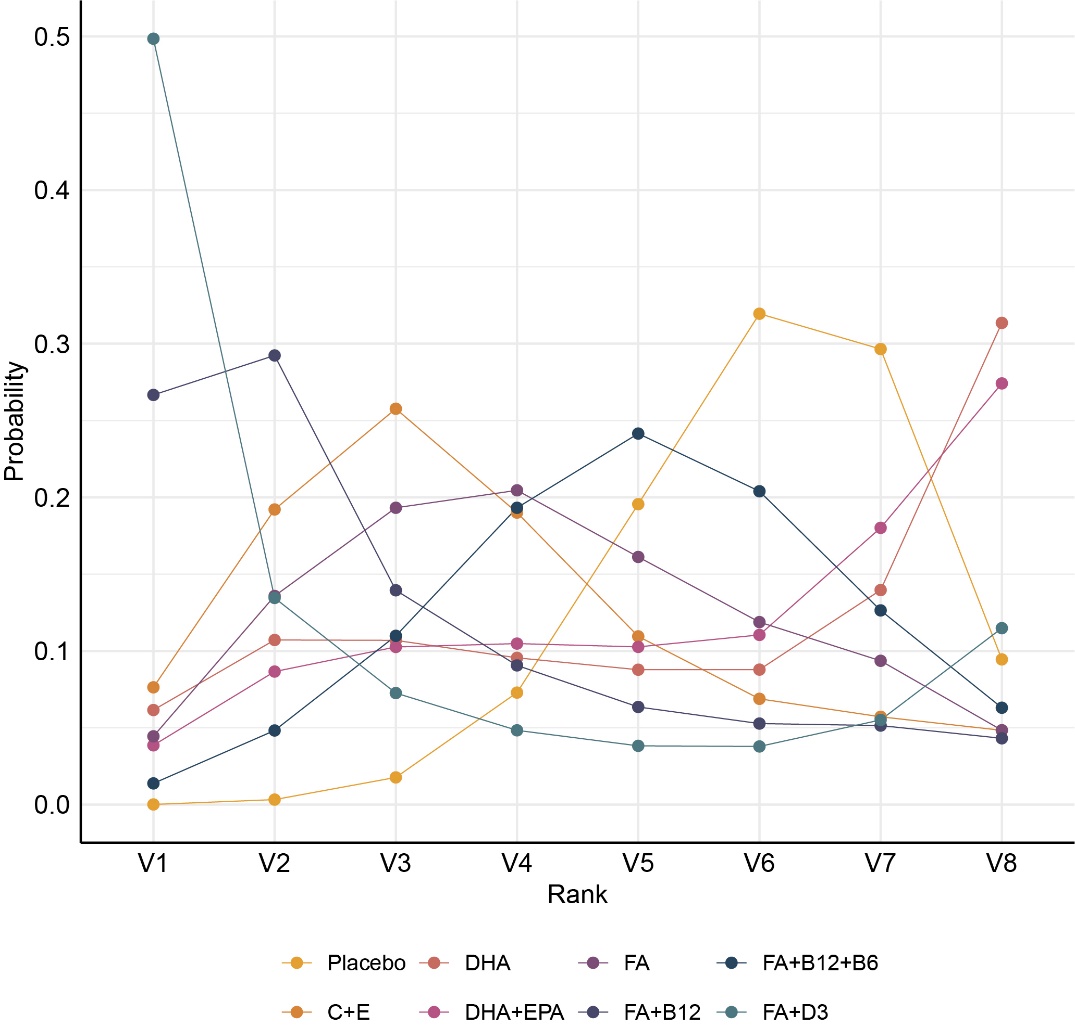


## Part Ⅱ Cognitive function assessment: Attention

### eFigure 6A Attention (All): Node splitting analysis


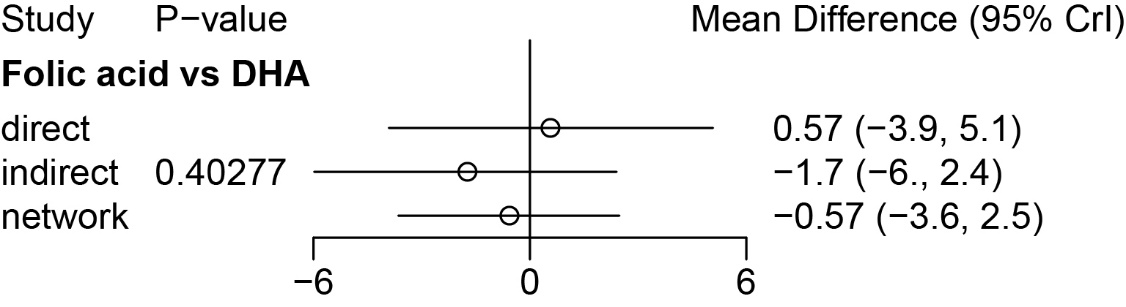


### eFigure 6B Attention (All): Bland Altman analysis


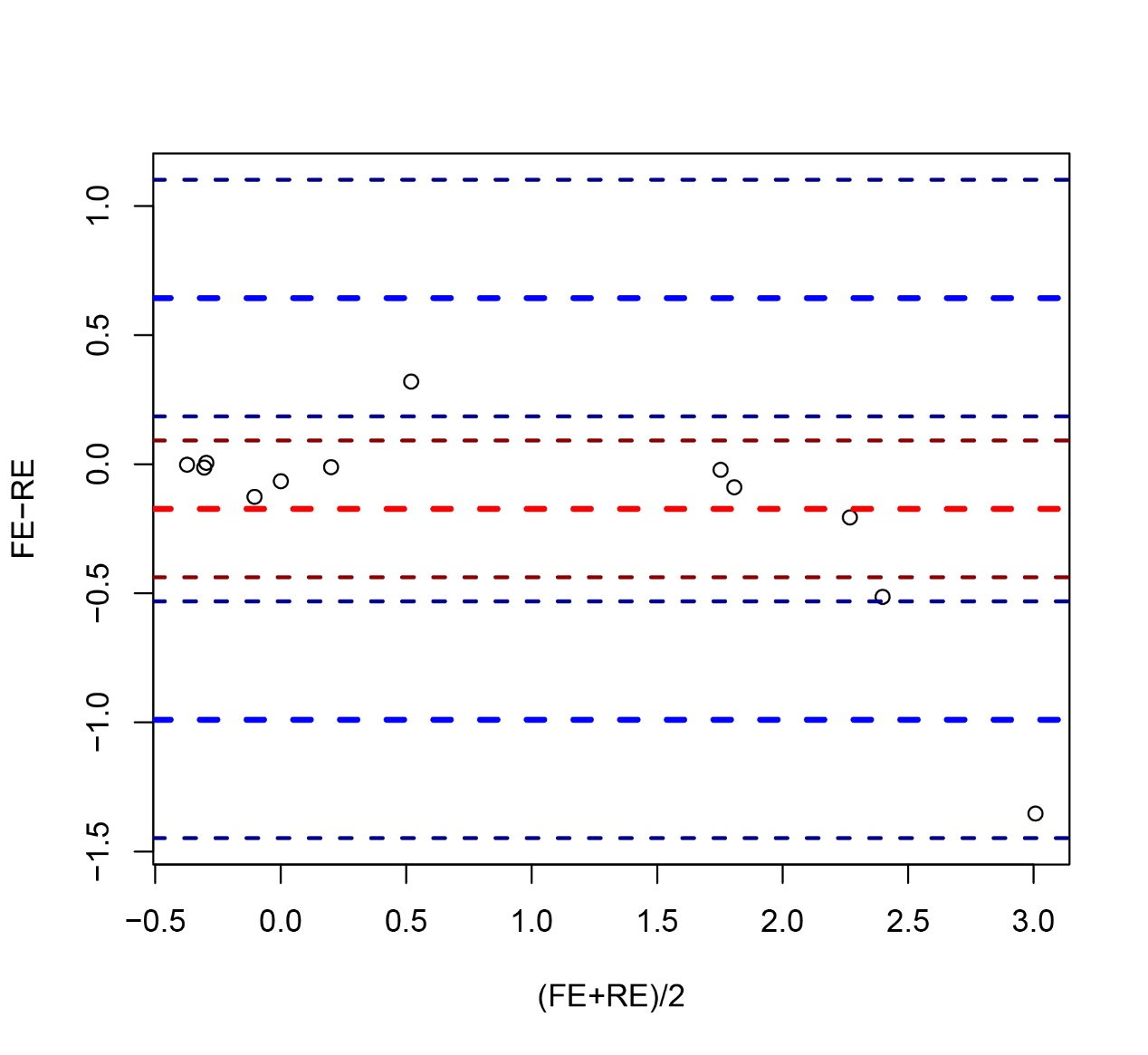


### eFigure 6C Attention (All): SUCRA plot


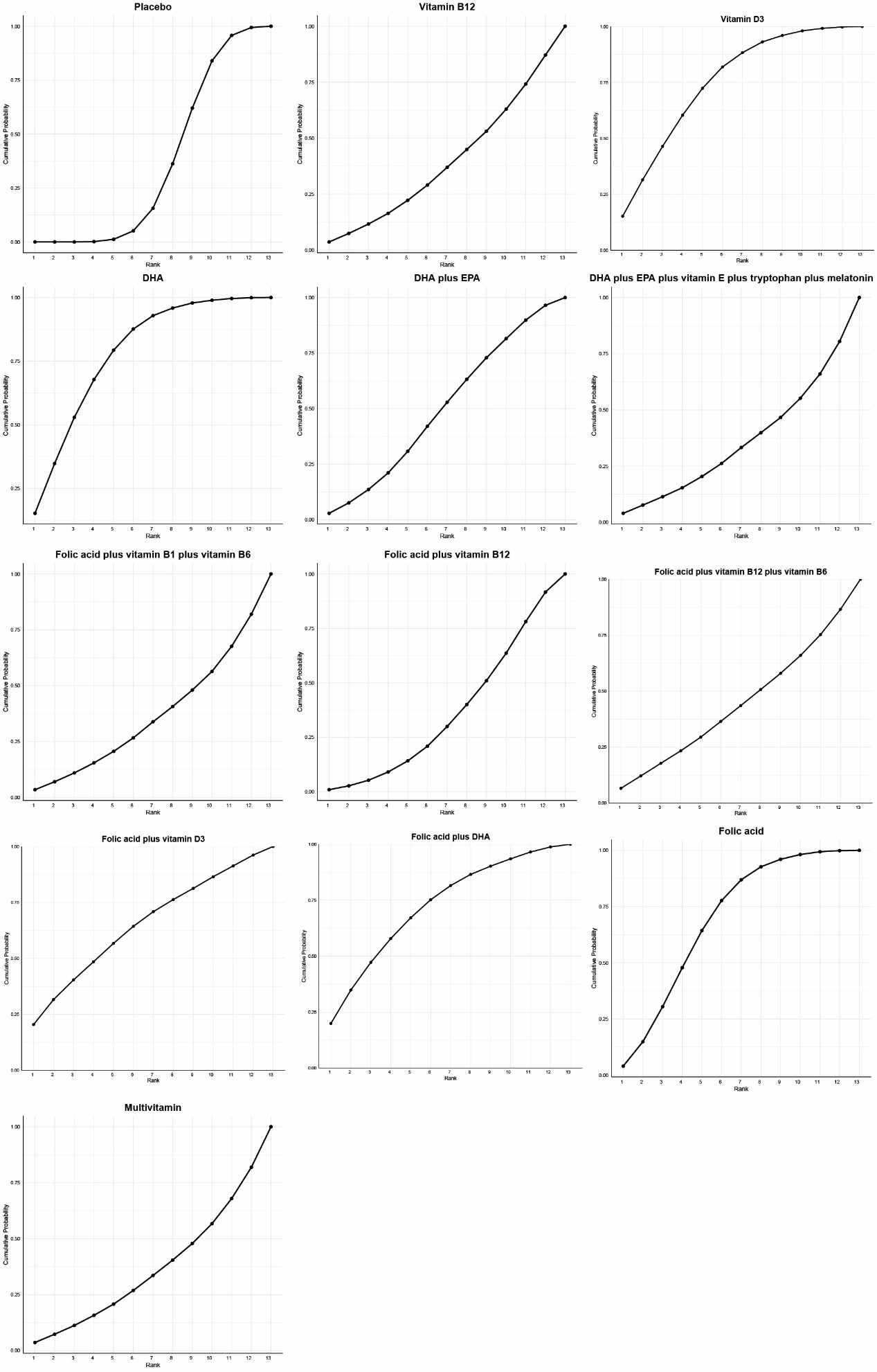


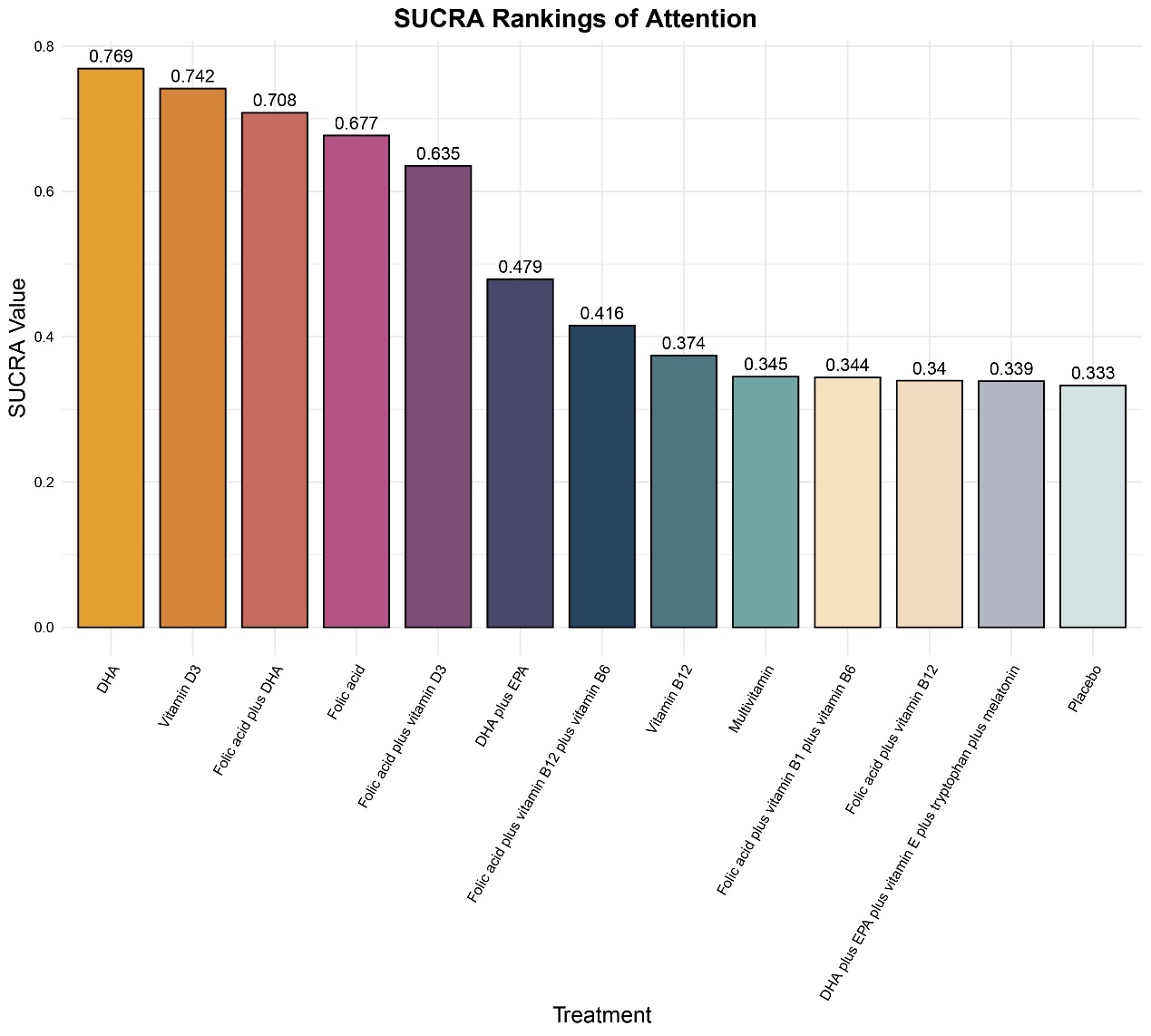


### eFigure 6D Attention (All): Ranking forest plot and ranking probability


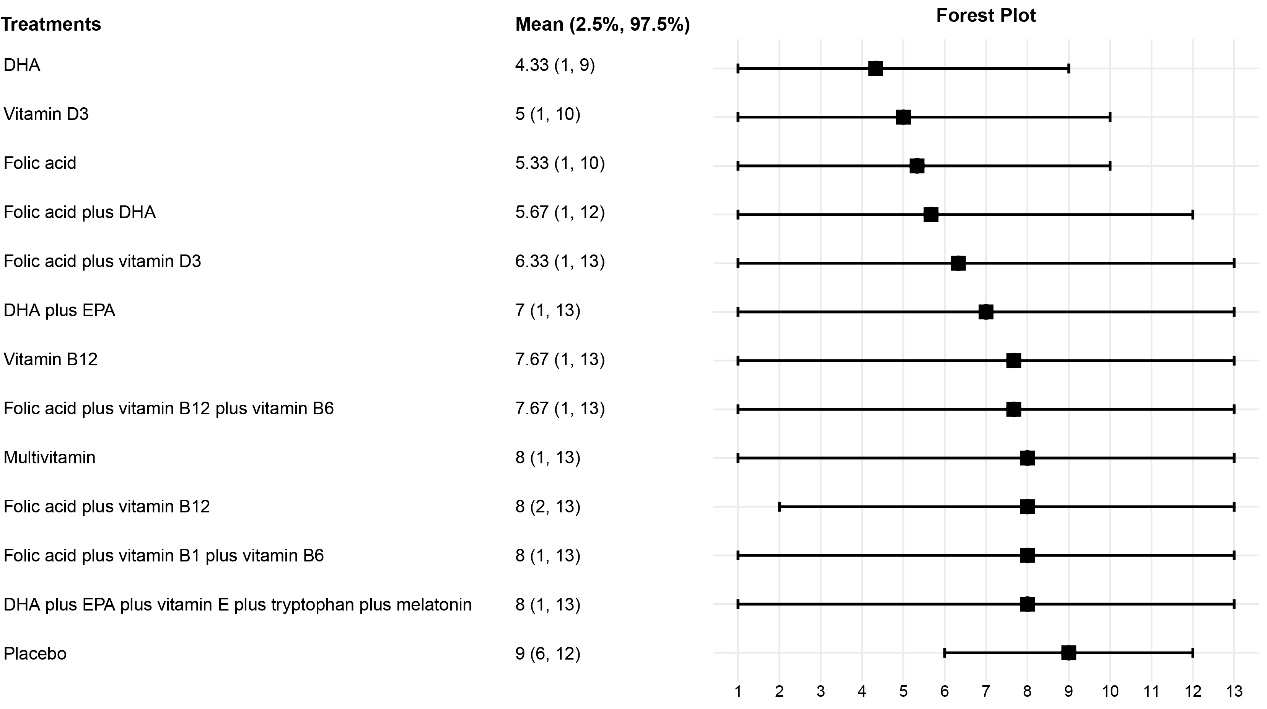


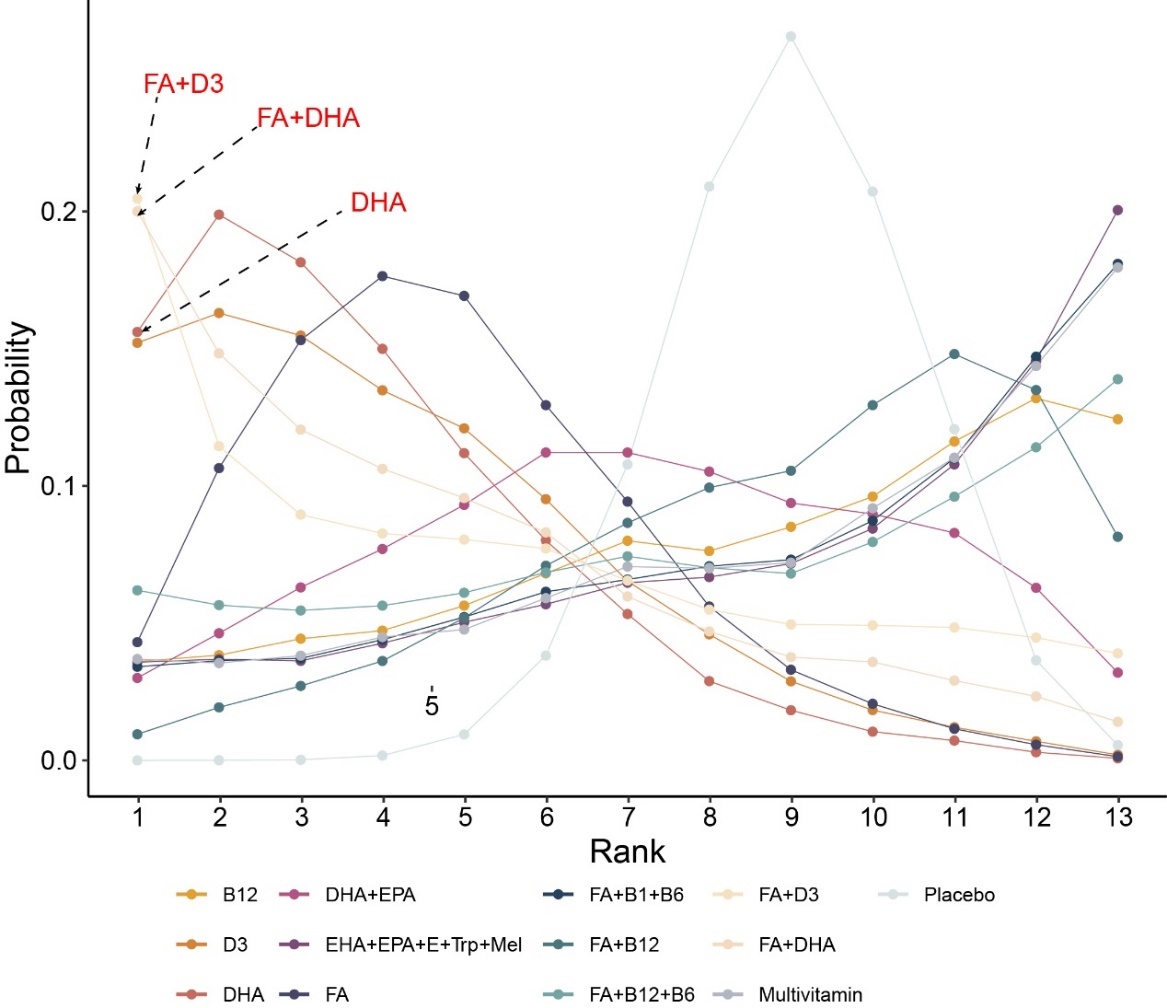


### eFigure 7A Attention (Subgroup analysis-Age): A, Network plot; B, funnel plot; C, forest plot.


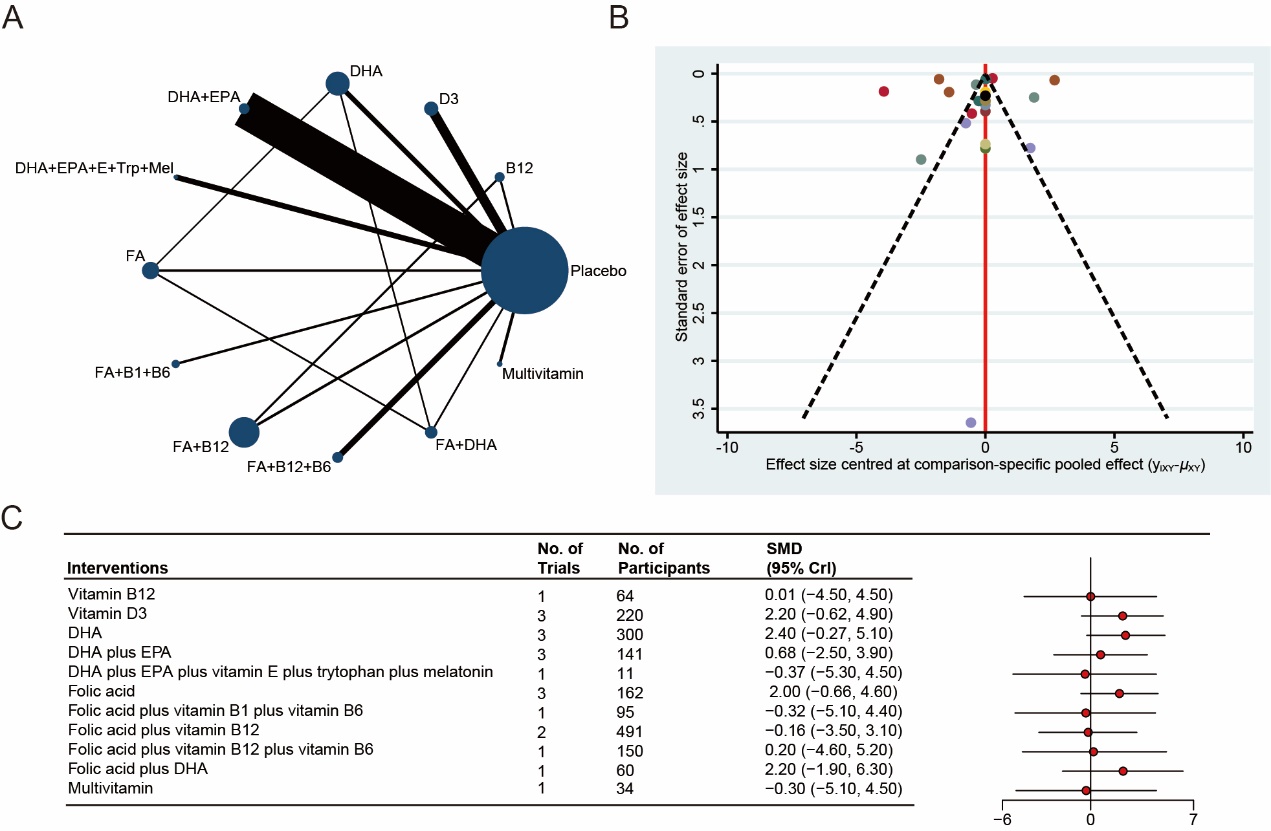


### eFigure 7B Attention (Subgroup analysis-Age): Node splitting analysis


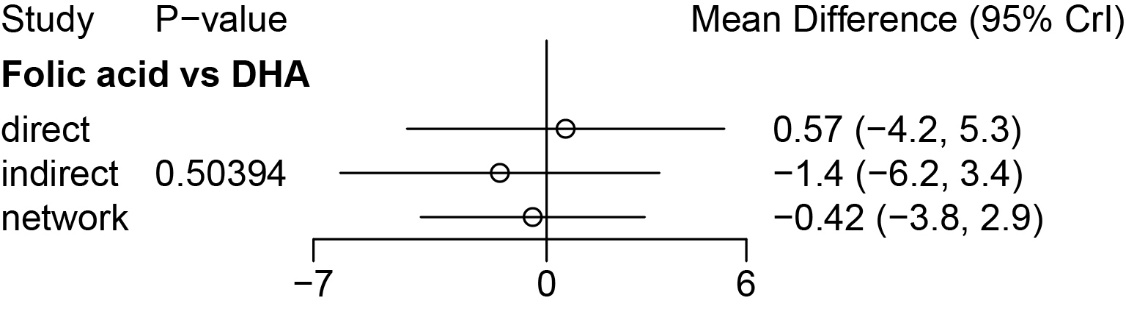


### eFigure 7C Attention (Subgroup analysis-Age): SUCRA plot


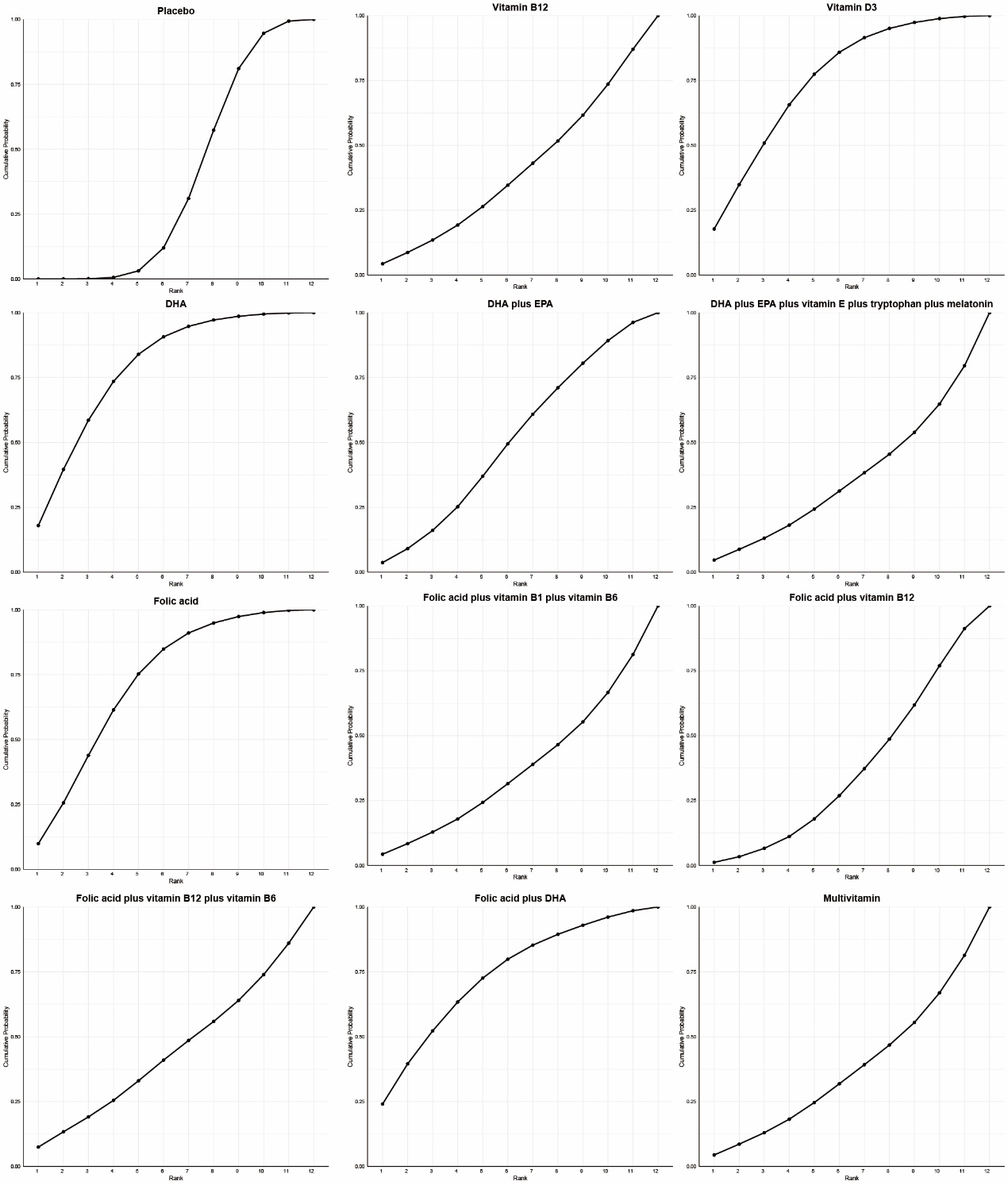


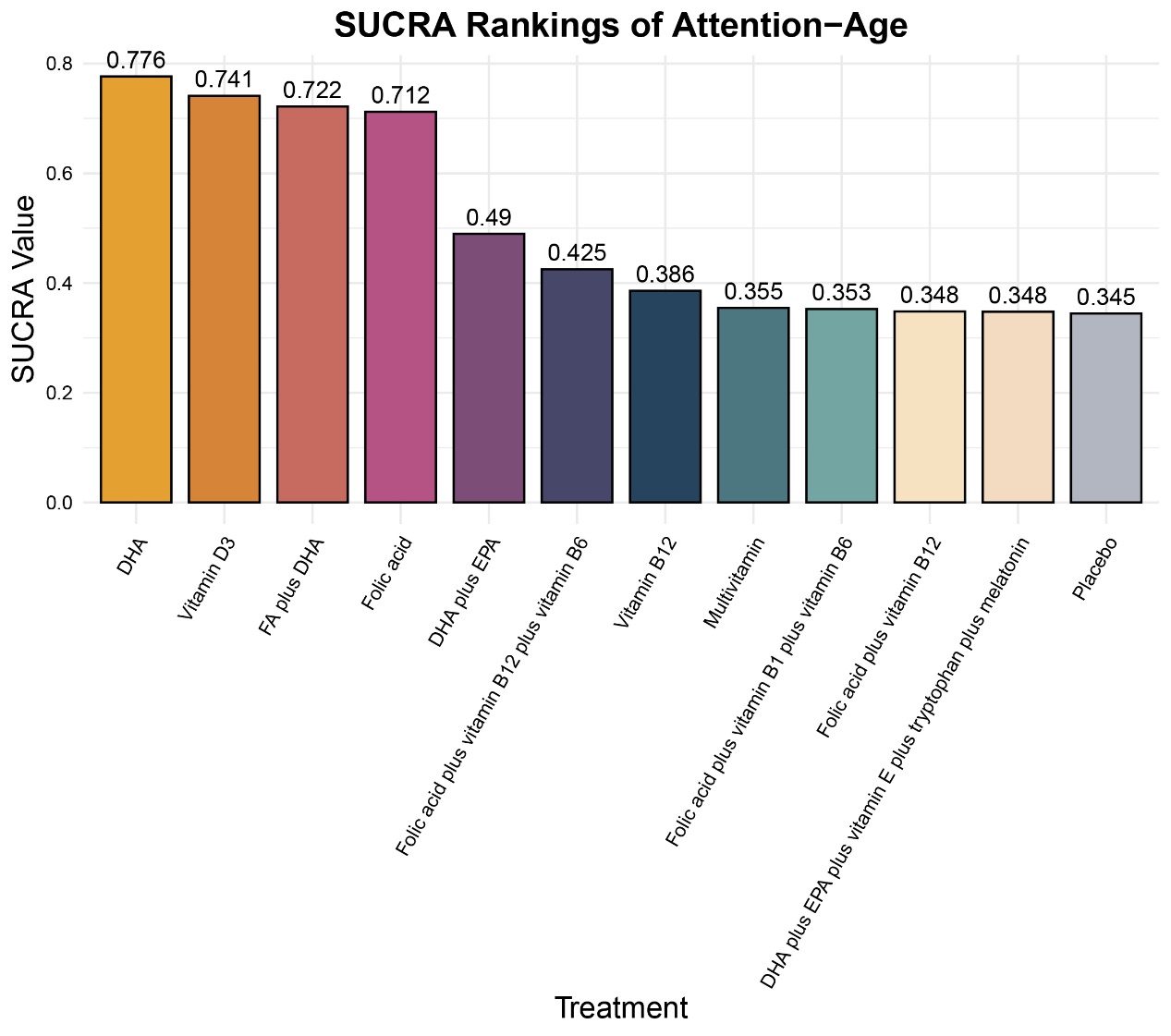


### eFigure 7D Attention (Subgroup analysis-Age): Ranking forest plot and ranking probability


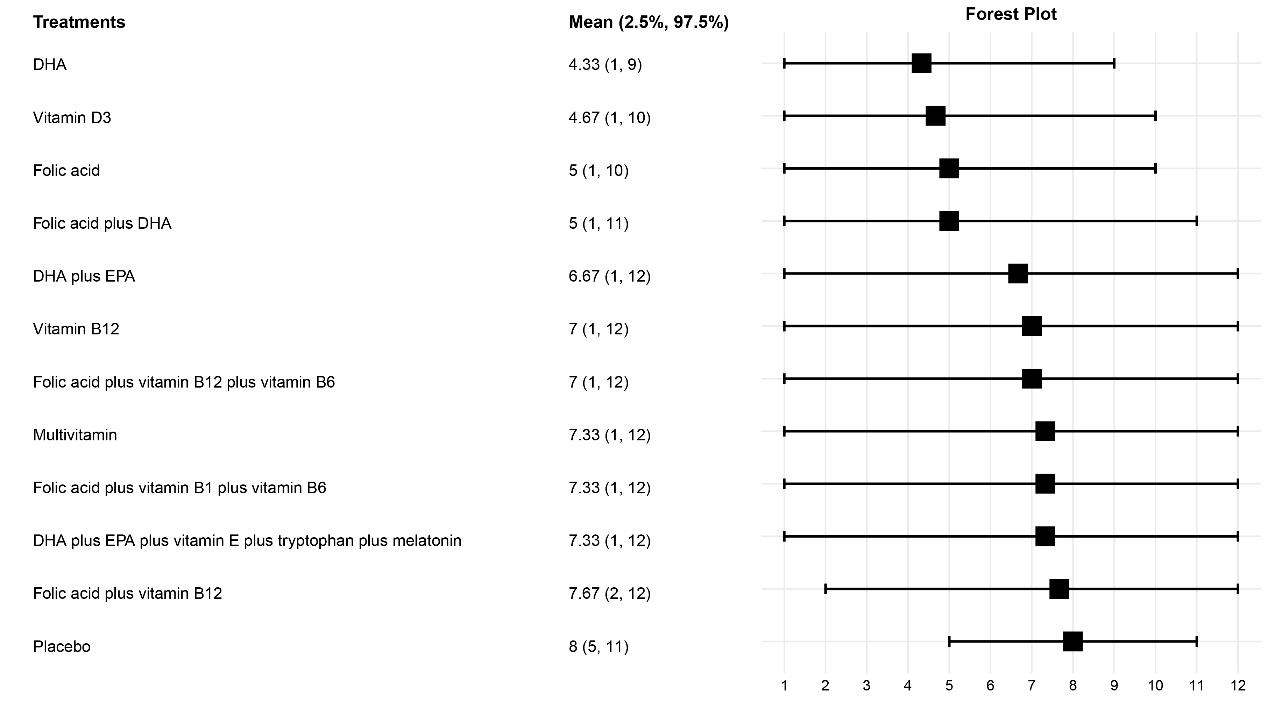


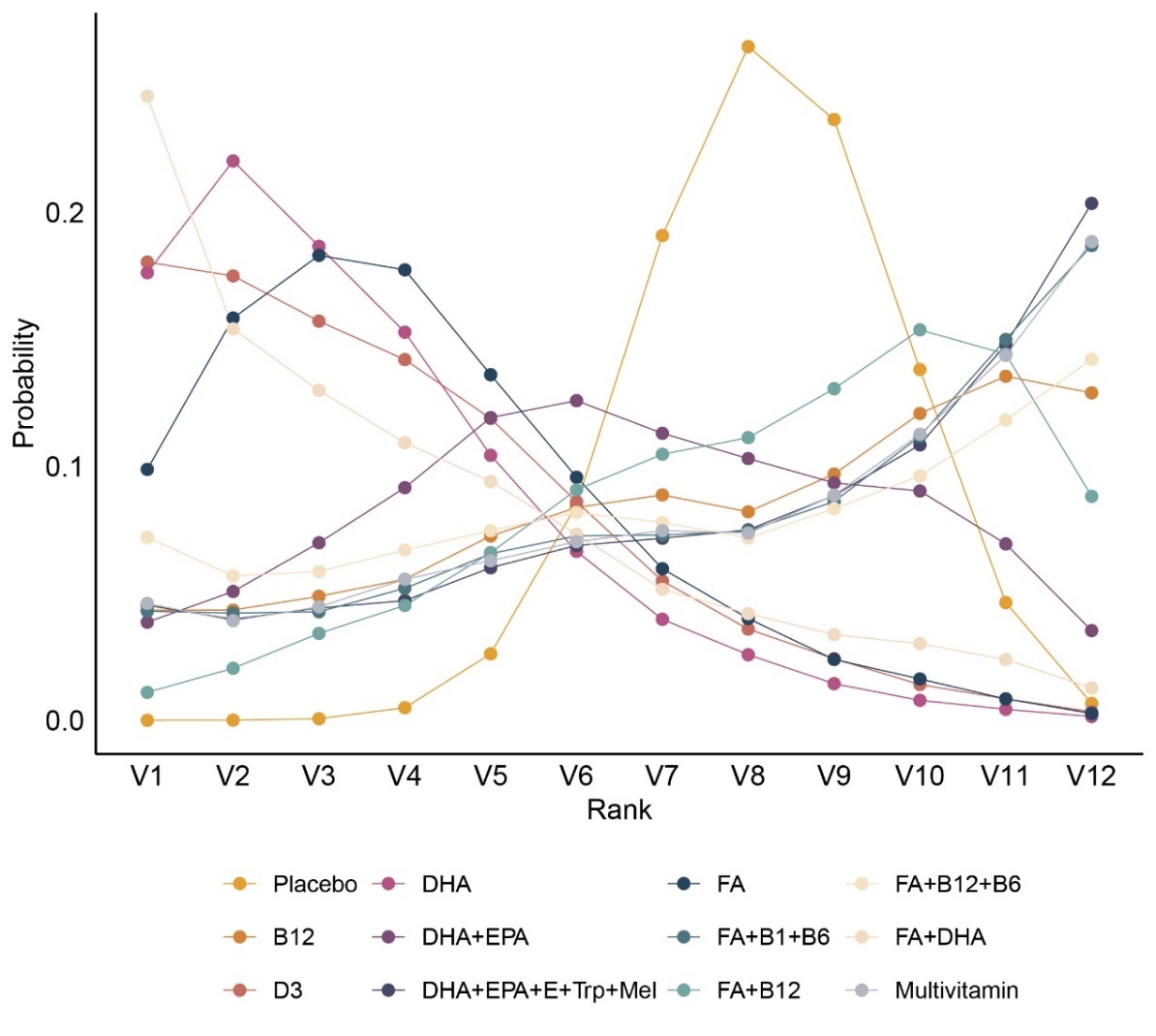


### eFigure 8A Attention (Subgroup analysis-MCI): A, Network plot; B, funnel plot; C, forest plot.


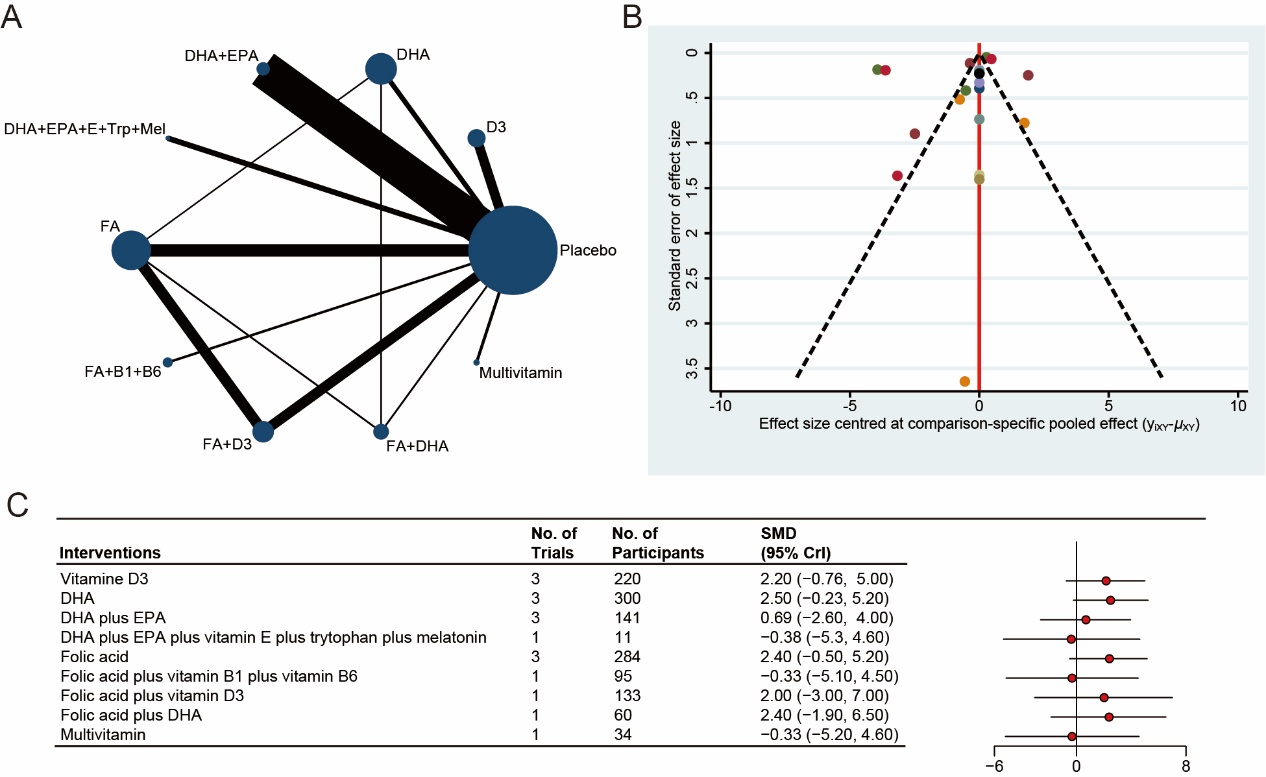


### eFigure 8B Attention (Subgroup analysis-MCI): Node splitting analysis


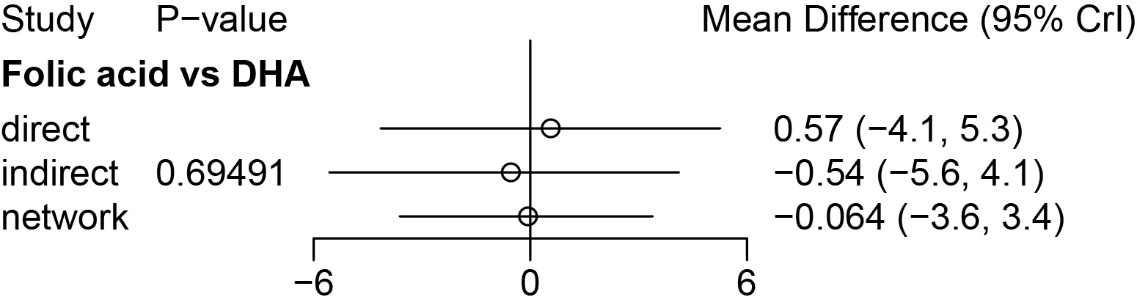


### eFigure 8C Attention (Subgroup analysis-MCI): SUCRA plot


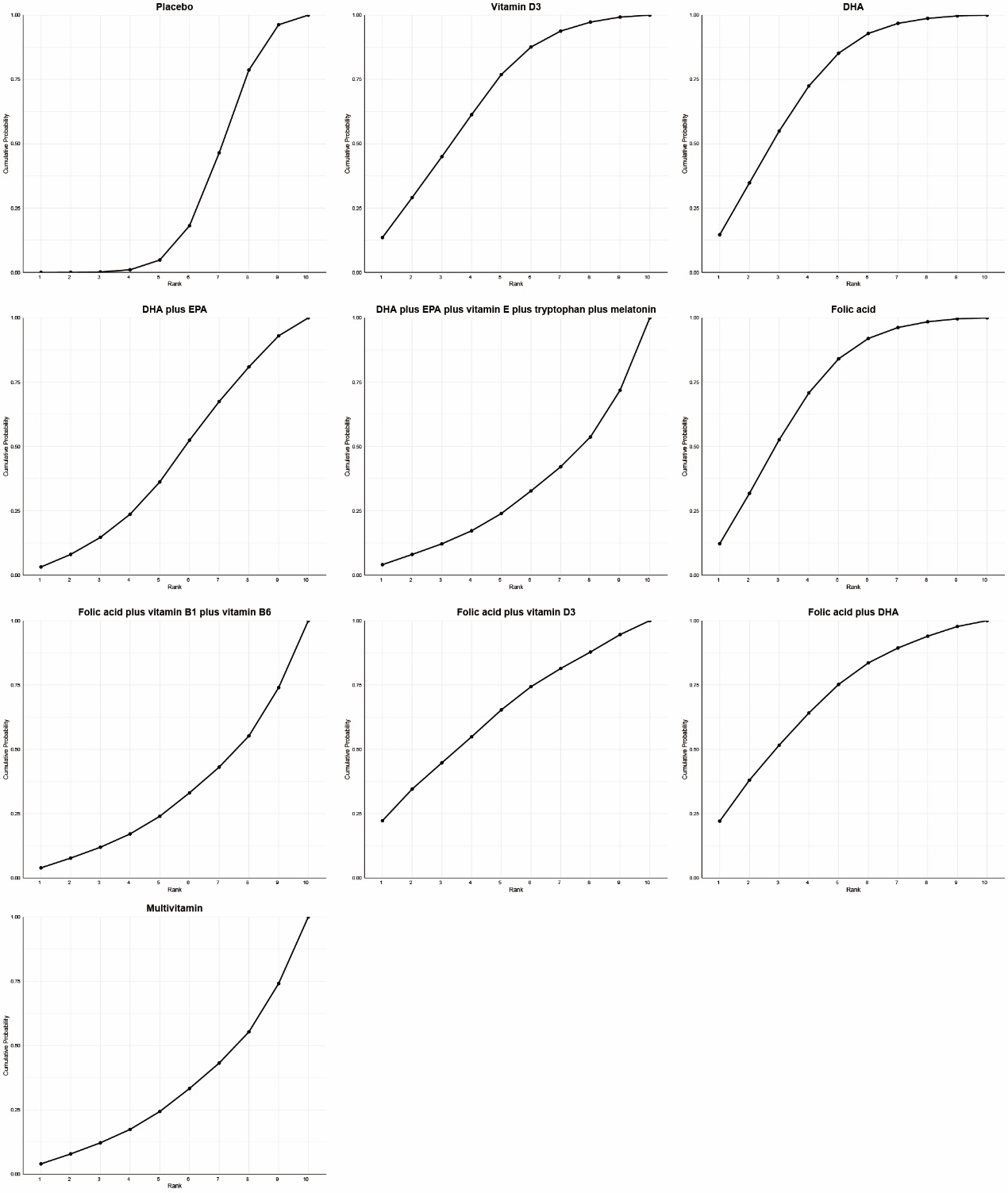


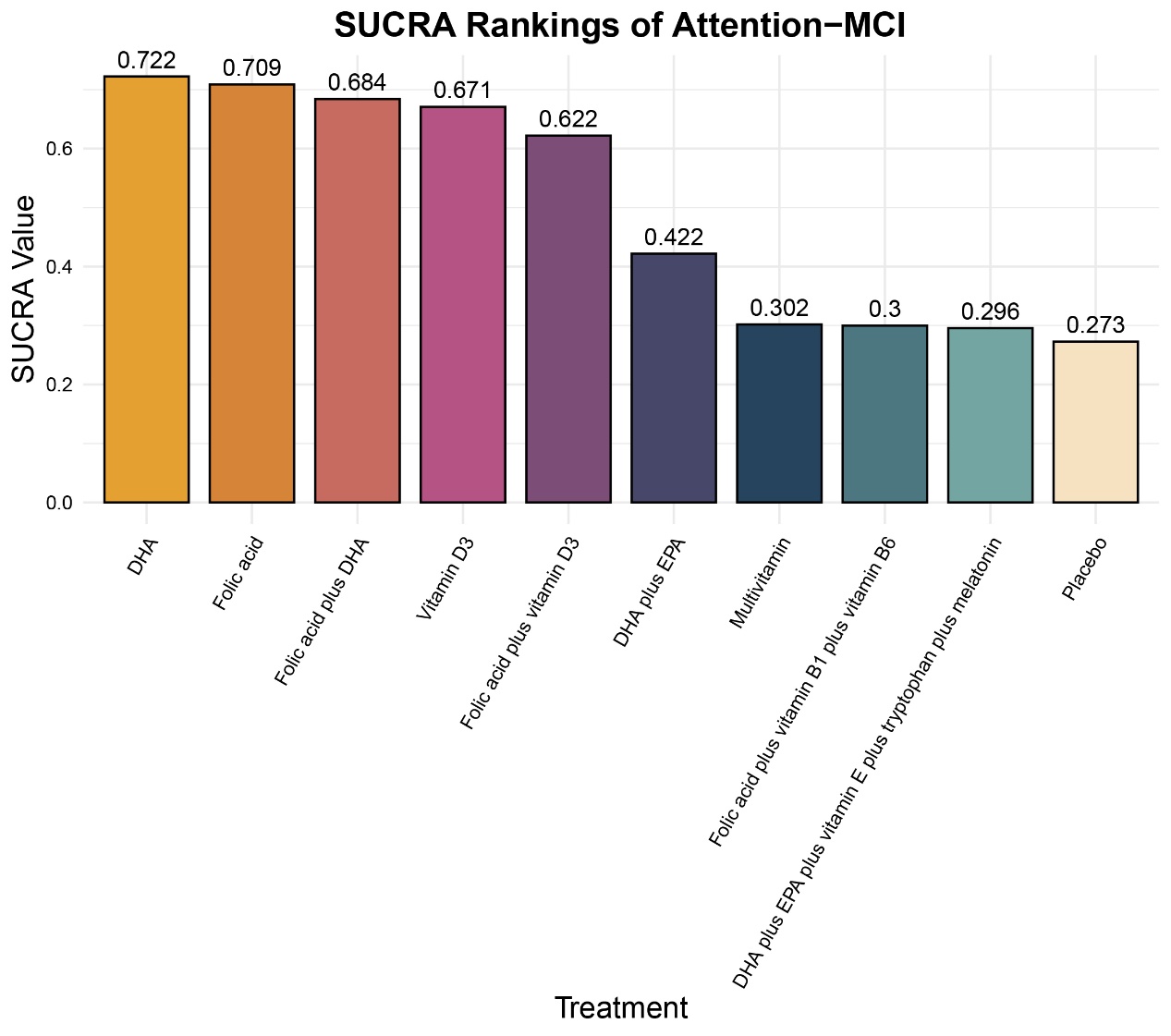


### eFigure 8D Attention (Subgroup analysis-MCI): Ranking forest plot and ranking probability


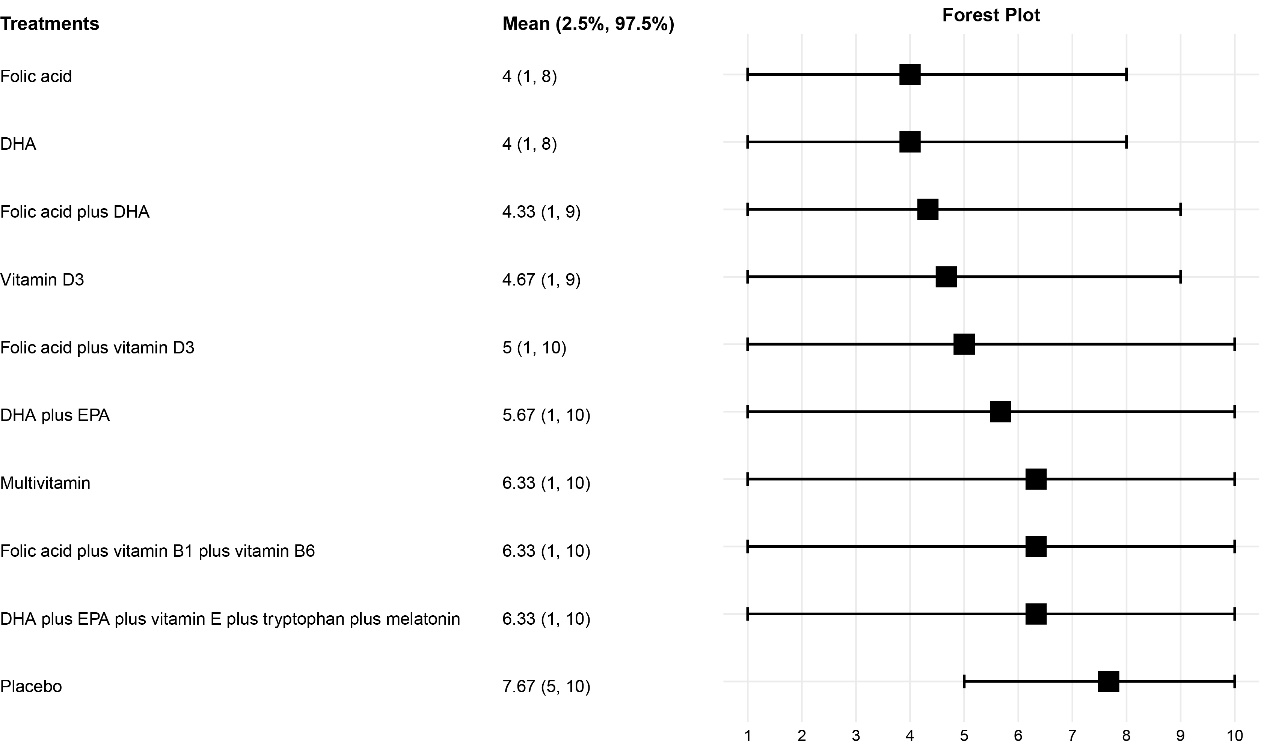


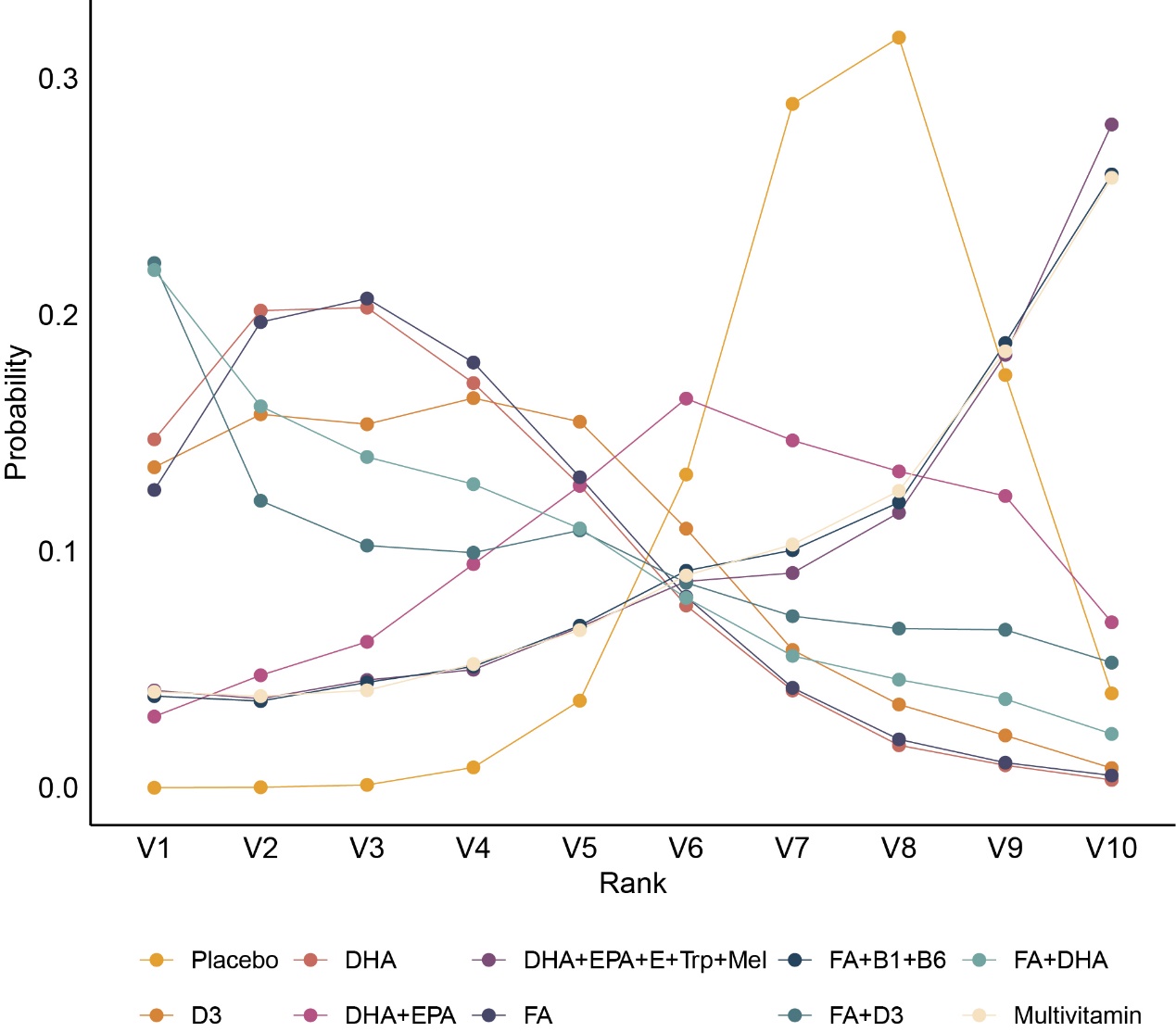


### eFigure 9A Attention (Subgroup analysis-Sample size over 100): A, Network plot; B, funnel plot; C, forest plot.


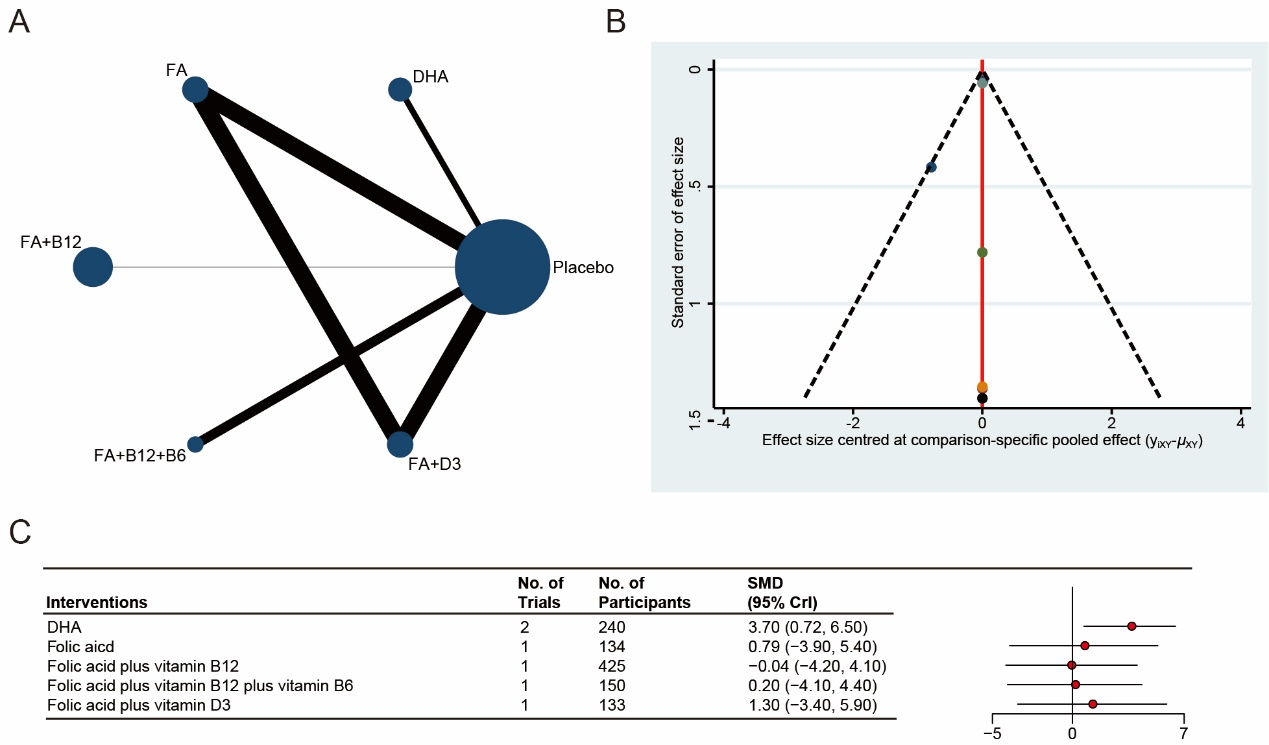


### eFigure 9B Attention (Subgroup analysis-Sample size over 100): SUCRA plot


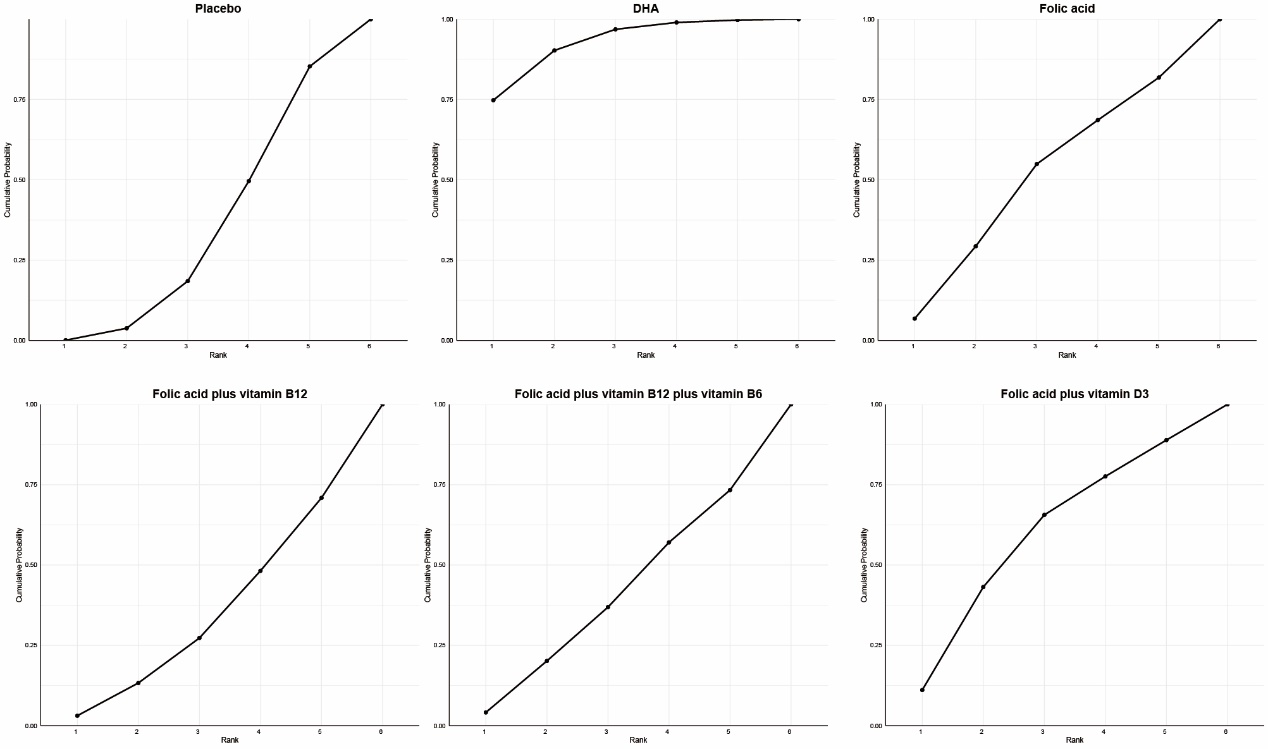


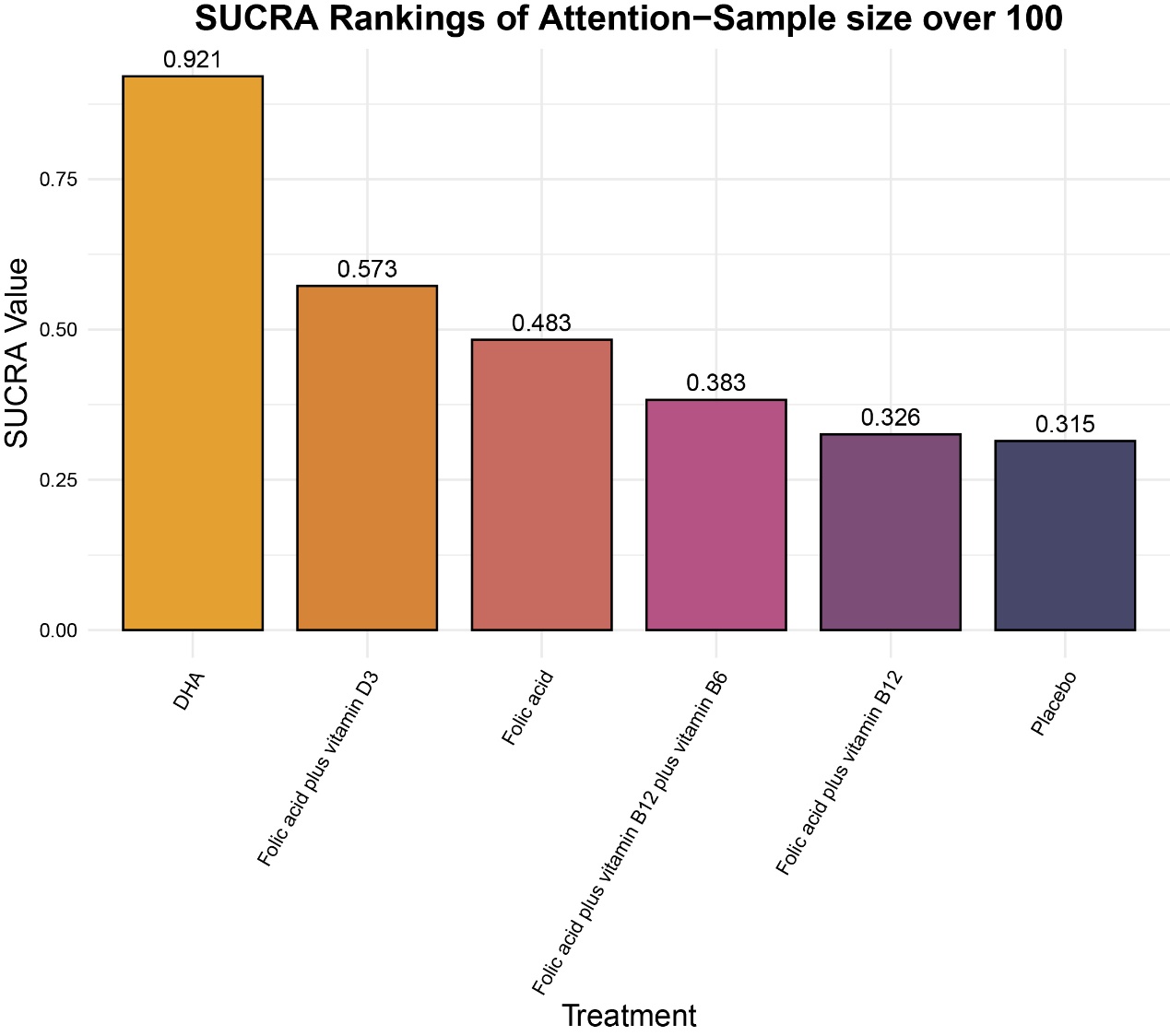


### eFigure 9C Attention (Subgroup analysis-Sample size over 100): Ranking forest plot and ranking probability


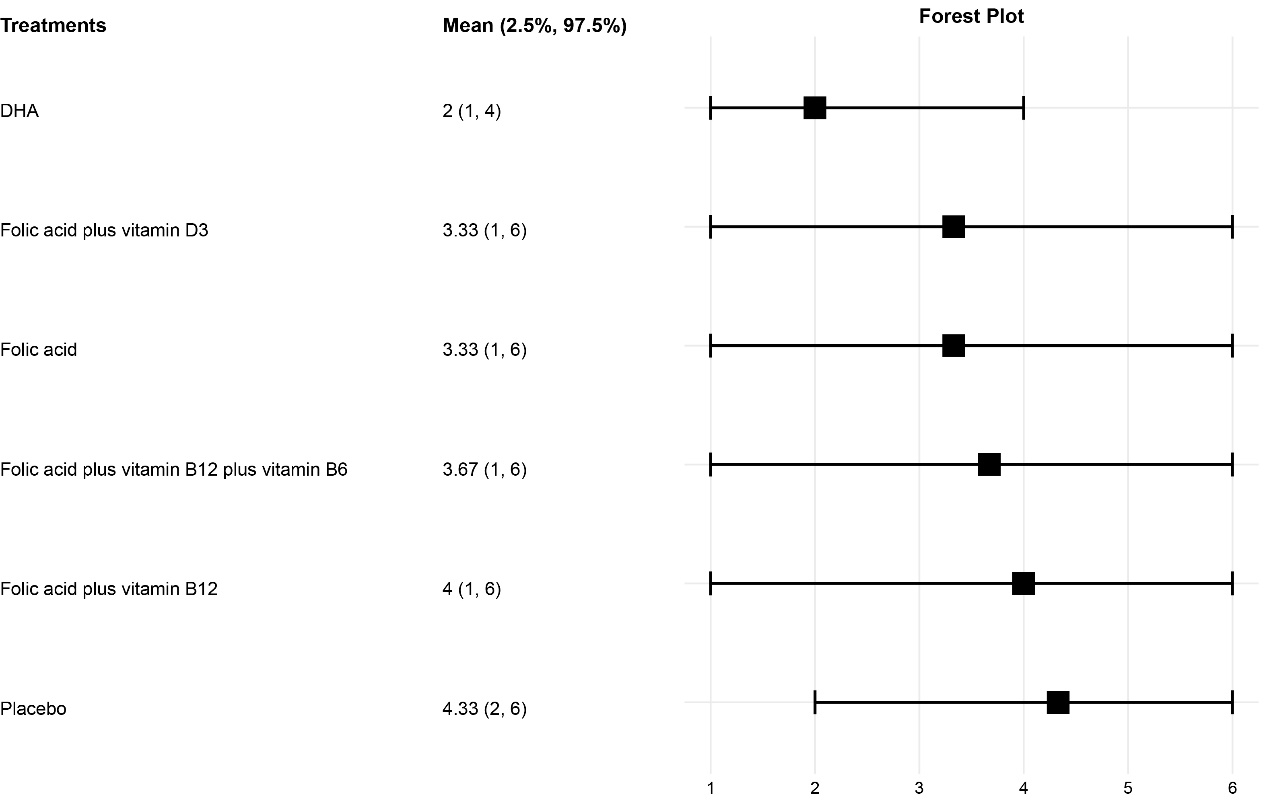


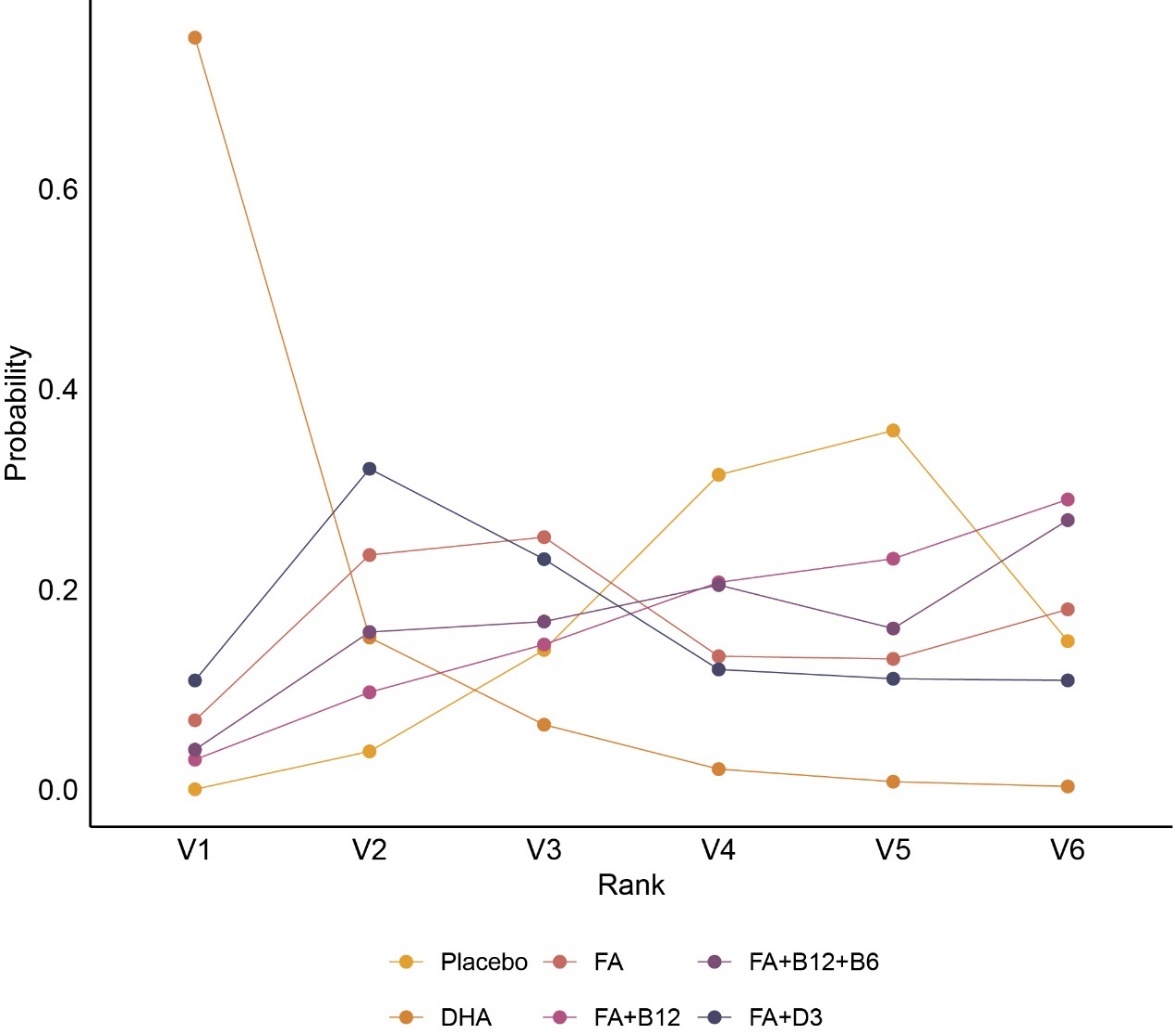


## Part Ⅲ Cognitive function assessment: Executive function

### eFigure 10A Executive function (All): Node splitting analysis


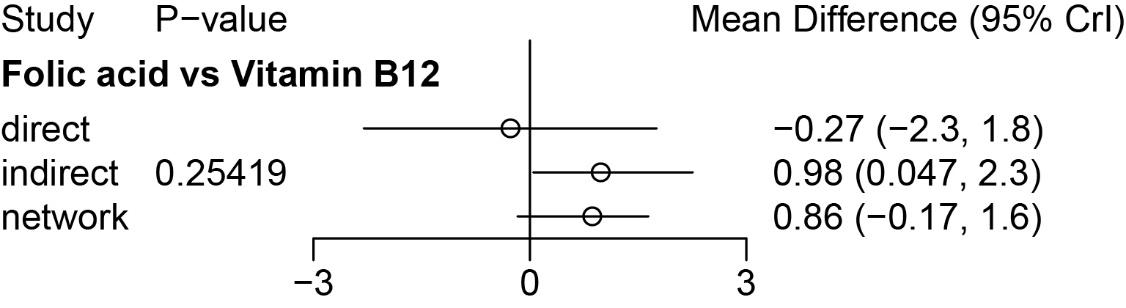


### eFigure 10B Executive function (All): Bland Altman analysis


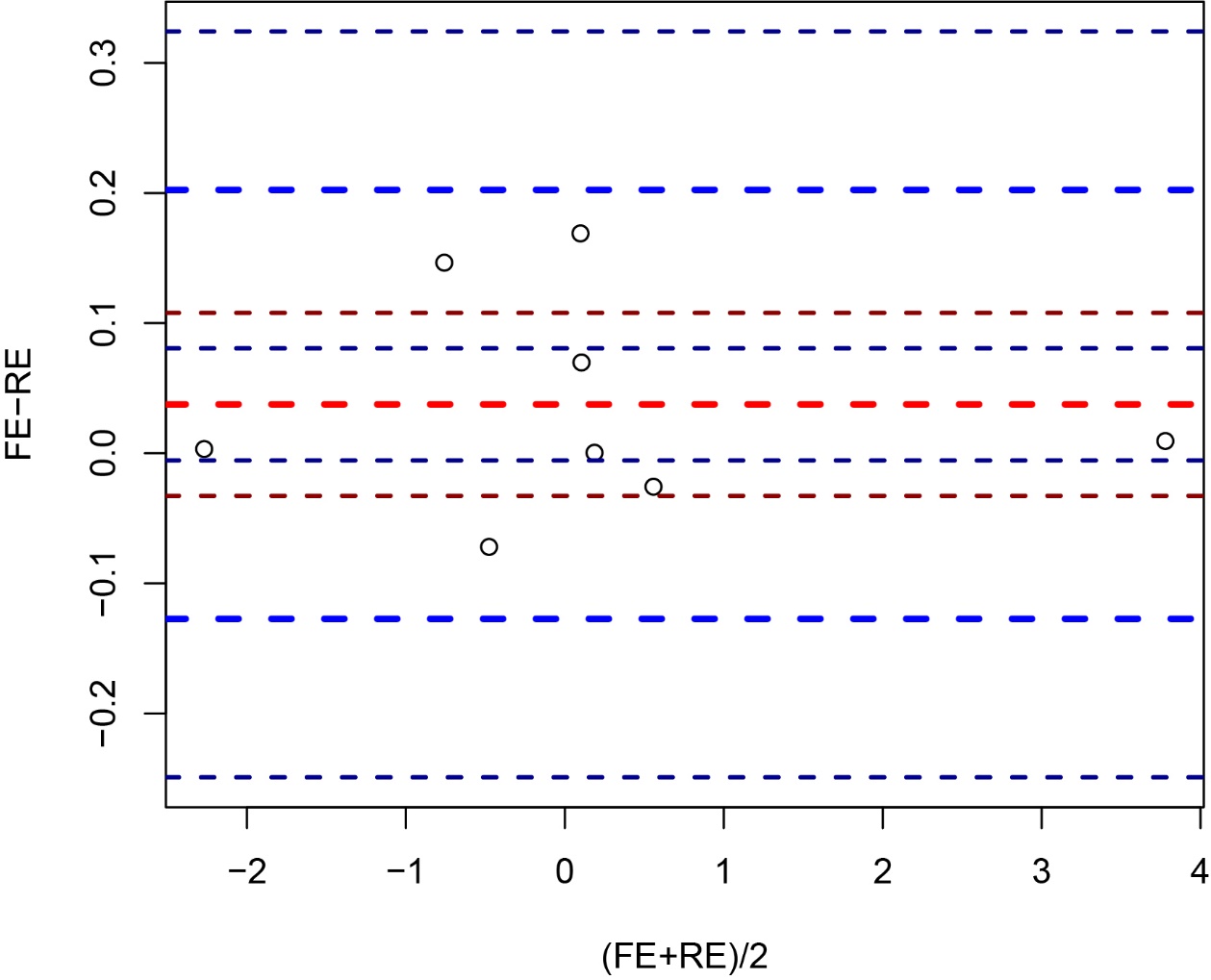


### eFigure 10C Executive function (All): SUCRA plot


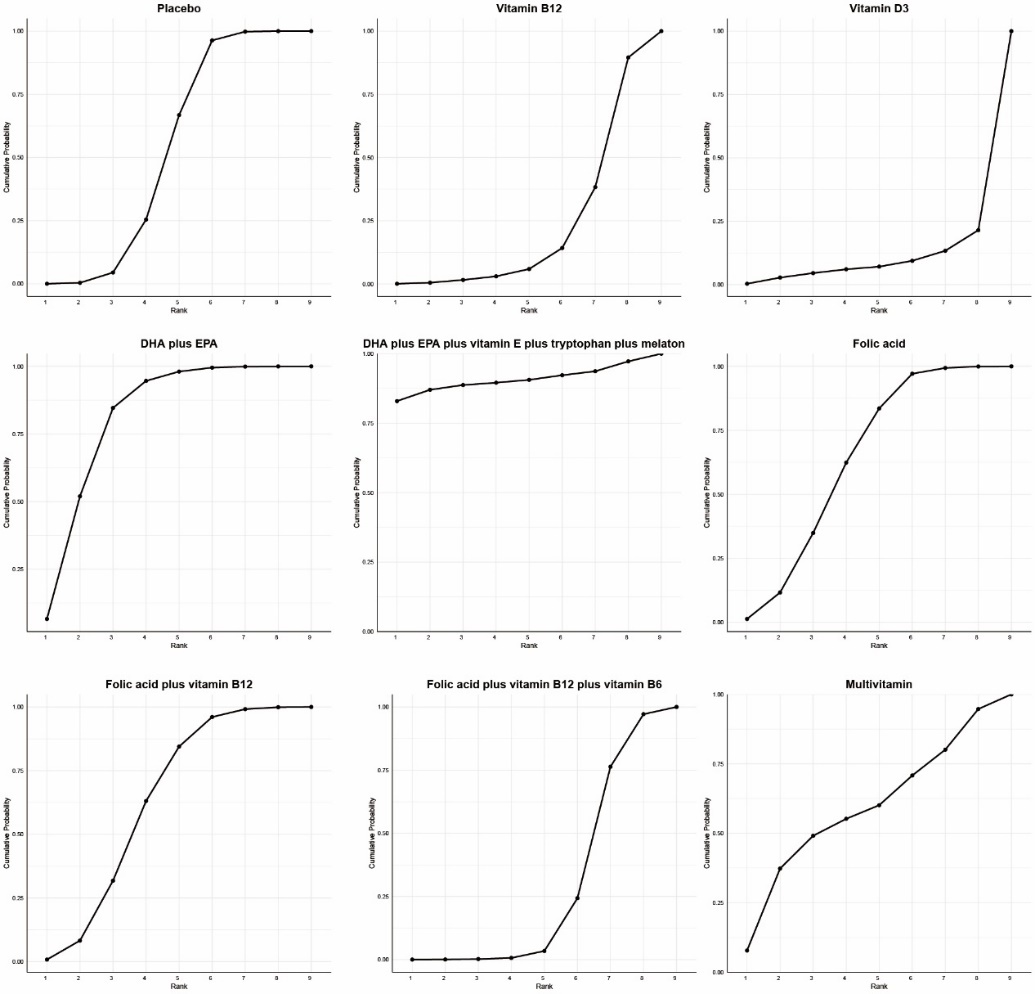


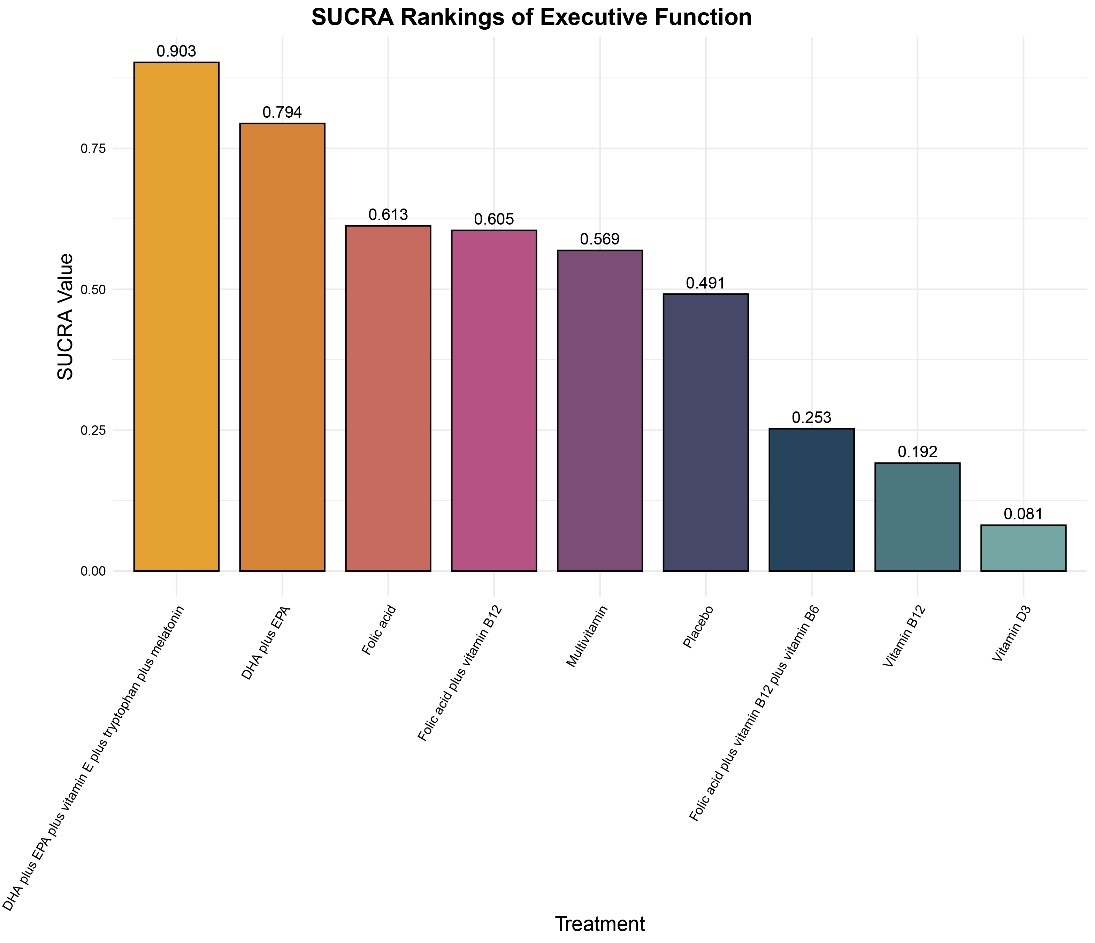


### eFigure 10D Executive function (All): Ranking forest plot and ranking probability


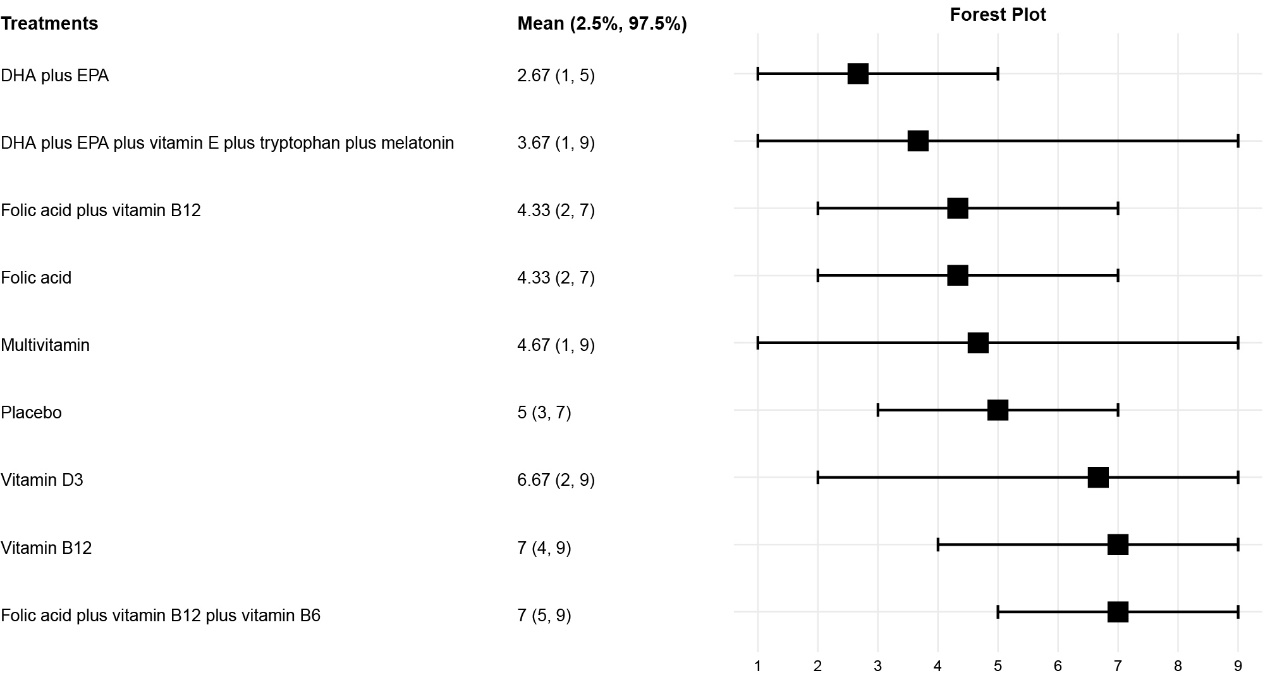


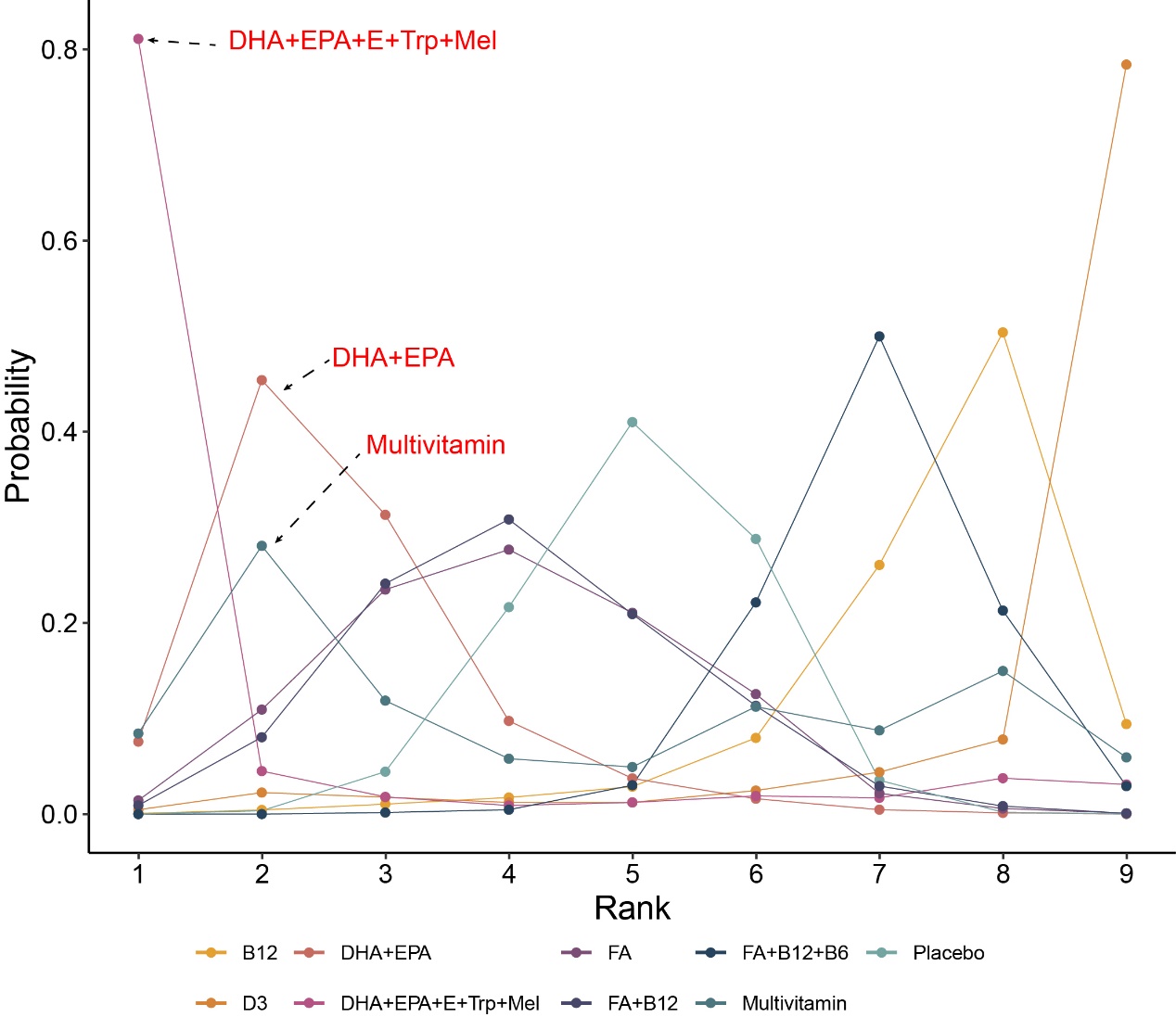


### eFigure 11A Executive function (Subgroup analysis-Age): A, Network plot; B, funnel plot; C, forest plot.


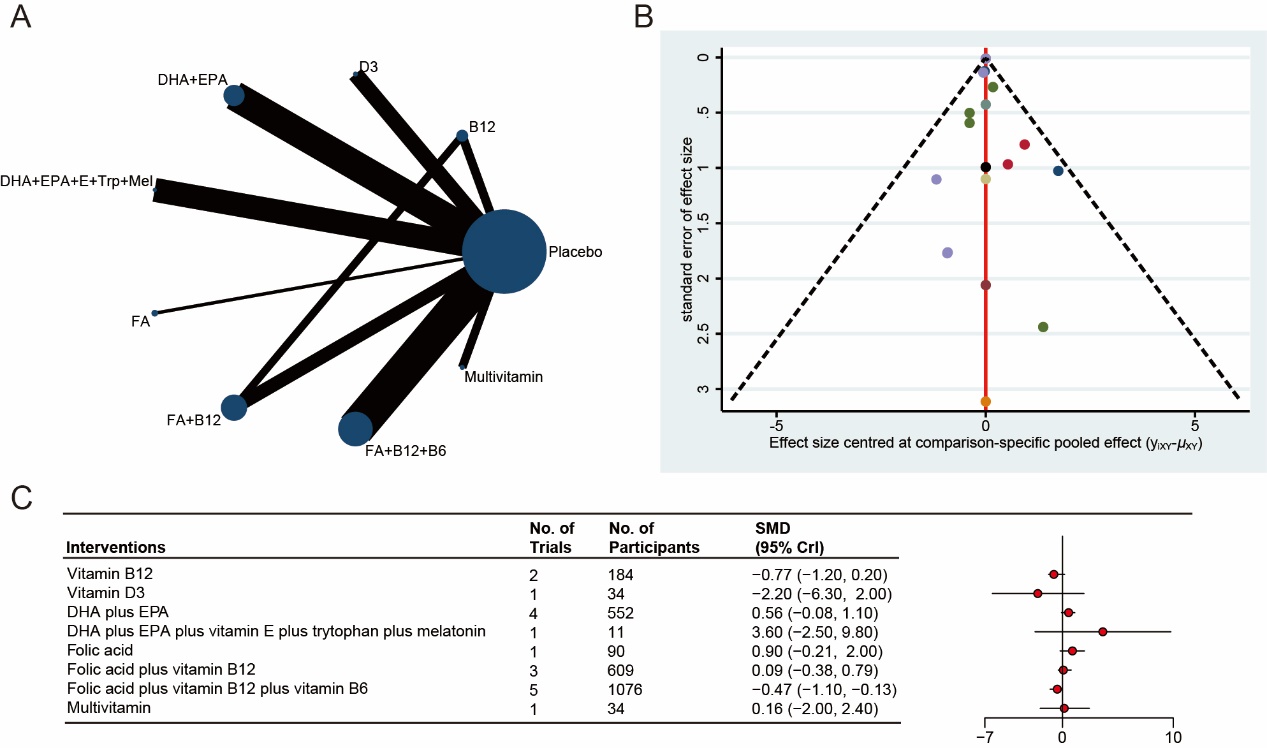


### eFigure 11B Executive function (Subgroup analysis-Age): Node splitting analysis


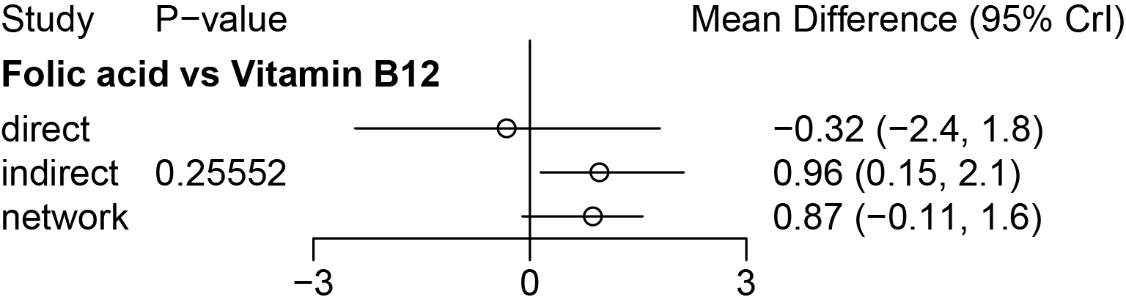


### eFigure 11C Executive function (Subgroup analysis-Age): SUCRA plot


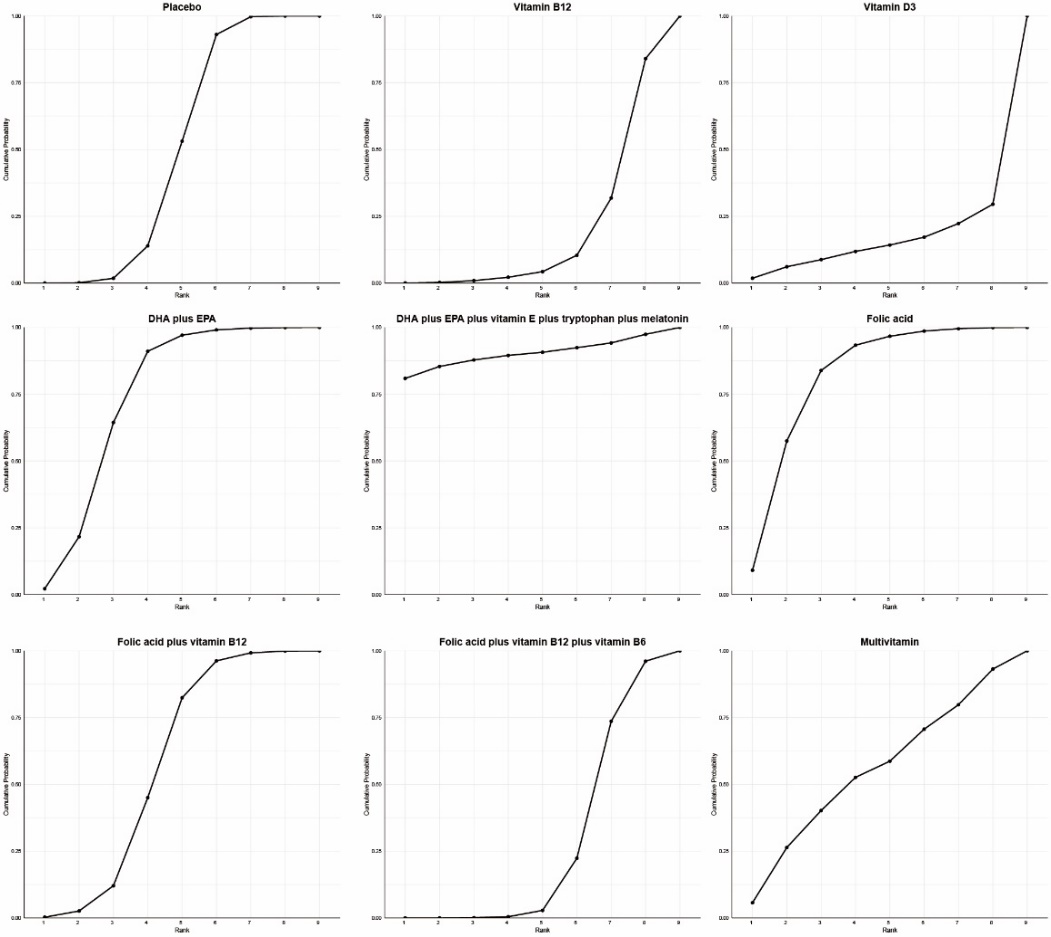


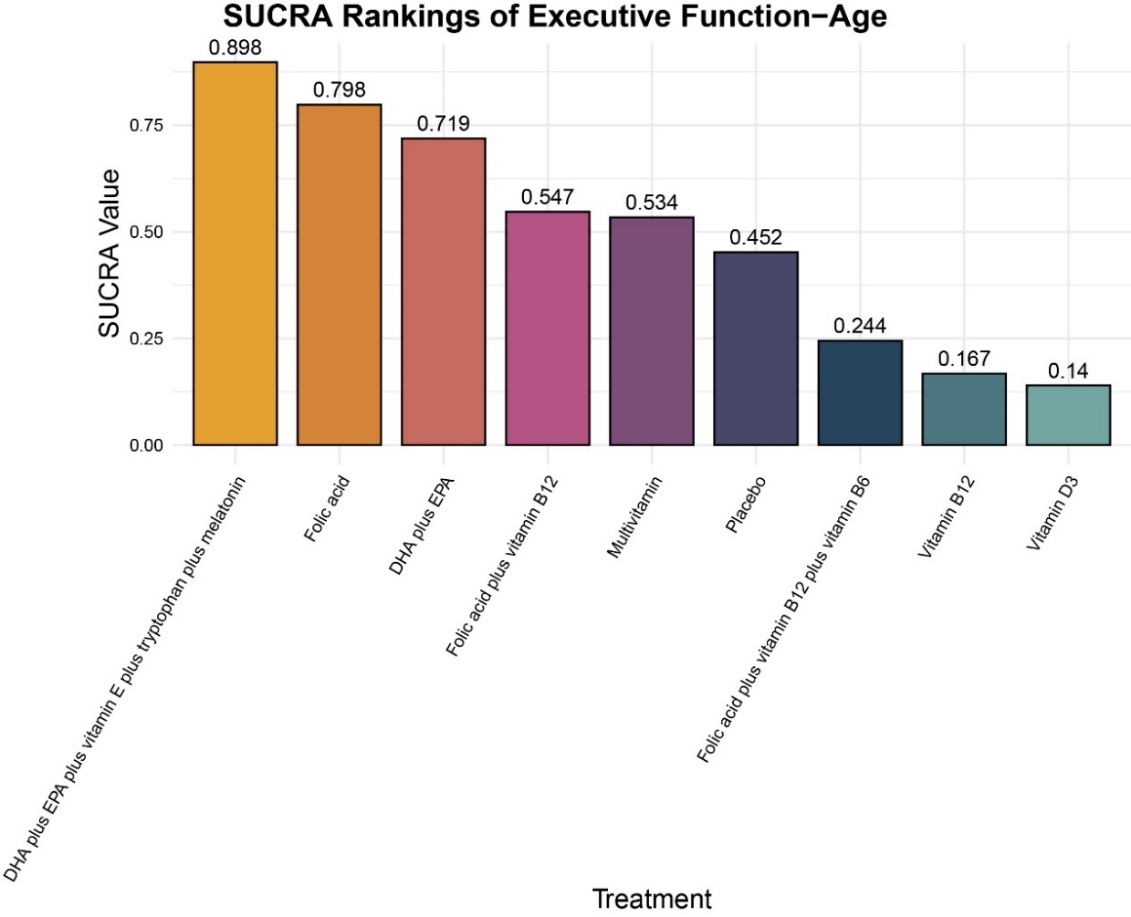


### eFigure 11D Executive function (Subgroup analysis-Age): Ranking forest plot and ranking probability


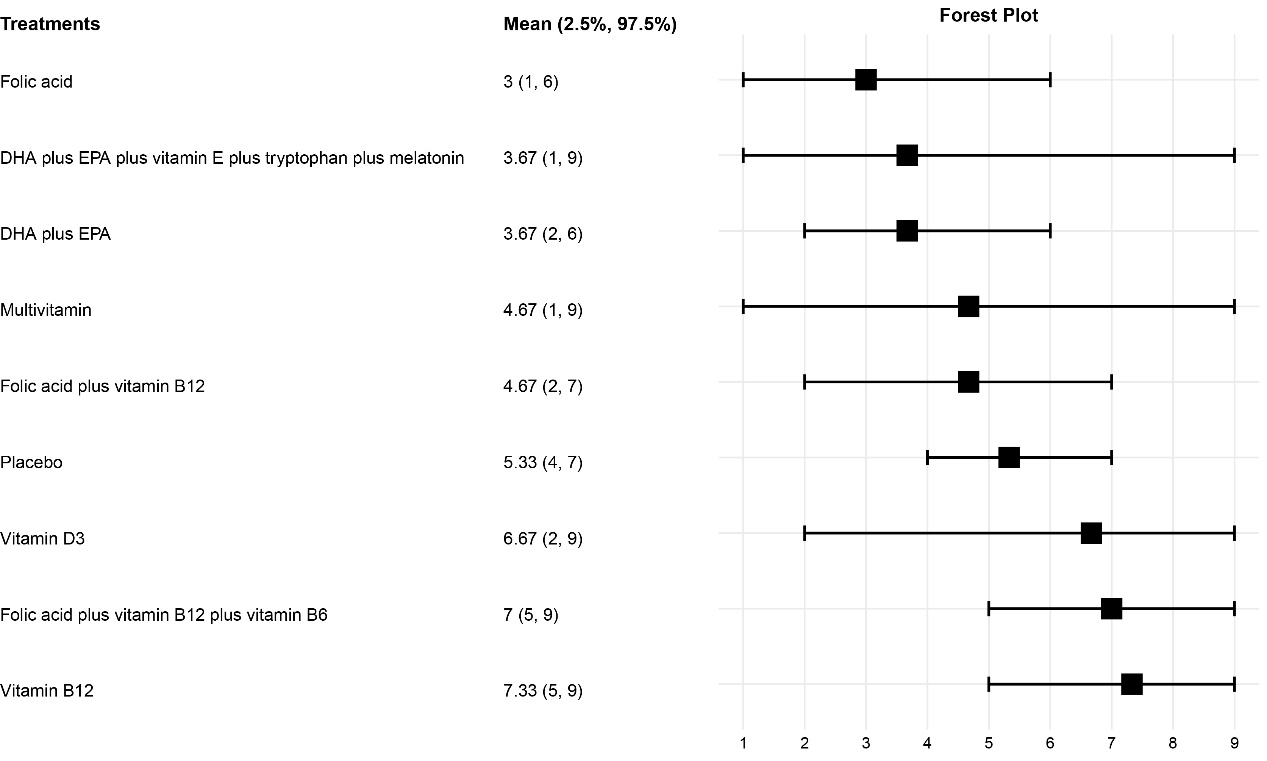


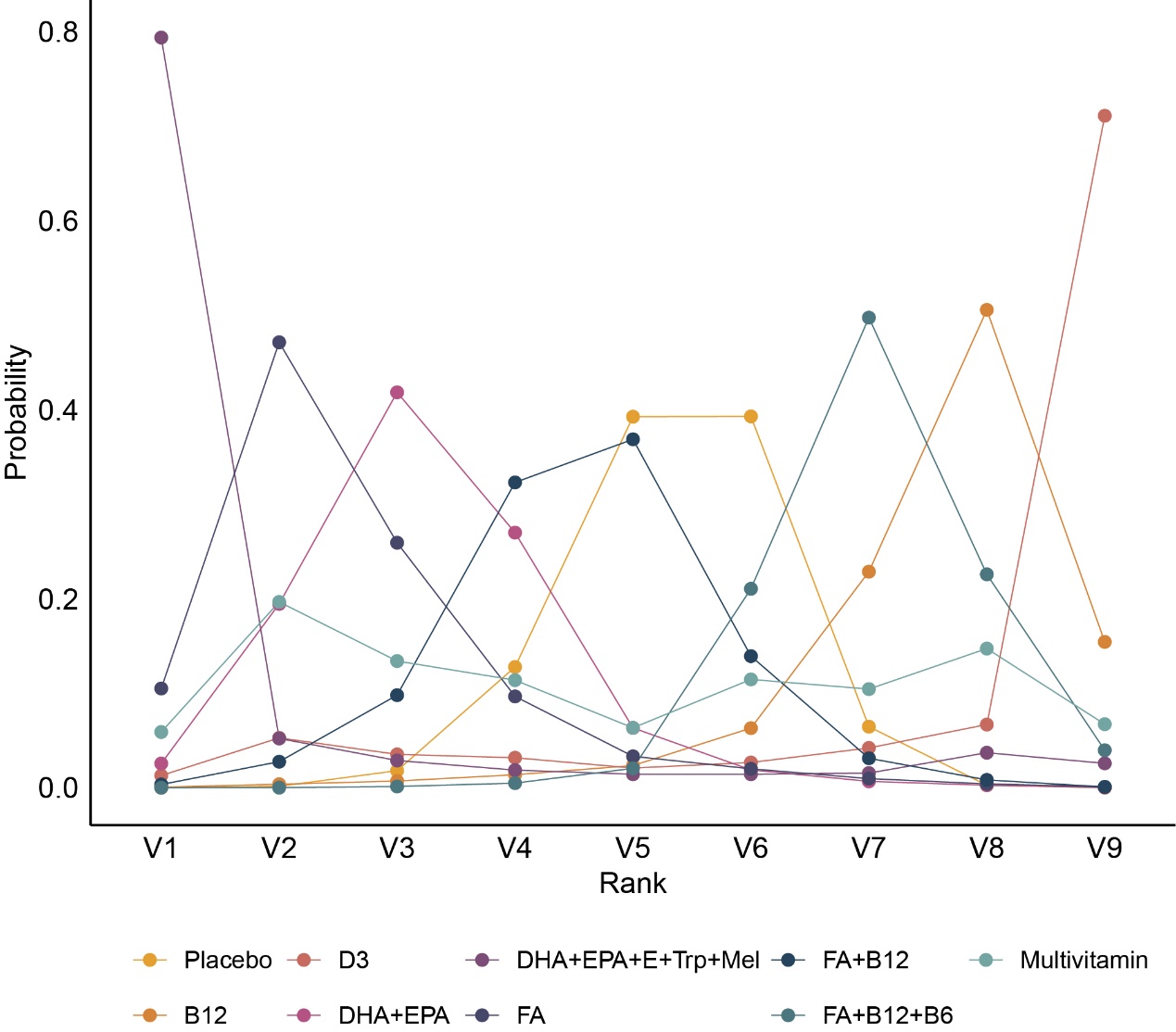


### eFigure 12A Executive function (Subgroup analysis-MCI): A, Network plot; B, funnel plot; C, forest plot.


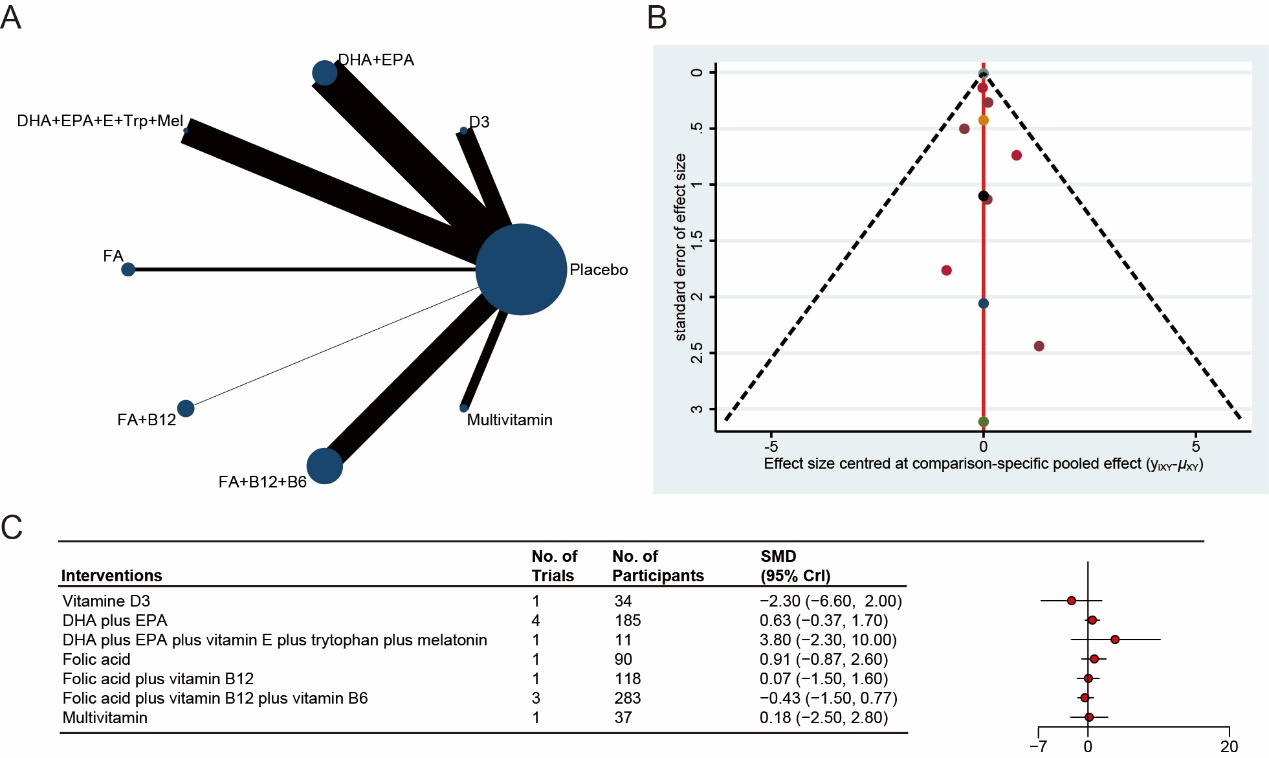


### eFigure 12B Executive function (Subgroup analysis-MCI): SUCRA plot


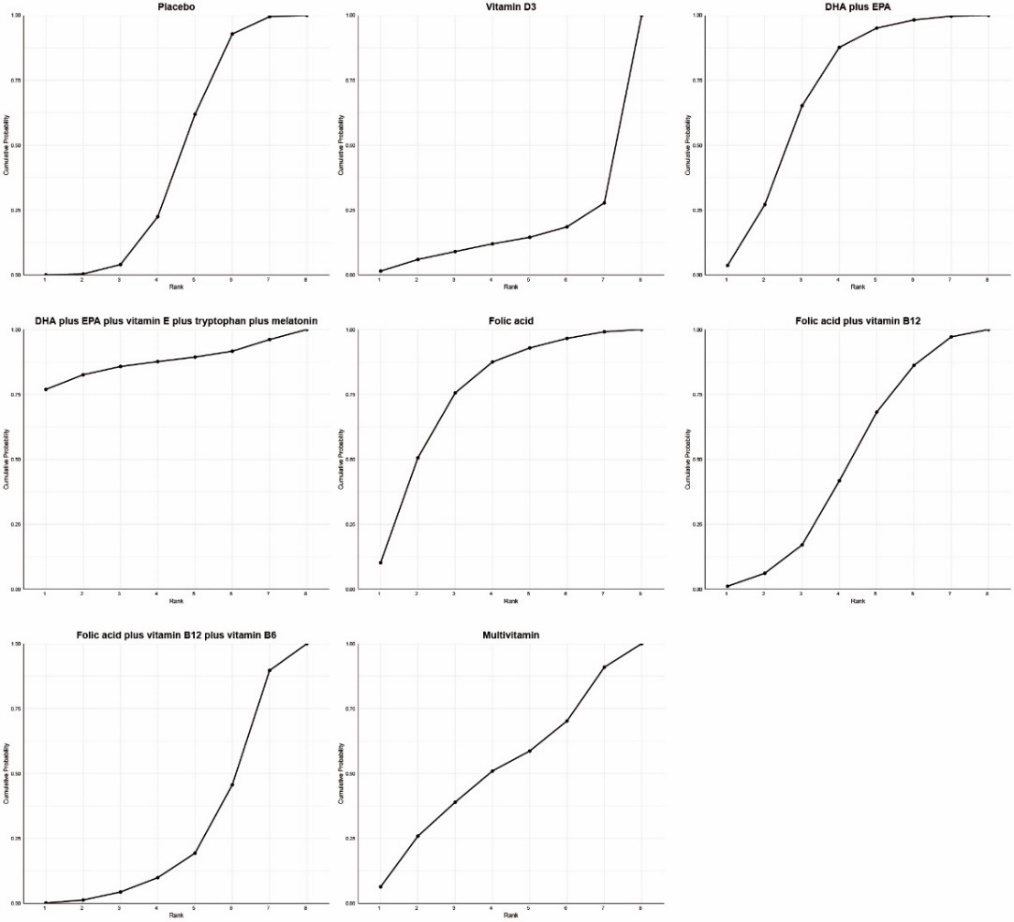


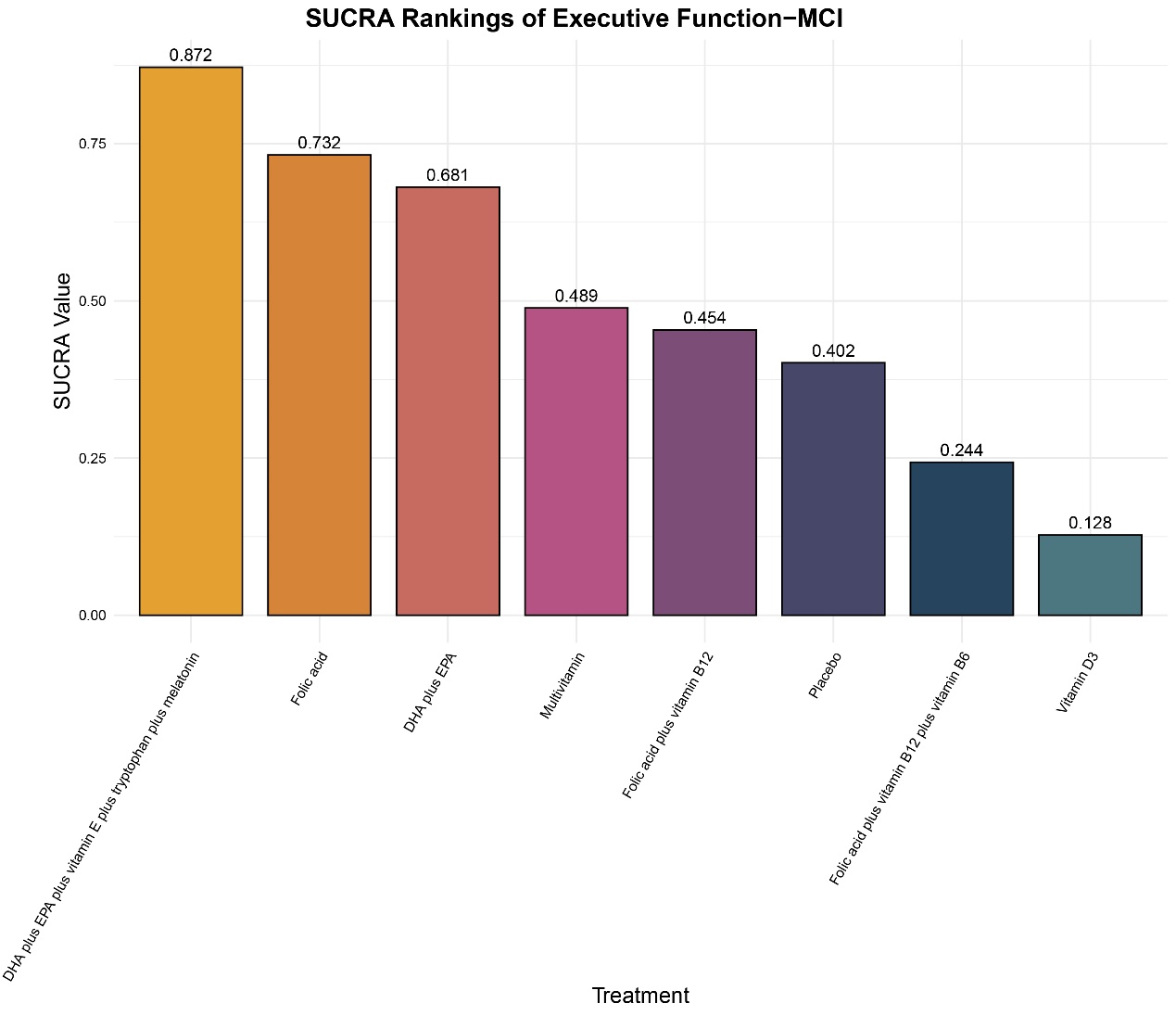


### eFigure 12C Executive function (Subgroup analysis-MCI): Ranking forest plot and ranking probability


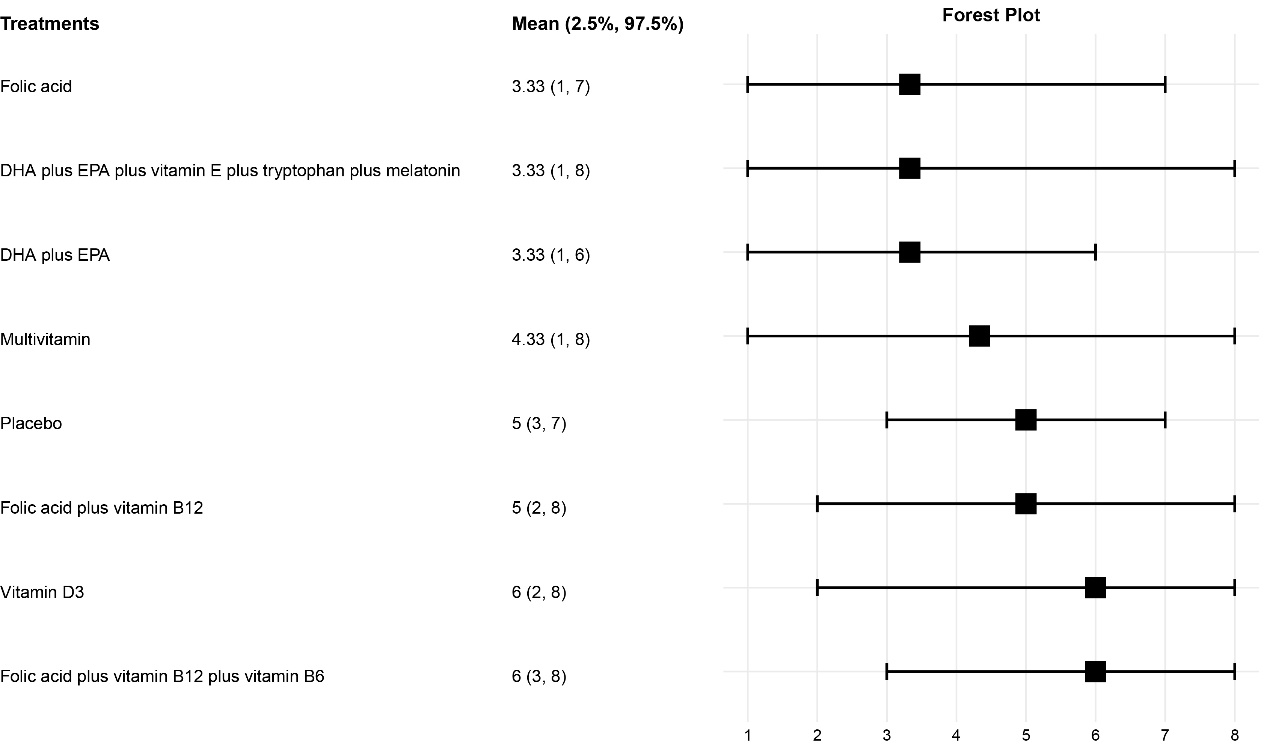


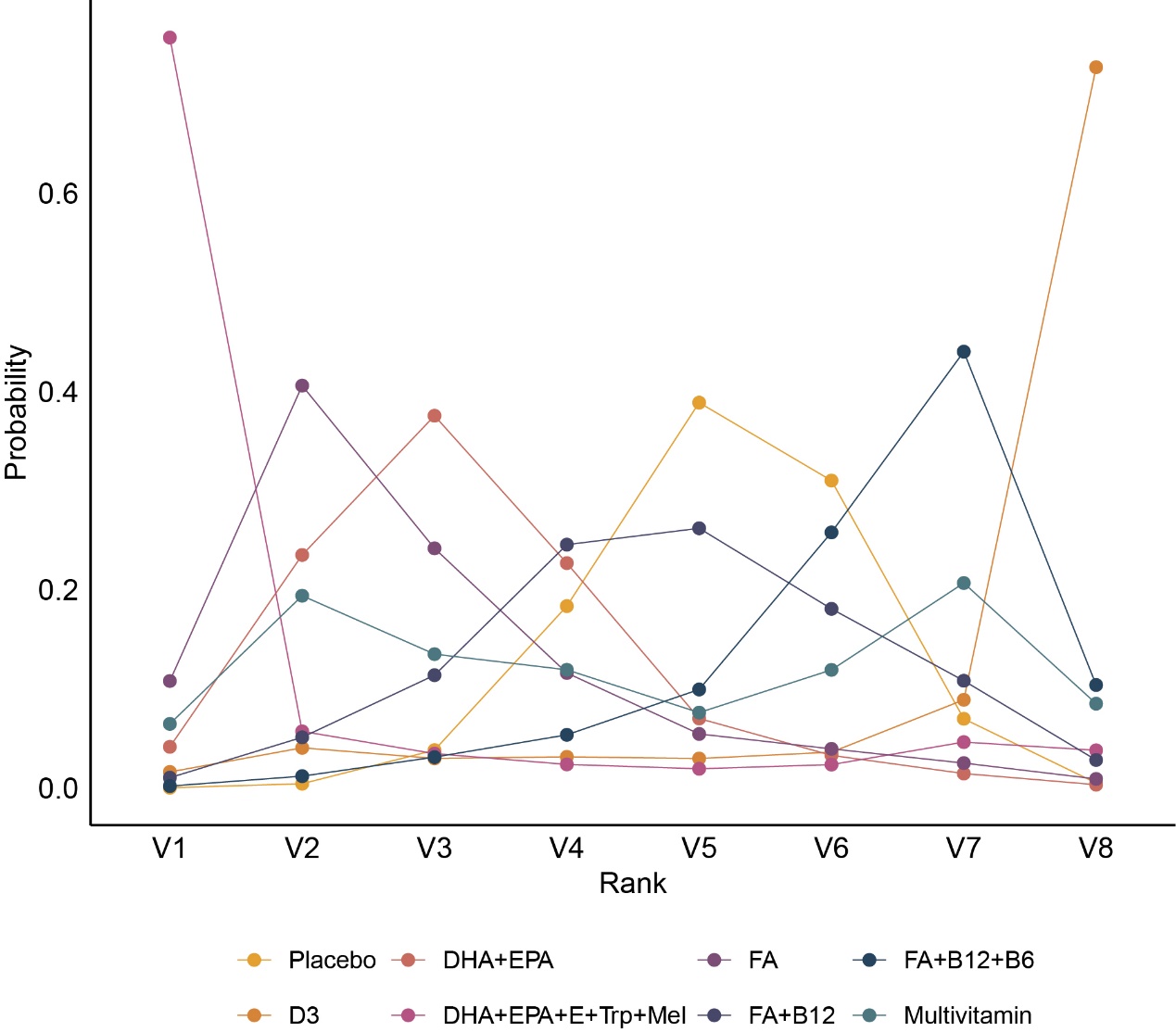


### eFigure 13A Executive function (Subgroup analysis-Sample size over 100): A, Network plot; B, funnel plot; C, forest plot.


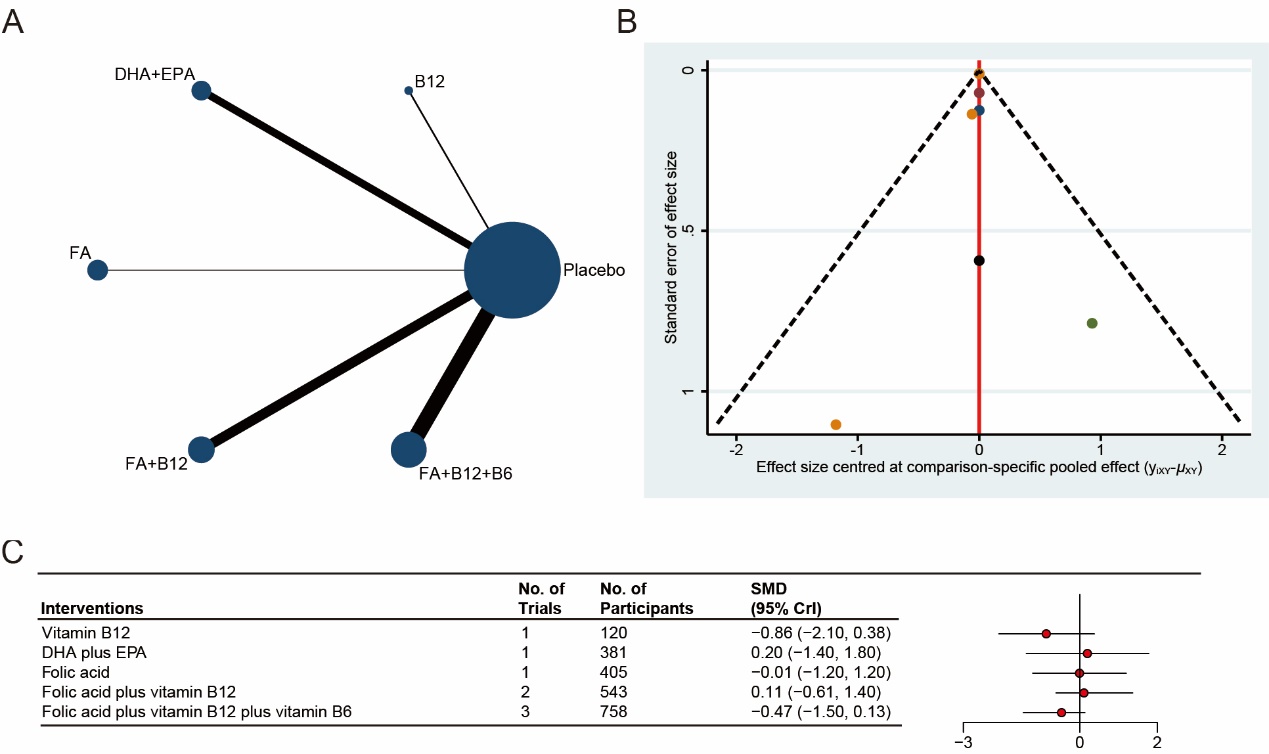


### eFigure 13B Executive function (Subgroup analysis-Sample size over 100): SUCRA plot


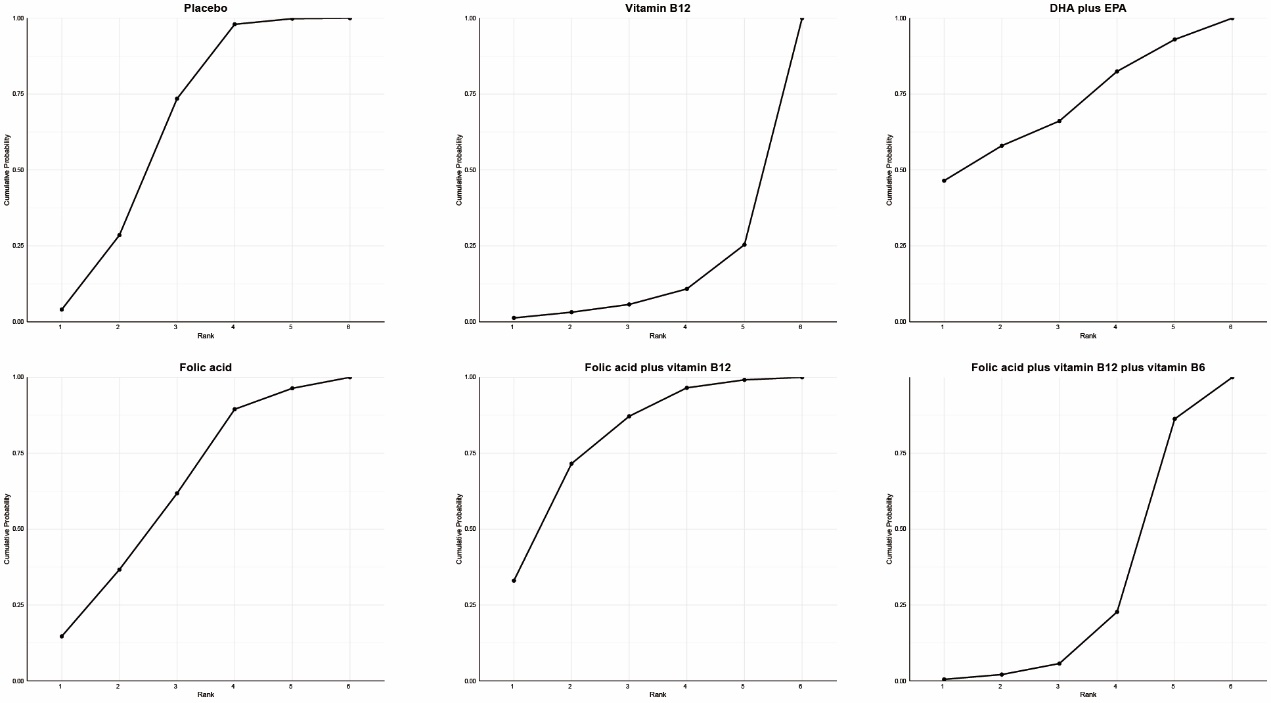


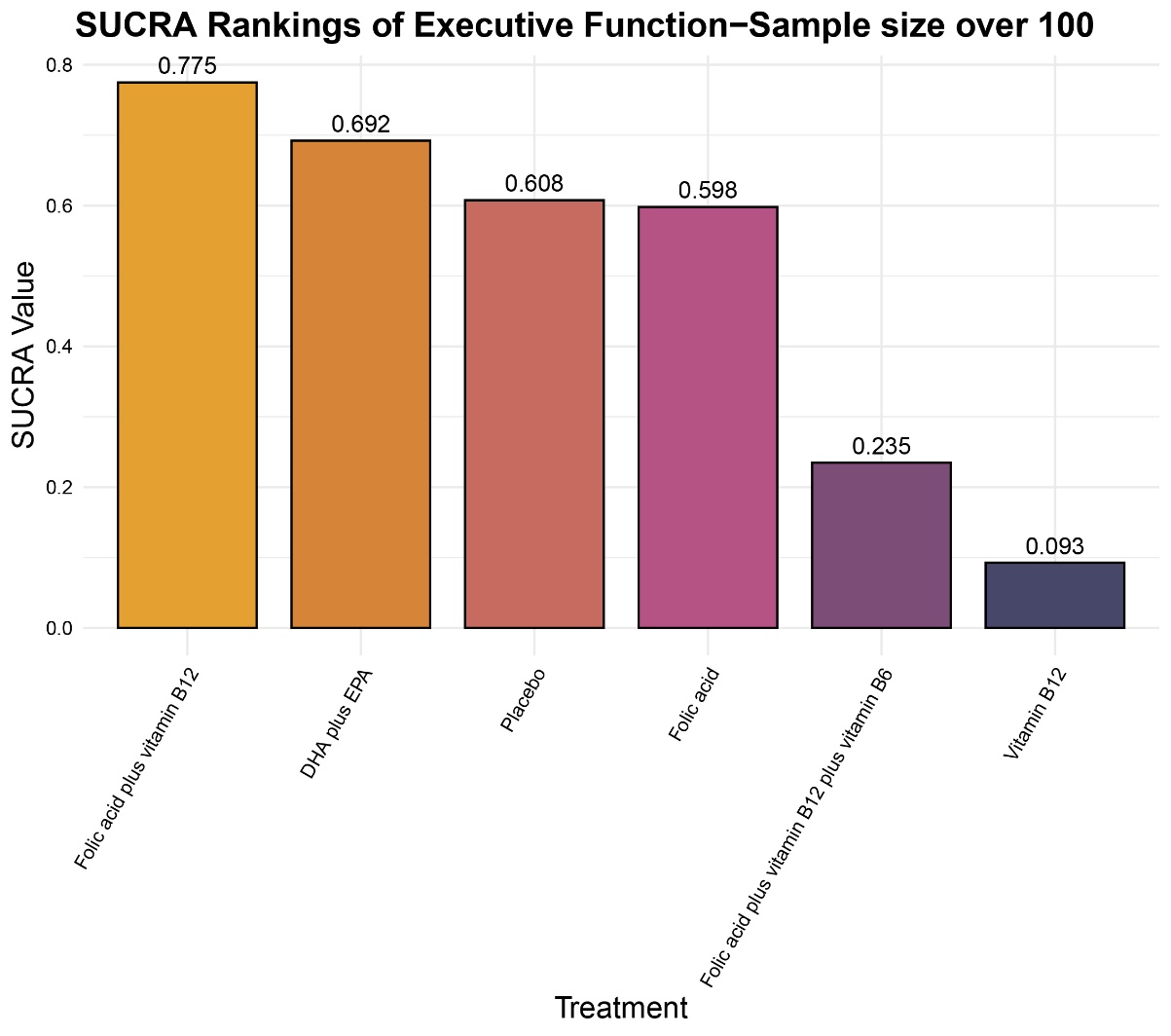


### eFigure 13C Executive function (Subgroup analysis-Sample size over 100): Ranking forest plot and ranking probability


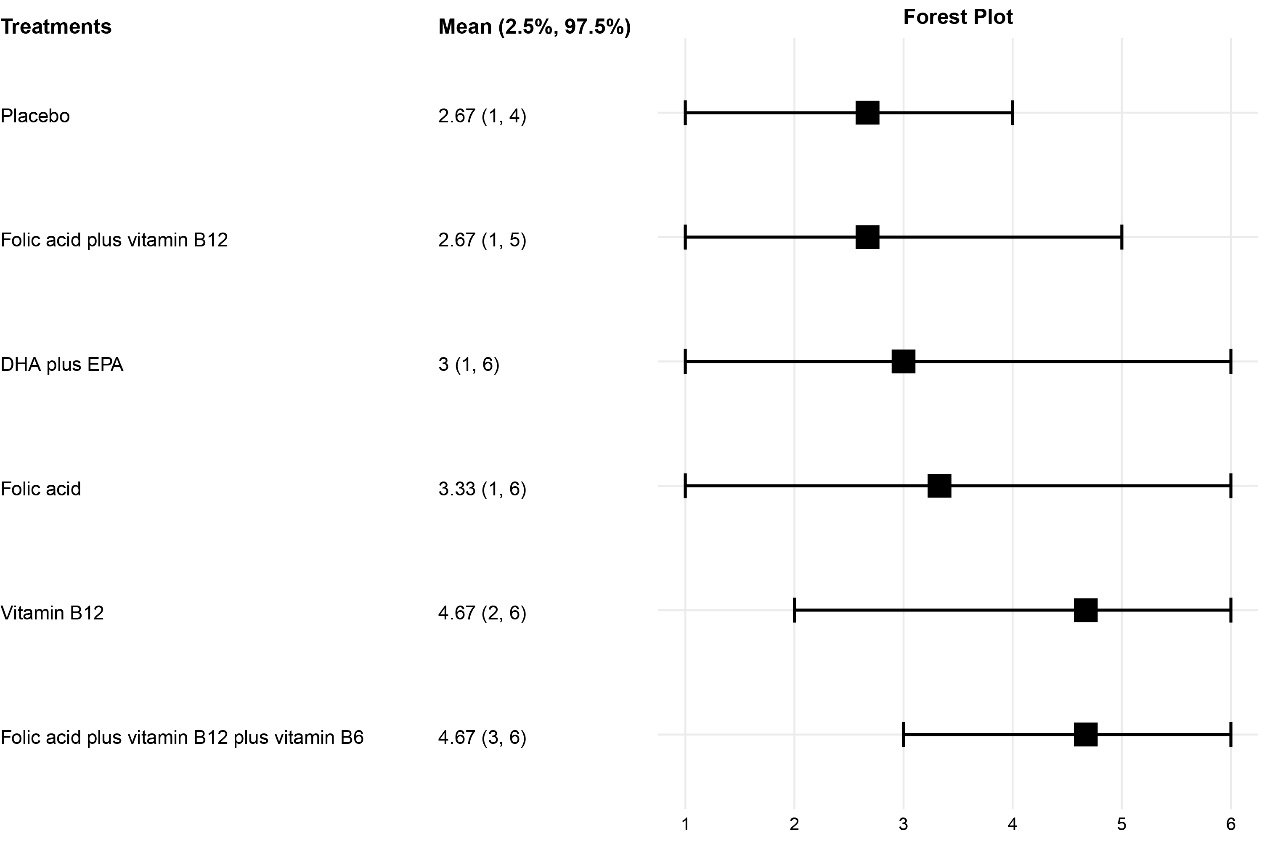


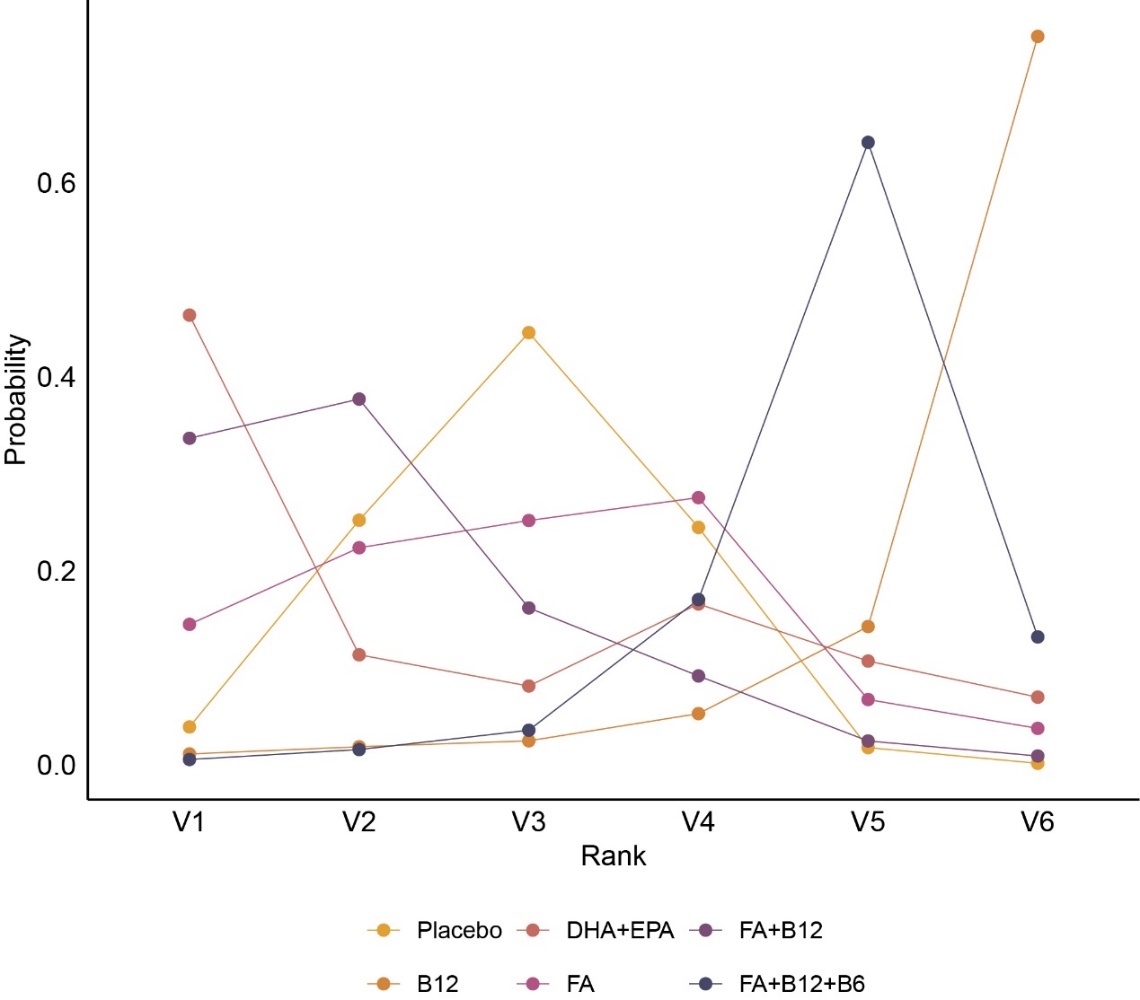


## Part Ⅳ Cognitive function assessment: Memory

### eFigure 14A Memory (All): Node splitting analysis


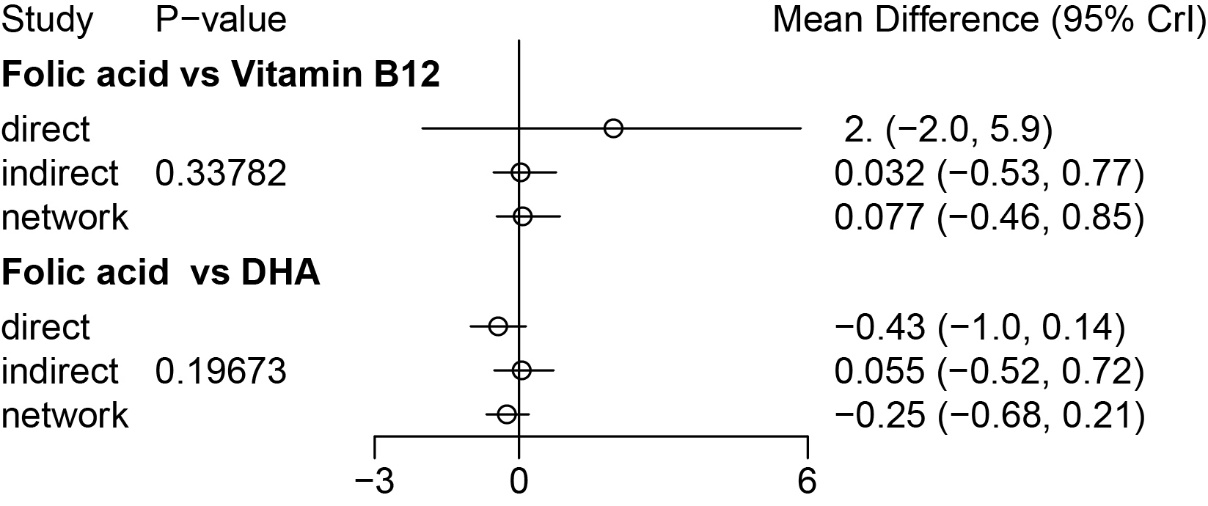


### eFigure 14B Memory (All): Bland Altman analysis


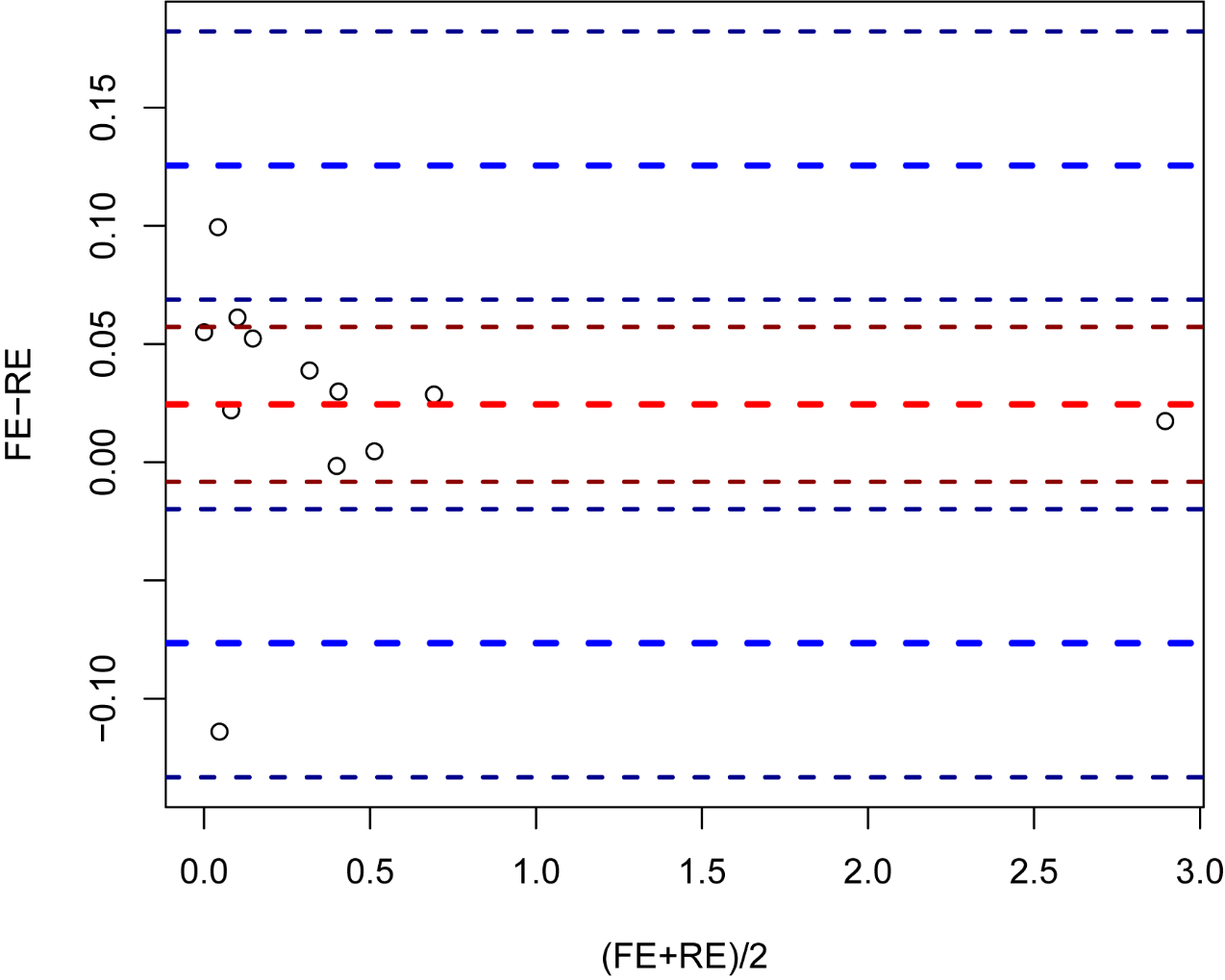


### eFigure 14C Memory (All): SUCRA plot


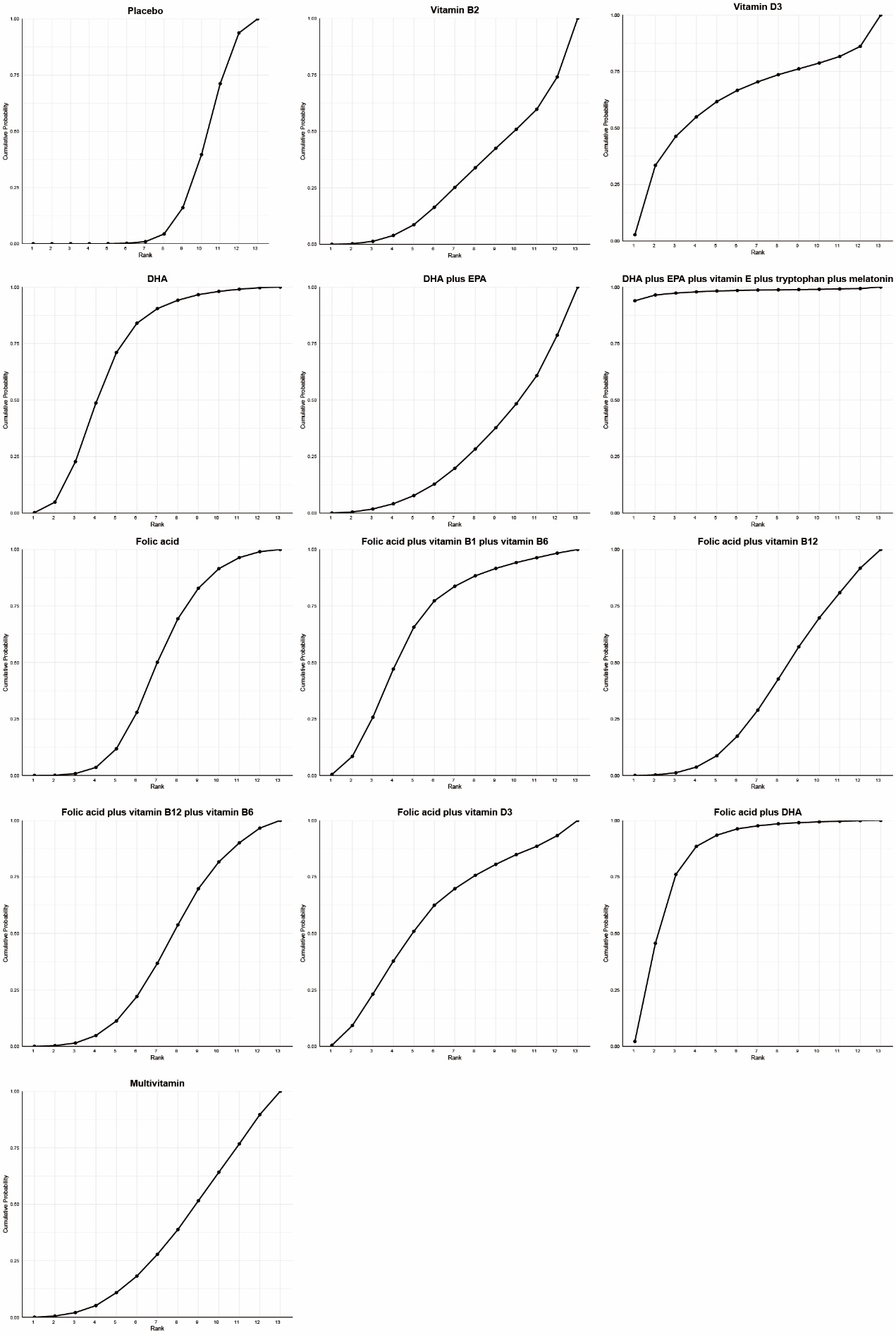


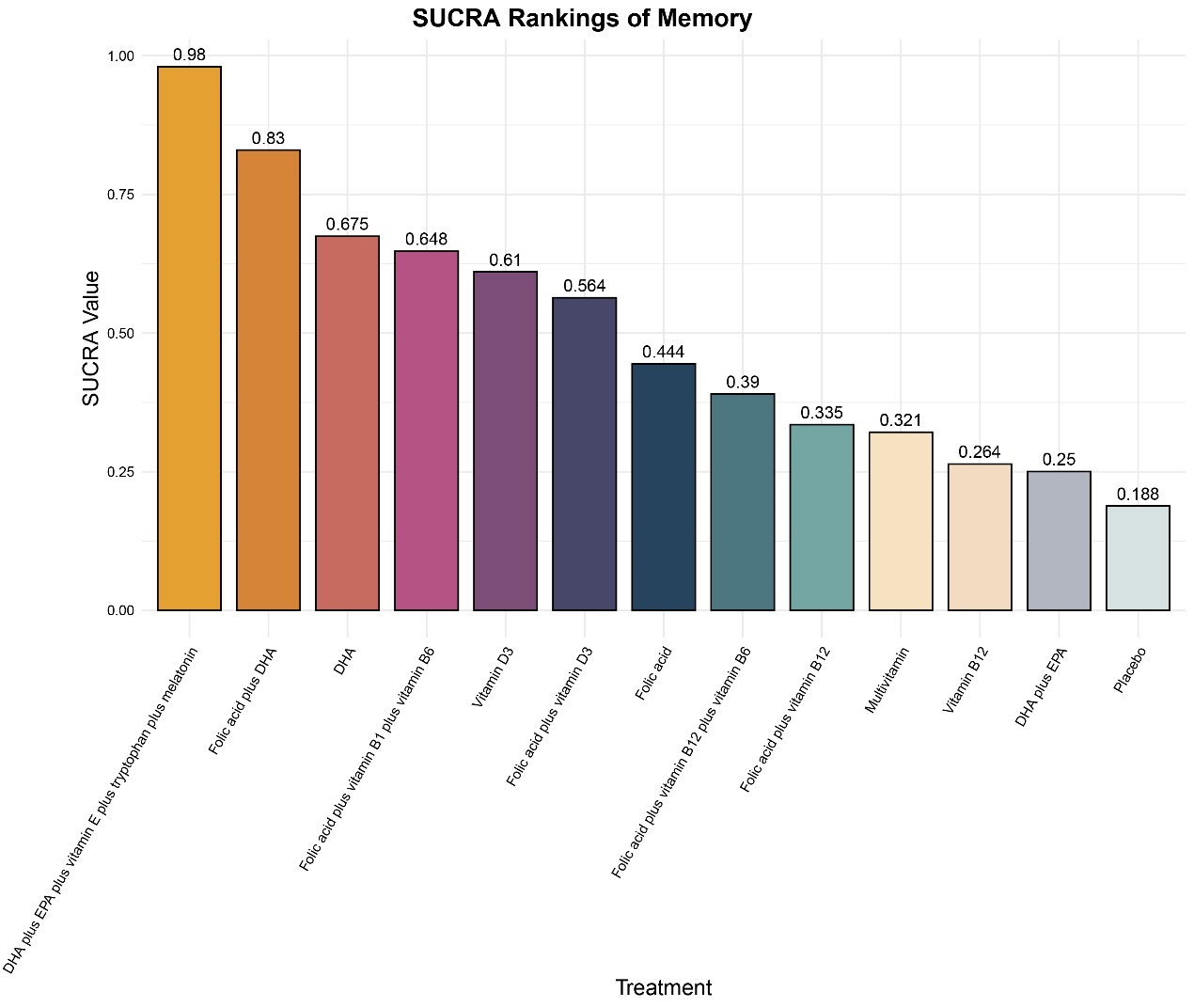


### eFigure 14D Memory (All): Ranking forest plot and ranking probability


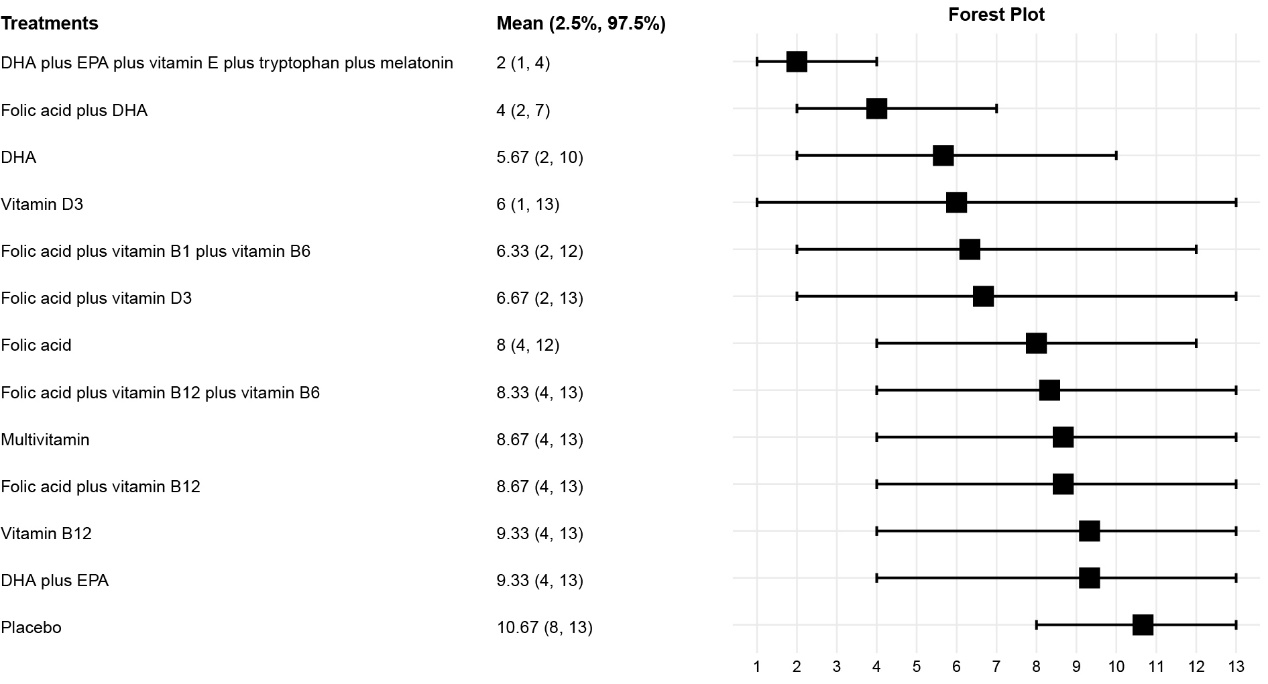


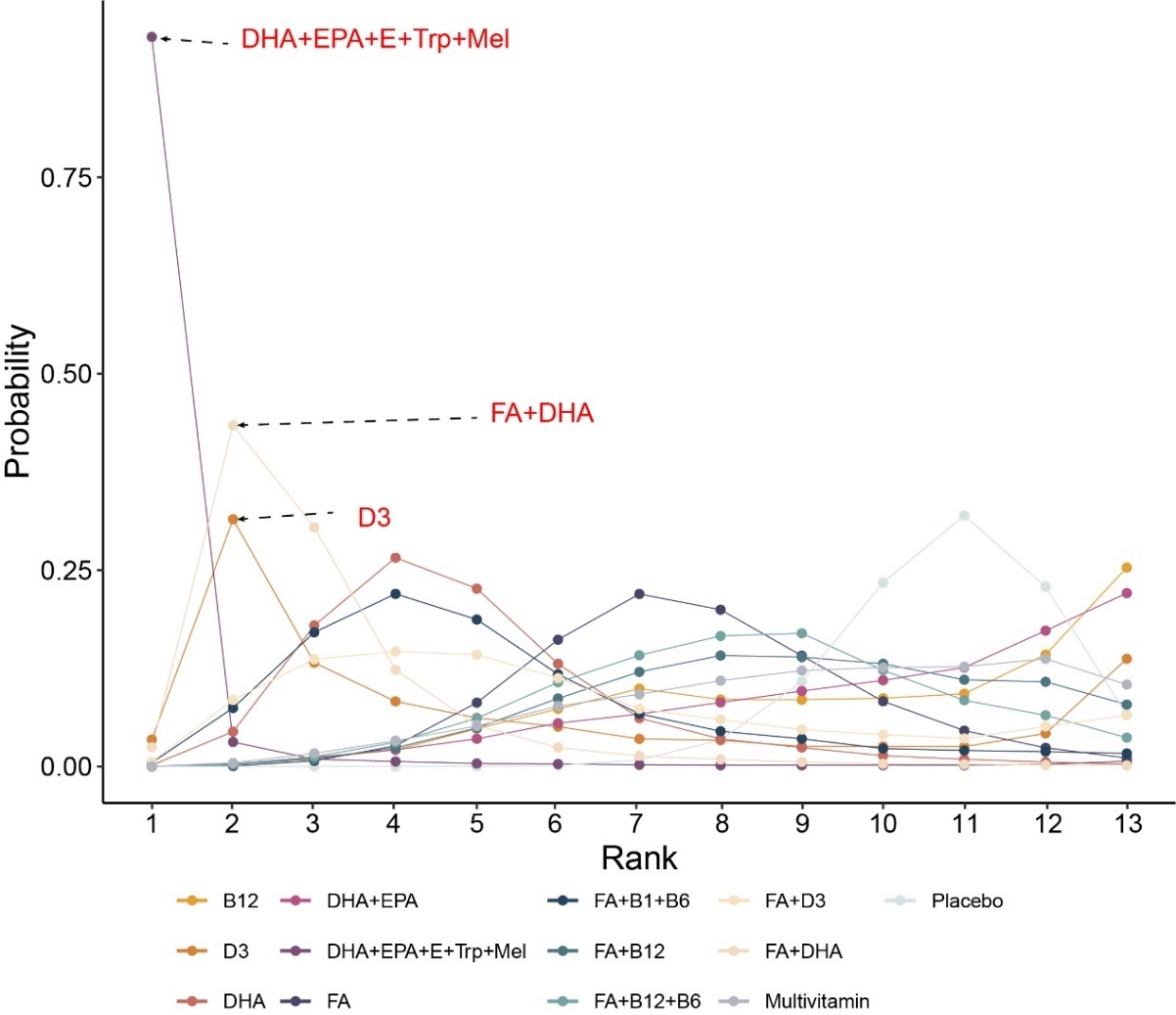


### eFigure 15A Memory (Subgroup analysis-Age): A, Network plot; B, funnel plot; C, forest plot.


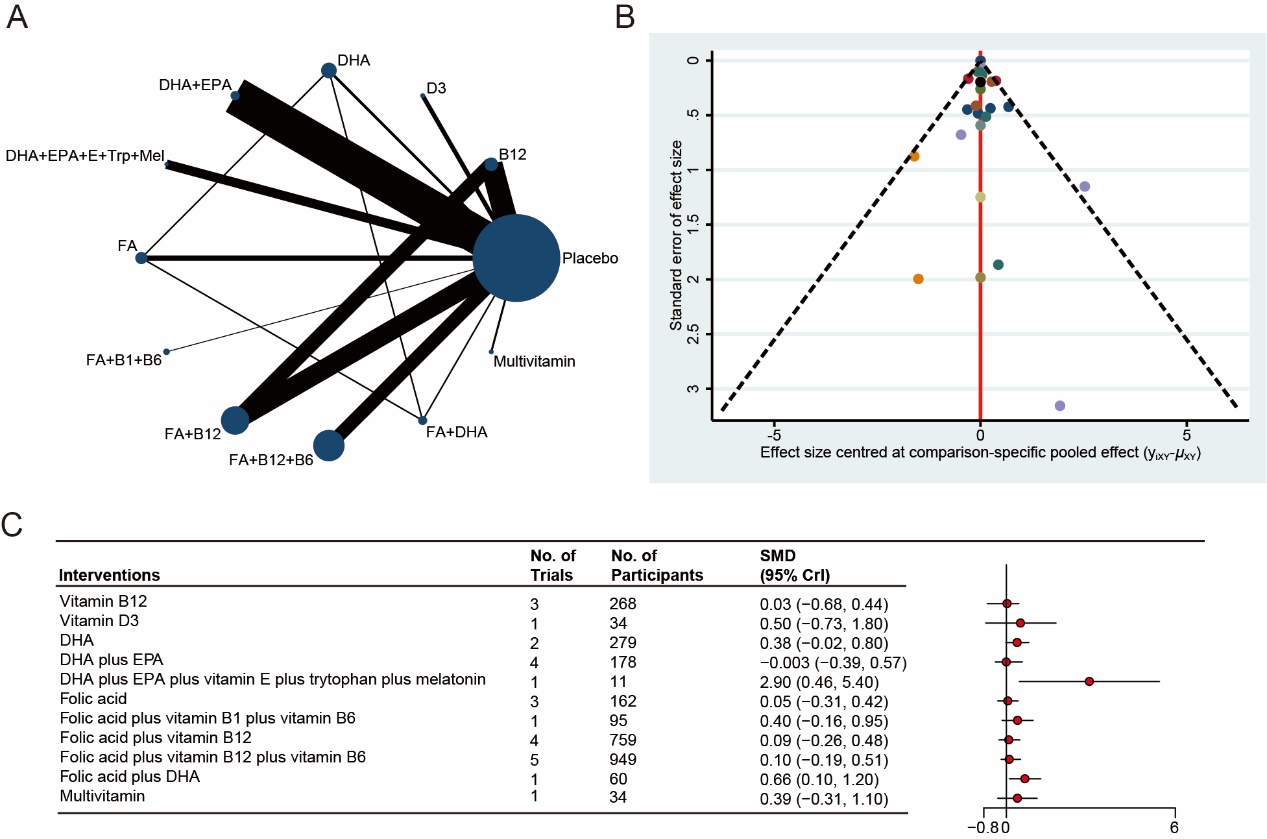


### eFigure 15B Memory (Subgroup analysis-Age): Node splitting analysis


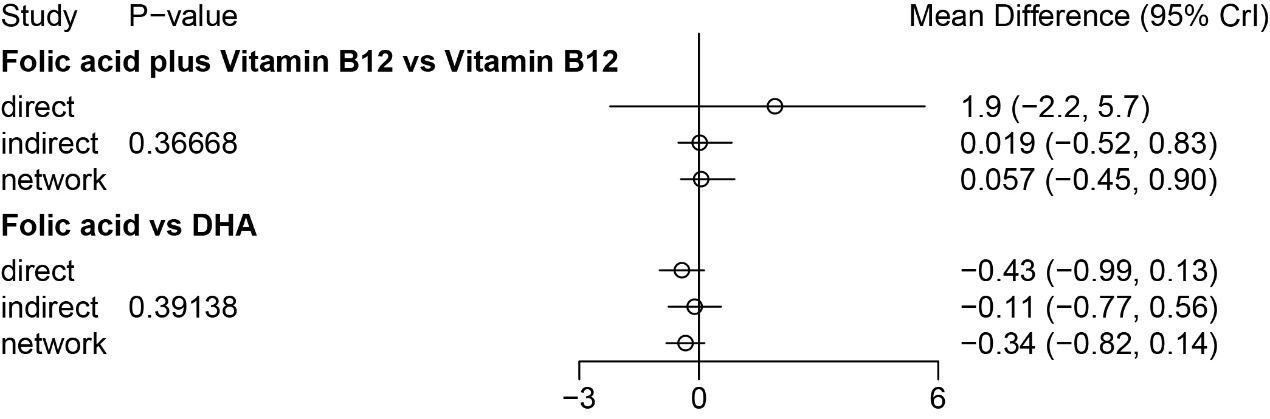


### eFigure 15C Memory (Subgroup analysis-Age): SUCRA plot


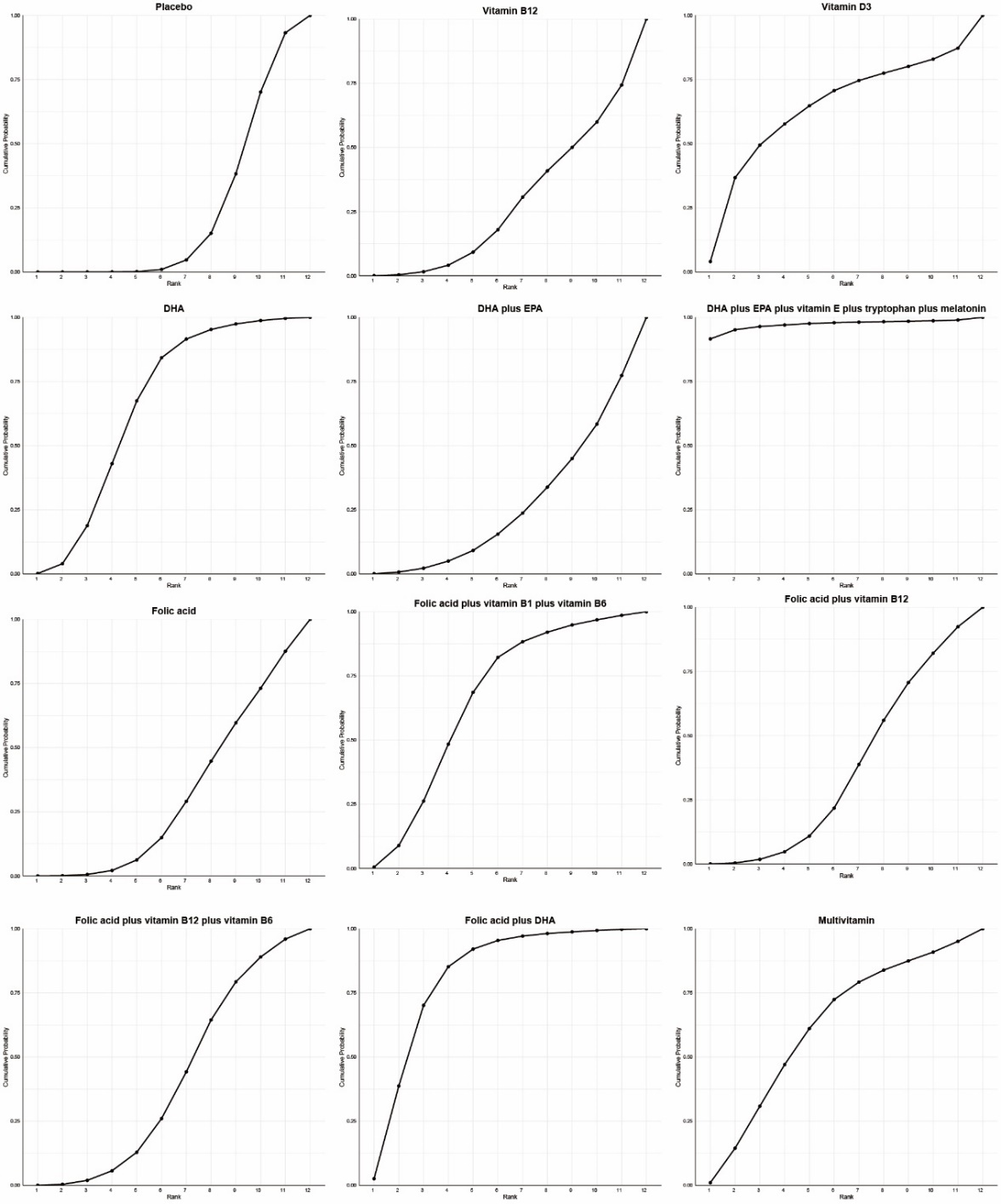


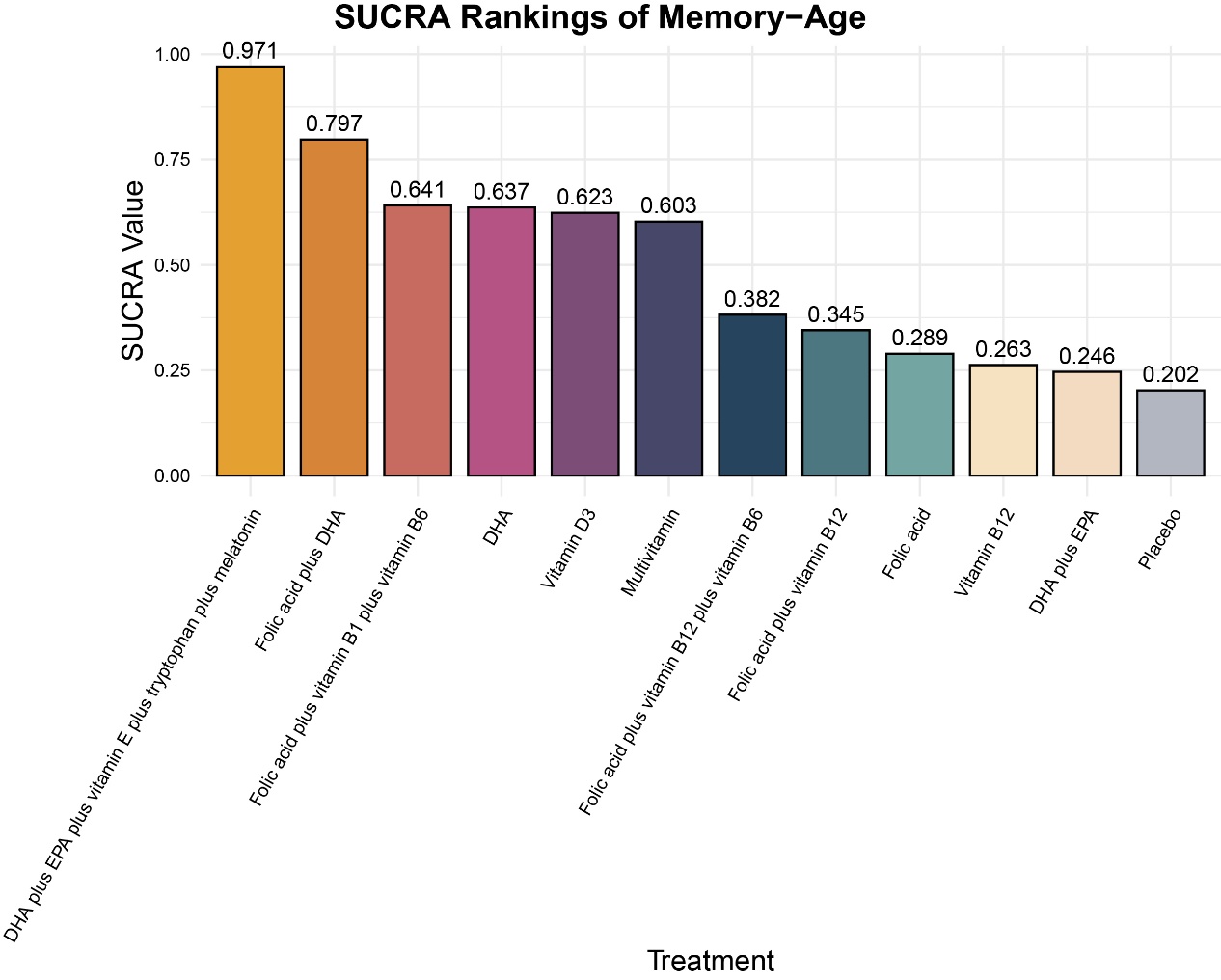


### eFigure 15D Memory (Subgroup analysis-Age): Ranking forest plot and ranking probability


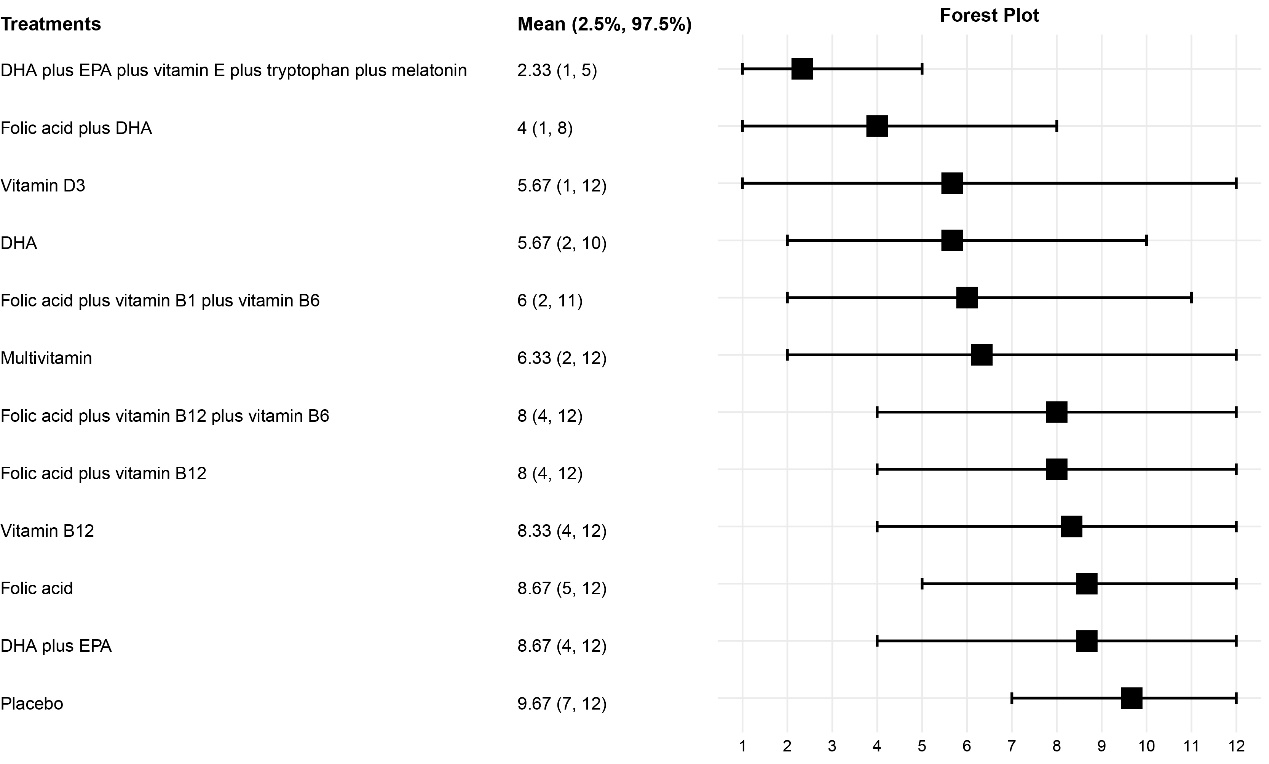


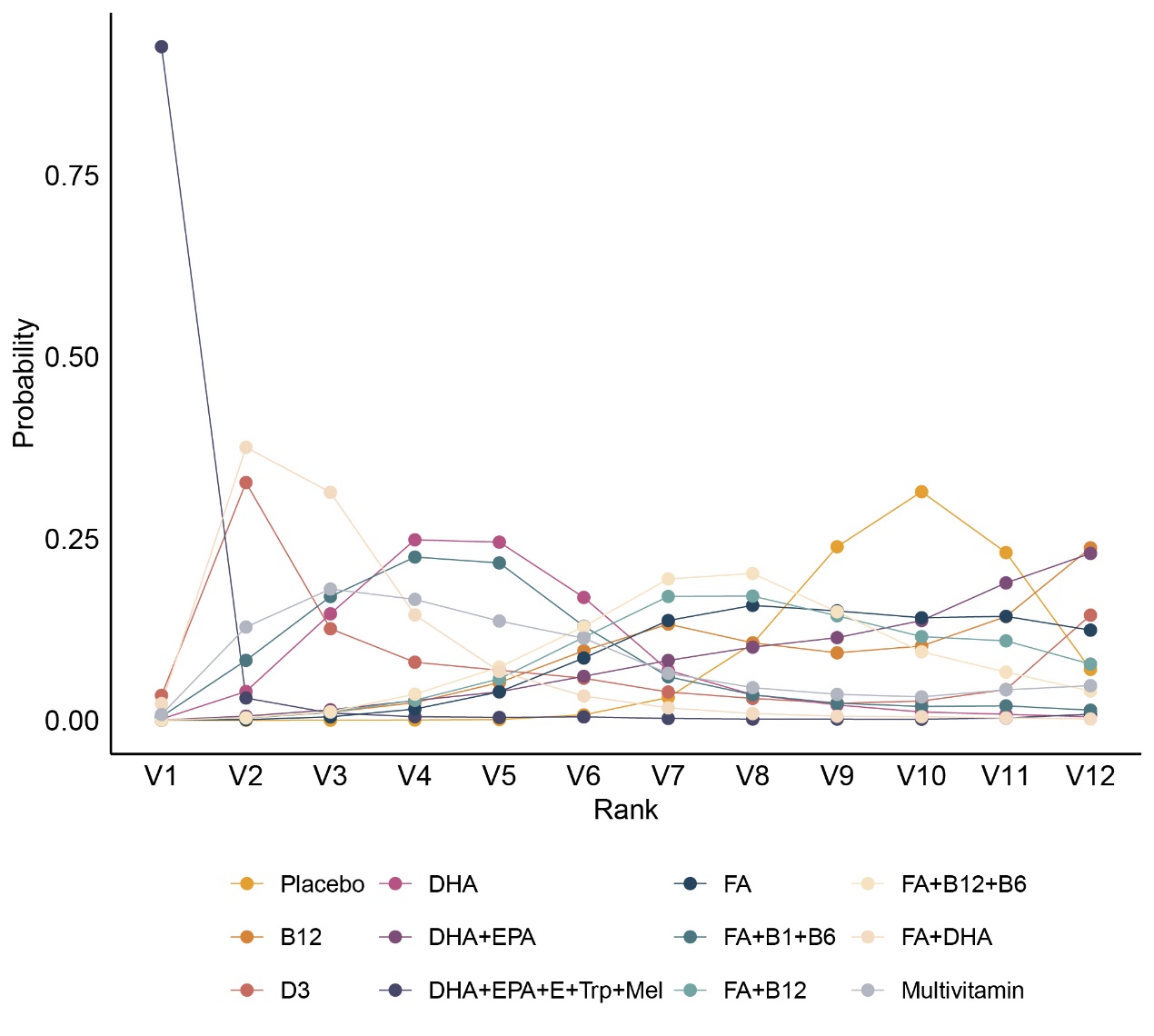


### eFigure 16A Memory (Subgroup analysis-MCI): A, Network plot; B, funnel plot; C, forest plot.


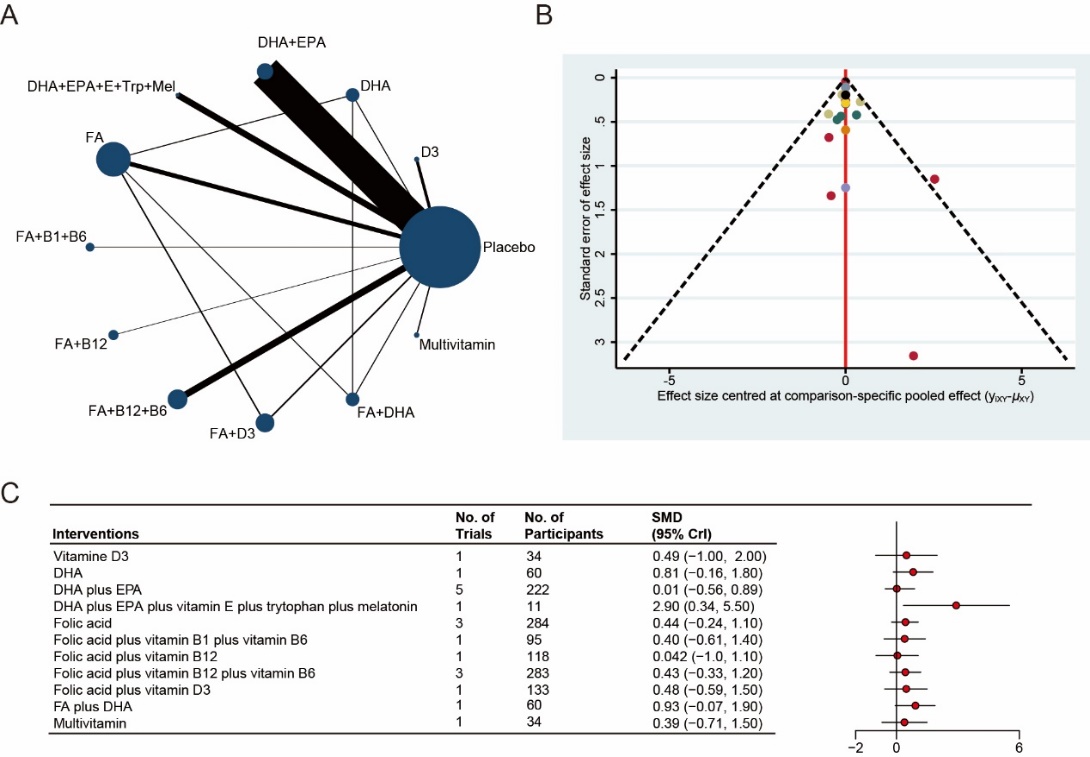


### eFigure 16B Memory (Subgroup analysis-MCI): SUCRA plot


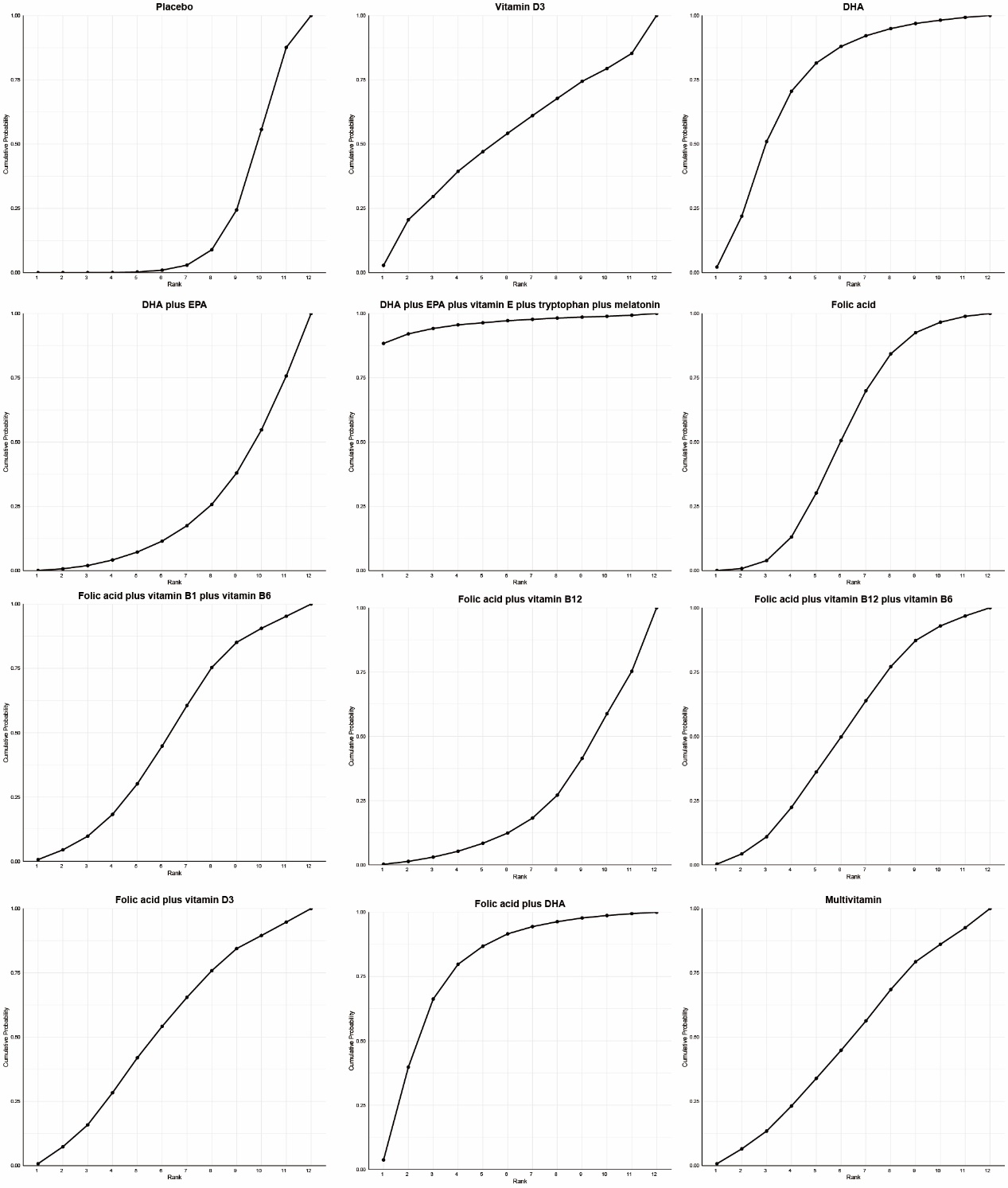


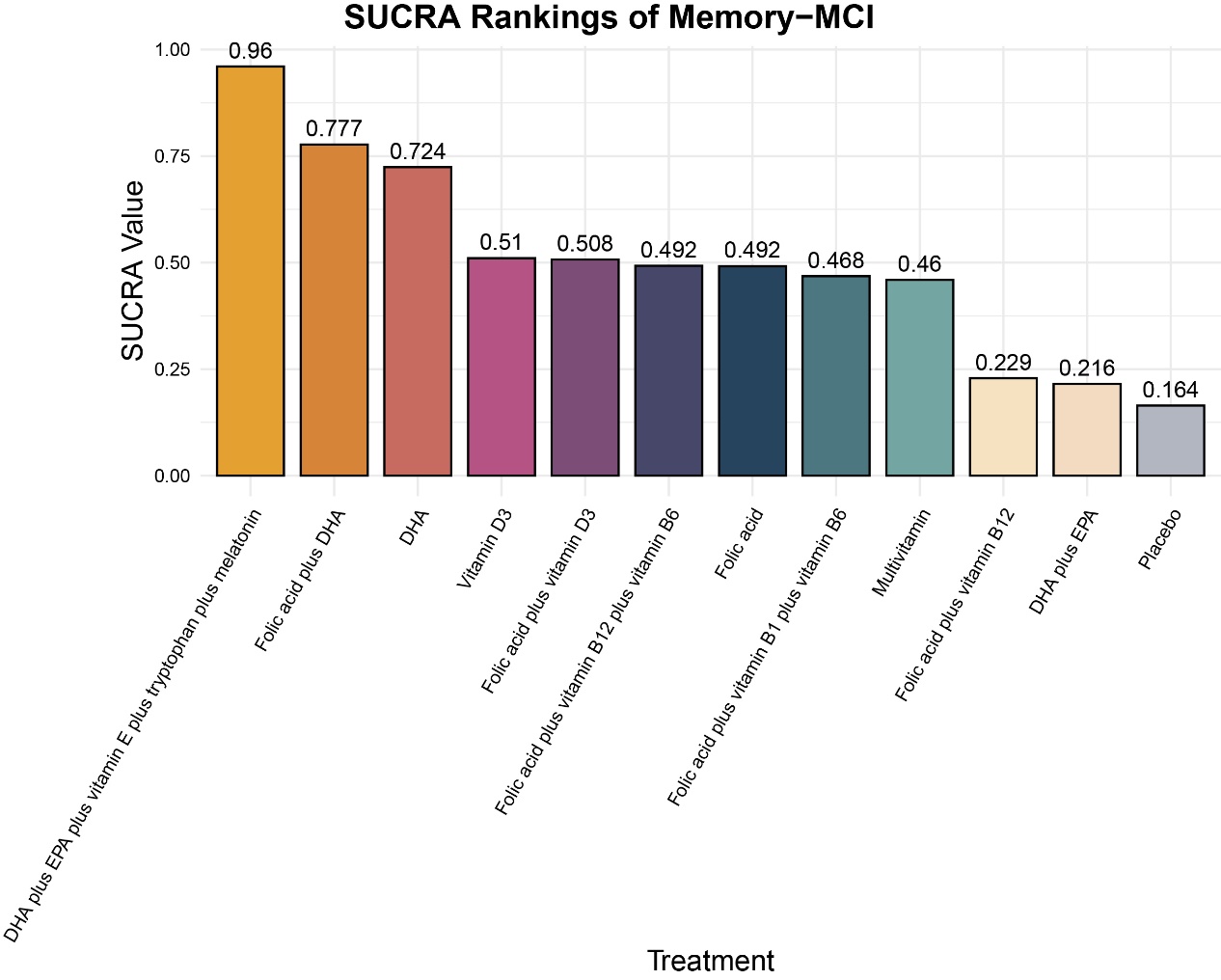


### eFigure 16C Memory (Subgroup analysis-MCI): Ranking forest plot and ranking probability


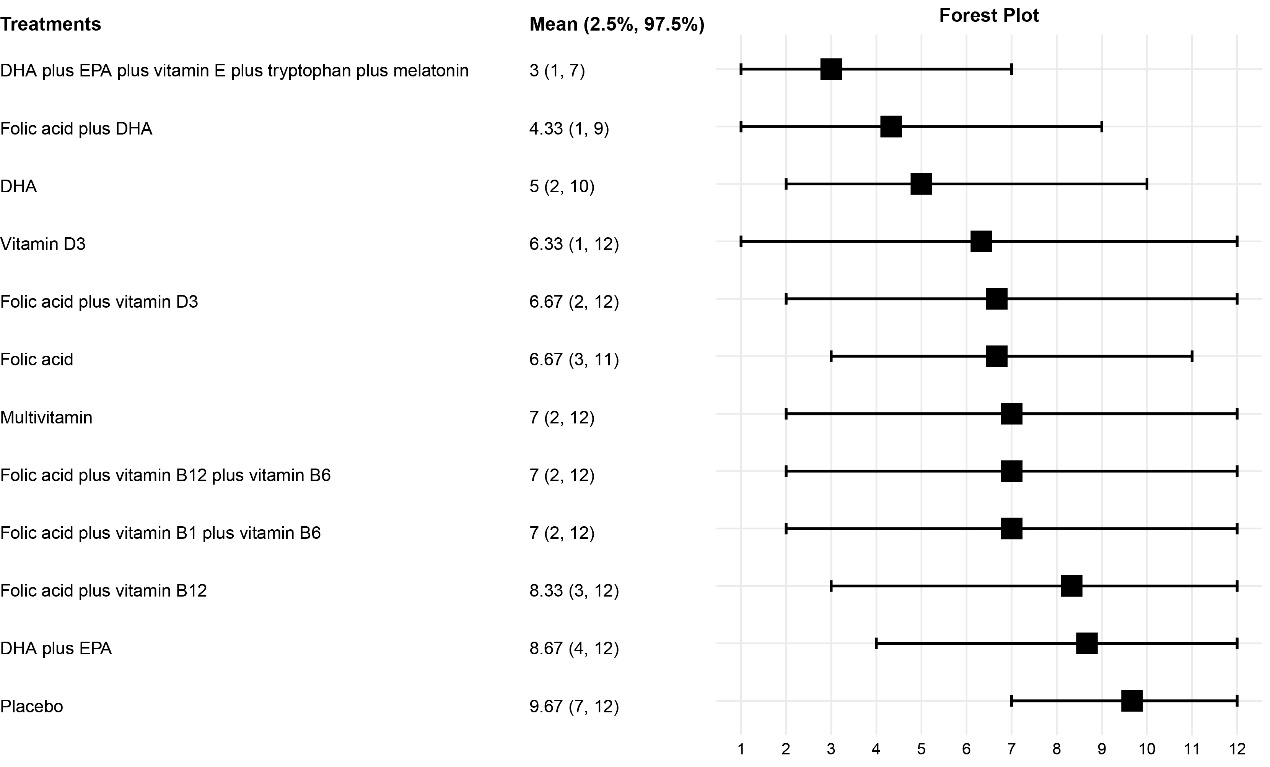


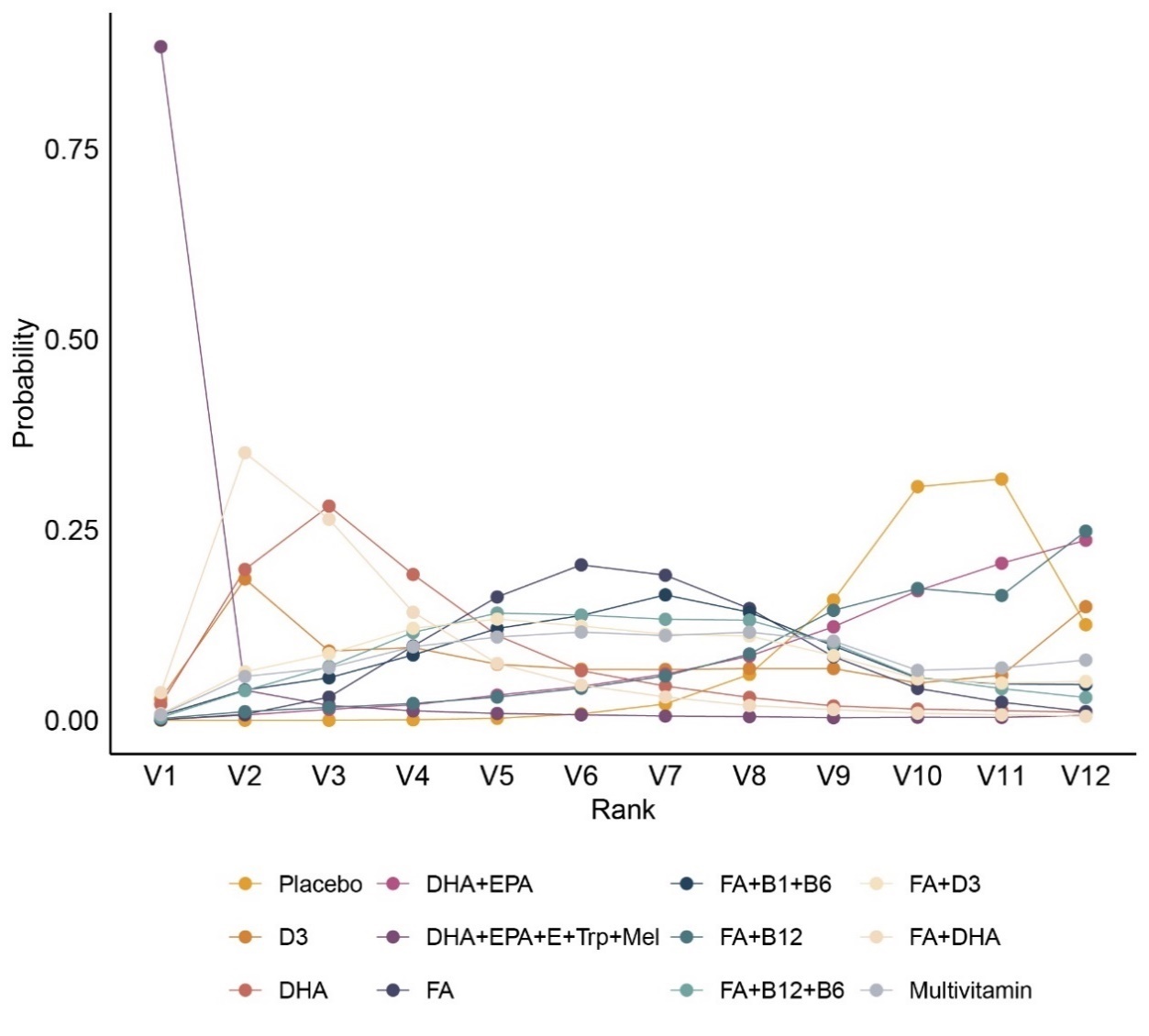


### eFigure 17A Memory (Subgroup analysis-Sample size over 100): A, Network plot; B, funnel plot; C, forest plot.


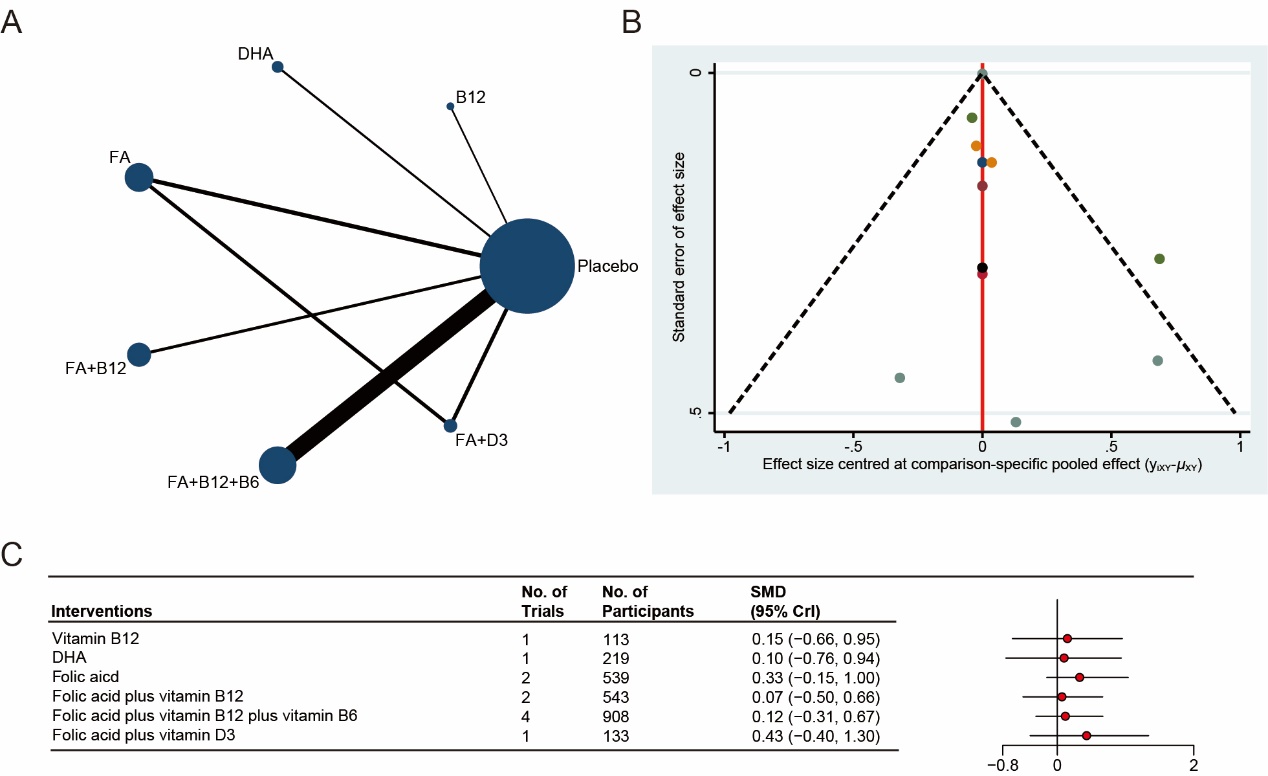


### eFigure 17B Memory (Subgroup analysis-Sample size over 100): SUCRA plot


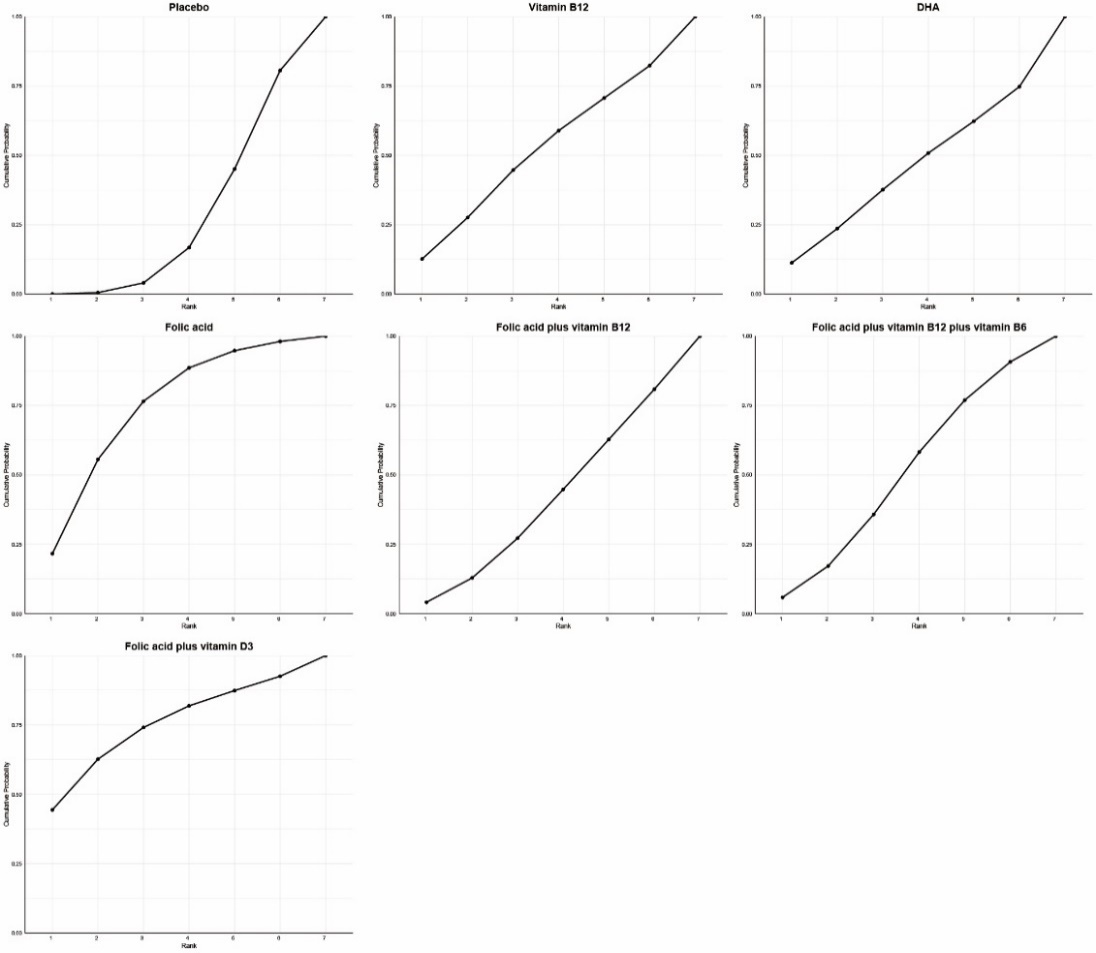


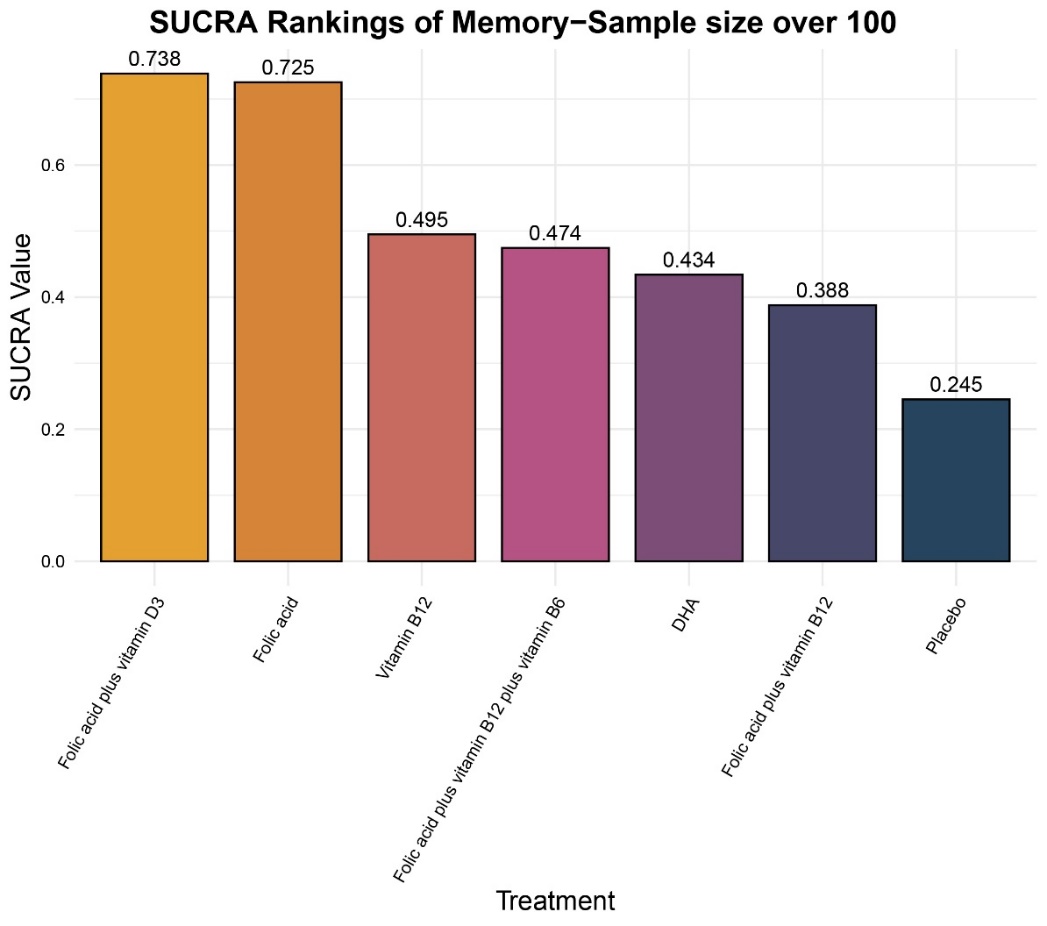


### eFigure 17C Memory (Subgroup analysis-Sample size over 100): Ranking forest plot and ranking probability


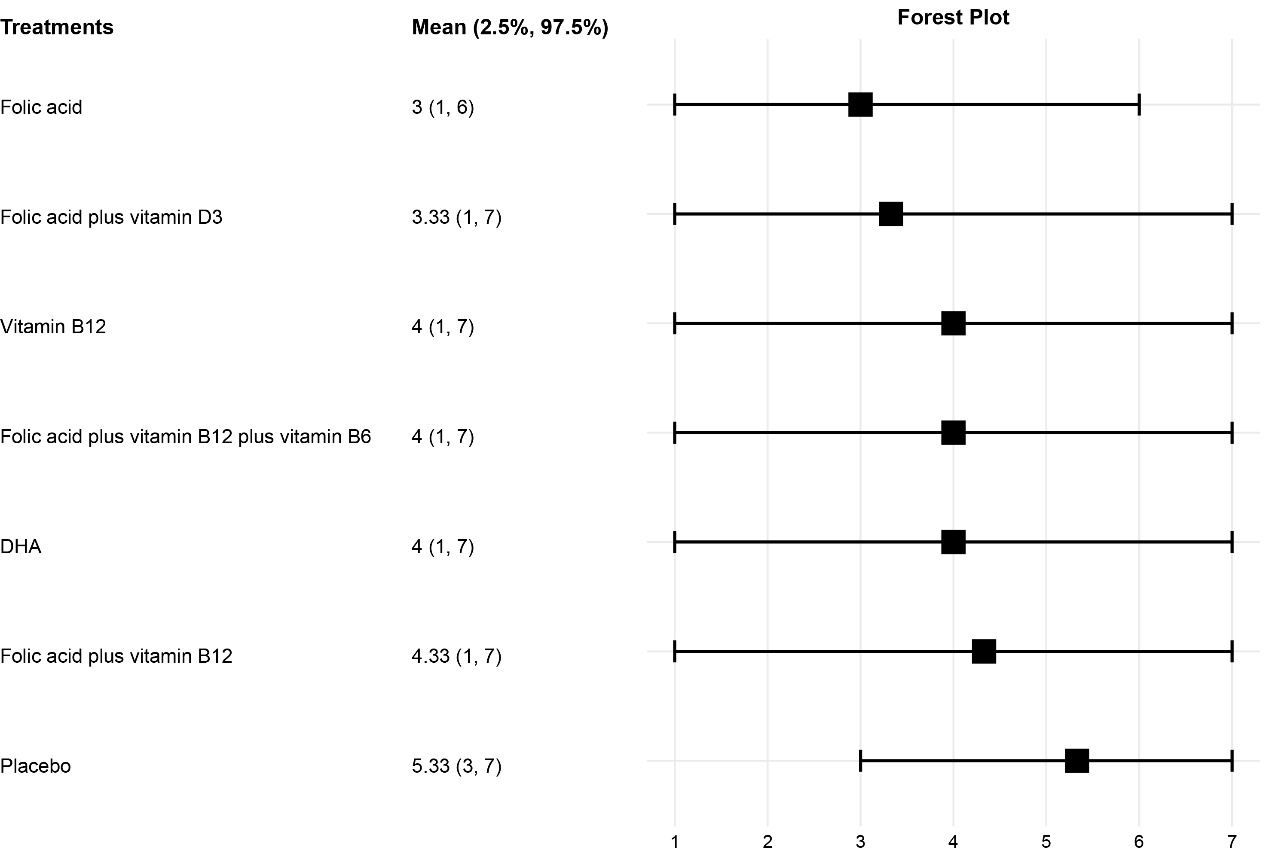


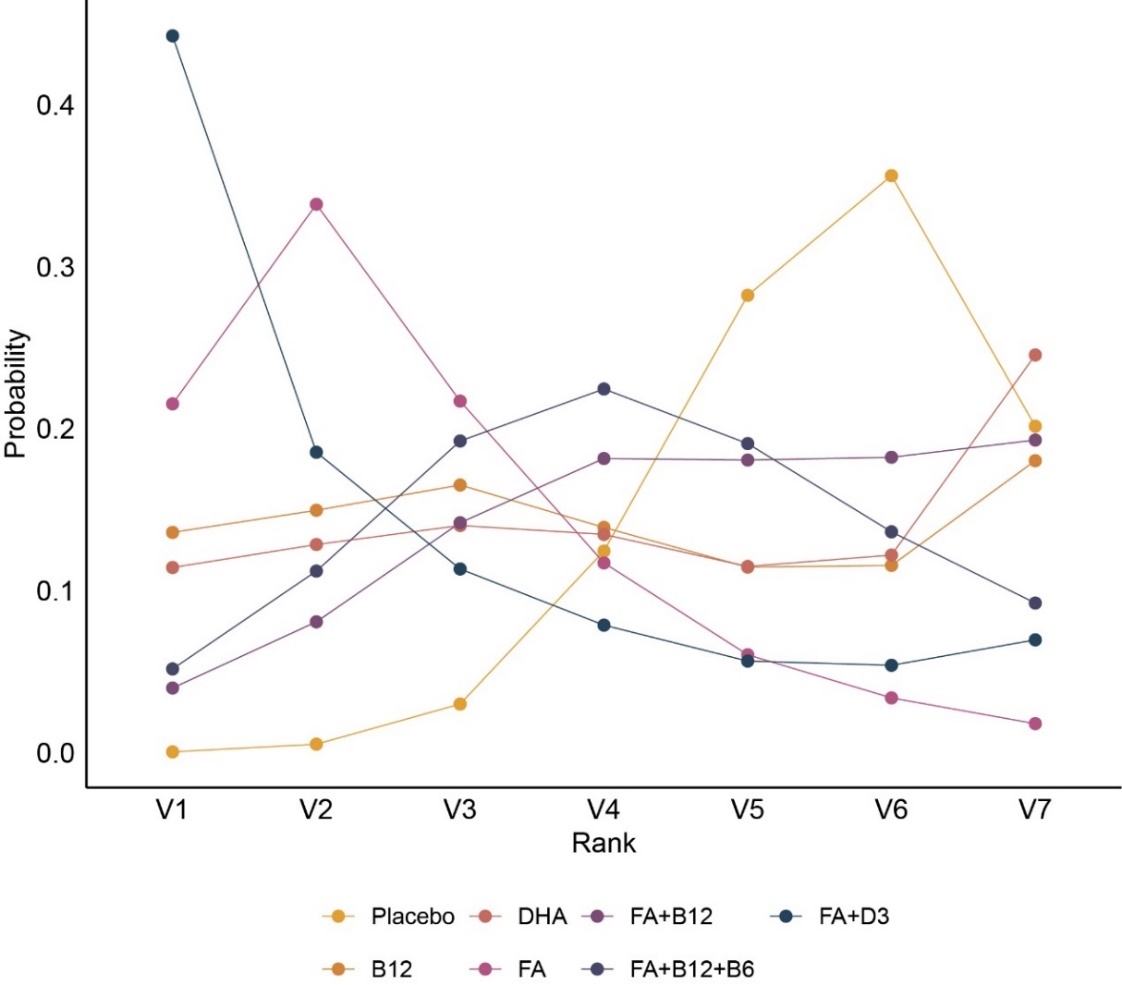


## Part Ⅴ Cognitive function assessment: Processing speed

### eFigure 18A Processing speed (All): Node splitting analysis


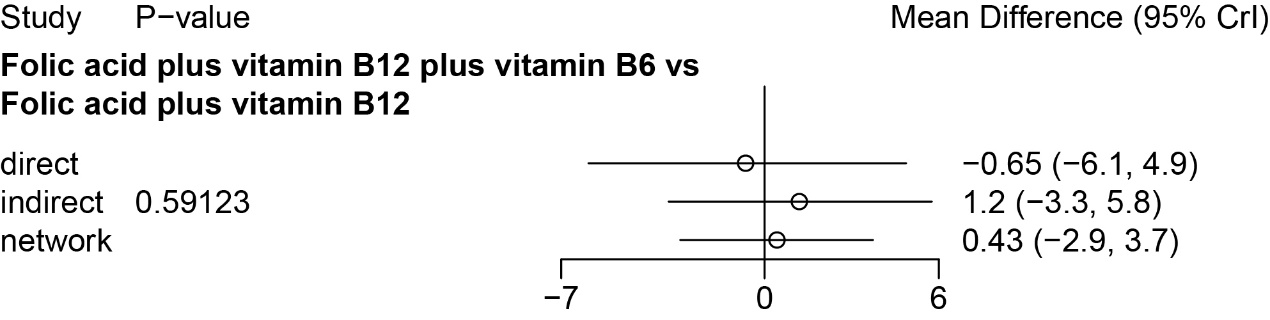


### eFigure 18B Processing speed (All): Bland Altman analysis

### eFigure 18C Processing speed (All): SUCRA plot

### eFigure 18D Processing speed (All): Ranking forest plot and ranking probability

### eFigure 19A Processing speed (Subgroup analysis-Age): A, Network plot; B, funnel plot; C, forest plot.

### eFigure 19B Processing speed (Subgroup analysis-Age): SUCRA plot

### eFigure 19C Processing speed (Subgroup analysis-Age): Ranking forest plot and ranking probability

### eFigure 20A Processing speed (Subgroup analysis-MCI): A, Network plot; B, funnel plot; C, forest plot.

### eFigure 20B Processing speed (Subgroup analysis-MCI): SUCRA plot

### eFigure 20C Processing speed (Subgroup analysis-MCI): Ranking forest plot and ranking probability

### eFigure 21A Processing speed (Subgroup analysis-Sample size over 100): A, Network plot; B, funnel plot; C, forest plot.

### eFigure 21B Processing speed (Subgroup analysis-Sample size over 100): SUCRA plot

### eFigure 21C Processing speed (Subgroup analysis-Sample size over 100): Ranking forest plot and ranking probability

## Part Ⅵ Cognitive function assessment: Visuospatial function

### eFigure 22A Visuospatial function (All): Node splitting analysis

### eFigure 22B Visuospatial function (All): Bland Altman analysis

### eFigure 22C Visuospatial function (All): SUCRA plot

### eFigure 22D Visuospatial function (All): Ranking forest plot and ranking probability

### eFigure 23A Visuospatial function (Subgroup analysis-Age): A, Network plot; B, funnel plot; C, forest plot.

### eFigure 23B Visuospatial function (Subgroup analysis-Age): SUCRA plot

.

### eFigure 23C Visuospatial function (Subgroup analysis-Age): Ranking forest plot and ranking probability.

### eFigure 24A Visuospatial function (Subgroup analysis-MCI): A, Network plot; B, funnel plot; C, forest plot.

### eFigure 24B Visuospatial function (Subgroup analysis-MCI): SUCRA plot

### eFigure 24C Visuospatial function (Subgroup analysis-MCI): Ranking forest plot and ranking probability

### eFigure 25A Visuospatial function (Subgroup analysis-Sample size over 100): A, Network plot; B, funnel plot; C, forest plot.

### eFigure 25B Visuospatial function (Subgroup analysis-Sample size over 100): SUCRA plot

### eFigure 25C Visuospatial function (Subgroup analysis-Sample size over 100): Ranking forest plot and ranking probability

## Part Ⅶ Biochemical analysis: Homocysteine

### eFigure 26A Homocysteine: Node splitting analysis

### eFigure 26B Homocysteine: SUCRA plot

### eFigure 26C Homocysteine: Ranking forest plot and ranking probability

## Part Ⅷ Biochemical analysis: Vitamin B12

### eFigure 27A Vitamin B12: SUCRA plot

### eFigure 27B Vitamin B12: Ranking forest plot and ranking probability

## Part Ⅸ Biochemical analysis: Serum folate

### eFigure 28A Serum-folate: SUCRA plot

### eFigure 28B Serum-folate: Ranking forest plot and ranking probability

## Part Ⅹ Biochemical analysis: Erythrocyte folate

### eFigure 29A Erythrocyte folate: SUCRA plot

### eFigure 29B Erythrocyte folate: Ranking forest plot and ranking probability

## Part Ⅺ Sensitivity analysis

**eFigure 30. Sensitivity analysis of nutritional supplementation efficacy across cognitive domains (intervention duration ≥ 6 months).** The network meta-analysis highlights the comparative effectiveness of various interventions when restricted to studies with a duration of at least six months. (A, C, E, G, I) Network evidence plots for global cognition, attention, executive function, memory, and visuospatial function, respectively. The size of the nodes represents the total number of participants for each intervention, while the thickness of the edges corresponds to the number of randomized controlled trials comparing the two connected interventions. (B, D, F, H, J) SUCRA (Surface Under the Cumulative Ranking) value plots for the corresponding cognitive domains. Higher SUCRA values indicate a greater probability that an intervention is among the most effective for a specific domain. Notable findings include the sustained high ranking of melatonin for global cognition (B), DHA for attention (D), the combination of DHA and EPA for executive function (F), folic acid combined with DHA for memory (H), and vitamin D3 for visuospatial function (J).

References

1. Luo D, Wan X, Liu J, Tong T. Optimally estimating the sample mean from the sample size, median, mid-range, and/or mid-quartile range. *Statistical methods in medical research.* 2018;27(6):1785-1805.

2. Wan X, Wang W, Liu J, Tong T. Estimating the sample mean and standard deviation from the sample size, median, range and/or interquartile range. *BMC medical research methodology.* 2014;14:135.
